# CCHCR1-astrin interaction promotes centriole duplication through recruitment of CEP72

**DOI:** 10.1186/s12915-022-01437-6

**Published:** 2022-10-24

**Authors:** Zhenguang Ying, Kaifang Wang, Junfeng Wu, Mingyu Wang, Jing Yang, Xia Wang, Guowei Zhou, Haibin Chen, Hongwu Xu, Stephen Cho Wing Sze, Feng Gao, Chunman Li, Ou Sha

**Affiliations:** 1 Department of Anatomy, Histology and Developmental Biology, Shenzhen University Health Science Centre, Shenzhen, 518000 China; 2 Medical AI Laboratory, School of Biomedical Engineering, Shenzhen University Health Science Centre, Shenzhen, 518000 China; 3 Shenzhen University Health Science Centre, Shenzhen, 518000 China; 4 Department of Histology and Embryology, Shantou University Medical College, Shantou, 515000 China; 5 Department of Neurosurgery, The First Affiliated Hospital of Shantou University Medical College, Shantou, 515000 China; 6 Department of Clinically Oriented Anatomy, Shantou University Medical College, Shantou, 515000 China; 7 Department of Biology, Faculty of Science, Hong Kong Baptist University, Hongkong, 999077 China; 8 Golden Meditech Centre for NeuroRegeneration Sciences, Hong Kong Baptist University, Hongkong, 999077 China; 9 School of Dentistry, Shenzhen University Health Science Centre, Shenzhen, 518000 China; 10 Department of Anatomy, Shantou University Medical College, Shantou, 515000 China; 11 Guangdong Provincial Key Laboratory of Infectious Diseases and Molecular Immunopathology, Shantou University Medical College, Shantou, 515000 China

**Keywords:** CCHCR1, Astrin, CEP72, Centrosome, Mitosis, Microtubule organization

## Abstract

**Background:**

The centrosome is one of the most important non-membranous organelles regulating microtubule organization and progression of cell mitosis. The coiled-coil alpha-helical rod protein 1 (CCHCR1, also known as HCR) gene is considered to be a psoriasis susceptibility gene, and the protein is suggested to be localized to the P-bodies and centrosomes in mammalian cells. However, the exact cellular function of HCR and its potential regulatory role in the centrosomes remain unexplored.

**Results:**

We found that HCR interacts directly with astrin, a key factor in centrosome maturation and mitosis. Immunoprecipitation assays showed that the coiled-coil region present in the C-terminus of HCR and astrin respectively mediated the interaction between them. Astrin not only recruits HCR to the centrosome, but also protects HCR from ubiquitin-proteasome-mediated degradation. In addition, depletion of either HCR or astrin significantly reduced centrosome localization of CEP72 and subsequent MCPH proteins, including CEP152, CDK5RAP2, and CEP63. The absence of HCR also caused centriole duplication defects and mitotic errors, resulting in multipolar spindle formation, genomic instability, and DNA damage.

**Conclusion:**

We conclude that HCR is localized and stabilized at the centrosome by directly binding to astrin. HCR are required for the centrosomal recruitment of MCPH proteins and centriolar duplication. Both HCR and astrin play key roles in keeping normal microtubule assembly and maintaining genomic stability.

**Supplementary Information:**

The online version contains supplementary material available at 10.1186/s12915-022-01437-6.

## Background

Microtubules constitute an essential part of the cytoskeleton, maintaining cell shape and regulating mitosis [1]. During mitosis, microtubules extend from the centrioles, forming a spindle [2–4]. As the microtubular organization center, the centrosome is composed of a pair of centrioles and pericentriolar materials (PCM, also known as pericentriolar satellites) [5, 6]. Centrioles that display polar barrel-shaped structures with radial symmetry play a key role in the organization of centrosomes [6]. The number of centrioles in a cell is strictly regulated by the cell cycle. In the G1 phase, there is only one centrosome, which contains two isolated centrioles. PCM proteins are gradually recruited to the centrioles as the cell enters the S phase, and new procentrioles are formed at the proximal end of the existing centrioles. During the G2 phase, two centrosomes appear after duplication, and each contains two closely attached centrioles, which ensures that the daughter cells receive one centrosome with two centrioles after mitosis [7]. The PCM consists of various proteins, including pericentriolar materials 1 (PCM1), pericentrin, and a large number of centrosomal protein (CEP) family, such as CEP152, CEP63, and CEP215 (also named as cyclin-dependent kinase 5 regulatory subunit-associated protein 2 (CDK5RAP2)) [8]. These CEPs are not called a family in terms of homology, but they are all located in centrosomes, some of which are near the centriole and others are located in the outer part of the PCM, and perform different functions [9]. This complex structure of multiple, intertwined proteins is considered a platform for regulating organelle transport, spindle assembly, and cilia formation [10–12].

Astrin, a centrosome-related protein, which is also named sperm-associated antigen 5 (SPAG5) or mitotic spindle-associated protein p126 (MAP 126), dynamically localizes to the PCM, spindle poles, or outer kinetochores at different stages of the cell cycle. It participates in maintaining the dual-polarization of the spindle, the connection between microtubules and kinetochores, and the cohesion between sister chromatids, ensuring that mitosis proceeds properly. Deletion or mutation of astrin can lead to mitotic errors, such as spindle multi-polarization and chromosome separation failure [13–16]. In the centrosome, astrin is involved in the assembly of microcephaly (MCPH) proteins during interphase, which promotes centriole duplication [17]. The high expression of astrin is also positively correlated with the malignant degree of many tumors, indicating that its role in the centrosome is crucial [18–20].

Coiled-coil alpha-helical rod protein 1 (CCHCR1 or HCR) is a centrosome and processing body (P-body)-localized protein composed of multiple coiled-coil domains [21–23]. Although HCR has been widely reported as a susceptibility gene of psoriasis in genome-wide association studies, its function in cells is far from clear [24–27]. HCR interacts with them RNA-decapping protein 4 (EDC4) in the P-body, a special membraneless organelle dedicated to regulating mRNA decay and storage [23, 28–30]. However, the specific function of HCR in the P-body is unknown. HCR also exhibits a wide range of roles in various physiological processes, such as cell proliferation and steroid production [31, 32], and is also associated with alopecia areata, type-2 diabetes, and squamous cell carcinoma [33–35]. Interestingly, HCR has been predicted to interact with a series of centrosome- and mitosis-related proteins, such as PCM1, centrin, astrin, and CEP72, which suggests that HCR may participate in PCM networks and processes related to centrosome replication and mitosis [23].

In this study, we present evidence indicating that HCR is a key regulator of centrosome replication and microtubule organization. We show that HCR is localized and stabilized at the centrosome by directly binding to astrin. We also demonstrate that both HCR and astrin are required for the centrosome recruitment of CEP72 and MCPH proteins, including CEP152, CEP63, and CDK5RAP2. These findings provide a deeper understanding of the molecular function of HCR and are helpful for better exploring the role of HCR in psoriasis and other diseases.

## Results

### HCR interacts with spindle-associated astrin and localizes at the centrosome and spindle

**Fig. 1:**
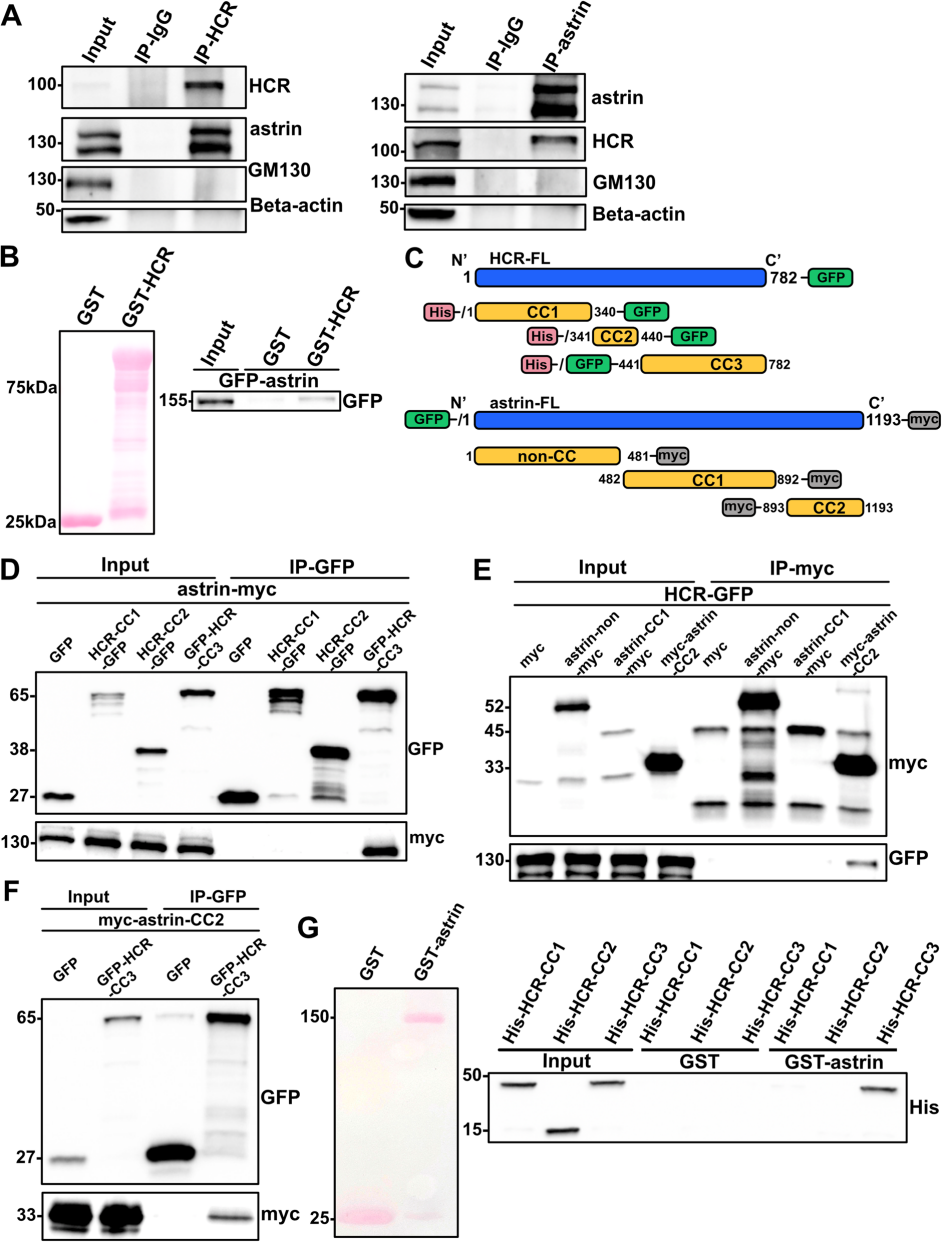
Direct interaction of HCR with astrin. **A** Reciprocal co-immunoprecipitation analysis of HCR binding to astrin. HeLa cell lysates were immunoprecipitated with astrin, HCR, or control rabbit IgG antibodies and analyzed by western blotting with anti-astrin and anti-HCR antibodies. Anti-GM130 and anti-beta actin antibodies were used as negative controls. **B** GST pull-down assay of the interaction between astrin and GST-tagged HCR. Total lysates of HeLa cells expressing GFP-astrin were incubated with GST alone or GST-HCR purified from bacterial cells. Precipitates were detected with an anti-GFP antibody. **C** Schematic models of the deletion mutants of HCR and astrin. **D** Co-immunoprecipitation analysis of the astrin-binding domain on HCR. GFP vector alone or each HCR-GFP fragment were co-transfected with myc-astrin into HeLa cells, and then, lysates were immunoprecipitated with an anti-myc antibody and analyzed by anti-myc and anti-GFP antibodies. **E** Co-immunoprecipitation analysis of the HCR-binding domain on astrin. PCMV-myc empty vector or each myc-astrin fragment was co-transfected with HCR-GFP into HeLa cells, and then, lysates were immunoprecipitated with an anti-myc antibody and analyzed by anti-GFP and anti-myc antibodies. **F** Co-immunoprecipitation analysis of the interactive domains between HCR and astrin. GFP vector alone or GFP-HCR-CC3 fragment was co-transfected with myc-astrin-CC2 into HeLa cells, and then, lysates were immunoprecipitated with an anti-myc antibody and analyzed by anti-GFP or anti-myc antibodies. **G** In vitro analysis of the domain in HCR required for interacting with astrin. GST-tagged astrin and His-tagged HCR fragments were purified from *E. coli* strain BL21(DE3), and a pull-down assay was performed to examine the astrin-binding domain in HCR

In previous reports, exogenous HCR has been found to localize to the centrosomes and P-bodies, and several P-body- and centrosome-associated proteins have been identified as candidate interactors with HCR [23]. In this study, we also examined the binding partners of CCHCR1 by proximity-dependent biotinylation (BioID)-coupled mass spectrometry (LC-MS/MS). Similar to the data reported by Ling et al., we found astrin and mRNA-decapping protein 4 (EDC4) on the identified list (Table 1). Reciprocal immunoprecipitations were performed in HeLa cells to confirm the interaction between HCR and astrin. The endogenous immunoprecipitation experiments showed that astrin and HCR bound together as they were co-precipitated (Fig. 1A, Additional file 1: Fig. S1A). In 293 cells and U2OS cells, the exogenous and endogenous immunoprecipitation experiments performed showed the same results (Additional file 1: Fig. S1B). To further investigate whether there is a direct interaction between HCR and astrin, a GST pull-down assay was performed, and the results showed that HCR directly interacted with astrin in vitro (Fig. 1B).

**Table 1:** Partial BioID-coupled LC-MS/MS results

Protein names	Gene names	Peptides	Sequence coverage [%]	MS/MS count
Coiled-coil alpha-helical rod protein 1	*CCHCR1*	82	75.8	420
Enhancer of mRNA-decapping protein 4	*EDC4*	38	39.6	71
Sperm-associated antigen 5	*SPAG5*	26	27.3	43

To map the binding sites between the two proteins, we analyzed the domains of HCR and astrin according to other studies [36, 37] and SMART Sequence Analysis Tools. Astrin consists of one unstructured region and two coiled-coil regions, whereas HCR contains three coiled-coil regions. Accordingly, we constructed a series of plasmids expressing truncated forms of HCR tagged with GFP or astrin tagged with myc (Fig. 1C). Immunoprecipitation assays revealed that the C-terminus of HCR (aa 441–782, coiled-coil 3, CC3) and the C-terminus of astrin (aa 893–1193, coiled-coil 2, CC2) mediated the interaction between them (Fig. 1D–F). In order to confirm whether there is a direct interaction in vitro, we also constructed a plasmid expressing GST-tagged astrin and a series of plasmids expressing truncated forms of His-tagged HCR to perform a GST pull-down experiment. The result confirmed that the third region of HCR interacted with astrin in vitro (Fig. 1G), which was consistent with the co-IP results in vivo.

**Fig. 2:**
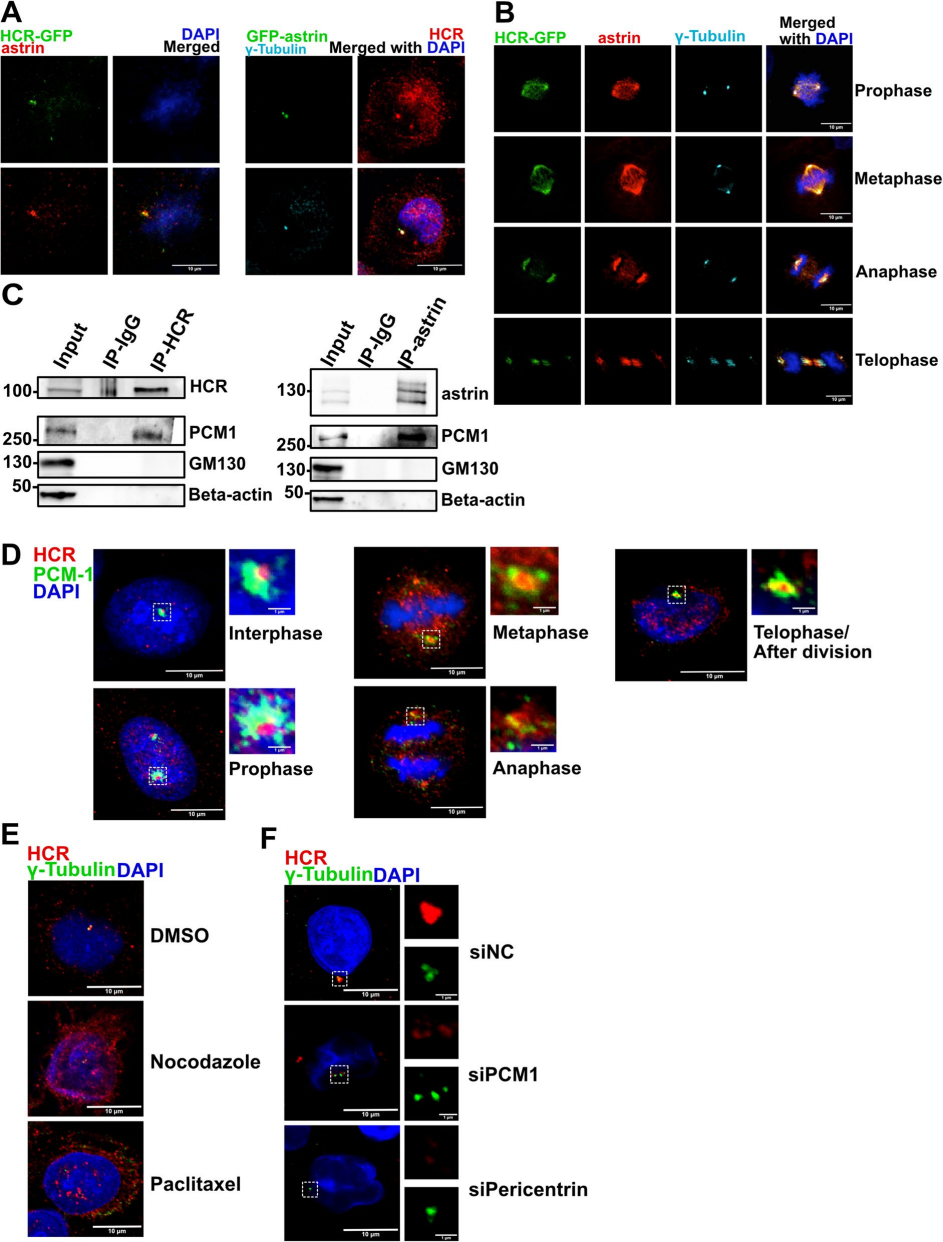
HCR co-localizes with astrin at the centrosome and mitotic spindle. **A** HeLa cells stably expressing HCR-GFP (green) were stained with an astrin antibody (red) and DAPI (blue) followed by confocal microscopy analysis (left panel); HeLa cells transfected with GFP-astrin (green) were stained with HCR (red) and gamma-tubulin (cyan) antibodies and DAPI (blue) for nuclear staining (right panel); scale bars, 10 μm. **B** Mitotic HeLa cells stably transfected with HCR-GFP (green) were stained with astrin (red), gamma-tubulin (cyan), and DAPI (blue); scale bars, 10 μm. **C** HeLa cell lysates were immunoprecipitated with control rabbit IgG or anti-HCR and detected by immunoblotting for HCR and PCM1. Beta-actin was used as a negative control (left panel) or immunoprecipitated with astrin and analyzed by western blotting for astrin and PCM1. Beta-actin was used as negative control (right panel). **D** HeLa cells were synchronized and stained with PCM1 (green), HCR (red), and DAPI (blue); scale bars, 10 μm; inset scale bars, 1 μm. **E** HeLa cells were treated with DMSO, 2 μg/ml nocodazole, or 1 μM paclitaxel and then stained with anti-HCR (red), anti-gamma-tubulin (green), and DAPI (blue); scale bars, 10 μm. **F** HeLa cells were transfected with the indicated siRNA and then immunostained for HCR (red) and gamma-tubulin (green). The nucleus was stained with DAPI (blue); scale bars, 10 μm; inset scale bars, 1 μm

Previous studies have reported that astrin is located in the centrosome and spindle [13, 17]. To more precisely examine the intracellular localization of HCR, we generated a stable HeLa cell line transfected with GFP-tagged HCR (Additional file 2: Fig. S2A). Immunofluorescence (IF) staining showed that stably transfected HCR co-localized with astrin. In addition, the IF image of GFP-tagged-astrin-transfected HeLa cells co-stained with HCR and gamma-tubulin showed that astrin and HCR were co-localized in the centrosome (Fig. 2A). While the CC2 domain of astrin was sufficient to be recruited by the kinetochore [38], we also found that it was colocalized with the CC3 domain of HCR around centriolar (Additional file 2: Fig. S2B). This further confirms that HCR and astrin bind to each other through their C-terminus. In mitotic cells, HCR showed spindle localization indicated by alpha-tubulin, similar to that of astrin (Additional file 2: Fig. S2C). To confirm that the spindle localization of HCR is real and reliable, we also knocked down HCR by RNA interference (RNAi), and the results showed that the spindle localization of HCR disappeared (Fig. S2D). Also, GFP-tagged HCR showed co-localization with astrin throughout mitosis (Fig. 2B). Since both HCR and astrin co-immunoprecipitated with PCM1 (Fig. 2C), HeLa cells were stained with HCR and PCM1. The results showed that HCR only overlapped on the edges of the PCM1 throughout the cell cycle, except for telophase, which suggests that HCR may function as a bridge between the PCM and centriole (Fig. 2D). To further investigate whether HCR is also recruited to the centrosome via the microtubule transport system as PCM1, we disrupted the balance of microtubules using either the microtubule inhibitor nocodazole or microtubule stabilizer paclitaxel. Both treatments caused centrosome disintegration and disrupted the localization of HCR (Fig. 2E, Additional file 2: Fig. S2E), suggesting that the localization of HCR requires balanced microtubule dynamics. We also investigated whether HCR localization is regulated by PCM1 and pericentrin. Depletion of either PCM1 or pericentrin resulted in the delocalization of HCR from the whole centrosome (Fig. 2F), which indicated that the centrosome localization of HCR was controlled by both PCM1 and pericentrin. In turn, the knockdown of HCR did not affect PCM1 localization (Additional file 2: Fig. S2F). These results indicate that HCR is indeed a centrosome-associated protein and is under the control of the PCM platform.

### Astrin deubiquitinates HCR and is essential for its centrosomal localization

**Fig. 3:**
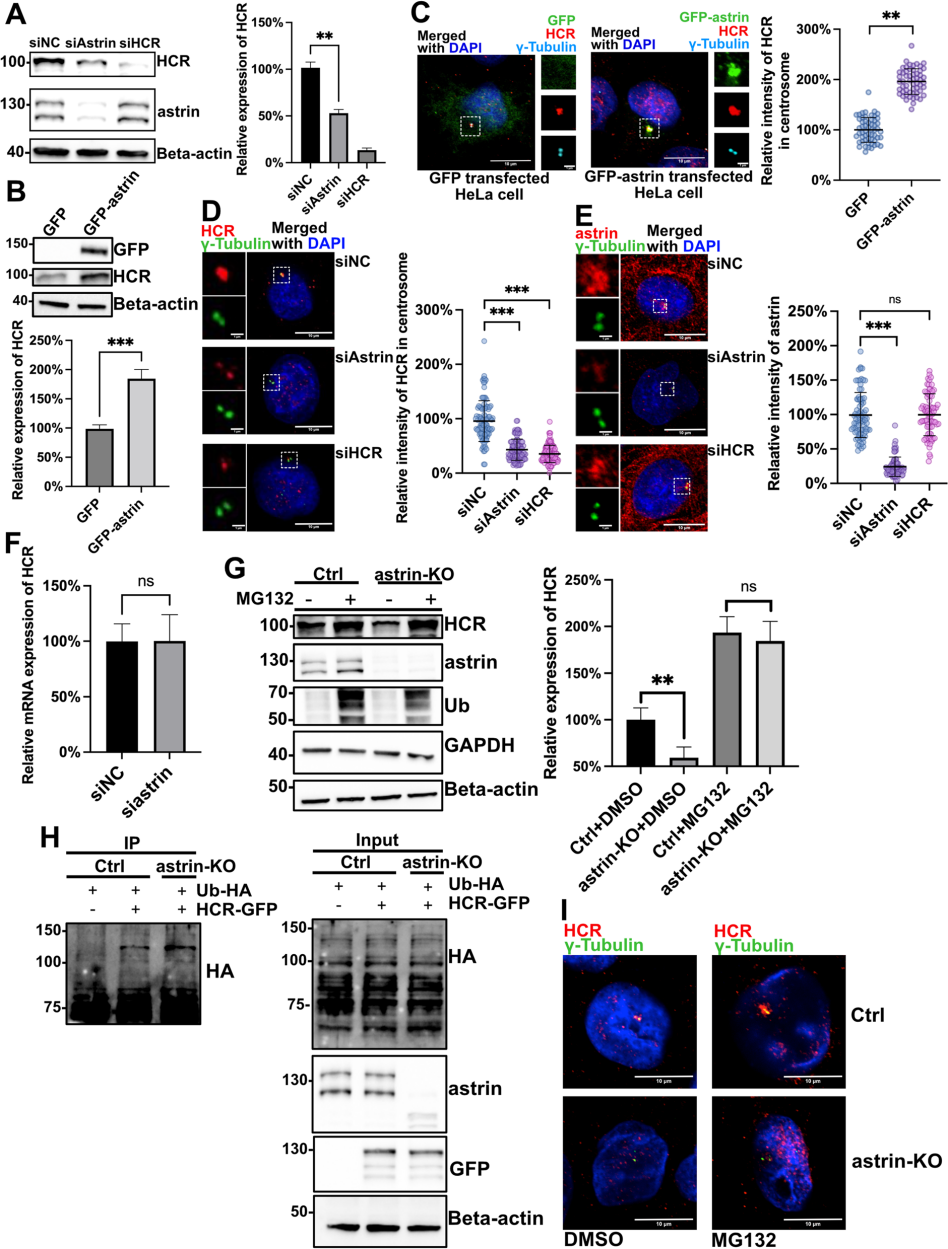
Astrin protects HCR from ubiquitination and ensures the centrosome localization of HCR. **A** Negative control, astrin, and HCR siRNA-treated HeLa cells were analyzed by western blotting with antibodies against HCR, astrin, and beta-actin. The relative abundance of HCR protein was normalized to beta-actin and statistically analyzed. Error bars represent the mean ± SD of three independently performed experiments (*n* = 3); **P < 0.01 and ***P < 0.001 (Student’s *t* test). The individual data values were provided in Additional file 6: Raw Data. **B** HeLa cells transfected with GFP alone or GFP-astrin were immunoblotted for GFP, HCR, and beta-actin. The relative protein levels of HCR were statistically analyzed across three independent experiments (*n* = 3). Error bars represent the mean ± SD; ***P < 0.001 (Student’s *t* test). The individual data values were provided in Additional file 6: Raw Data. **C** GFP alone or GFP-astrin-transfected HeLa cells were subjected to immunostaining with HCR (red), gamma-tubulin (cyan), and DAPI (blue); scale bars, 10 μm; inset scale bars, 1 μm. The relative intensity of HCR in the centrosome was normalized to gamma-tubulin and statistically analyzed. Error bars represent the mean ± SD; **P < 0.01 (Student’s *t* test). **D** Negative control, astrin, or HCR siRNA-treated HeLa cells were co-stained with HCR (red), gamma-tubulin (green), and DAPI (blue); scale bars, 10 μm; inset scale bars, 1 μm. The relative intensity of HCR in the centrosome was normalized to gamma-tubulin and statistically analyzed. One hundred cells (*n* = 100) per group were counted for each condition from three independent experiments. Error bars represent the mean ± SD; ***P < 0.001 (Student’s *t* test). **E** Negative control, astrin, and HCR siRNA-treated HeLa cells were co-stained with astrin (red), gamma-tubulin (green), and DAPI (blue); scale bars, 10 μm; inset scale bars, 1 μm. The relative intensity of HCR in the centrosome was normalized by gamma-tubulin and statistically analyzed. One hundred cells (*n* = 100) per group were counted for each condition from three independent experiments. Error bars represent the mean ± SD; ***P < 0.001; ns, no significance (Student’s *t* test). **F** Real-time PCR analysis of *HCR* mRNA levels in control and astrin siRNA-transfected HeLa cells. For statistical analysis, *HCR* mRNA levels were normalized to *GAPDH*; *n* = 4; ns, no significance (Student’s *t* test). The individual data values were provided in Additional file 6: Raw Data. **G** Parental and astrin-KO HeLa cells were treated with MG132 or DMSO for 16 h to suppress the ubiquitin-proteasome pathway. The lysates of each group were analyzed by immunoblotting with antibodies to HCR, astrin, ubiquitin, GAPDH, and beta-actin. For quantitative analysis, the level of HCR of each group was normalized to beta-actin. Error bars represent the mean ± SD of three independently performed experiments (*n* = 3); **P < 0.01; ns, no significance (Student’s *t* test). The individual data values were provided in Additional file 6: Raw Data. **H** mCherry empty vector or HCR-mCherry plasmid in conjunction with ubiquitin-HA was transfected into parental or astrin-KO HeLa cells for immunoprecipitation. The precipitates were analyzed by western blot with the indicated antibodies. **I** DMSO- or MG132-treated parental HeLa cells and astrin-KO HeLa cells were co-stained with HCR (red), gamma-tubulin (green), and DAPI (blue); scale bars, 10 μm

To further analyze the functional relationship between HCR and astrin, we used siRNA to knockdown astrin and HCR in HeLa cells. Interestingly, depletion of astrin simultaneously reduced the protein level of HCR, while the protein level of astrin did not change after knockdown of HCR (Fig. 3A), and the decrease of HCR caused by depletion of astrin was not due to apoptosis or changes in the cell cycle (Additional file 3: Fig. S3A). Correspondingly, transient transfection of GFP-astrin in HeLa cells also increased the expression of endogenous HCR (Fig. 3B). These results suggested that astrin positively regulated the protein level of HCR.

Additionally, IF staining showed that more HCR was recruited to the centrosome in cells overexpressing astrin as compared to astrin-depleted cells (Fig. 3C, D). By contrast, the depletion of HCR did not affect the centrosomal localization of astrin (Fig. 3E).

To address the mechanism by which astrin affects the expression of HCR, we first examined whether astrin regulates HCR at the mRNA level. Real-time quantitative PCR results showed that the knockdown of astrin did not change the mRNA expression of HCR, suggesting that the regulation does not occur at the transcriptional level (Fig. 3F). Since the ubiquitin-proteasome pathway is one of the most common protein degradation pathways in mammalian cells [39], we speculated that astrin may affect the ubiquitination of HCR and then reduce the degradation of HCR. To test this hypothesis, an astrin knockout (KO) HeLa cell line was generated using the CRISPR/Cas9 technology and was verified by western blot (Additional file 3: Fig. S3B). It was shown that the level of HCR protein decreased significantly after astrin knockout. However, there was almost no difference in the expression level of HCR between astrin-KO and parental HeLa cells when treated with the proteasome inhibitor MG132 (Fig. 3G). Furthermore, immunoprecipitation analysis showed that the loss of astrin caused an increase in ubiquitinated HCR (Fig. 3H). Taken together, these results indicate that astrin protects HCR from ubiquitin-proteasome-mediated degradation and therefore maintains the protein level of HCR. Next, we questioned whether astrin was also responsible for the localization of HCR. The IF image in Fig. 3I showed that the recruitment of HCR on the centrosome was enhanced in HeLa cells treated with MG132. However, in astrin-KO cells treated with MG132, the centrosome localization of HCR did not significantly increase (Fig. 3I). Collectively, these results suggest that astrin not only protects HCR from ubiquitinated degradation, but also is responsible for the centrosome localization of HCR.

### Both HCR and astrin contribute to the centrosome localization of CEP72

**Fig. 4:**
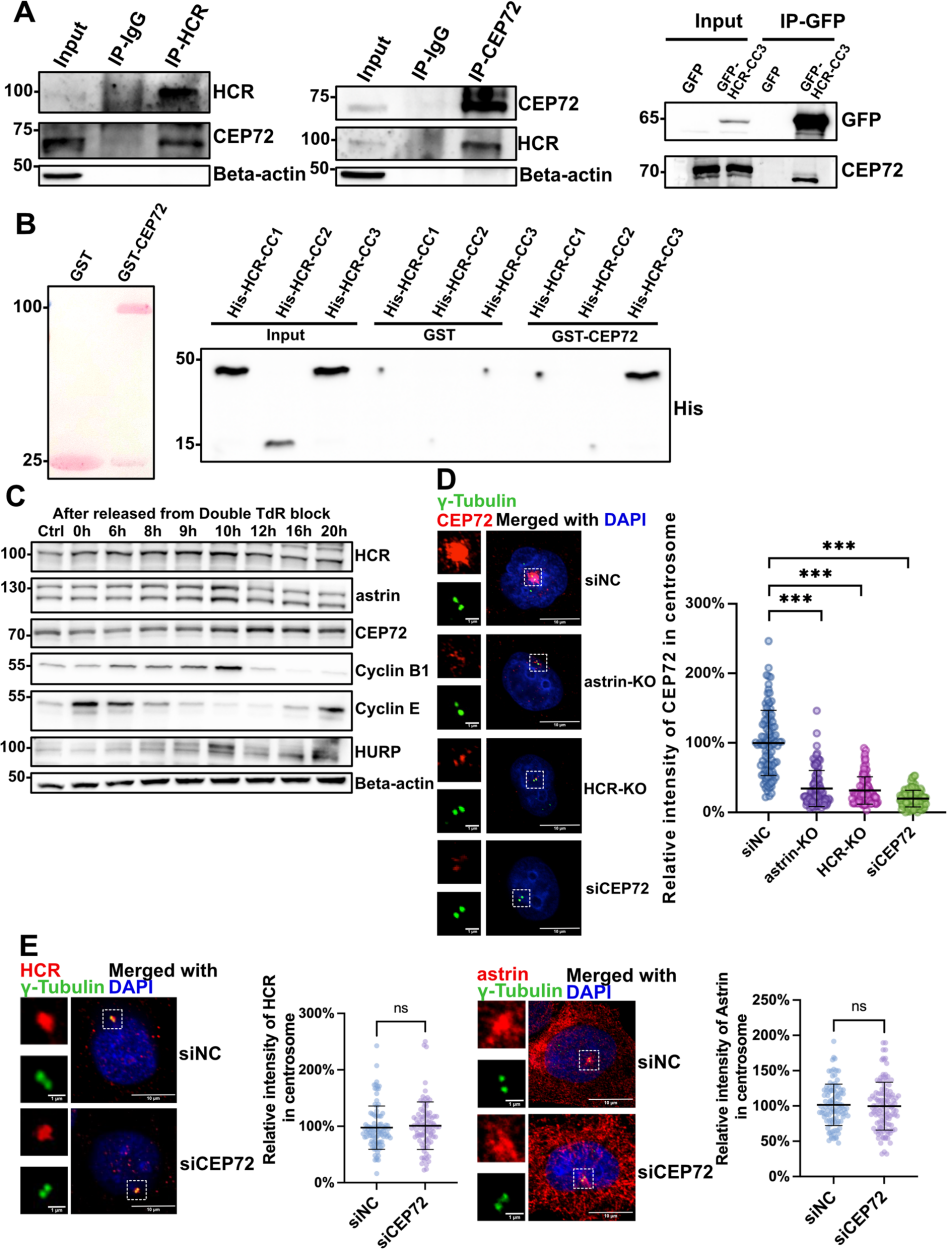
HCR directly binds to and ensures the centrosomal localization of CEP72. **A** Co-immunoprecipitation analysis of HCR binding to CEP72. HeLa cell lysates were immunoprecipitated with CEP72, HCR, or control rabbit IgG antibodies and analyzed by western blotting with anti-CEP72 and anti-HCR antibodies. Beta-actin was used as a negative control (left penal). GFP alone or GFP-HCR-CC3 plasmid was transfected into HeLa cells and immunoprecipitated using an GFP antibody. The precipitates were detected by immunoblotting with antibodies to GFP and CEP72 (right panel). **B** In vitro binding assay of HCR coiled-coil domains with CEP72. GST alone, GST-tagged CEP72, and His-tagged HCR fragments were purified from *E. coli* strain BL21(DE3), and a pull-down assay was performed to examine the CEP72-binding domain in HCR. **C** HeLa cells released from double-thymidine arrest were harvested at each time point and were analyzed by immunoblotting with antibodies against HCR, astrin, CEP72, cyclin B1, cyclin E, HURP, and beta-actin. **D** Negative control, CEP72 siRNA-treated HeLa cells, astrin-KO cells, and HCR-KO cells were co-stained with CEP72 (red), gamma-tubulin (green), and DAPI (blue); scale bars,10 μm; inset scale bars, 1 μm. For quantitative analysis, the intensity of CEP72 at the centrosome was normalized by gamma-tubulin. One hundred cells (*n* = 100) per group were counted from three independent experiments. Error bars represent the mean ± SD. ***P < 0.001 (Student’s *t* test). **E** Negative control or CEP72 siRNA-treated HeLa cells were co-stained with HCR (red) and gamma-tubulin (green) antibodies and DAPI (blue) for nuclear staining (upper panel) or co-stained with astrin (red) and gamma-tubulin (green) antibodies and DAPI (blue) for nuclear staining (lower panel). For quantitative analysis, the intensity of HCR (upper panel) or astrin (lower panel) at the centrosome was normalized to gamma-tubulin. One hundred cells (*n* = 100) per group were counted from three independent experiments. Each bar represents the mean ± SD (upper panel); ns, no significance (Student’s *t* test); scale bars, 10 μm; inset scale bars, 1 μm

Another candidate binding partner of HCR is CEP72, a centrosome protein localized to the PCM [17, 40]. Both astrin and CEP72 are essential for the centrosome localization of a series of MCPH proteins, such as CDK5RAP2 (CEP215), CEP152, and CEP63, which ensure the successful duplication of centrioles [17]. Since astrin directly binds to CEP72, we wondered whether the association between HCR and CEP72 is direct or mediated by astrin or other proteins. Co-IP and GST pull-down assays confirmed that HCR directly binds to CEP72 with the third coiled-coil domain (Fig. 4A, B). As a cell cycle-dependent protein, the expression level of astrin changes at different stages of the cell cycle [15]. To examine the expression pattern of HCR and CEP72 in the cell cycle, HeLa cells at each cycle stage were obtained by the double-thymidine block method and analyzed by western blotting. It was revealed that the protein level of HCR increased from S to G2/M phase, peaked in the M phase, and then significantly decreased in the G1 phase, which was almost consistent with that of astrin, whereas the peak expression of CEP72 was later than that of astrin and HCR (Fig. 4C), suggesting that CEP72 might be under the regulation of astrin and HCR. In order to better understand whether astrin and HCR regulate CEP72, an HCR-knockout (KO) HeLa cell line was generated using CRISPR/Cas9 technology and was verified by western blotting (Additional file 2: Fig. S2A). Knocking out either HCR or astrin significantly reduced the signal of CEP72 on the centrosomes (Fig. 4D), while the expression level of CEP72 was almost unaffected (Additional file 4: Fig. S4). On the other hand, the depletion of CEP72 by siRNA did not affect the signals of HCR and astrin on the centrosomes (Fig. 4E).

### HCR recruits MCPH proteins to centrioles and promotes centriole replication

**Fig. 5:**
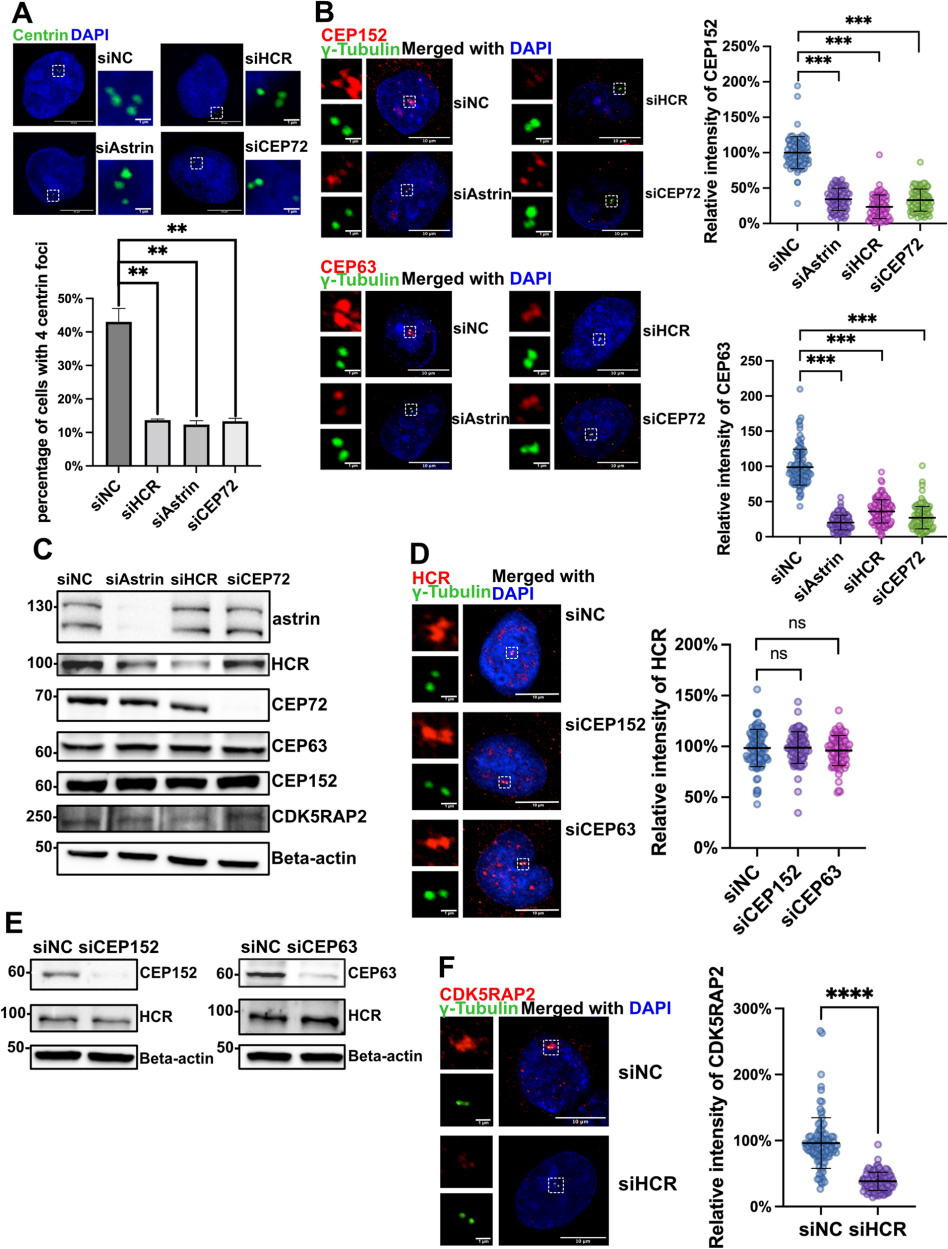
HCR promotes centriole duplication by recruiting MCPH proteins to the centrosome. **A** Negative control, astrin, HCR, and CEP72 siRNA-treated HeLa cells were co-stained with centrin-1 (green) and DAPI (blue). For quantitative analysis, the number of centrioles in each cell was counted for a total of 100 cells from three independent experiments. Error bars represent the mean ± SD; **P < 0.01 (Student’s *t* test); scale bars, 10 μm. **B** Negative control, astrin, HCR, and CEP72 siRNA-treated HeLa cells were co-stained with anti-CEP152 (red), anti-gamma-tubulin (green), and DAPI (blue) for nuclear staining (upper panel) or co-stained with anti-CEP63 (red), anti-gamma-tubulin (green), and DAPI (blue) for nuclear staining (lower panel). For quantitative analysis, the intensity of CEP152 or CEP63 at the centrosome was normalized to gamma-tubulin. Cells (*n* = 100 per group) were counted from three independent experiments. Error bars represent the mean ± SD; ***P < 0.001 (Student’s *t* test); scale bars,10 μm; inset scale bars, 1 μm. **C** Negative control, astrin, HCR, and CEP72 siRNA-treated HeLa cells were analyzed by immunoblotting with antibodies against astrin, HCR, CEP72, CEP152, CEP163, CDK5RAP2, and beta-actin. **D** Negative control, CEP152, and CEP63 siRNA-treated HeLa cells were co-stained with HCR (red), gamma-tubulin (green), and DAPI (blue). For quantitative analysis, the intensity of HCR at the centrosome was normalized by gamma-tubulin. Cells (*n* = 100 per group) were counted from three independent experiments. Error bars represent the mean ± SD; ns, no significance (Student’s *t* test); scale bars, 10 μm; inset scale bars, 1 μm. **E** Negative control, CEP152, and CEP63 siRNA-treated HeLa cells were analyzed by western blotting using antibodies against astrin, HCR, CEP72, and beta-actin. **F** Negative control and HCR siRNA-treated HeLa cells were co-stained with CDK5RAP2 (red), gamma-tubulin (green), and DAPI (blue) for immunofluorescence detection. For quantitative analysis, the intensity of CDK5RAP2 at the centrosome was normalized to gamma-tubulin. scale bars, 10 μm; inset scale bars, 1 μm. Cells (*n* = 100 per group) were counted from three independent experiments. Error bars represent the mean ± SD; ***P < 0.001 (Student’s *t* test)

Previous studies have revealed that centriole duplication relies on the centrosome localization of MCPH-associated proteins and PCM proteins. Among them, depletion of astrin or CEP72 reduced the recruitment of MCPH proteins, such as CEP152 and CEP63, to the centrosome, resulting in the inability of the centriole to duplicate properly from two to four foci [17, 41]. We found that knocking down HCR, astrin, or CEP72 by using siRNA lowered the 4 centriole foci ratio (Fig. 5A) and reduced the signals of CEP152 and CEP63 on the centrosomes (Fig. 5B) while not affecting their protein levels (Fig. 5C). In turn, depletion of CEP152 and CEP63 by siRNA did not affect the localization and expression levels of HCR, astrin, or CEP72 (Fig. 5D, E). Furthermore, immunoprecipitation analysis showed that HCR had no direct interactions with CEP152 and CEP63 (Additional file 4: Fig. S5). In addition to CEP152 and CEP63, another MCPH protein closely related to astrin-CEP72 recruitment is CDK5RAP2, which is also responsible for ensuring the replication of the centrosome [17]. Consistent with the results of astrin and CEP72 in the work of Kodani et al., the depletion of HCR by siRNA also caused the delocalization of CDK5RAP2 (Fig. 5F). These results suggested that, like astrin, HCR is also a key factor determining the centrosome localization of MCPH protein.

### Depletion of HCR impedes microtubule assembly due to the loss of centrosome localization of CEP72

**Fig. 6:**
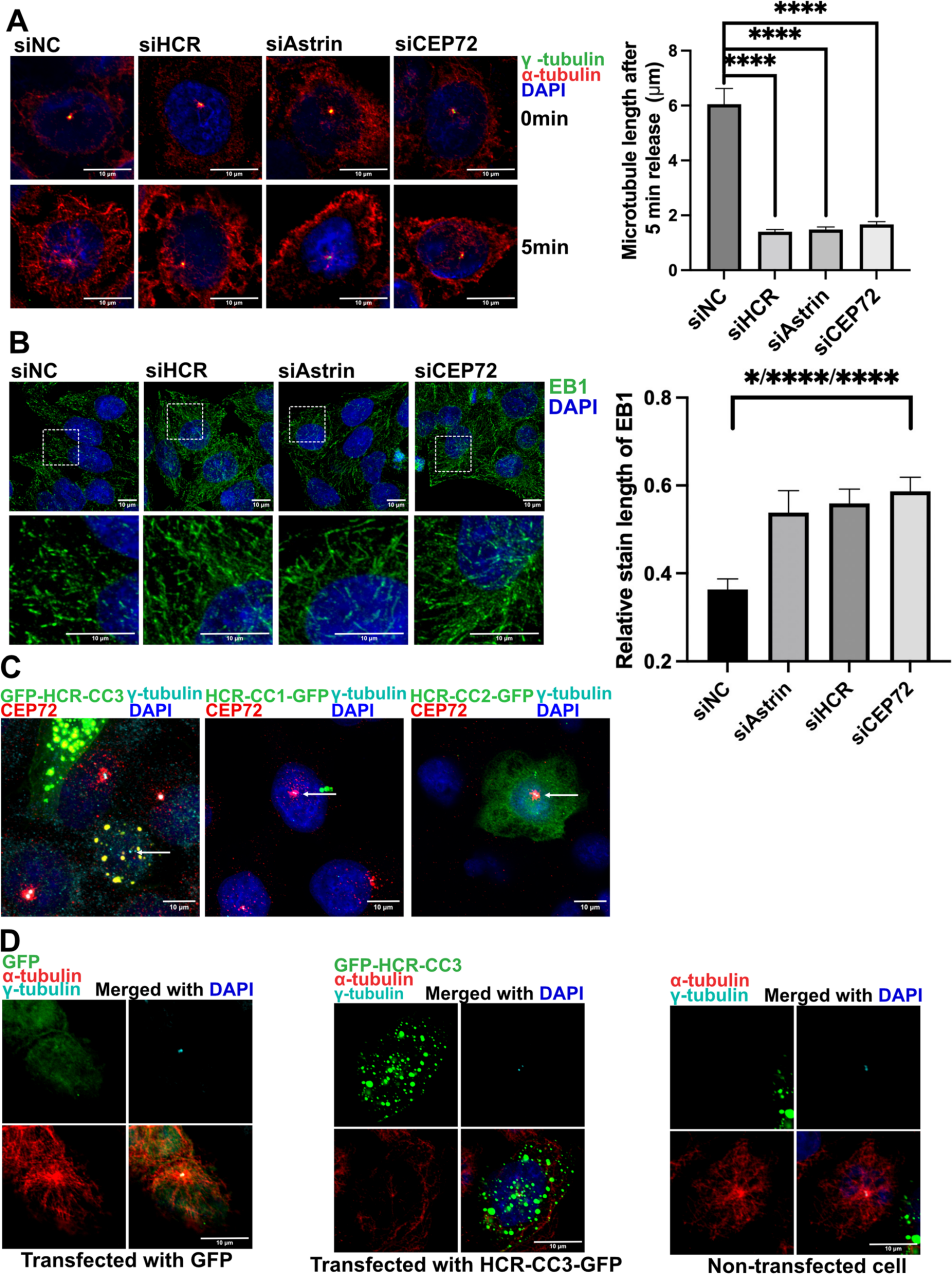
HCR regulates microtubule organization by recruiting CEP72 to the centrosome. **A** Microtubule regrowth assay of negative control, astrin, HCR, and CEP72 siRNA-treated HeLa cells co-stained with gamma-tubulin (green), alpha-tubulin (red), and DAPI (blue). The quantified analysis was based on alpha-tubulin staining length. Error bars represent the mean ± SD of cells (*n* = 100 per group) from three independently performed experiments; ****P < 0.0001 (Student’s *t* test); scale bars, 10 μm. **B** Negative control, astrin, HCR, and CEP72 siRNA-treated HeLa cells were co-stained with EB1 (green) and DAPI (blue) for immunofluorescence detection. Quantitative analysis was based on the staining length of EB1. Error bars represent the mean ± SD of cells (*n* = 100 each group) from three independently performed experiments. ****P < 0.0001 (Student’s *t* test); scale bars, 10 μm. **C** GFP-tagged HCR deletion fragments (green) were transfected into HeLa cells and co-stained with CEP72 (red) and DAPI (blue). Arrows represent the area of the centrosome; scale bars, 10 μm. **D** Microtubule regrowth assay of vector- or HCR-3-GFP-transfected HeLa cells co-stained with alpha-tubulin (red), gamma-tubulin (cyan), and DAPI (blue); scale bars indicate 10 μm

One of the most important roles of the centrosome is to regulate microtubule dynamics. PCM proteins play a critical role in the recruitment and assembly of microtubules. A previous study showed that depletion of CEP72 affected the nucleation activity of the microtubules and therefore decreased microtubule regrowth [40]. Similar results were obtained after the depletion of astrin and HCR by siRNA in HeLa cells (Fig. 6A) and RPE cells (Additional file 4: Fig. S6). It was reported that the destruction of the microtubule organization center could increase the length of microtubule plus-end tracking protein EB1 along the microtubules, which represents a decrease in the polymerization speed of the MT plus ends [42]. Compared with mock-treated cells, depletion of HCR, astrin, and CEP72 by siRNA caused a longer staining length of EB1, indicating that the polymerization of microtubules was slowed down (Fig. 6B). Together, these results revealed that lack of any of these three proteins could lead to microtubule nucleation defects and abnormal localization of EB1.

Since the interaction between HCR and CEP72 relied on the C-terminal coiled-coil of HCR (CC3), we transfected GFP-tagged HCR-CC3 into HeLa cells to observe the effect on microtubule organization. In IF images, overexpressed HCR-CC3 showed many large puncta all over the cytoplasm, and the endogenous CEP72 was captured into these puncta, thus losing centrosome localization (Fig. 6C). This phenomenon indicated that overexpressed HCR-CC3 functioned as a dominant-negative inhibitor of endogenous HCR activity. Moreover, the microtubule organization center was seriously disrupted in HCR-CC3-transfected cells, which was in strong contrast to the clear microtubule aster in the surrounding non-transfected cells (Fig. 6D). These results provided further evidence that HCR-dependent centrosome localization of CEP72 is essential for microtubule organization.

### Depletion of HCR results in mitotic defects, DNA damage, and decreased tumor proliferation

**Fig. 7:**
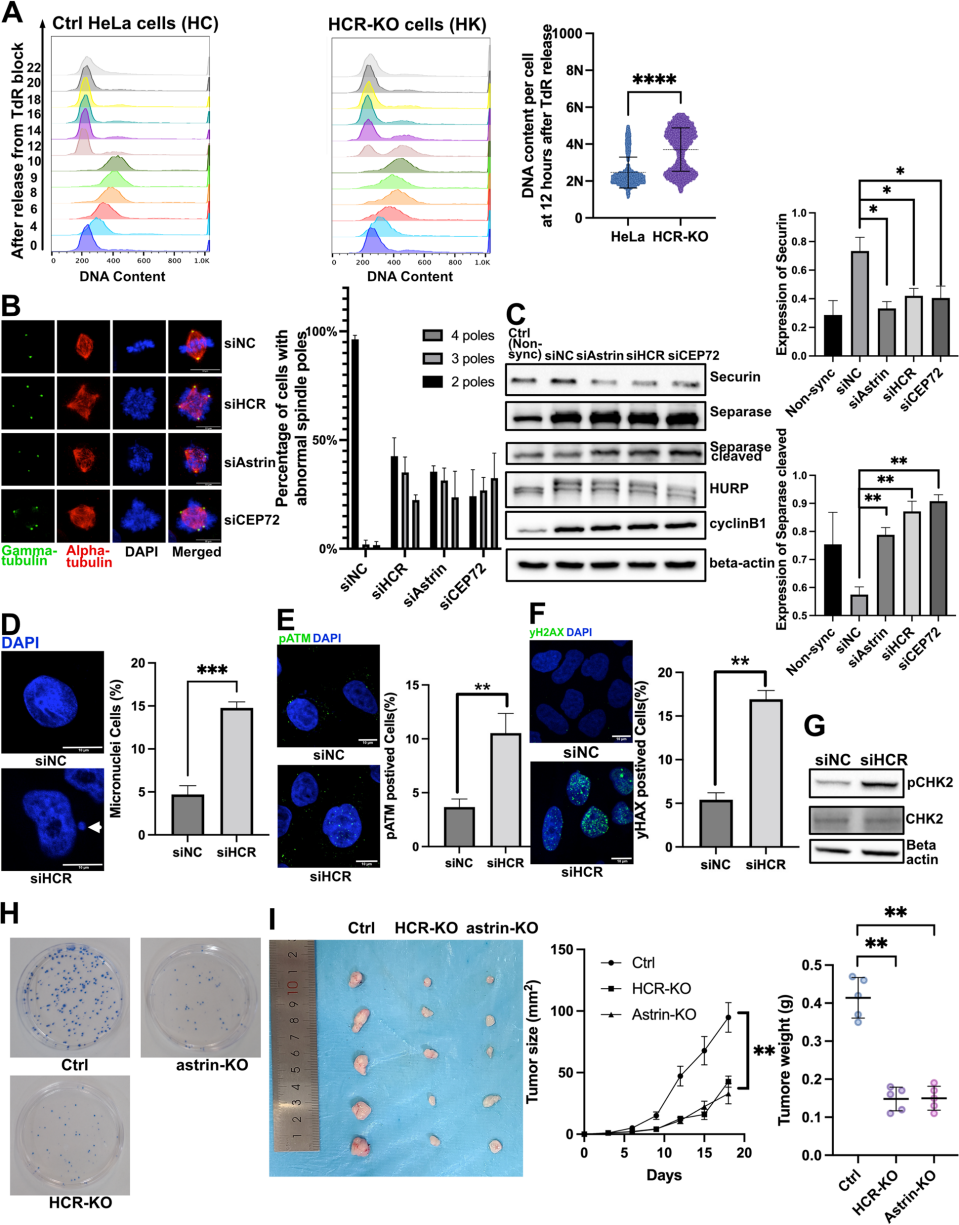
HCR depletion causes mitotic defects, DNA damage, and decreased tumor proliferation. **A** After releasing from double-thymidine arrest for the indicated time, parental HeLa cells and HCR-KO HeLa cells were fixed and stained with PI (DNA staining) for flow cytometry. The DNA content in cells is diploid (2N) in the G1 phase and becomes tetraploid (4N) from S to G2/M phase. When mitosis ends, the DNA content in the cell should revert from 4N to 2N. The cell cycle results were analyzed and plotted based on the DNA content in cells. A total of 10,000 cells were counted per group. **B** Negative control, astrin, HCR, and CEP72 siRNA-treated HeLa cells were treated with 100 ng/ml nocodazole for 16 h to be arrested in M phase and co-stained with gamma-tubulin (green), alpha-tubulin (red), and DAPI (blue). Cells (*n* = 100 each group) were counted from three independent experiments. Error bars represent the mean ± SD; *P < 0.05 (Student’s *t* test). **C** Negative control, astrin, HCR, and CEP72 siRNA-treated HeLa cells were treated with 100 ng/ml nocodazole for 16 h to be arrested in M phase and were analyzed by immunoblotting with antibodies to securin, separase, HURP, cyclin B1, and beta-actin, and the relative protein levels of securin and cleaved separase were analyzed statistically. Error bars represent the mean ± SD of three independently performed experiments (*n* = 3); *P < 0.05 and **P < 0.01 (Student’s *t* test). The individual data values were provided in Additional file 6: Raw Data. **D** Negative control and HCR siRNA-treated HeLa cells were stained with DAPI (blue). The quantified analysis was based on the percentage of the cells containing micronucleus. Cells (*n* = 100) were counted from three independent experiments. Each bar represents the mean ± SD; ***P < 0.001 (Student’s *t* test). Arrow represents the micronuclei. **E** Negative control and HCR siRNA-treated HeLa cells were co-stained with pATM (green) and DAPI (blue). The quantified analysis was based on the percentage of pATM-positive cells. Cells (*n* = 100 each group) were counted from three independent experiments. Error bar represents the mean ± SD; **P < 0.01 (Student’s *t* test). **F** Negative control and HCR siRNA-treated HeLa cells were co-stained with gamma-H2AX (green) and DAPI (blue). The quantified analysis was based on the percentage of gamma-H2AX-positive cells. Cells (*n* = 100 each group) were counted from three independent experiments. Error bar represents the mean ± SD; **P < 0.01 (Student’s *t* test). **G** Negative control and HCR siRNA-treated HeLa cells were analyzed by immunoblotting with antibodies to pCHK2, CHK2, and beta-actin. **H** Colony formation assays of parental HeLa cells, HCR-KO cells, and astrin-KO cells. **I** Parental HeLa cells, HCR-KO cells, and astrin-KO cells (1 × 10^6^) were transplanted in the athymic mice, and tumor sizes were measured every 3 days after the formation of a measurable tumor. Error bars represent the mean ± SD for different animal measurements (*n* = 5 each group); *P* < 0.01, one-way ANOVA for tumor weight analysis and two-way ANOVA for tumor size analysis. The individual data values were provided in Additional file 6: Raw Data

Apart from their roles in centrosome replication, depletion of astrin or CEP72 also led to mitotic spindle pole defects and mitotic arrest [15, 40]. Cell cycle analysis by flow cytometry showed that almost half of the HCR-KO cells remained in M phase, while almost all the parental HeLa cells returned from M phase to G1 phase (Fig. 7A). This indicated that the loss of HCR might also lead to mitotic spindle defects and mitosis progression arrest.

In mitotic cells, depletion of HCR by siRNA also caused multipolar spindle formation, suggesting that the absence of HCR could prevent the normal assembly of spindles (Fig. 7B, Additional file 4: Fig. S7). Similar results were obtained when astrin or CEP72 was knocked down, which is consistent with previous studies (Fig. 7B, Additional file 4: Fig. S7) [16, 40]. During the assembly of mitotic spindles, securin, a negative regulator of separase, can inhibit the production of activated separase before the onset of anaphase, which maintained the integrity of the mitotic centrosomes [43–46]. We showed here that securin was significantly downregulated, and separase was upregulated in HCR-depleted mitotic cells, similar to that in the astrin-depleted or CEP72-depleted cells (Fig. 7C) [15, 16]. This means that the absence of HCR, astrin, and CEP72 can cause abnormal activation of separase, which in turn leads to the polar division of the spindle to form a multi-polarization structure.

In addition, we found an increased ratio of micronuclei in HCR-depleted cells, which indicates frequent chromosome segregation errors (Fig. 7D). In line with this phenomenon, IF results showed that phosphorylation of the DNA damage checkpoint kinases ATM (Fig. 7E) and gamma-H2AX (Fig. 7F) was increased in HCR-depleted cells. Western blot analysis showed that phosphorylation of Chk2 was also increased in HCR-depleted cells (Fig. 7G). These results suggested that the depletion of HCR caused frequent mitotic errors, resulting in genomic instability and DNA damage response.

Astrin is also thought to be related to tumorigenesis [47–49]. To address whether HCR is involved in it, a colony formation assay was conducted. It showed that the knockout of astrin or HCR significantly impeded the colony formation ability of HeLa cells (Fig. 7H). To further verify that HCR knockdown could lead to a decrease in tumor proliferation, we constructed a subcutaneous transplantation tumor model in athymic mice. Tumor size in mice transplanted with either astrin-KO or HCR-KO cells was significantly smaller than that of mice transplanted with parental HeLa cells (Fig. 7I). These data indicated that loss of HCR is associated with a decrease in tumor proliferation, which may be due to a mitosis defect and genomic instability caused by HCR deletion.

## Discussion

HCR was initially reported as a centrosome and P-body-related protein [23, 32]. However, little is known about its cellular function and how it localizes to the centrosome. In this study, we provided evidence that HCR acted as an important link in the centrosomal protein recruitment chain. In fact, a variety of centrosomal components assemble at the centrosome in a PCM1-dependent manner, including centrin, ninein, astrin, and CEP131 [10]. PCM1 may deliver these proteins to the centrosome via the dynein-dynactin motor system [10, 50]. HCR is undoubtedly one of them because either depolymerization of the microtubule system or knockdown of PCM1 made HCR lose centrosome localization. Like astrin, CEP72, and CEP131, HCR did co-immunoprecipitate with PCM1. However, we would like to emphasize that astrin may play a more important role in maintaining centrosome localization of HCR. A previous study reported protein interaction between astrin and CEP72 [17]. Here, we show that astrin, HCR, and CEP72 interact with each other. Further analysis showed that astrin is in the most upstream position, which is essential for the centrosome localization of HCR and CEP72. HCR is in the middle, which does not affect astrin localization, but is required for CEP72 centrosome recruitment, while CEP72 is at the most downstream, which does not affect the positioning of HCR and astrin. However, we found that astrin was essential for stabilizing HCR and CEP72, whereas HCR and CEP72 had no significant effect on the protein level of astrin. It is worth noting that Kodani et al. reported that astrin and CEP72 stabilize each other, which differs from our results.

The potential of a centrosome to anchor microtubules requires the correct assembly of a subset of proteins. According to the recruitment chain described by Kodani et al., CDK5RAP2 is recruited to the centrosome by astrin and CEP72, followed by CEP152, WDR62, and CEP63 in a stepwise, hierarchical manner, and finally comes CDK2, a protein kinase critical for centriolar duplication [17]. The localization of HCR is in the middle of PCM1 and centrin1 (Additional file 5: Fig. S8), which means that it may act as part of the chain linking PCM and centriole. We did find that depletion of HCR phenocopied the effect of astrin or CEP72 depletion on the centrosomal localization of CDK5RAP2. Accordingly, the centrosomal localization of CEP152 and CEP63, two factors downstream of CEP72, were also regulated by HCR, but no direct interactions were detected (Additional file 4: Fig. S5).

In addition, we found that there was an interaction between HCR and CEP131 (also named AZI1) (Additional file 5: Fig. S9), which is consistent with the predictions of Ling et al. [23]. In the study of Kodani et al., CEP131, as a pericentriolar satellite protein, was responsible for ensuring the localization of CEP152 [17]. The interaction between HCR and CEP131 suggests that the recruitment of these MCPH proteins to the centrosome is more complicated than currently known. Like HCR, CEP131 is also considered to play an important role in maintaining genomic stability and tumor proliferation [51, 52].

One of the important roles of astrin in mitosis is to strengthen the connection between microtubules and the outer kinetochore of the chromosome, allowing the chromosome to withstand the tension from the spindle filament. In this process, astrin forms a complex with SKAP, MYCBP, and LC8 in kinetochore microtubules [36, 37, 53]. However, our results did not support the interaction between HCR and this complex (Additional file 5: Fig. S10). Although there is no evidence that HCR localizes to the kinetochore, it is still possible that HCR indirectly influences the role of astrin at the kinetochore, such as the transport of astrin between the spindle pole and the kinetochore, just like NuMA does [54]. Interestingly, we also found an interaction between HCR and NuMA (Additional file 5: Fig. S11). There may be an unknown relationship between NuMA and HCR on the spindle, which can affect or be affected by astrin to participate in the assembly and activity of mitotic spindles. Alternatively, HCR may be associated with important kinases, such as Plk-1 or PP1, which are responsible for the phosphorylation of astrin on kinetochore [36, 55, 56].

Another important role of astrin is to participate in the cohesion between sister chromatids in mitosis, which is the key point at which the existence of astrin can prevent early activation of separase before the onset of anaphase [15, 16]. In this study, we found that the knockdown of HCR increased the expression level of the active form of separase in M phase cells (Fig. 6E), suggesting that HCR is likely to affect sister chromatid cohesion. These critical mitosis processes are regulated by Aurora, a key family of kinases in charge of mitosis [57–60]. Since the Aurora kinases regulate the active conversion of astrin during mitosis, it is definitely worth exploring whether they also regulate HCR [53, 61, 62].

HCR is also localized to P-bodies and interacts with EDC4. Astrin was reported to recruit raptor to stress granules (SGs) upon oxidative stress, where it colocalized with G3BP1, an SG marker [63]. In fact, P-bodies and SGs are closely linked in function [64]. Interestingly, we also found that HCR co-localized with astrin and EDC4 in HeLa cells treated with arsenite (Additional file 5: Fig. S12), and the centrosomal protein CEP85 was also considered to be related to P-bodies [65]. Furthermore, we also found that EDC4 co-localized with the HCR in the centrosome and punctate staining around the spindle during mitosis (Additional file 5: Fig. S13). Additionally, a pair of P-bodies were found to reside at the centrosome in U2OS cells, as well as diverse non-malignant cells [66, 67]. Although the mechanism is unknown, the knockdown of some P-body components by RNA interference impaired primary cilium formation in human astrocytes [67]. Further in-depth study of HCR may reveal clearer functional links between the two structures.

In a more macroscopic direction, the elucidation of the intracellular mechanisms of HCR also contributes to the understanding of various diseases. Recent reports have proposed that HCR is closely related to alopecia areata, psoriasis, and diabetes [31, 33, 68]. HCR-deficient mice showed stress-induced alopecia [35]. Since primary cilia play an important role in the development of hair follicles, the role of HCR in ciliogenesis deserves future attention. In addition, the interaction between HCR and astrin also suggested that HCR might be related to cancers. Numerous reports have confirmed that astrin overexpression is often associated with malignancy, so HCR, as a protein regulated by astrin, may also be upregulated in tumor tissues [20, 48, 49, 53, 69, 70]. Moreover, analysis of the TCGA database (portal.gdc.cancer.gov) also revealed that the transcription of HCR did significantly increase in a variety of tumors (Additional file 5: Fig. S14). Although we did not investigate whether HCR was involved in tumorigenesis, as a regulator of the cell cycle and mitosis, this possibility exists. Interestingly, HCR may even have a potential link with COVID-19 [71]. In fact, linking microtubule and centrosome to virus infection is not a new idea. Previous studies have found that retroviruses, such as human immunodeficiency virus type 1 (HIV-1) infection, can affect changes in centrosome function [72]. Although there is no clear evidence as to whether HCR is a direct target of COVID-19, further in-depth research may help explain the biological mysteries of the centrosome and provide substantial clinical value.

## Conclusion

**Fig. 8:**
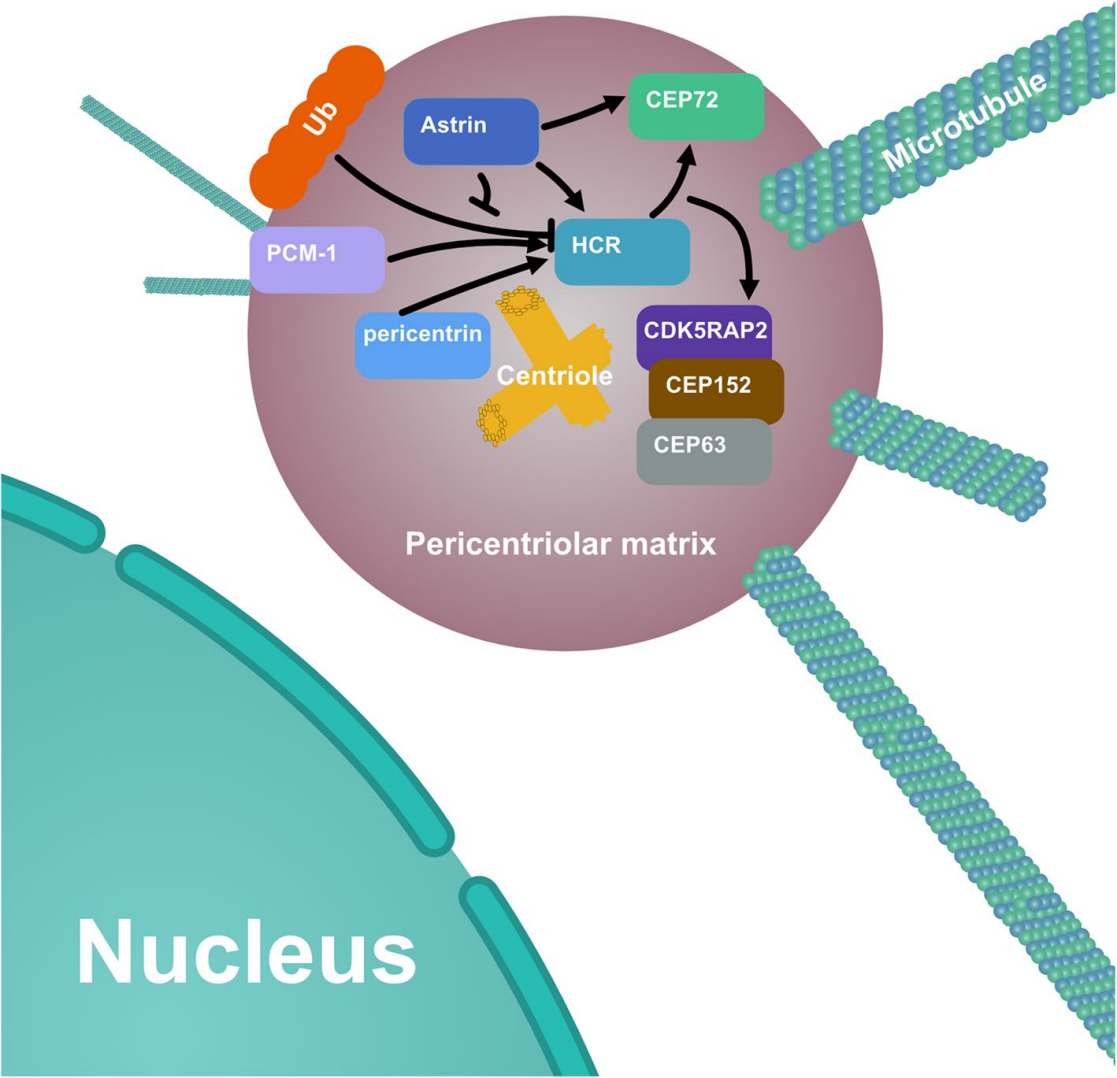
Localization and regulatory relationship of HCR and its related proteins on centrosomes. HCR delivery to centrosome requires PCM1, pericentrin, and astrin. HCR is protected by astrin from ubiquitination and recruits CEP72 and MCPH proteins to the centrosome

In conclusion, our results reveal the role of previously unfocused P-body protein HCR on centrosome, whereby HCR interacts with astrin to recruit CEP72 and MCPH proteins to the centrosome and ensures efficient centriole replication and other centrosome-related functions such as spindle-pole formation and microtubules organization (Fig. 8). Therefore, HCR not only acts as P-body component, but also plays an important role in the development of centrosome and the stability of the genome.

## Methods

### cDNA, plasmids, antibodies, and reagents

Human CCHCR1 cDNA (NM_019052) was amplified from HeLa cDNA by PCR amplification and subcloned into pEGFPN1 or pmCherryN2 vectors. Human astrin cDNA (NM_006461) in the pEGFPC2 vector was gifted by Dr. Yi-Ren Hong (Kaohsiung Medical University, Taiwan China) [73] and was subcloned into the pCMV-myc vector. CEP72 cDNA (NM_018140) was amplified from the pEBTet-CEP72-SNAP plasmid purchased from Addgene (plasmid #136819) and subcloned into the pEGFPN1 vector. Serial deletion fragments of indicated regions of HCR and astrin were amplified from HCR and astrin cDNA, respectively, and subcloned into pEGFPN1 and pCMV-Myc vectors, respectively.

Antibodies used in this study included: astrin (14726-1-AP, for western blotting (WB) 1:2000, for immunofluorescence (IF) 1:500); CEP72 (19928-1-AP, for WB 1:1000, for IF 1:400); CEP152 (21815-1-AP, for WB 1:1000, for IF 1:400); CEP63 (16268-1-AP, for WB 1:1000, for IF 1:400); CEP131 (25735-1-AP, for WB 1:1000); centrin-1 (12794-1-AP, for IF 1:400) from Proteintech (Wuhan, China); CCHCR1 (sc-135052, for IF: 1:100, WB 1:500); γ-tubulin (sc-17788, for IF 1:200); cyclin B1 (sc-245, for WB 1:500); cyclin E (sc-247, for WB 1:500); cyclin D1 (sc-246, for WB 1:500); securin (sc-56207, for WB 1:500); separase (sc-390314, for WB 1:500); EB1 (sc-47704, for IF 1:100); PCM1 (sc-398365, for IF 1:200, for WB 1:500); pericentrin (sc-376111, for WB 1:500. IF 1:200); c-myc (sc-40, WB 1:1000) from Santa Cruz Biotechnology (Dallas, Texas, USA); γ-tubulin (GTX113286, for IF 1:500); astrin (GTX115449, for IF 1:400, WB 1:1000) from Genetex (Irvine, CA, USA); CDK5RAP2 (A15476, for IF 1:200); GFP (AE012, WB1:1000); and mCherry (AE002, WB 1:1000) from Abclonal (Wuhan, China). Nocodazole (GC14075) and thymidine (GC15815) were purchased from GPLBIO (Montclair, CA, USA). Paclitaxel (S1748) and MG132 (SC0213) were purchased from Beyotime Biotechnology (Shanghai, China).

### Cell culture

The cell lines of HeLa (human cervical carcinoma cell), U2OS (human osteosarcoma cells), hTERT-RPE1 (immortalized human retinal pigment epithelial cells), and HEK293 (human embryonic kidney cells) were obtained from China Center for Type Culture Collection (Wuhan, China) and were cultured in DMEM high-glucose medium (Hyclone, Waltham, MA, USA) with 10% fetal bovine serum (FBS, Gibco, Waltham, MA, USA), 100 units/ml penicillin, and 10 μg/ml streptomycin (Biosharp, Hefei, China) in a humidified chamber with 5% CO_2_ at 37 °C. HCR-KO HeLa cells and astrin-KO HeLa cells were customized by VigeneBio (Jinan, Shandong, China) and Ubigene (Guangzhou, Guangdong, China) and cultured under the same conditions as HeLa cells.

### Construction of KO cells

The HCR-KO cell line was created by using CRISPR-Cas9 in HeLa cells with sgRNAs as follows:

- sgRNA1: CCCGAATGGTGTGGACCTTG
- sgRNA2: GCGGGAAGAACGGAACCGCC
- sgRNA3: AACGGGATGTTTCCAGTGAC
- sgRNA4: TGAGGTTGTCCGGAAGAACT

These sgRNAs are designed to target exon 3-13 of the human CCHCR1 gene.

The astrin-KO cell line was created by using CRISPR-Cas9 in HeLa cells with sgRNAs as follows:

- SPAG5-gRNA1: CTCTACTCCTAAAACGTCTG AGG
- SPAG5-gRNA2: ACCAGATCGTCTGTTCTCAA AGG

These sgRNAs are designed to target exon 3 of the human SPAG5 gene. The specific verification reports refer to Additional file 7 and Additional file 8.

### Cell cycle synchronization

HeLa cells and HCR-KO HeLa cells were first synchronized with 5 mM thymidine for 16 h, washed with phosphate-buffered saline (PBS) three times, and cultured in DMEM without thymidine for 12 h, After treatment with 5 mM thymidine for another 12 h, cells were released from thymidine and harvested at each time point according to experimental needs. For collecting mitotic cells, cells were released for about 10 h from a double-thymidine block to initiate prometaphase [54]. For separase and securin analysis in mitotic cells, cells were treated with siRNA for 72 h and incubated with nocodazole (100 ng/ml in medium) for another 16 h [15, 16].

### Plasmid transfection

HeLa cells were transfected with 15 μg of DNA plasmid in a 10-cm dish or 2 μg in each well of a 6-well plate using Lipo6000 Transfection Reagent (Beyotime Biotechnology, Shanghai, China) following the manufacturer’s instructions. Cells were harvested and then lysed for co-IP or fixed for IF after treatment for 24 h.

### siRNA interference

HeLa cells were transfected with 10 nM siRNA using Lipo6000 Transfection Reagent (Beyotime Biotechnology, Shanghai China) following the manufacturer’s instructions. The cells were harvested and then lysed or fixed for further analysis after treatment for 72 h. The CCHCR1 ON-TARGETplus SMARTpool siRNA was purchased from Dharmacon (Lafayette, CO, USA). The siRNAs targeting astrin (5′-CAAUACCAAGACCAACUGG-3′), CEP72 (5′-TTGCAGATCGCTGGACTTC-3′), CEP152 (5′-GCAUUGAGGUUGAGACUAA-3′), CEP63 (5′-GAGUUACAUCAGCGAGAUA-3′), Percentrin (5′-GCAGCUGAGCUGAAGGAGA-3′), and PCM1 (5′-UCAGCUUCGUGAUUCUCAG-3′) were synthesized by Ribobio (Guangzhou, Guangdong China).

### Co-immunoprecipitation

For the immunoprecipitation, plated cells were washed three times with PBS and then lysed with RIPA buffer (50 mM Tris, 150 mM NaCl, 0.1% NP40, with cocktail protease inhibitors (MCE Monmouth Junction, NJ, USA, Cat. No.: HY-K0011)) for 30 min on ice. Samples were then centrifuged at 14,000 rpm for 30 min to obtain lysate, and 5% of the lysates were saved as input. Then, 500 μg of the lysates was incubated with the 2 μg of antibodies for 2 h at 4 °C on a rotator, and then 50 μl of a mixed suspension of 50% protein A and protein G beads (pre-washed with PBS 3 times) was then added. Mixtures were incubated at 4 °C for 16 h on a rotator. The beads were collected by centrifuging at 2000 rpm for 2 min at 4 °C and then washed with PBS 3 times. The samples were eluted by resuspending washed beads in 30–50 μl of 2× SDS-loading buffer and heating at 95 °C for 5 min, followed by separation via SDS-PAGE and immunoblotting with appropriate antibodies.

### Immunofluorescence imaging

For immunofluorescence imaging, cells plated on glass coverslips were fixed with cold methanol, blocked with 10% FBS, and probed with primary antibodies and then secondary antibodies coupled with AlexFluor 488/555/594/647. DNA was stained with DAPI. Immunofluorescence pictures were imaged under an Olympus Confocal Laser Scanning Microscope FV3000 (Olympus Co., Tokyo, Japan) and processed by ImageJ (https://imagej.nih.gov/ij/download.html) when necessary.

### Microtubule regrowth assay

siRNA-treated or plasmid-transfected cells were treated with 1 μM nocodazole on ice for 30 min to depolymerize the microtubules and were then released from cold nocodazole after 0 min and 5 min to repolymerize the microtubules. For the microtubule regrowth assay, the cells were fixed and co-stained with gamma-tubulin and alpha-tubulin to show the microtubule organization center and microtubules, and the length of microtubules of each cell was measured to compare the differences between the groups.

### Cell flow cytometry

For cell cycle analysis, cells were trypsinized and fixed in 70% ethanol at 4 °C for 16 h, washed with PBS 3 times, and stained with 50 mg/ml propidium iodide (PI, DNA stain) and 0.025 mg/ml RNase A in PBS for 30 min at 37 °C. Cells were analyzed with FACS Calibur (Becton Dickinson, Franklin Lakes, NJ, USA). The cell cycle results were analyzed based on the DNA content in cells. For statistical analysis, the results of 10,000 cells in each group were counted and plotted.

### Real-time qPCR

Total RNA was isolated with TRIzol (Invitrogen, Waltham, MA, USA) and used for cDNA reverse transcription with the Goldenstar RT6 cDNA synthesis kit (Tsingke, Beijing, China). Quantitative PCR analysis of gene transcripts was performed by the qPCR method using qPCR Master Mix (Promega, Madison, WI, USA) and Jena qTOWER3 system with the expression of GAPDH as the endogenous control.

### Colony formation assay

Parental HeLa cells, HCR-KO HeLa cells, and astrin-KO HeLa cells were maintained in culture media in a 10-cm dish for 2 weeks, followed by staining with Giemsa stain. Then the number of stained colonies were counted.

### Tumor xenografts

Animals were randomly grouped in three groups with 5 mice per group. Parental HeLa cells, HCR-KO HeLa cells, or astrin-KO HeLa cells were injected into the subcutaneous prothorax of 6-week-old athymic mice with 1 × 10^6^ cells per mice (BALB/c, Guangzhou Medical Animal Center, Guangzhou, China). After visible tumors were observed, tumor size was measured every 3 days and calculated according to the following formula: length × width. The measurement and data processing were performed with blinding. All mice received a humane diet and living environment during the experiment. At the end of the experiment, all mice were executed in a humane manner, and the subcutaneous tumor was exfoliated and weighed. This study was approved by the Animal Care Committee of Shenzhen University Science Health Center.

### Domains analysis

For the construction of HCR fragments plasmids, the SMART Sequence Analysis Tools (https://smart.embl-heidelberg.de) was used to analysis the protein domains.

### Statistical analysis

For western blot results and immunofluorescence images, ImageJ (https://imagej.nih.gov/ij/download.html) was used to measure the intensity of the protein of interest. Microsoft Office Excel and GraphPad Prism were used to perform statistical analyses and graphing. For statistical analysis of blotting experiments, each experiment was performed three times independently. For statistical analysis of immunofluorescence images, 100 cells were counted from three independent experiments. All statistical results are presented as mean ± SD and tested with a two-tailed Student’s *t* test (GraphPad Prism software) to calculate the *P*-values between unpaired samples. The differences were considered statistically significant when *P* < 0.05.

## Supplementary Information

**Additional file 1: Fig. S1.** Repeated verification of the interaction between HCR and astrin. (A) Astrin Co-IP HCR and CEP72 and HCR Co-IP astrin and CEP72. HeLa cell lysates were immunoprecipitated with astrin, HCR, or control rabbit IgG antibodies and analyzed by western blotting with anti-astrin, anti-HCR antibodies. Anti-GM130 and anti-beta actin antibodies were used as negative control. The blotting of astrin, HCR and GM130 antibodies were incubated on the same membrane by repeatedly washing the membrane with antibody removal solution to increase comparability. (B) HCR interacts with astrin in HEK293 and U2OS cells. mCherry vector alone or HCR-mCherry in conjunction with the GFP-astrin plasmid were transfected into HEK293 cells for immunoprecipitation using an mCherry antibody. The eluted proteins were analyzed with mCherry and GFP antibodies. HCR-mCherry plasmid-transfected HEK293 cells were immunoprecipitated with astrin antibody or negative control rabbit IgG. The precipitates were analyzed with mCherry and astrin antibodies. U2OS cell lysates were immunoprecipitated with an HCR antibody or negative control rabbit IgG. The precipitates were analyzed with antibodies against HCR and astrin.

**Additional file 2: Fig. S2.** Identification of HCR-GFP stable Cell Line and Localization of HCR in cells. (A) Identification of HCR-KO HeLa cell line and stably expressing HCR-GFP cell line. Parental HeLa cells, HCR-KO HeLa cells, HCR-KO cells transfected with HCR-GFP, and stably transfected HCR-GFP HCR-KO cells were immunoblotted with an HCR antibody. (B) Co-localization of astrin-CC2 and HCR-CC3. HeLa cells transfected with astrin-CC2-myc and HCR-GFP or astrin-CC2-myc and HCR-CC3-GFP were co-stained with myc (red) and gamma-tubulin (cyan); scale bars, 10 μm. (C) Co-localization of HCR with alpha-tubulin. Mitotic HeLa cells stained with an alpha-tubulin antibody (green), HCR antibody (red), and DAPI (blue) for nuclear staining (left panel) or stained with anti-alpha-tubulin (green), anti-astrin (red), and DAPI (blue) (right panel); scale bars, 10 μm. (D) Identification of antibody staining to HCR. Negative control, HCR siRNA-treated HeLa cells were co-stained with HCR (red) and alpha-tubulin (green); scale bars, 10 μm. (E) The effect of Nocodazole on HCR is dose-dependent and recoverable. HeLa cells were treated with 1μM, 0.75μM, 0.5μM Nocodazole for 5 hours or treated with 1μM Nocodazole for 5hours then released from Nocodazole for 30 min, 1 hour, 2 hours, then co-stained with HCR (red) and gamma-tubulin (green). (F) Knockdown of HCR does not affect PCM1 localization. Negative control, HCR siRNA-treated HeLa cells were co-stained with HCR (red) and PCM1 (green); scale bars, 10 μm.

**Additional file 3: Fig. S3.** Apoptosis or cycle changes in astrin-KO cells and identification of astrin-KO cell line. (A) Parental, astrin-KO and HCR-KO HeLa cells were analyzed with astrin, HCR, cyclin B1 and Cleaved PARP antibodies. HeLa cells treated with DMSO, Paclitaxel, Bafilomycin and MG132 were analyzed with Cleaved PARP and HCR antibodies. (B) Parental and astrin-KO HeLa cells were immunoblotted with an astrin antibody.

**Additional file 4: Fig. S4.** Loss of either HCR or astrin slightly affects the protein level of CEP72. Parental HeLa cells, HCR-KO HeLa cells, and astrin-KO HeLa cells were analyzed by immunoblotting with CEP72 and beta-actin antibodies. **Fig. S5.** HCR does not bind to CEP63 and CEP152. HeLa cell lysates were immunoprecipitated with antibodies specific for HCR and negative control IgG. The precipitates were analyzed by immunoblotting with antibodies against CEP63 and CEP152. **Fig. S6.** Knockdown of astrin and HCR also caused microtubules organization defects in RPE cells. Microtubule regrowth assay of negative control, astrin and HCR siRNA-treated RPE cells co-stained with gamma-tubulin (green), alpha-tubulin (red), and DAPI (blue); scale bars, 10 μm. **Fig. S7.** Knockdown of astrin and HCR also caused mitotic spindle defects in RPE cells. Negative control, astrin and HCR siRNA-treated HeLa cells were treated with 100 ng/ml nocodazole for 16 hours to be arrested in M-phase and co-stained with gamma-tubulin (green), alpha-tubulin (red), and DAPI (blue); scale bars, 10 μm.

**Additional file 5: Fig. S8.** Co-localization of HCR with centrin1 and PCM1. HeLa cells transfected with HCR-GFP were co-stain with centrin1 (red) and gamma-tubulin (cyan) and DAPI (blue) or PCM1 (red) and gamma-tubulin (cyan) and DAPI (blue); scale bars, 10 μm. **Fig. S9.** HCR interacts with CEP131. HeLa cell lysates were immunoprecipitated with antibodies specific for HCR, astrin, or negative control rabbit IgG. The precipitates were analyzed by immunoblotting with antibodies against HCR and CEP131. **Fig. S10.** HCR did not interact with SKAP, MYCBP and LC8. HeLa cell lysates were immunoprecipitated with astrin, HCR, or control rabbit IgG antibodies and analyzed by western blotting with astrin, HCR, SKAP, MYCBP and LC8 antibodies. Beta actin antibody was used as negative control; HeLa cell lysates were immunoprecipitated with SKAP, MYCBP and LC8, or control rabbit IgG antibodies and analyzed by western blotting with astrin, HCR, SKAP, MYCBP and LC8 antibodies. Beta actin antibody was used as negative control. **Fig. S11.** HCR interacts with NuMA. mCherry vector alone or HCR-mCherry was transfected into HeLa cells for immunoprecipitation with mCherry antibody. The precipitates were analyzed by immunoblotting with HCR and NuMA antibodies. **Fig. S12.** HCR co-localizes with astrin and EDC4.HCR-GFP-transfected HeLa cells were treated with arsenite for 30 min and then co-stained with astrin (red) and EDC4 (cyan) for immunofluorescence detection; scale bars, 10 μm. **Fig. S13.** HCR co-localizes with EDC4 in mitosis. Negative control, HCR siRNA-treated HeLa cells were co-stained with HCR (red) and EDC4 (green); scale bars, 10 μm. **Fig. S14.** HCR was closely related to tumorigenesis. The dataset was from TCGA (portal.gdc.cancer.gov) and analyzed by TIMER 2.0 (cistrome.org).

**Additional file 6.** Individual data values. Raw data of Fig. 3A,B,F,G,7C and 7I.

**Additional file 7.** Raw data for Table 1.

**Additional file 8.** Verification reports of HCR-KO cell line.

**Additional file 9.** Verification reports of astrin-KO cell line.

**Additional file 10.** All Original and uncropped blots images used in manuscript.

## Data Availability

All data generated or analyzed during this study are included in this published article and its supplementary information files.

## Abbreviations

CCHCR1: Coiled-coil alpha-helical rod protein 1
SPAG5: Sperm-associated antigen 5
PCM: Pericentriolar materials
CEP: Centrosomal proteins
CDK5RAP2: Cyclin-dependent kinase 5 regulatory subunit-associated protein 2
MCPH: Microcephaly
SKAP: Small kinetochore-associated protein
LC8: Dynein light chain 8
MYCBP: Myc-binding protein
P-body: Processing body
EDC4: mRNA-decapping protein 4
WDR62: WD40-repeat protein 62
WB: Western blot
IF: Immunofluorescence
PCR: Polymerase chain reaction
GFP: Green fluorescent protein
GST: Glutathione S-transferase
