# Supplementary material for: CCHCR1-astrin interaction promotes centriole duplication through recruitment of CEP72
Source: BMC Biol. 2022 Oct 24;20:240. doi: 10.1186/s12915-022-01437-6 (PMC9590400; doi:10.1186/s12915-022-01437-6)
Supplement: Supplementary file 1 — Additional file 1: Fig. S1. Repeated verification of the interaction between HCR and astrin. (A) Astrin Co-IP HCR and CEP72 and HCR Co-IP astrin and CEP72. HeLa cell lysates were immunoprecipitated with astrin, HCR, or control rabbit IgG antibodies and analyzed by western blotting with anti-astrin, anti-HCR antibodies. Anti-GM130 and anti-beta actin antibodies were used as negative control. The blotting of astrin, HCR and GM130 antibodies were incubated on the same membrane by repeatedly washing the membrane with antibody removal solution to increase comparability. (B) HCR interacts with astrin in HEK293 and U2OS cells. mCherry vector alone or HCR-mCherry in conjunction with the GFP-astrin plasmid were transfected into HEK293 cells for immunoprecipitation using an mCherry antibody. The eluted proteins were analyzed with mCherry and GFP antibodies. HCR-mCherry plasmid-transfected HEK293 cells were immunoprecipitated with astrin antibody or negative control rabbit IgG. The precipitates were analyzed with mCherry and astrin antibodies. U2OS cell lysates were immunoprecipitated with an HCR antibody or negative control rabbit IgG. The precipitates were analyzed with antibodies against HCR and astrin. [file 12915_2022_1437_MOESM1_ESM.pdf]

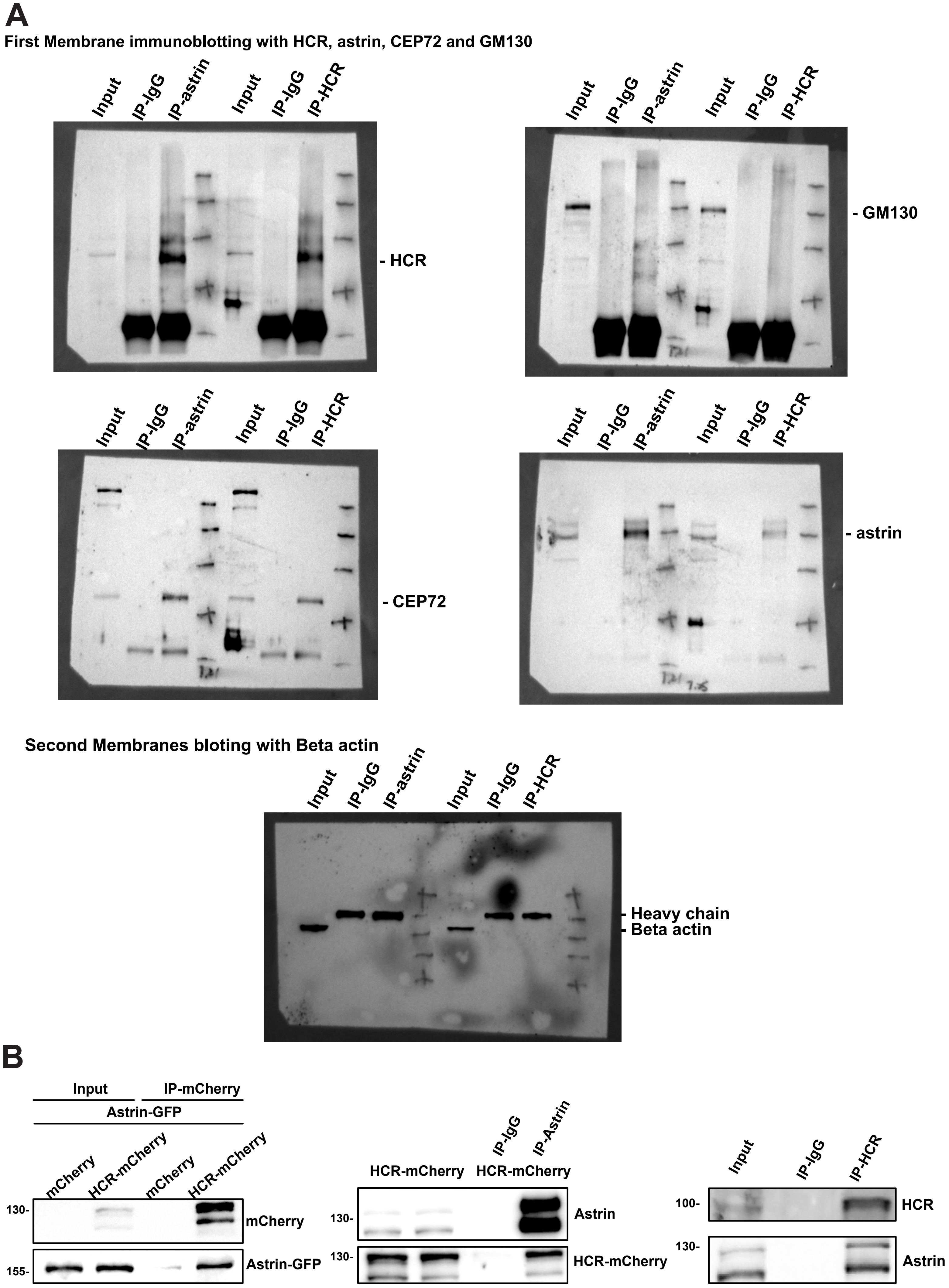
A
First Membrane immunoblotting with HCR, astrin, CEP72 and GM130
Input
IP-IgG
IP-astrin
Input
IP-IgG
IP-HCR
- HCR
- GM130
- CEP72
- astrin
Second Membranes bloting with Beta actin
- Heavy chain
- Beta actin
B
Input
IP-mCherry
Astrin-GFP
mCherry
HCR-mCherry
mCherry
HCR-mCherry
130-
mCherry
155-
Astrin-GFP
IP-IgG
IP-Astrin
HCR-mCherry
HCR-mCherry
130-
Astrin
130-
HCR-mCherry
Input
IP-IgG
IP-HCR
100-
HCR
130-
Astrin
