# Supplementary material for: CCHCR1-astrin interaction promotes centriole duplication through recruitment of CEP72
Source: BMC Biol. 2022 Oct 24;20:240. doi: 10.1186/s12915-022-01437-6 (PMC9590400; doi:10.1186/s12915-022-01437-6)
Supplement: Supplementary file 2 — Additional file 2: Fig. S2. Identification of HCR-GFP stable Cell Line and Localization of HCR in cells. (A) Identification of HCR-KO HeLa cell line and stably expressing HCR-GFP cell line. Parental HeLa cells, HCR-KO HeLa cells, HCR-KO cells transfected with HCR-GFP, and stably transfected HCR-GFP HCR-KO cells were immunoblotted with an HCR antibody. (B) Co-localization of astrin-CC2 and HCR-CC3. HeLa cells transfected with astrin-CC2-myc and HCR-GFP or astrin-CC2-myc and HCR-CC3-GFP were co-stained with myc (red) and gamma-tubulin (cyan); scale bars, 10 μm. (C) Co-localization of HCR with alpha-tubulin. Mitotic HeLa cells stained with an alpha-tubulin antibody (green), HCR antibody (red), and DAPI (blue) for nuclear staining (left panel) or stained with anti-alpha-tubulin (green), anti-astrin (red), and DAPI (blue) (right panel); scale bars, 10 μm. (D) Identification of antibody staining to HCR. Negative control, HCR siRNA-treated HeLa cells were co-stained with HCR (red) and alpha-tubulin (green); scale bars, 10 μm. (E) The effect of Nocodazole on HCR is dose-dependent and recoverable. HeLa cells were treated with 1μM, 0.75μM, 0.5μM Nocodazole for 5 hours or treated with 1μM Nocodazole for 5hours then released from Nocodazole for 30 min, 1 hour, 2 hours, then co-stained with HCR (red) and gamma-tubulin (green). (F) Knockdown of HCR does not affect PCM1 localization. Negative control, HCR siRNA-treated HeLa cells were co-stained with HCR (red) and PCM1 (green); scale bars, 10 μm. [file 12915_2022_1437_MOESM2_ESM.pdf]

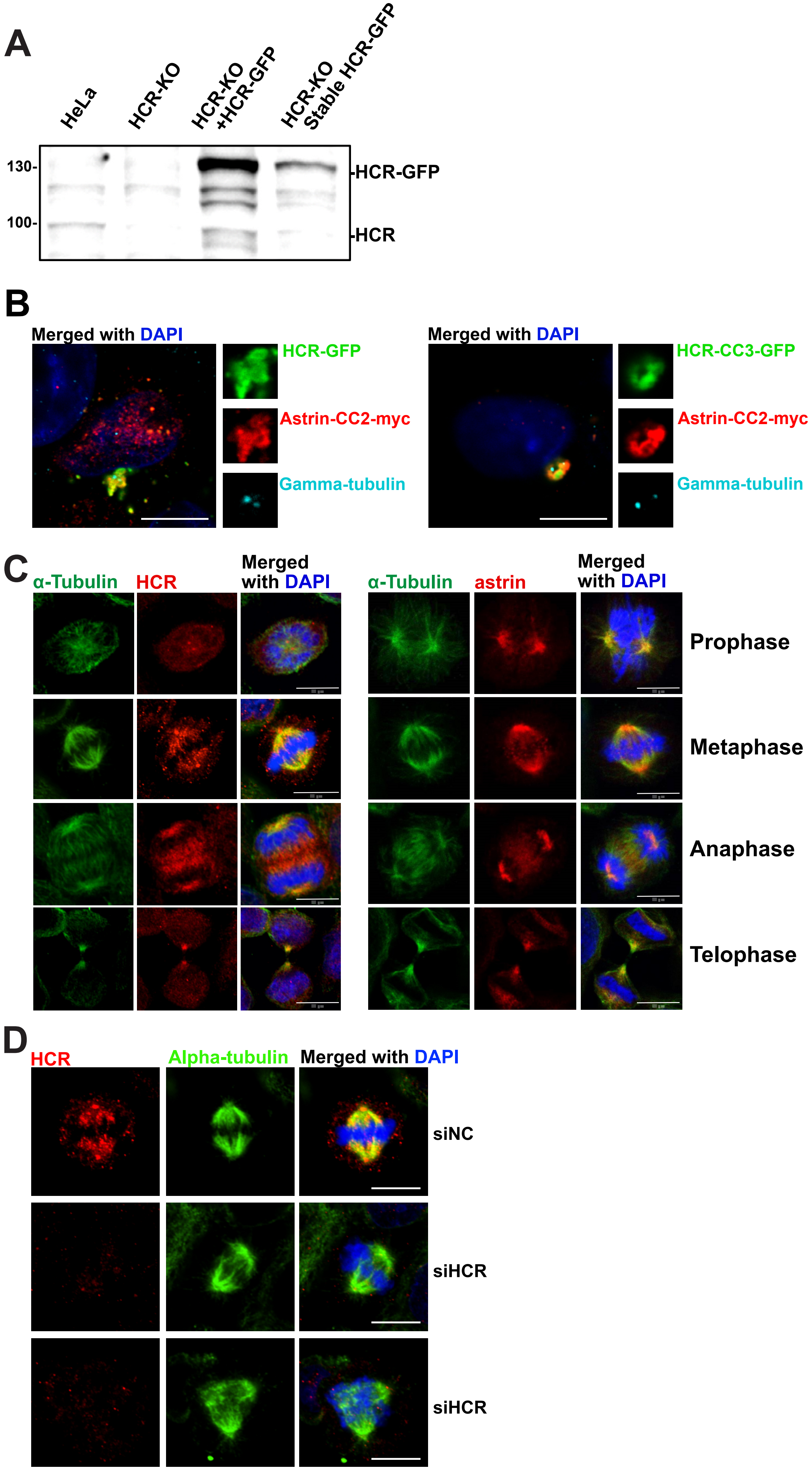
A
HeLa
HCR-KO
HCR-KO +HCR-GFP
HCR-KO Stable HCR-GFP
130-
100-
HCR-GFP
HCR
B
Merged with DAPI
HCR-GFP
Astrin-CC2-myc
Gamma-tubulin
Merged with DAPI
HCR-CC3-GFP
Astrin-CC2-myc
Gamma-tubulin
C
α-Tubulin
HCR
Merged with DAPI
α-Tubulin
astrin
Merged with DAPI
Prophase
Metaphase
Anaphase
Telophase
D
HCR
Alpha-tubulin
Merged with DAPI
siNC
siHCR
siHCR

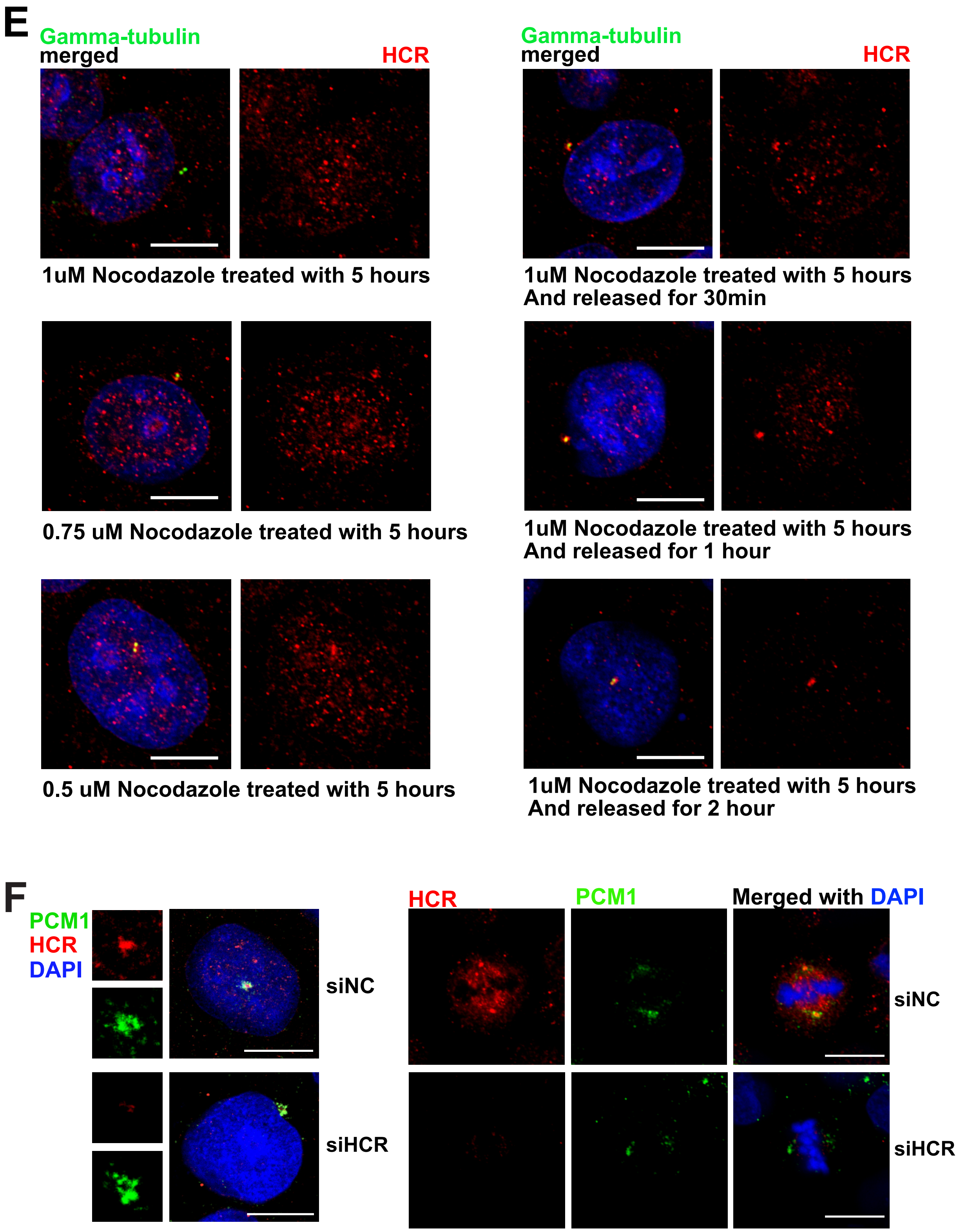

E
Gamma-tubulin
merged
HCR
1uM Nocodazole treated with 5 hours
1uM Nocodazole treated with 5 hours
And released for 30min
0.75 uM Nocodazole treated with 5 hours
1uM Nocodazole treated with 5 hours
And released for 1 hour
0.5 uM Nocodazole treated with 5 hours
1uM Nocodazole treated with 5 hours
And released for 2 hour
F
PCM1
HCR
DAPI
siNC
siHCR
HCR
PCM1
Merged with DAPI
siNC
siHCR
