# Supplementary figures and images for: CCHCR1-astrin interaction promotes centriole duplication through recruitment of CEP72 (part 1 of 2)
Source: BMC Biol. 2022 Oct 24;20:240. doi: 10.1186/s12915-022-01437-6 (PMC9590400; doi:10.1186/s12915-022-01437-6)

**A**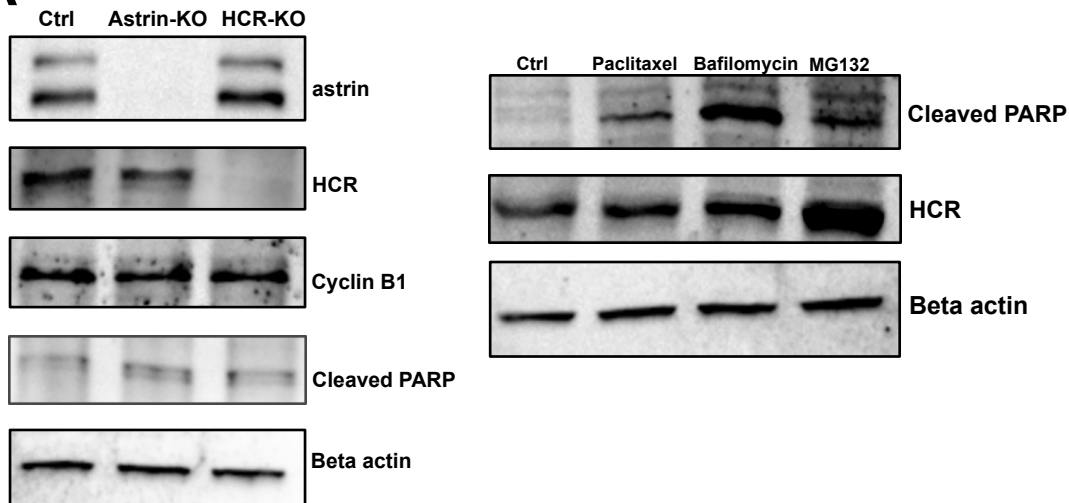**B**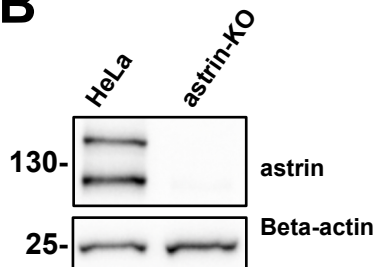

Supplement: Supplementary file 3 — Additional file 3: Fig. S3. Apoptosis or cycle changes in astrin-KO cells and identification of astrin-KO cell line. (A) Parental, astrin-KO and HCR-KO HeLa cells were analyzed with astrin, HCR, cyclin B1 and Cleaved PARP antibodies. HeLa cells treated with DMSO, Paclitaxel, Bafilomycin and MG132 were analyzed with Cleaved PARP and HCR antibodies. (B) Parental and astrin-KO HeLa cells were immunoblotted with an astrin antibody. [file 12915_2022_1437_MOESM3_ESM.pdf]

**Fig. S4**

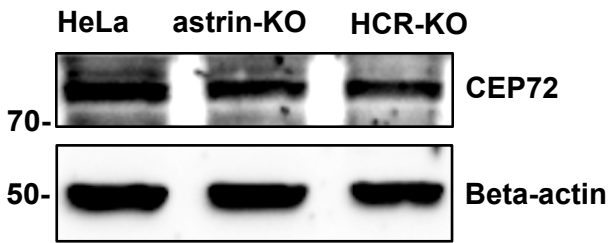

**Fig. S5**

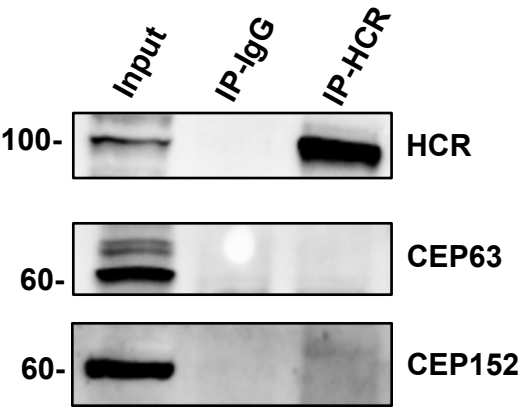

**Fig. S6**

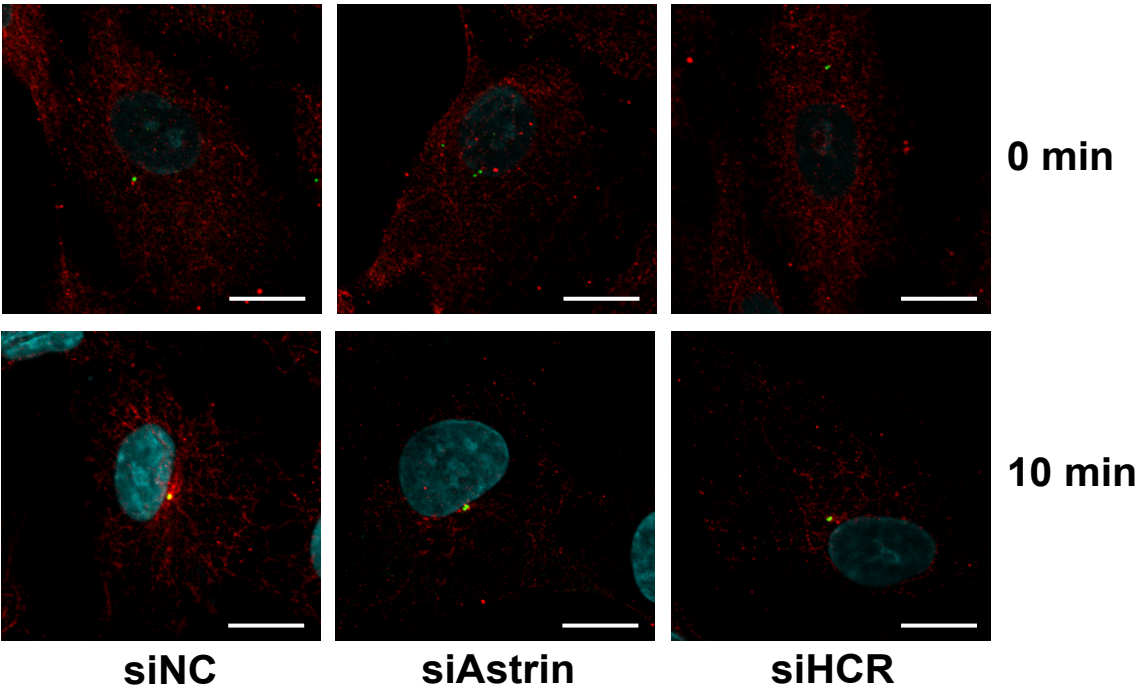

**Fig. S7**

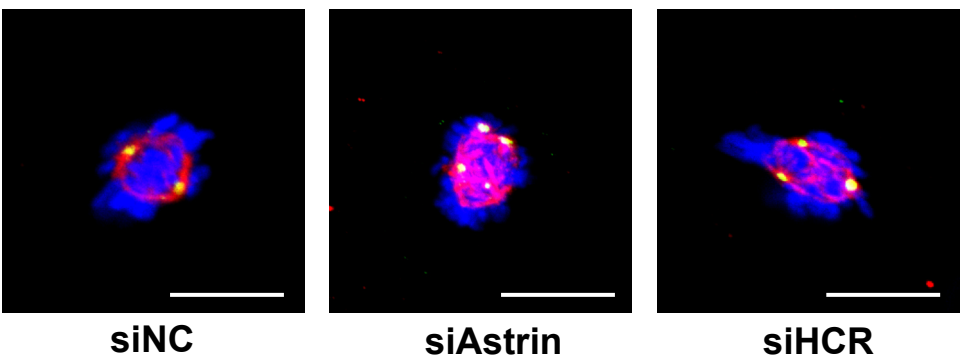

Supplement: Supplementary file 4 — Additional file 4: Fig. S4. Loss of either HCR or astrin slightly affects the protein level of CEP72. Parental HeLa cells, HCR-KO HeLa cells, and astrin-KO HeLa cells were analyzed by immunoblotting with CEP72 and beta-actin antibodies. Fig. S5. HCR does not bind to CEP63 and CEP152. HeLa cell lysates were immunoprecipitated with antibodies specific for HCR and negative control IgG. The precipitates were analyzed by immunoblotting with antibodies against CEP63 and CEP152. Fig. S6. Knockdown of astrin and HCR also caused microtubules organization defects in RPE cells. Microtubule regrowth assay of negative control, astrin and HCR siRNA-treated RPE cells co-stained with gamma-tubulin (green), alpha-tubulin (red), and DAPI (blue); scale bars, 10 μm. Fig. S7. Knockdown of astrin and HCR also caused mitotic spindle defects in RPE cells. Negative control, astrin and HCR siRNA-treated HeLa cells were treated with 100 ng/ml nocodazole for 16 hours to be arrested in M-phase and co-stained with gamma-tubulin (green), alpha-tubulin (red), and DAPI (blue); scale bars, 10 μm. [file 12915_2022_1437_MOESM4_ESM.pdf]

**Fig. S8**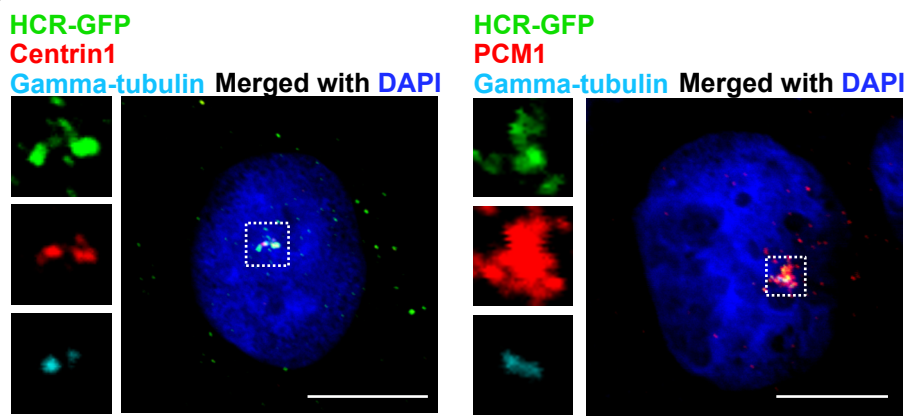**Fig. S9**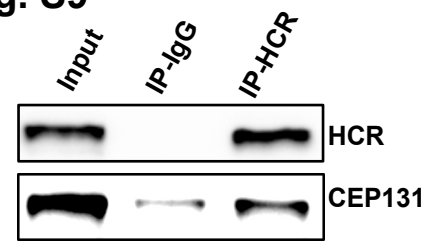**Fig. S10**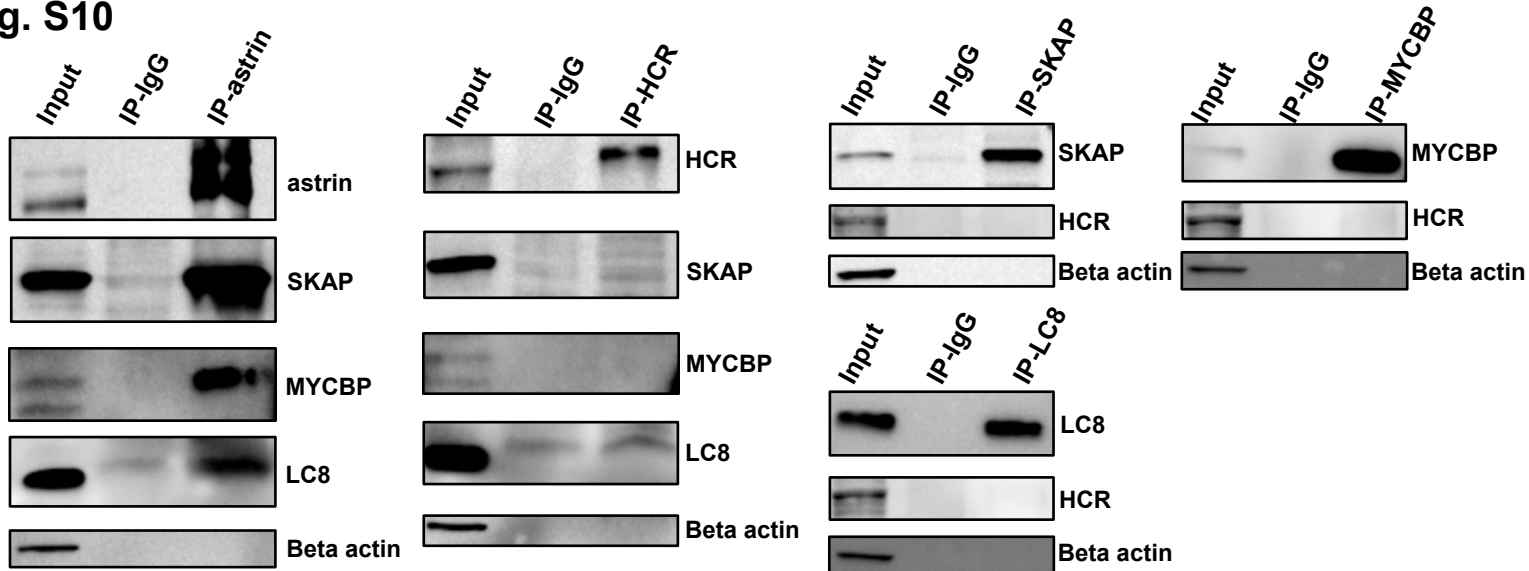**Fig. S11**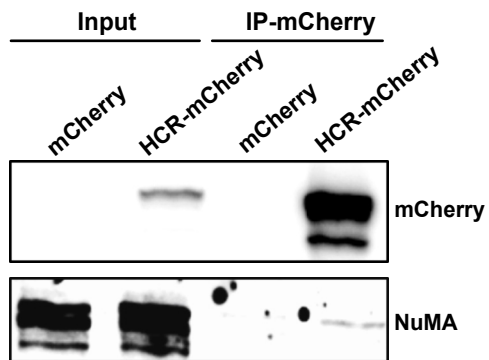**Fig. S12**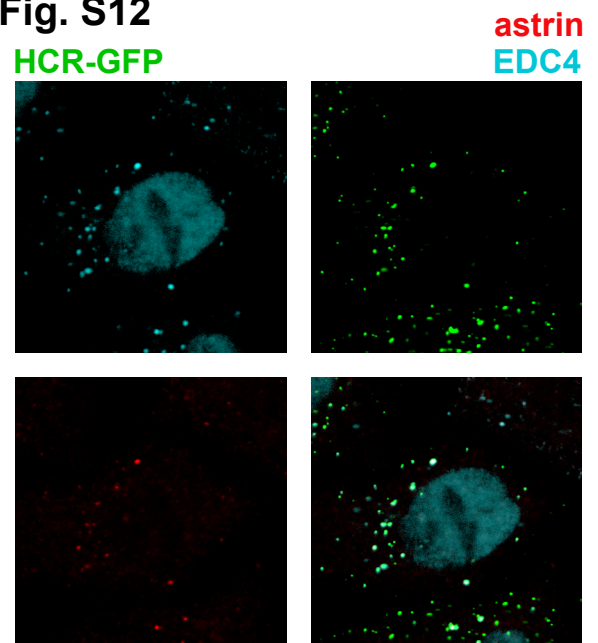**Fig. S13**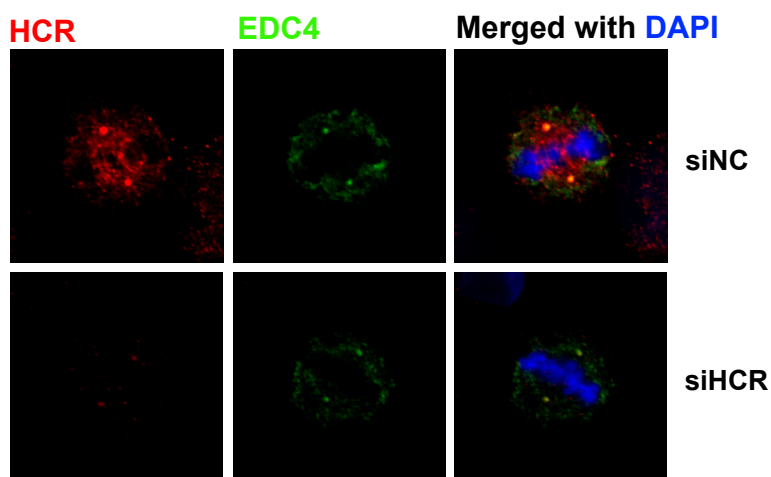

Fig S14

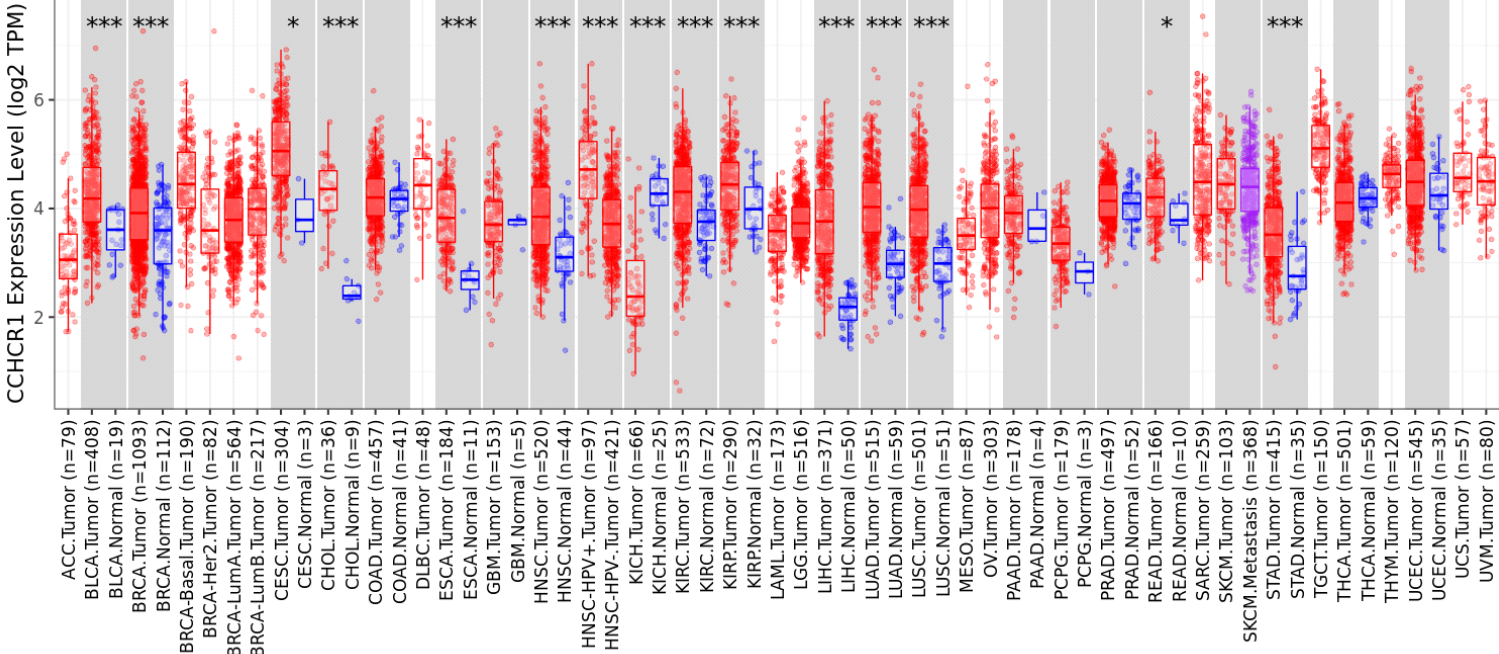

Supplement: Supplementary file 5 — Additional file 5: Fig. S8. Co-localization of HCR with centrin1 and PCM1. HeLa cells transfected with HCR-GFP were co-stain with centrin1 (red) and gamma-tubulin (cyan) and DAPI (blue) or PCM1 (red) and gamma-tubulin (cyan) and DAPI (blue); scale bars, 10 μm. Fig. S9. HCR interacts with CEP131. HeLa cell lysates were immunoprecipitated with antibodies specific for HCR, astrin, or negative control rabbit IgG. The precipitates were analyzed by immunoblotting with antibodies against HCR and CEP131. Fig. S10. HCR did not interact with SKAP, MYCBP and LC8. HeLa cell lysates were immunoprecipitated with astrin, HCR, or control rabbit IgG antibodies and analyzed by western blotting with astrin, HCR, SKAP, MYCBP and LC8 antibodies. Beta actin antibody was used as negative control; HeLa cell lysates were immunoprecipitated with SKAP, MYCBP and LC8, or control rabbit IgG antibodies and analyzed by western blotting with astrin, HCR, SKAP, MYCBP and LC8 antibodies. Beta actin antibody was used as negative control. Fig. S11. HCR interacts with NuMA. mCherry vector alone or HCR-mCherry was transfected into HeLa cells for immunoprecipitation with mCherry antibody. The precipitates were analyzed by immunoblotting with HCR and NuMA antibodies. Fig. S12. HCR co-localizes with astrin and EDC4.HCR-GFP-transfected HeLa cells were treated with arsenite for 30 min and then co-stained with astrin (red) and EDC4 (cyan) for immunofluorescence detection; scale bars, 10 μm. Fig. S13. HCR co-localizes with EDC4 in mitosis. Negative control, HCR siRNA-treated HeLa cells were co-stained with HCR (red) and EDC4 (green); scale bars, 10 μm. Fig. S14. HCR was closely related to tumorigenesis. The dataset was from TCGA (portal.gdc.cancer.gov) and analyzed by TIMER 2.0 (cistrome.org). [file 12915_2022_1437_MOESM5_ESM.pdf]

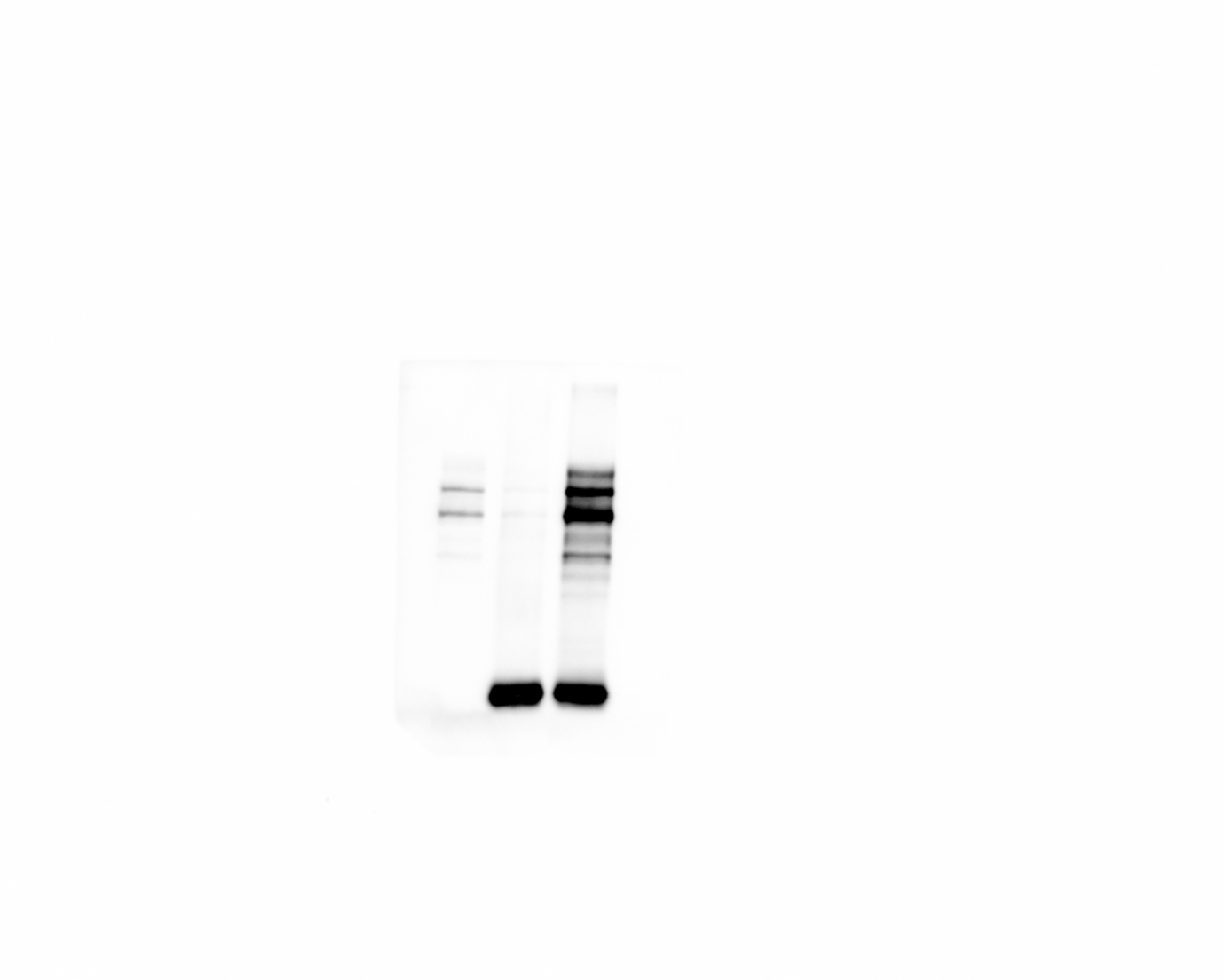

Supplement: Supplementary file 10 — Additional file 10. All Original and uncropped blots images used in manuscript. [file 12915_2022_1437_MOESM10_ESM.zip › blots images/Fig 1/Fig 1 A/astrin-coIP-HCR-anti-astrin.tif]

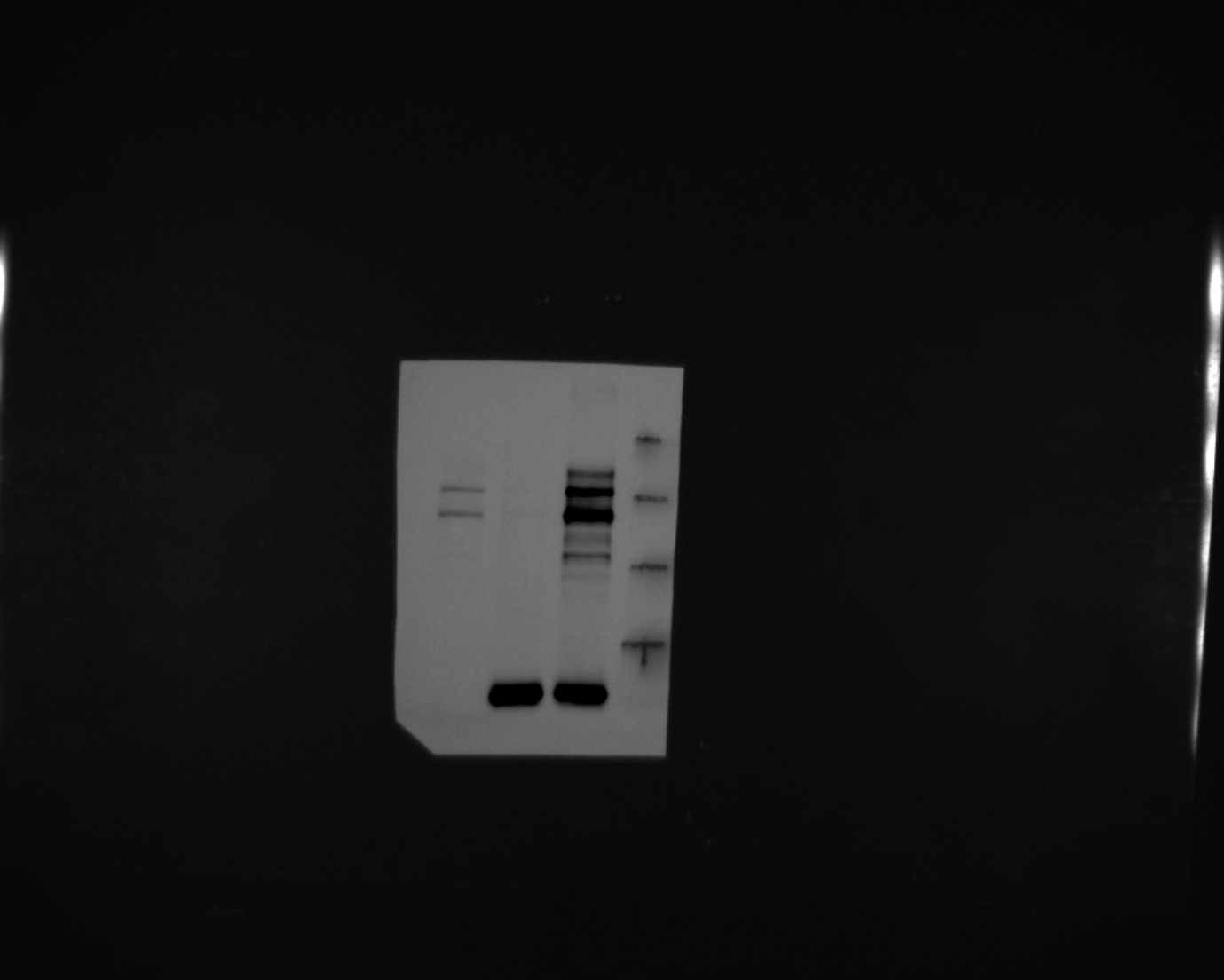

Supplement: Supplementary file 10 — Additional file 10. All Original and uncropped blots images used in manuscript. [file 12915_2022_1437_MOESM10_ESM.zip › blots images/Fig 1/Fig 1 A/astrin-coIP-HCR-anti-astrin_marker.tif]

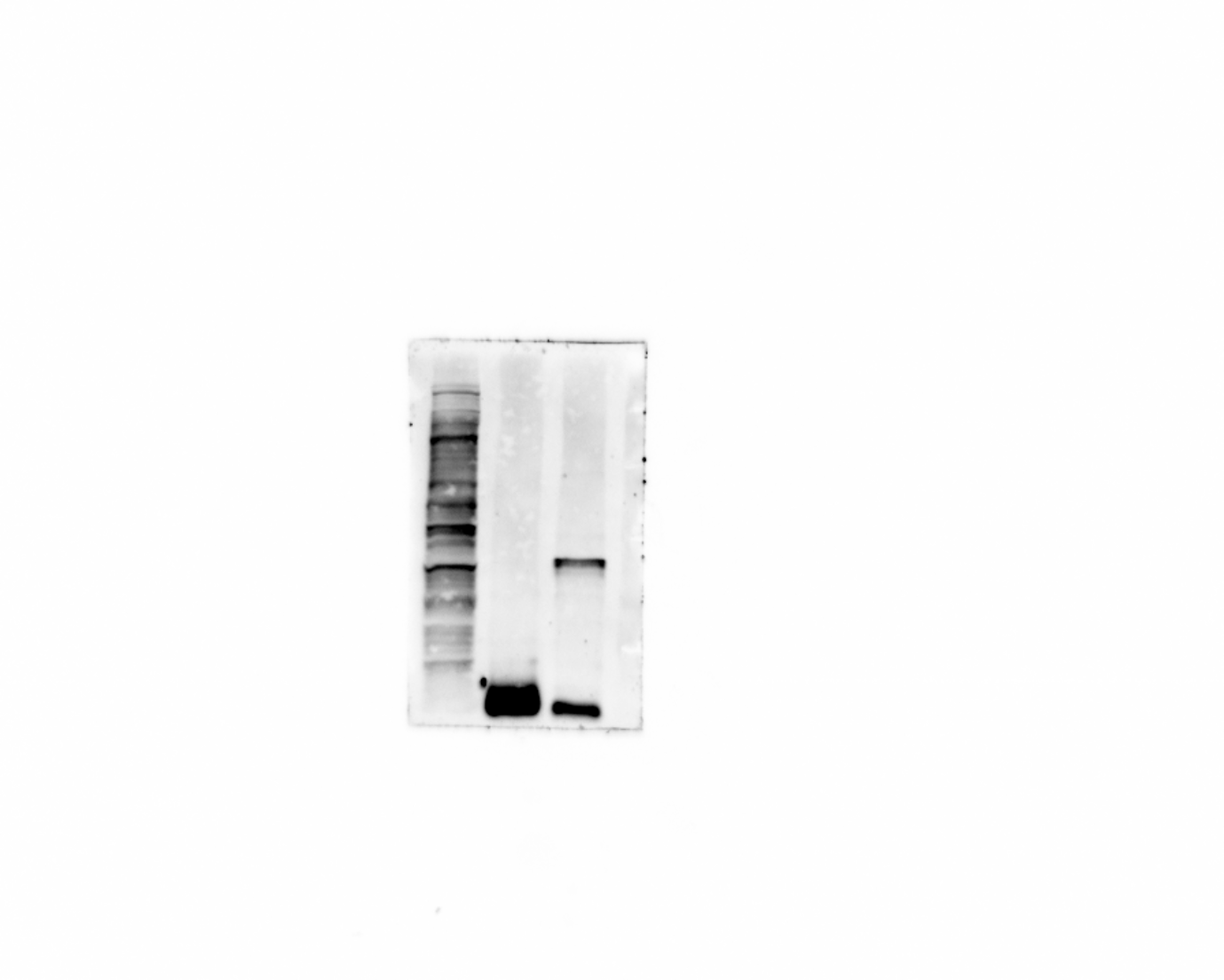

Supplement: Supplementary file 10 — Additional file 10. All Original and uncropped blots images used in manuscript. [file 12915_2022_1437_MOESM10_ESM.zip › blots images/Fig 1/Fig 1 A/astrin-coIP-HCR-anti-HCR.tif]

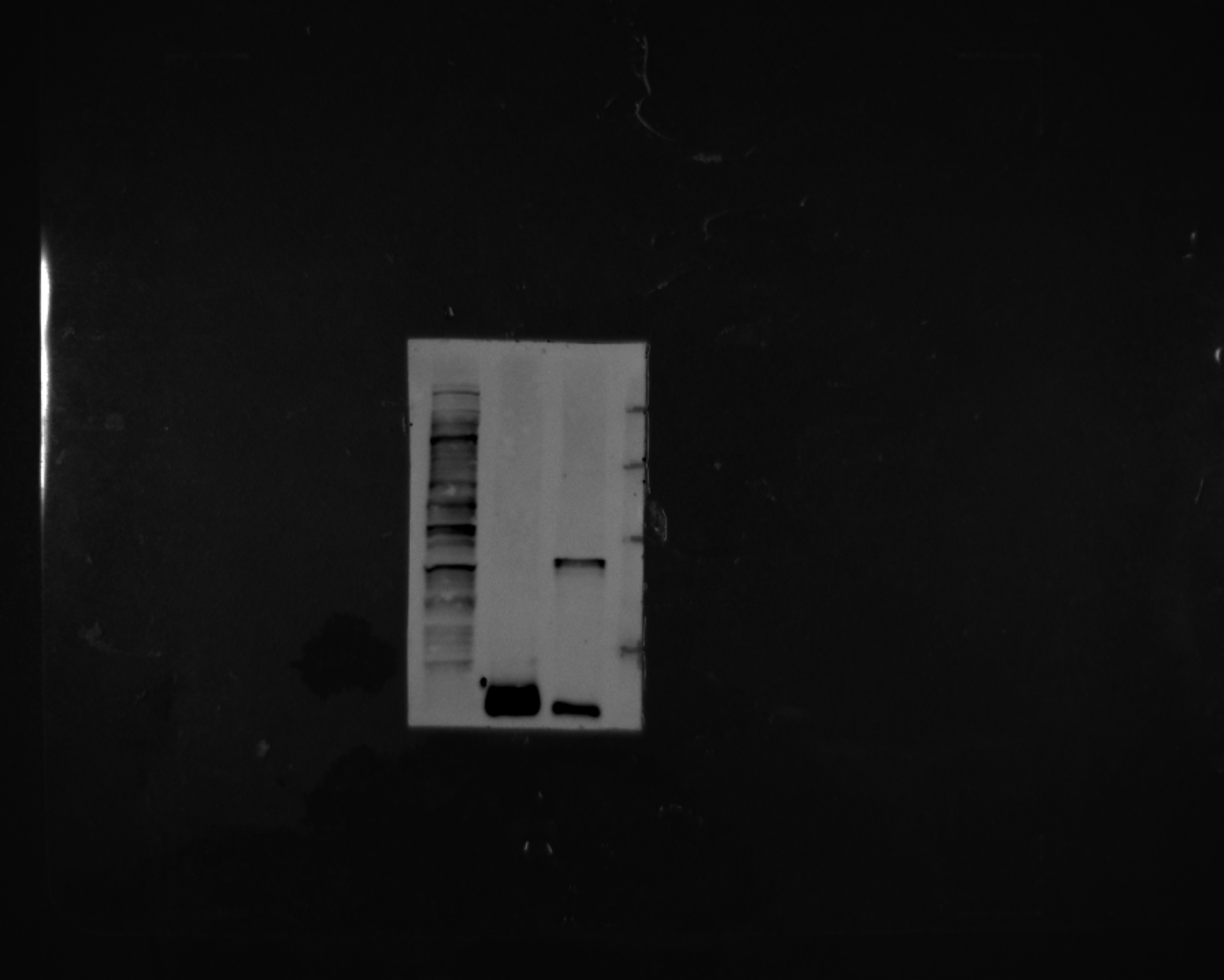

Supplement: Supplementary file 10 — Additional file 10. All Original and uncropped blots images used in manuscript. [file 12915_2022_1437_MOESM10_ESM.zip › blots images/Fig 1/Fig 1 A/astrin-coIP-HCR-anti-HCR_marker.tif]

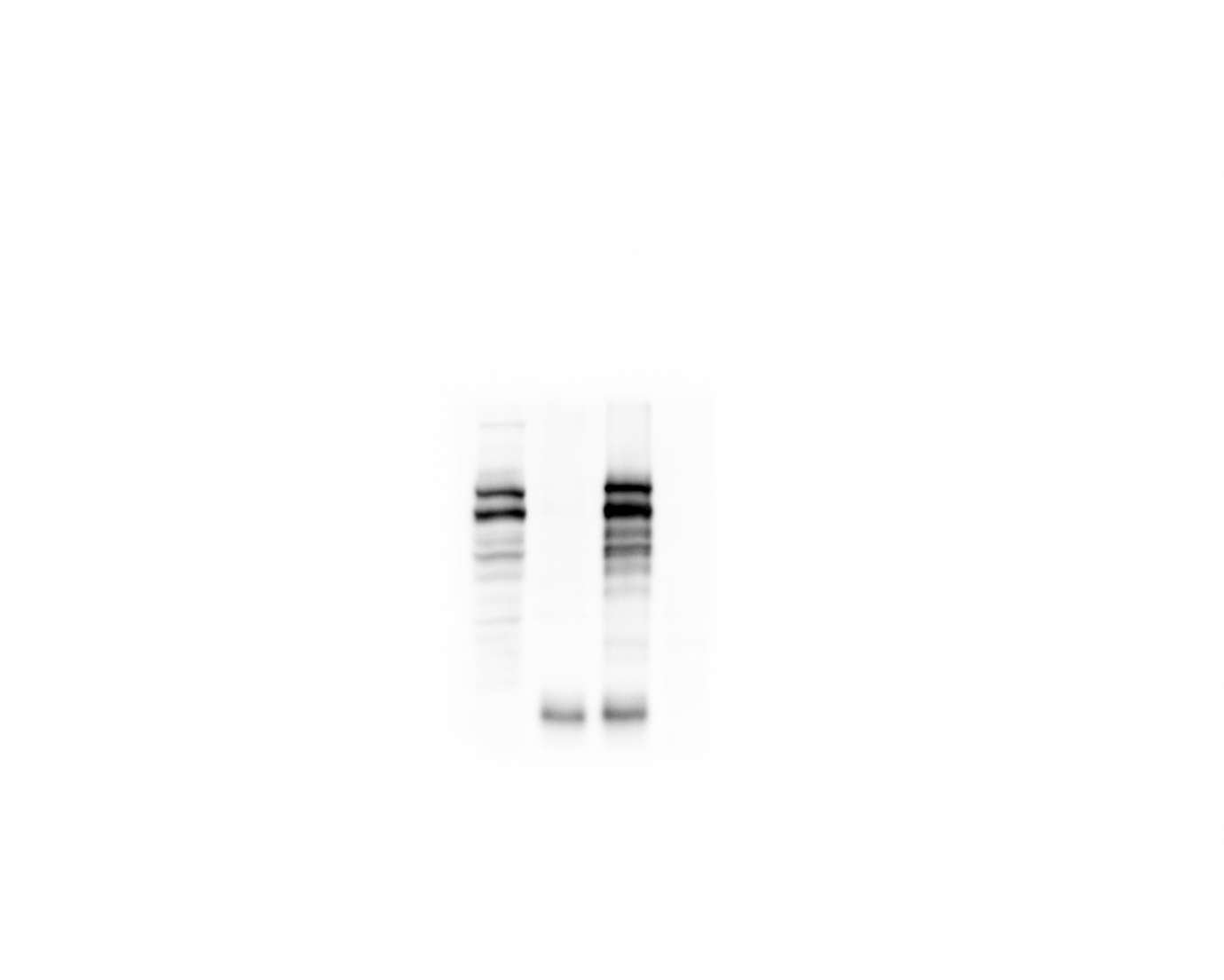

Supplement: Supplementary file 10 — Additional file 10. All Original and uncropped blots images used in manuscript. [file 12915_2022_1437_MOESM10_ESM.zip › blots images/Fig 1/Fig 1 A/HCR-coIP-astrin-anti-astrin.tif]

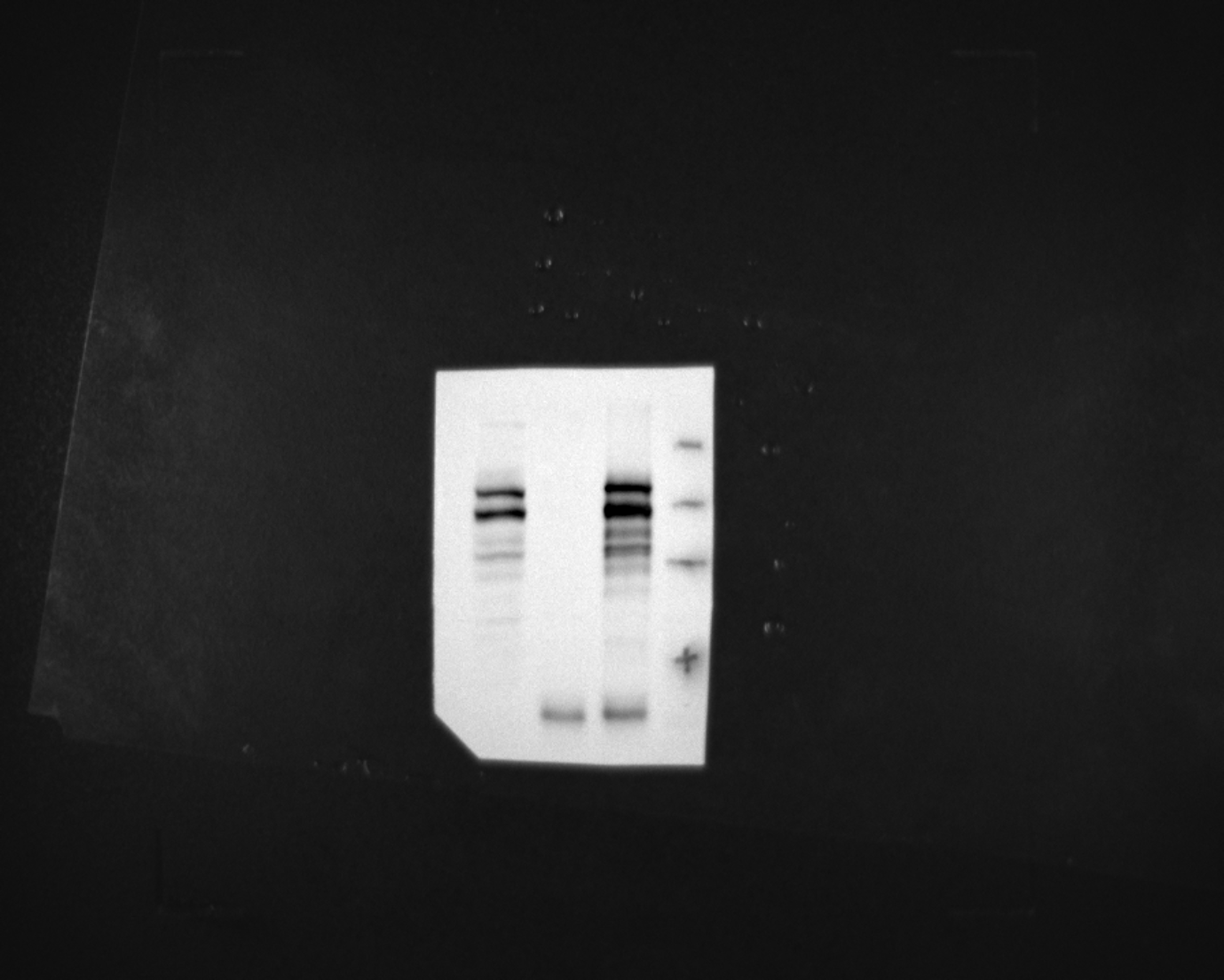

Supplement: Supplementary file 10 — Additional file 10. All Original and uncropped blots images used in manuscript. [file 12915_2022_1437_MOESM10_ESM.zip › blots images/Fig 1/Fig 1 A/HCR-coIP-astrin-anti-astrin_marker.tif]

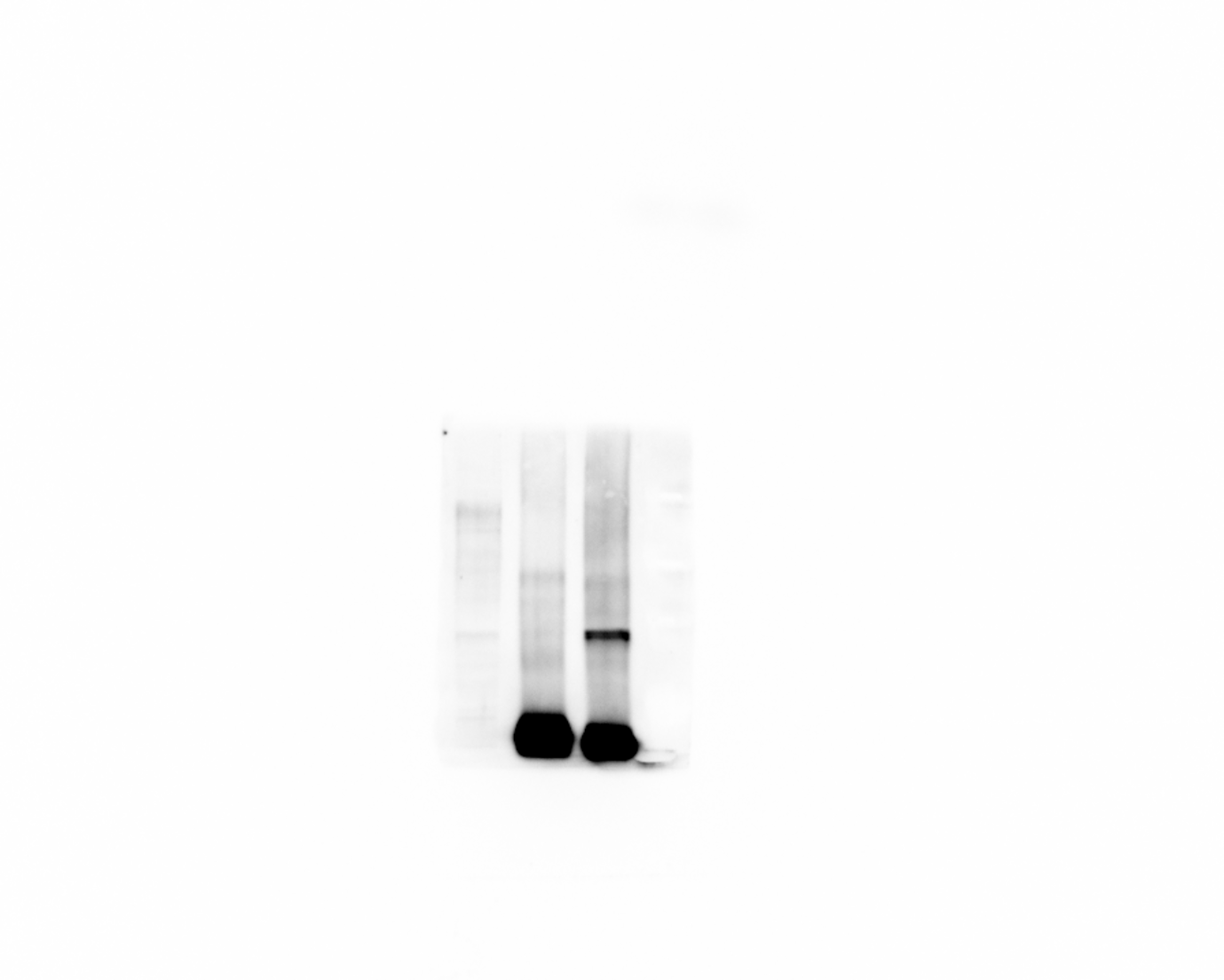

Supplement: Supplementary file 10 — Additional file 10. All Original and uncropped blots images used in manuscript. [file 12915_2022_1437_MOESM10_ESM.zip › blots images/Fig 1/Fig 1 A/HCR-coIP-astrin-anti-HCR.tif]

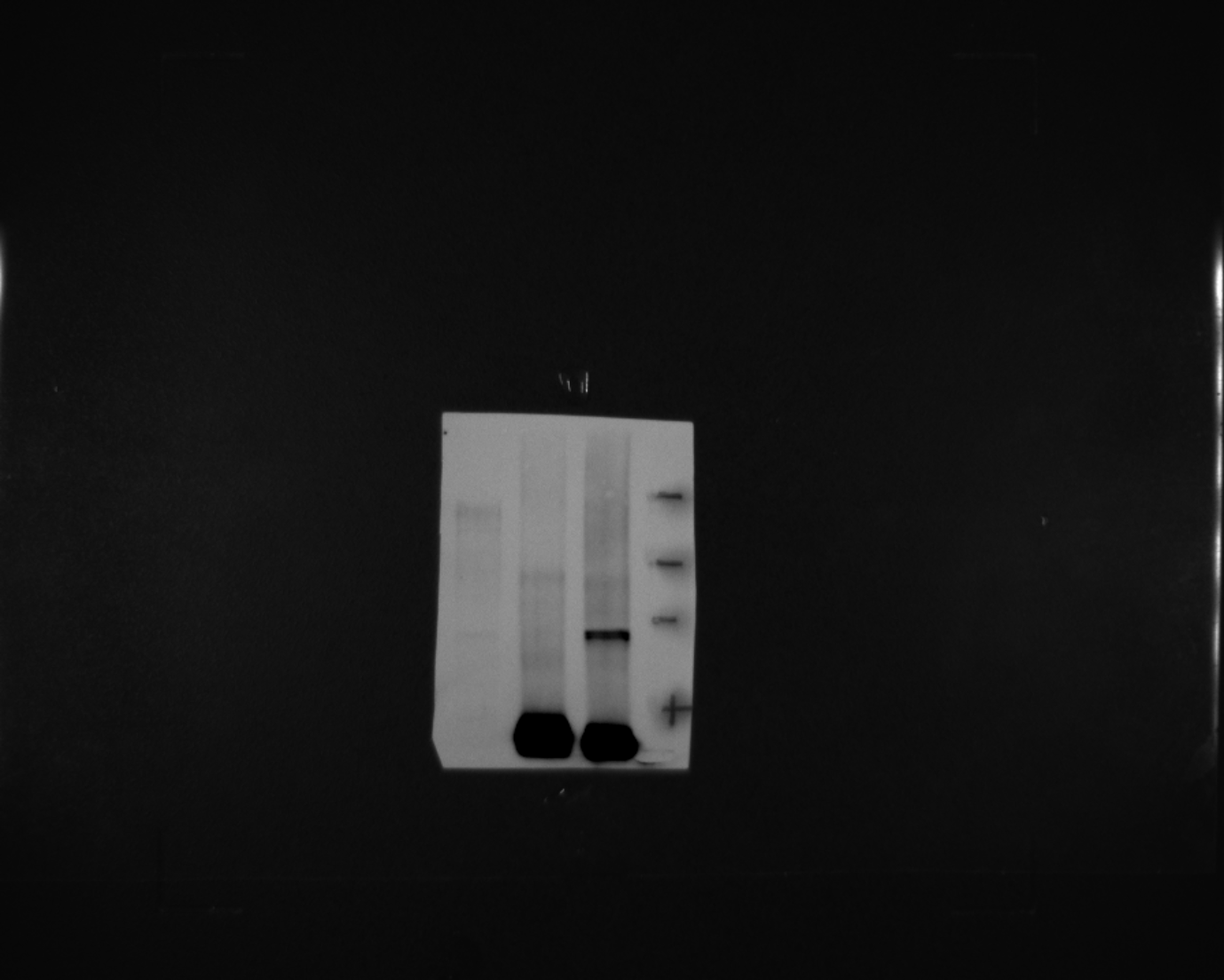

Supplement: Supplementary file 10 — Additional file 10. All Original and uncropped blots images used in manuscript. [file 12915_2022_1437_MOESM10_ESM.zip › blots images/Fig 1/Fig 1 A/HCR-coIP-astrin-anti-HCR_withmarker.tif]

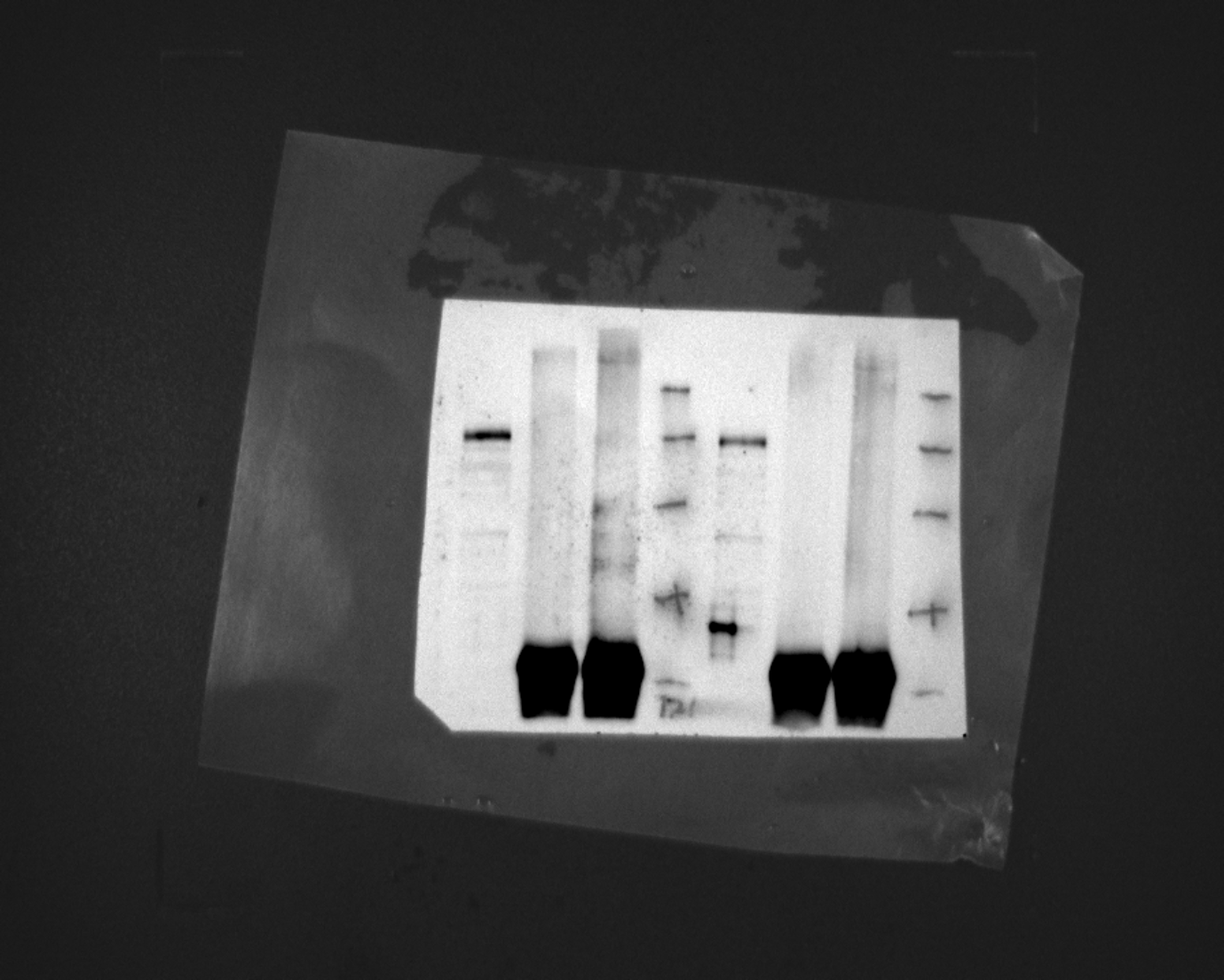

Supplement: Supplementary file 10 — Additional file 10. All Original and uncropped blots images used in manuscript. [file 12915_2022_1437_MOESM10_ESM.zip › blots images/Fig 1/Fig 1 A/Left-astrin-coIP-HCR-Right-HCR-coIP-astrin-anti-GM130 with marker.tif]

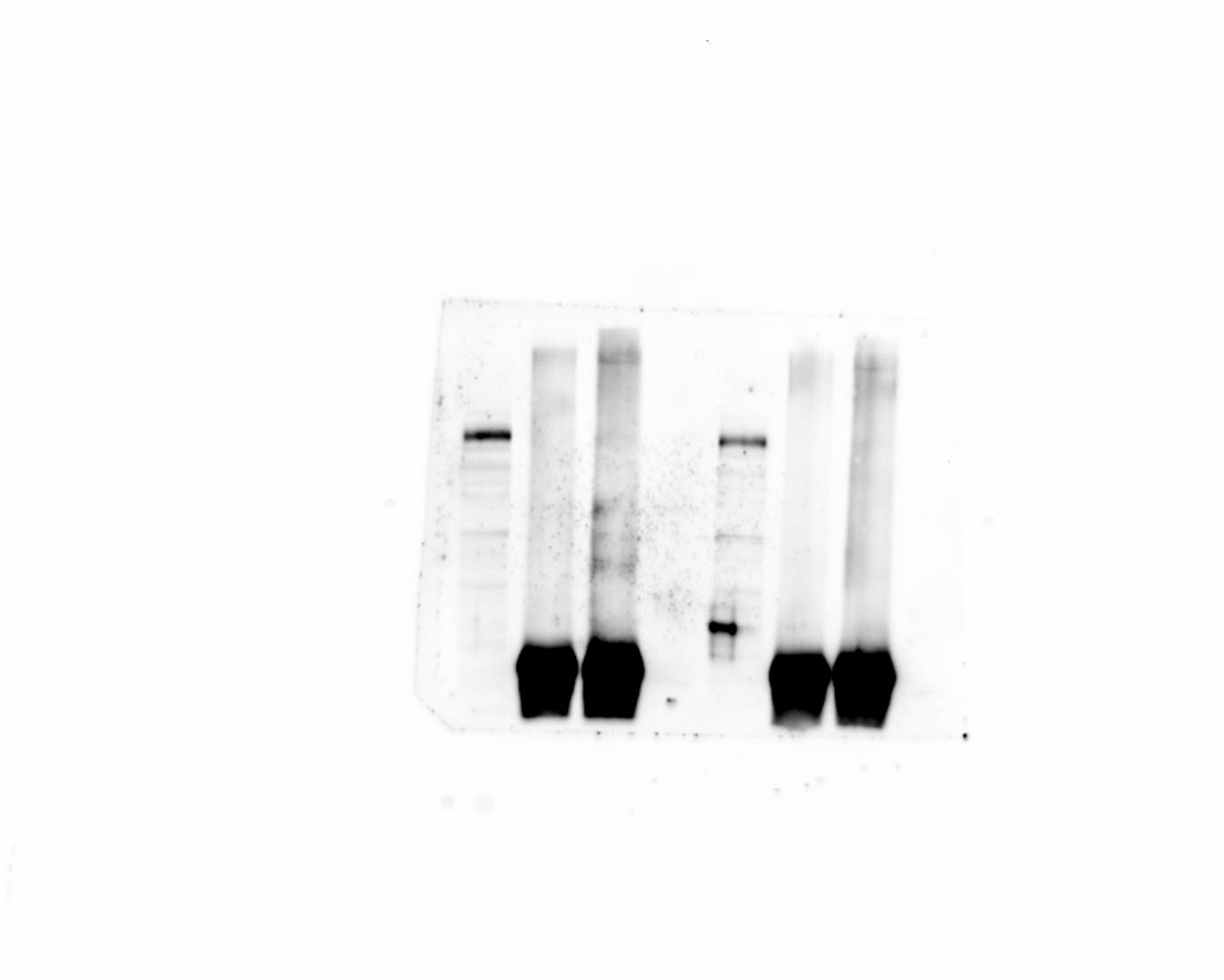

Supplement: Supplementary file 10 — Additional file 10. All Original and uncropped blots images used in manuscript. [file 12915_2022_1437_MOESM10_ESM.zip › blots images/Fig 1/Fig 1 A/Left-astrin-coIP-HCR-Right-HCR-coIP-astrin-anti-GM130.tif]

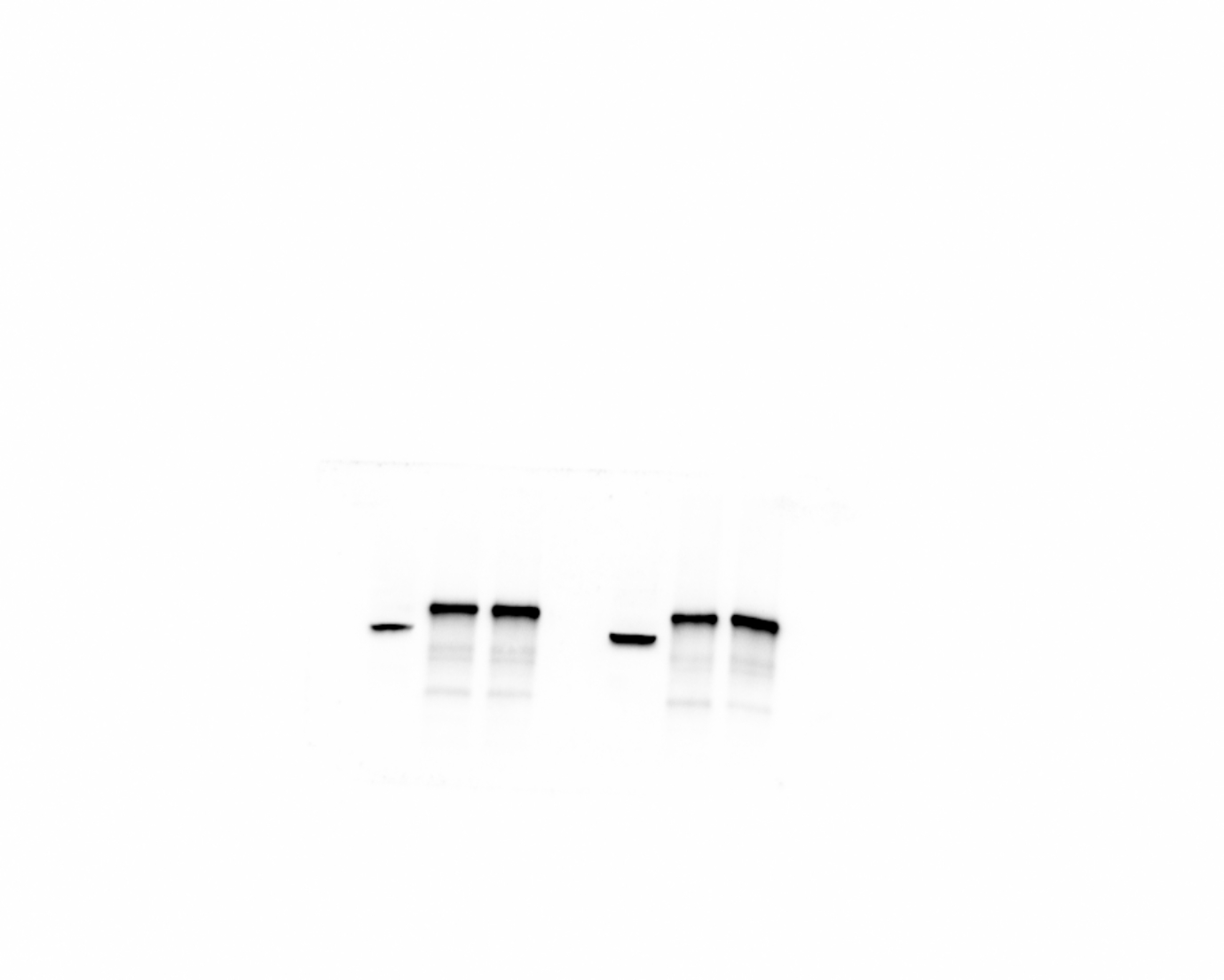

Supplement: Supplementary file 10 — Additional file 10. All Original and uncropped blots images used in manuscript. [file 12915_2022_1437_MOESM10_ESM.zip › blots images/Fig 1/Fig 1 A/Left-HCR-coIP-HCR-astrin-astrin-coIP-HCR-anti-GM130.tif]

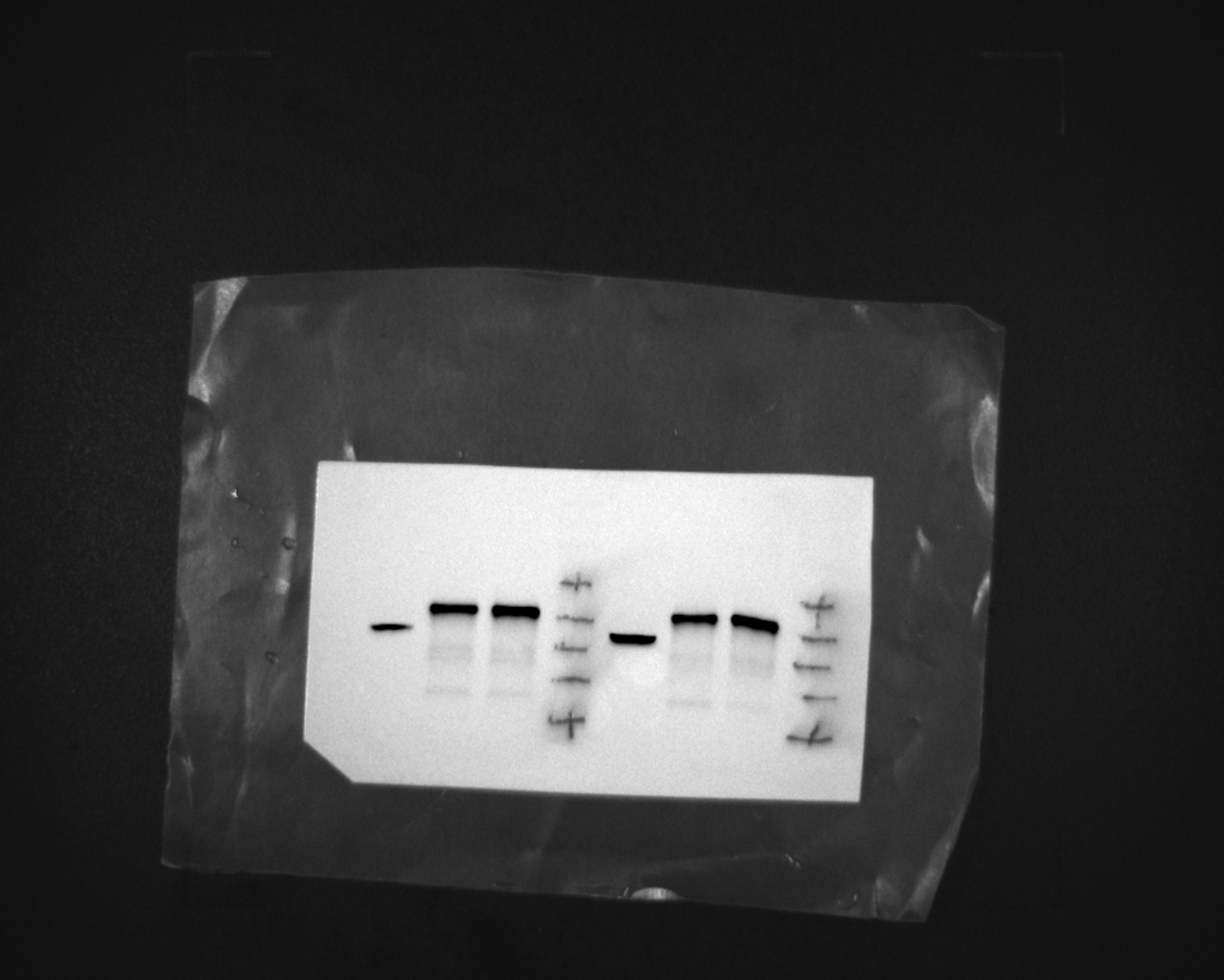

Supplement: Supplementary file 10 — Additional file 10. All Original and uncropped blots images used in manuscript. [file 12915_2022_1437_MOESM10_ESM.zip › blots images/Fig 1/Fig 1 A/Left-HCR-coIP-HCR-astrin-astrin-coIP-HCR-anti-GM130_marker.tif]

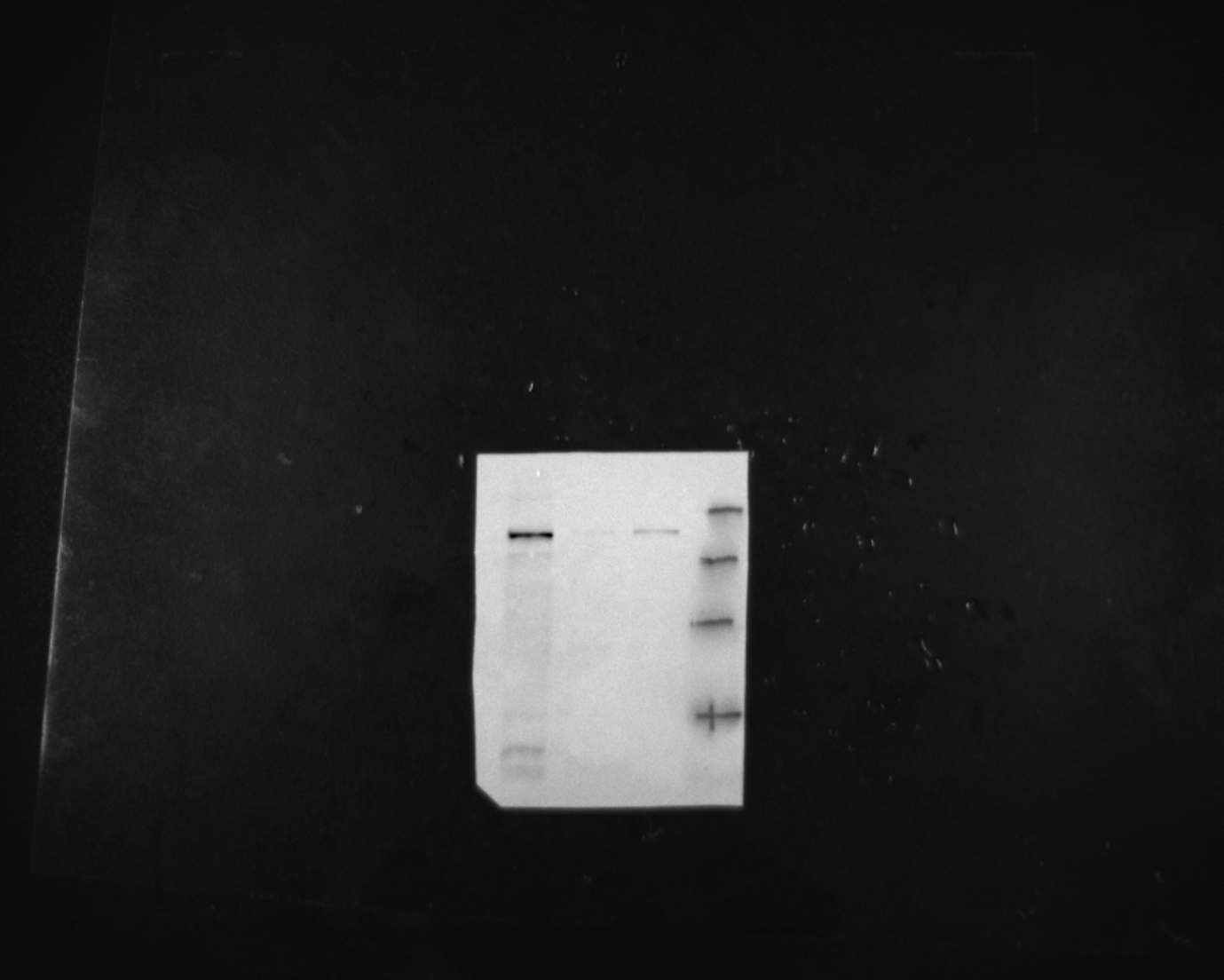

Supplement: Supplementary file 10 — Additional file 10. All Original and uncropped blots images used in manuscript. [file 12915_2022_1437_MOESM10_ESM.zip › blots images/Fig 1/Fig 1 B/Fig1B-GST-pulldown-GFP-astrin-marker.tif]

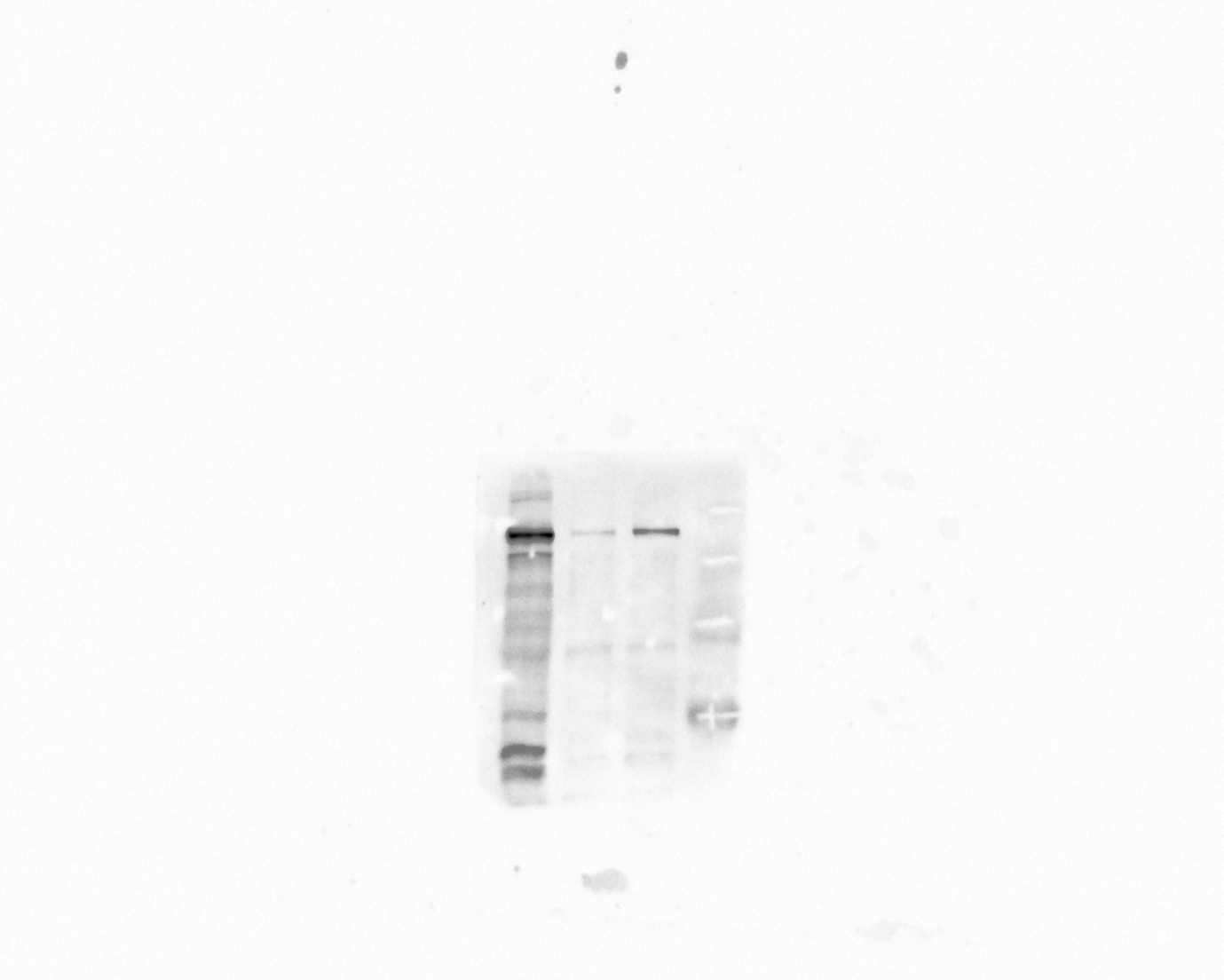

Supplement: Supplementary file 10 — Additional file 10. All Original and uncropped blots images used in manuscript. [file 12915_2022_1437_MOESM10_ESM.zip › blots images/Fig 1/Fig 1 B/Fig1B-GST-pulldown-GFP-astrin.tif]

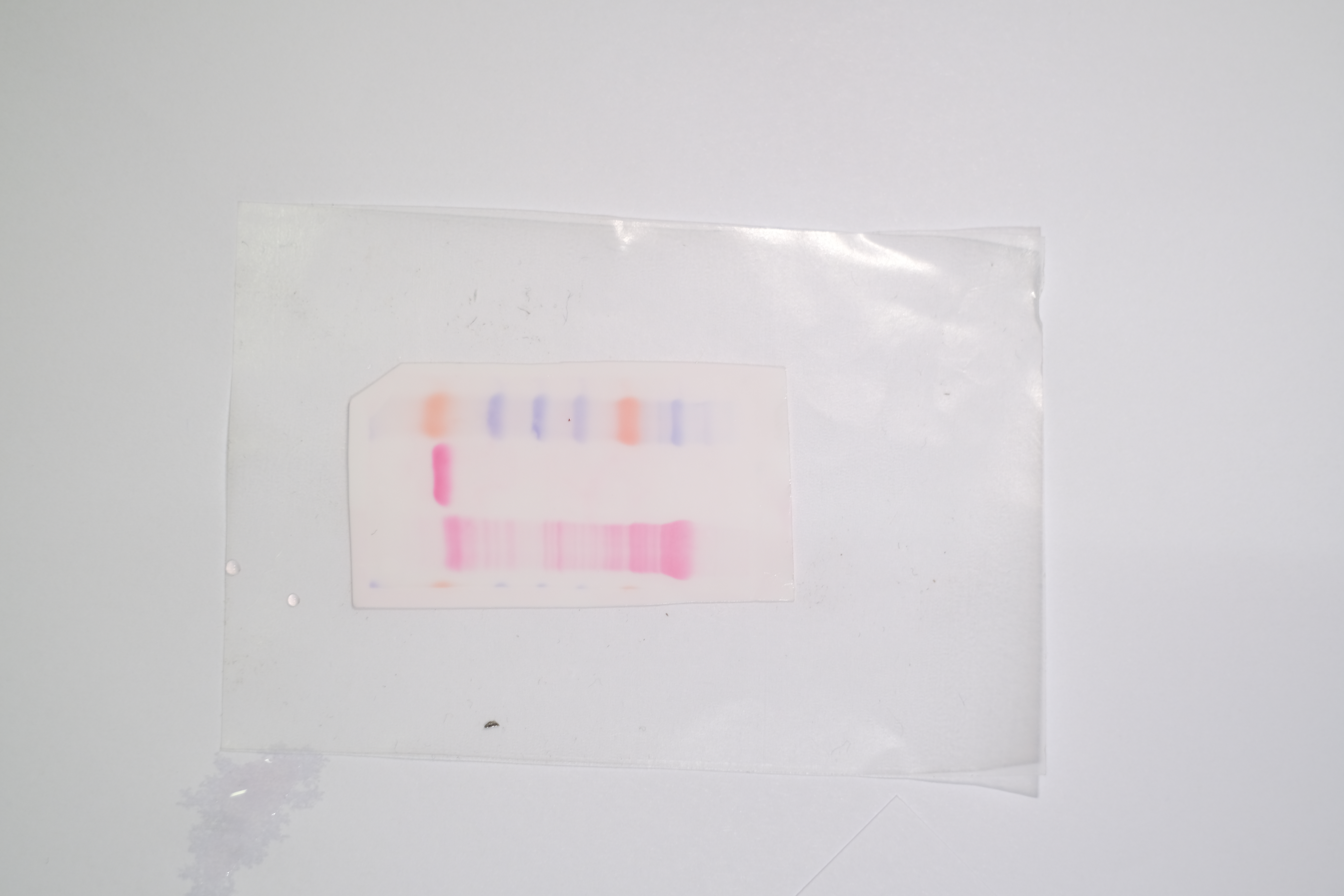

Supplement: Supplementary file 10 — Additional file 10. All Original and uncropped blots images used in manuscript. [file 12915_2022_1437_MOESM10_ESM.zip › blots images/Fig 1/Fig 1 B/GST-HCR.JPG]

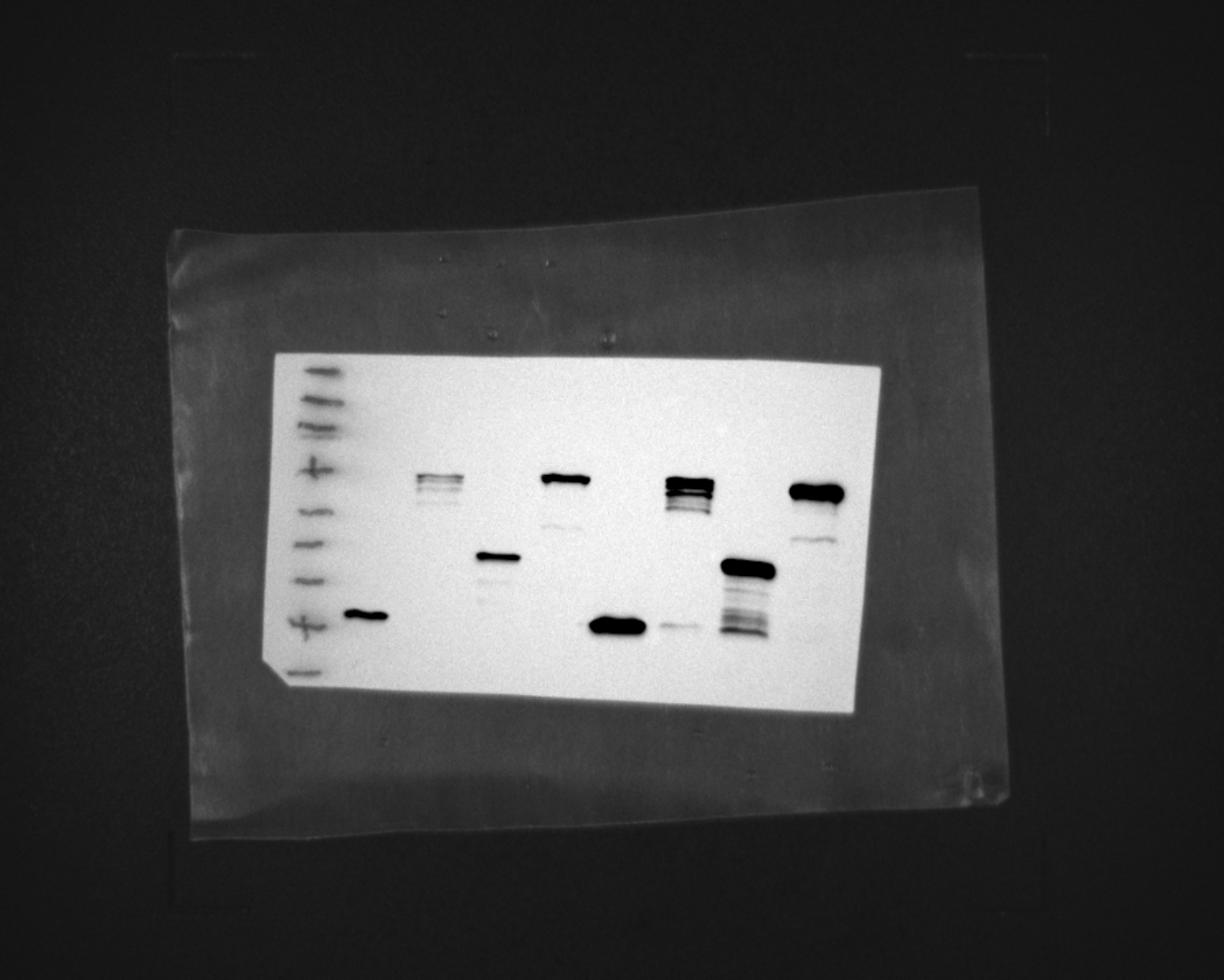

Supplement: Supplementary file 10 — Additional file 10. All Original and uncropped blots images used in manuscript. [file 12915_2022_1437_MOESM10_ESM.zip › blots images/Fig 1/Fig 1 D/GFP-IP-astrin-myc-anti-GFP-marker.tif]

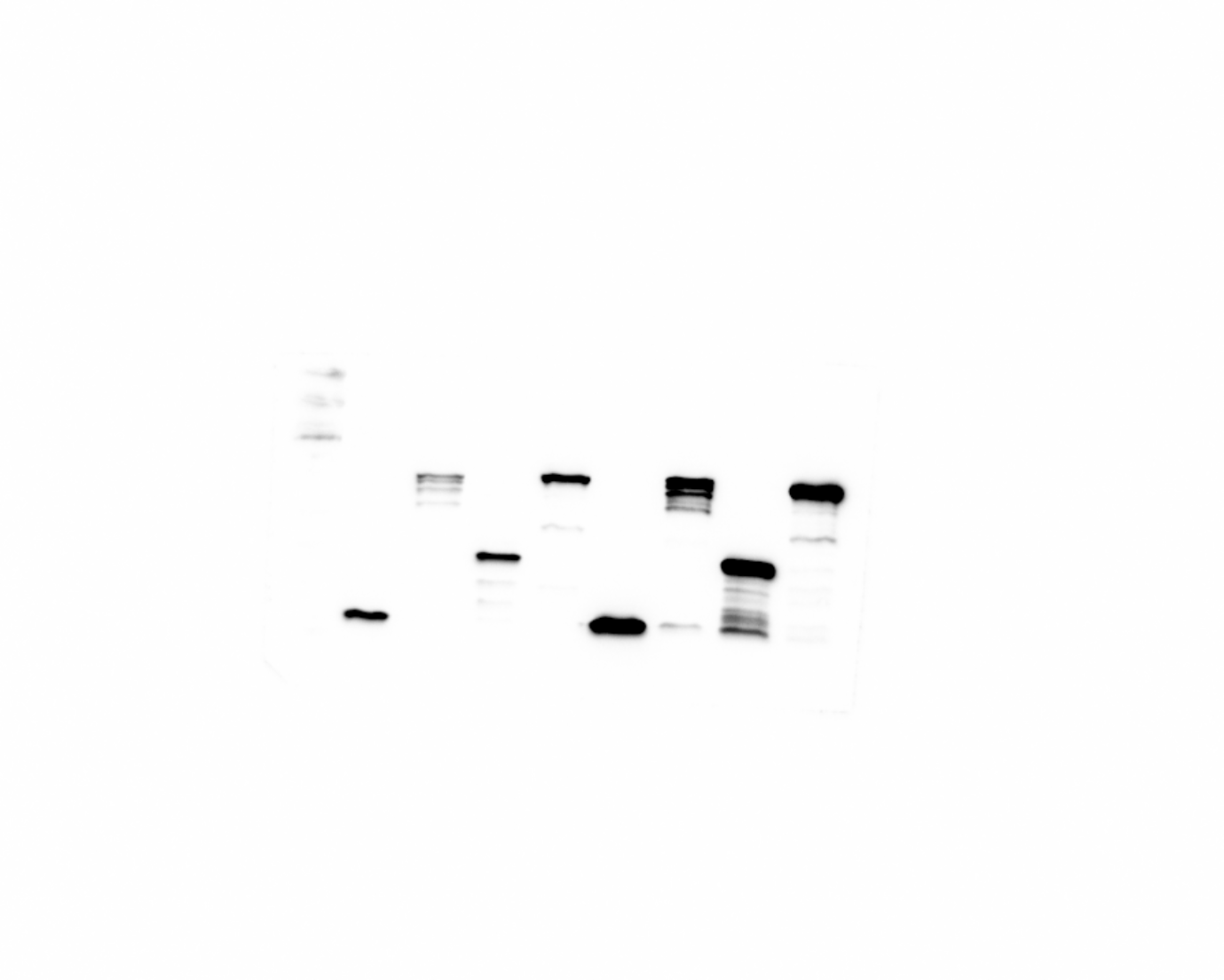

Supplement: Supplementary file 10 — Additional file 10. All Original and uncropped blots images used in manuscript. [file 12915_2022_1437_MOESM10_ESM.zip › blots images/Fig 1/Fig 1 D/GFP-IP-astrin-myc-anti-GFP.tif]

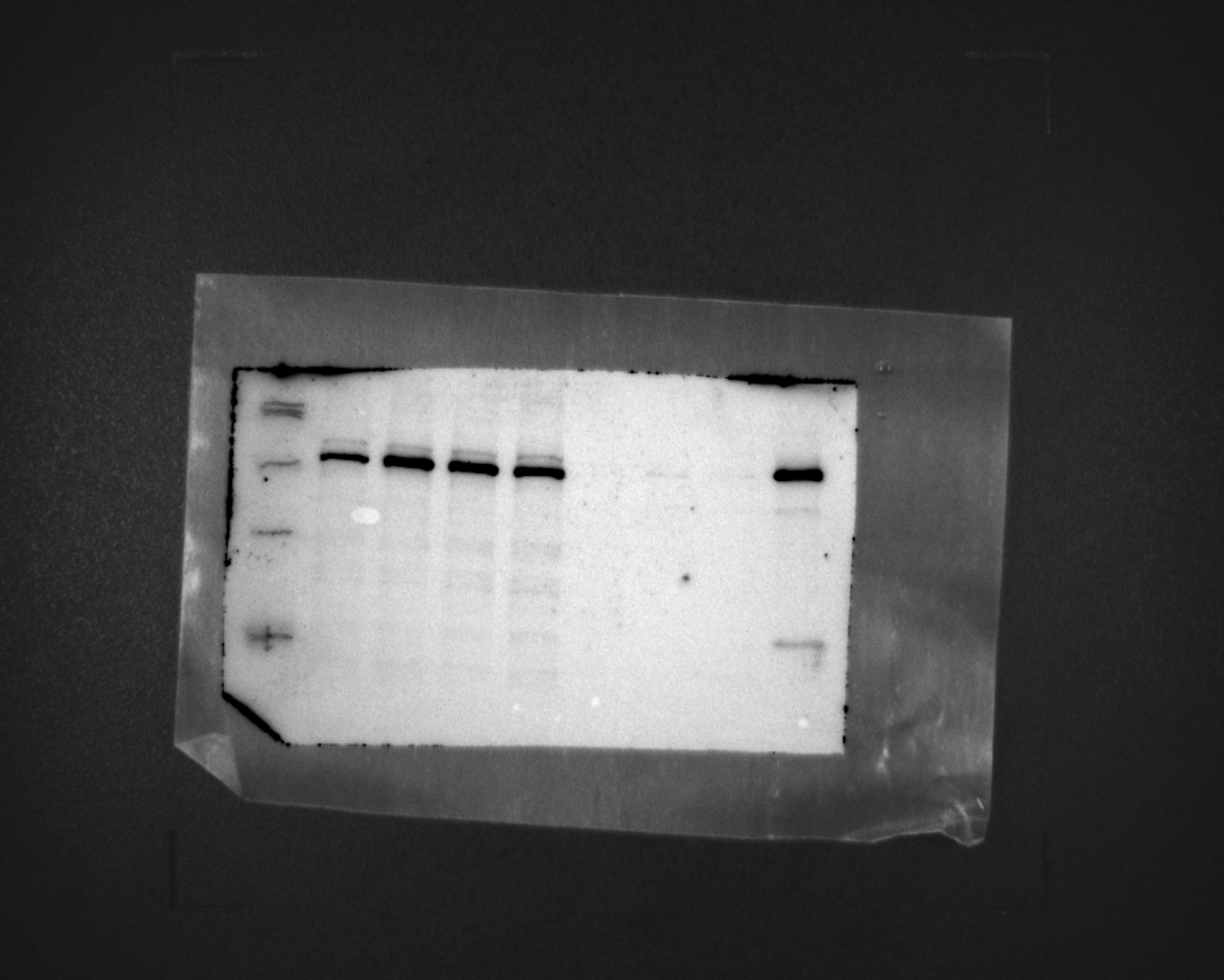

Supplement: Supplementary file 10 — Additional file 10. All Original and uncropped blots images used in manuscript. [file 12915_2022_1437_MOESM10_ESM.zip › blots images/Fig 1/Fig 1 D/GFP-IP-astrin-myc-anti-myc-marker.tif]

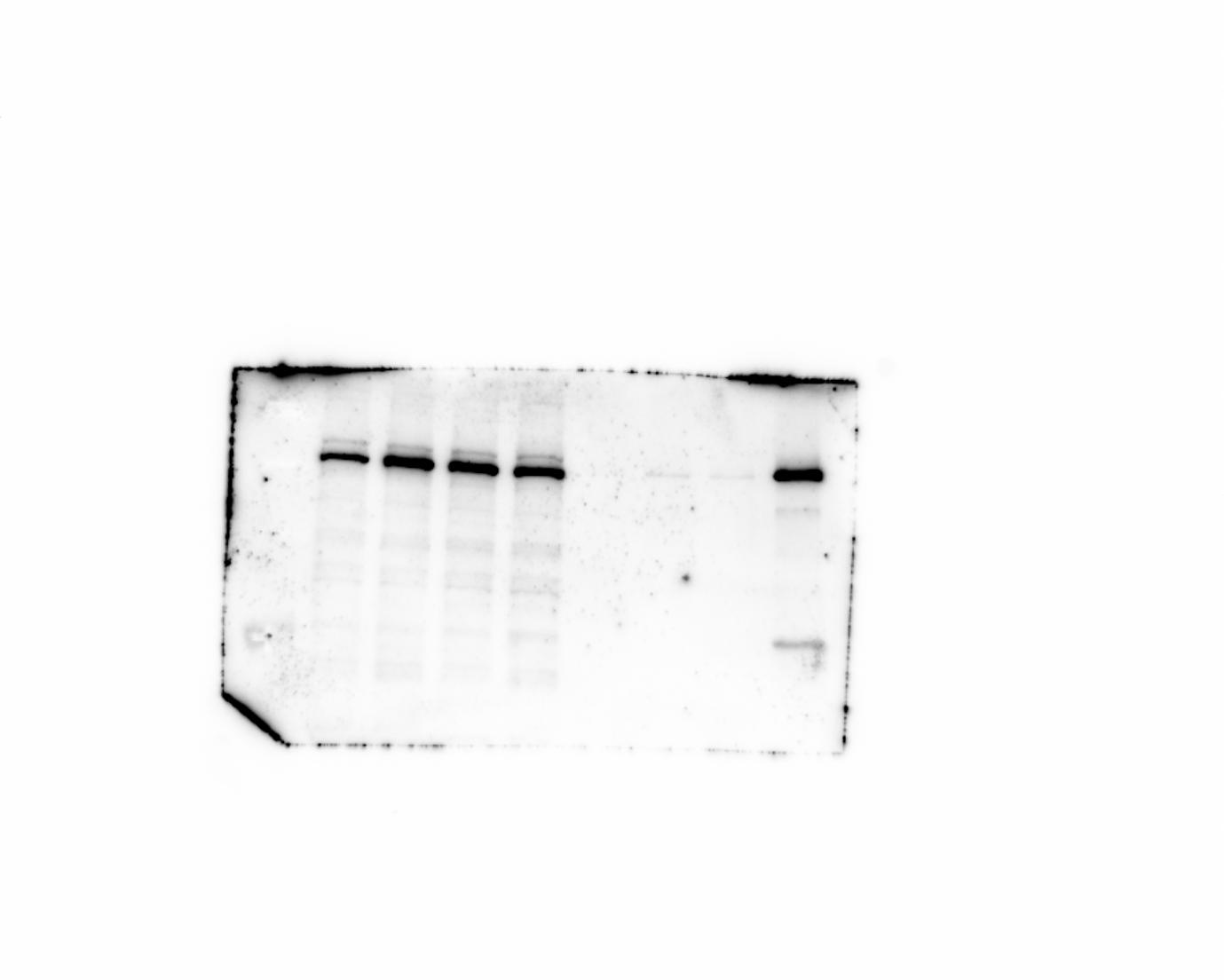

Supplement: Supplementary file 10 — Additional file 10. All Original and uncropped blots images used in manuscript. [file 12915_2022_1437_MOESM10_ESM.zip › blots images/Fig 1/Fig 1 D/GFP-IP-astrin-myc-anti-myc.tif]

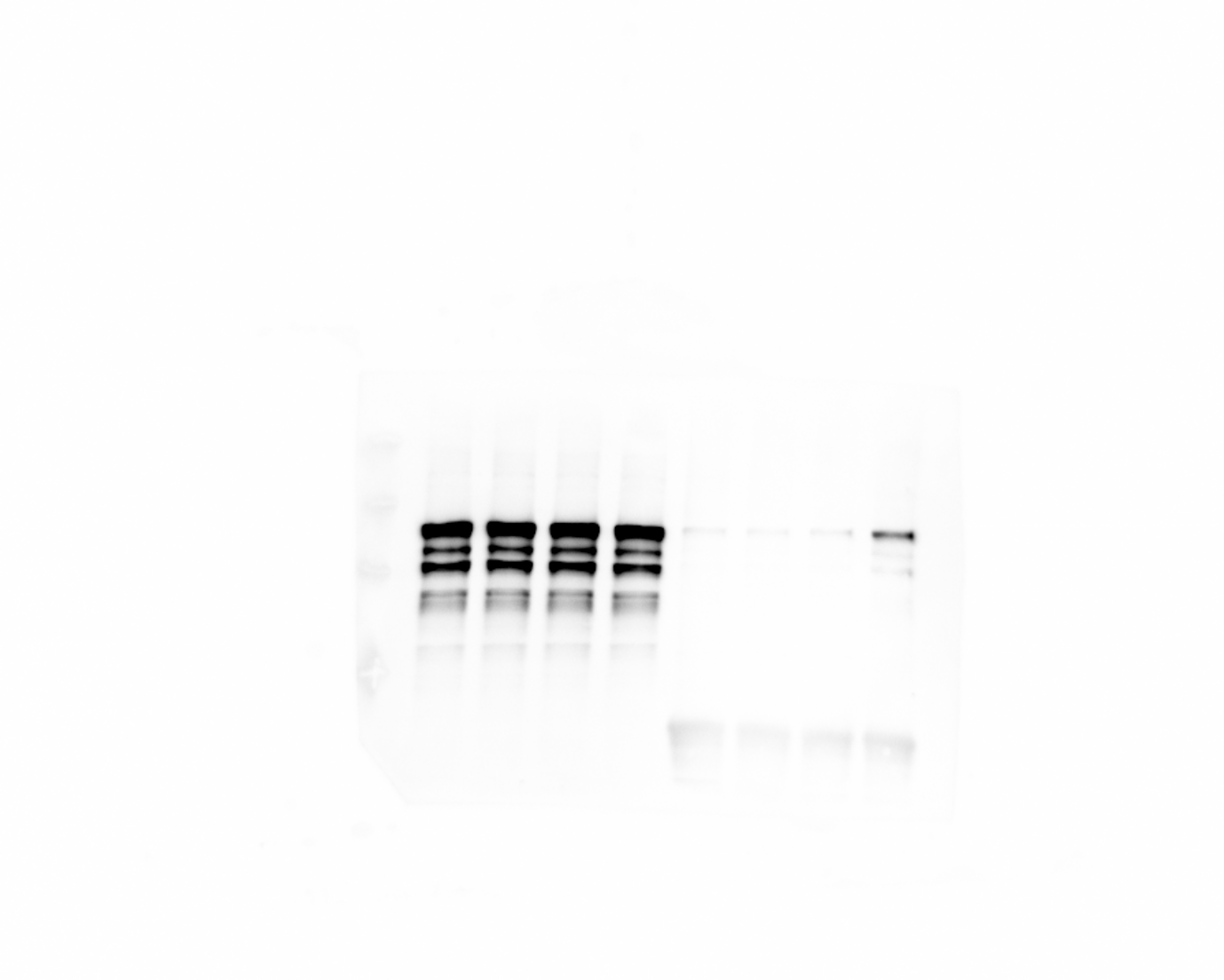

Supplement: Supplementary file 10 — Additional file 10. All Original and uncropped blots images used in manuscript. [file 12915_2022_1437_MOESM10_ESM.zip › blots images/Fig 1/Fig 1 E/myc-IP-HCR-GFP-anti-GFP.tif]

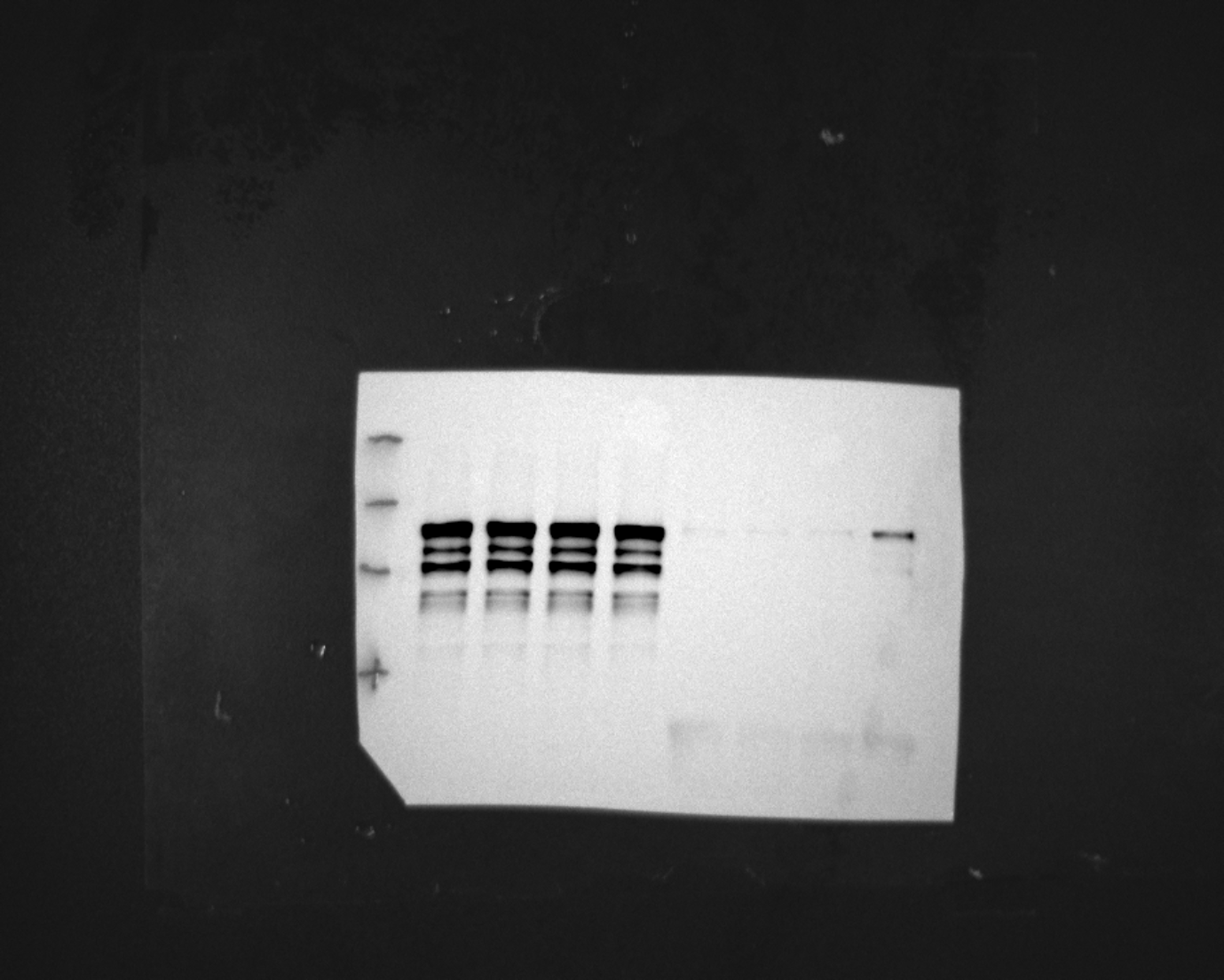

Supplement: Supplementary file 10 — Additional file 10. All Original and uncropped blots images used in manuscript. [file 12915_2022_1437_MOESM10_ESM.zip › blots images/Fig 1/Fig 1 E/myc-IP-HCR-GFP-anti-GFP_marker.tif]

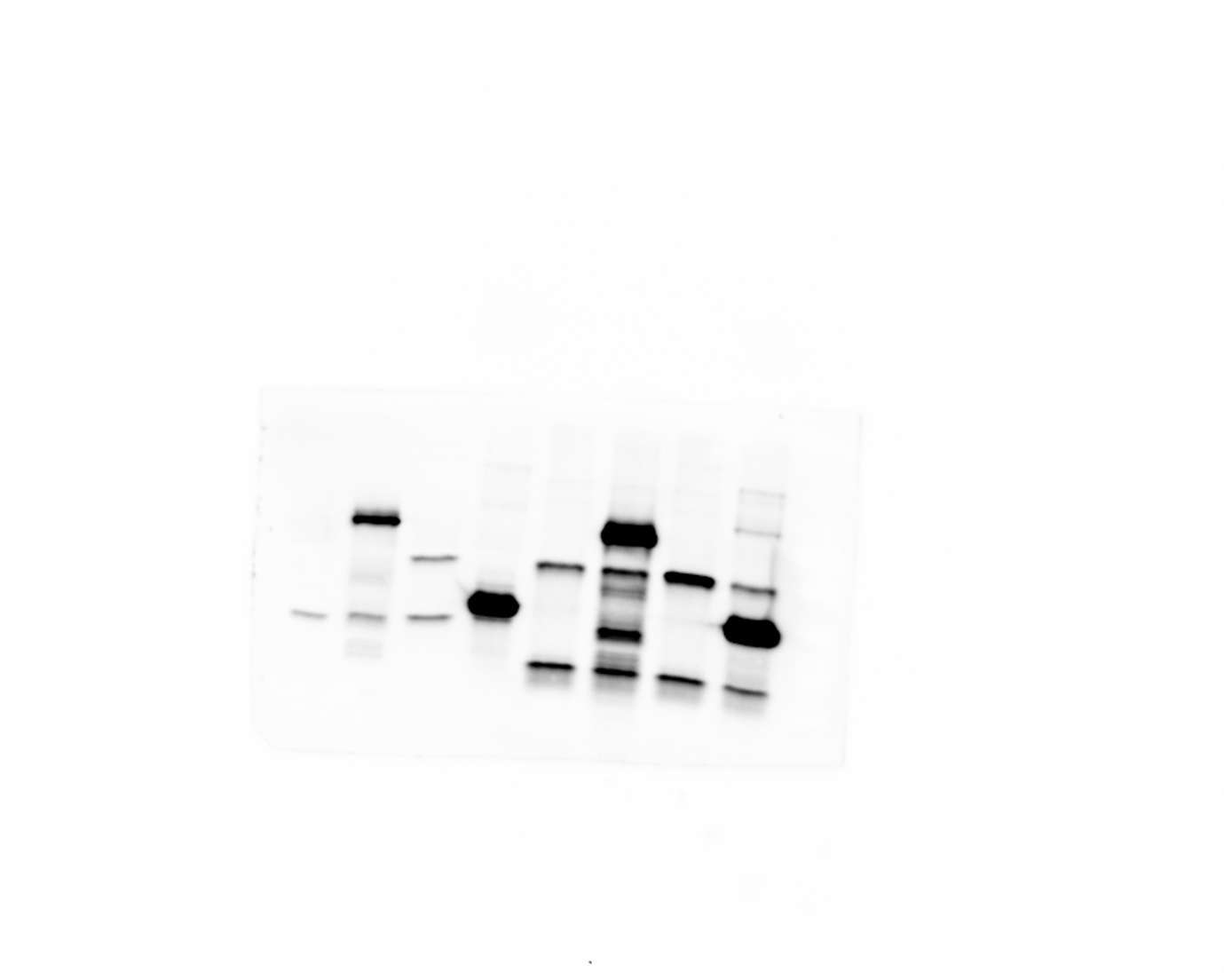

Supplement: Supplementary file 10 — Additional file 10. All Original and uncropped blots images used in manuscript. [file 12915_2022_1437_MOESM10_ESM.zip › blots images/Fig 1/Fig 1 E/myc-IP-HCR-GFP-anti-myc.tif]

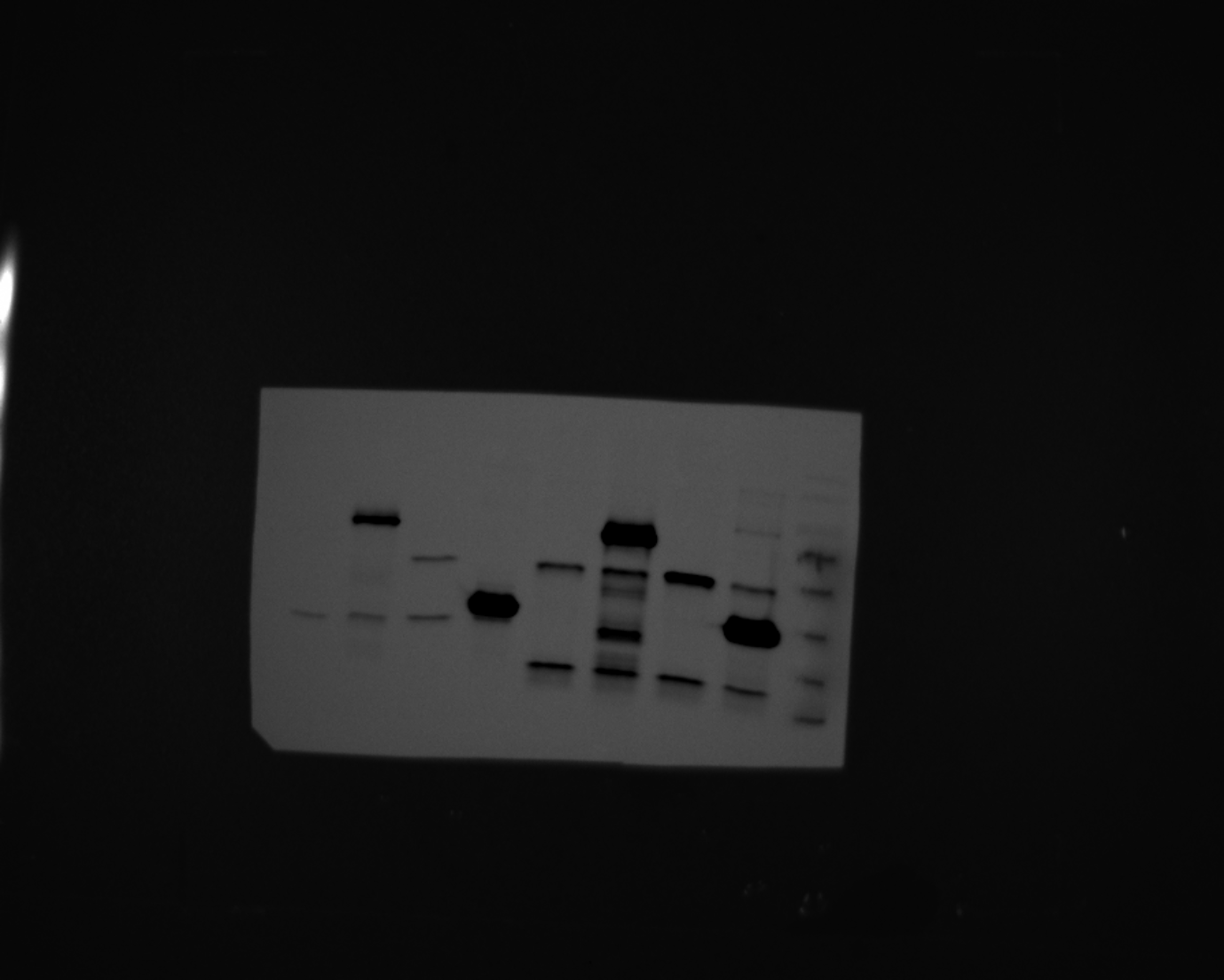

Supplement: Supplementary file 10 — Additional file 10. All Original and uncropped blots images used in manuscript. [file 12915_2022_1437_MOESM10_ESM.zip › blots images/Fig 1/Fig 1 E/myc-IP-HCR-GFP-anti-myc_marker.tif]

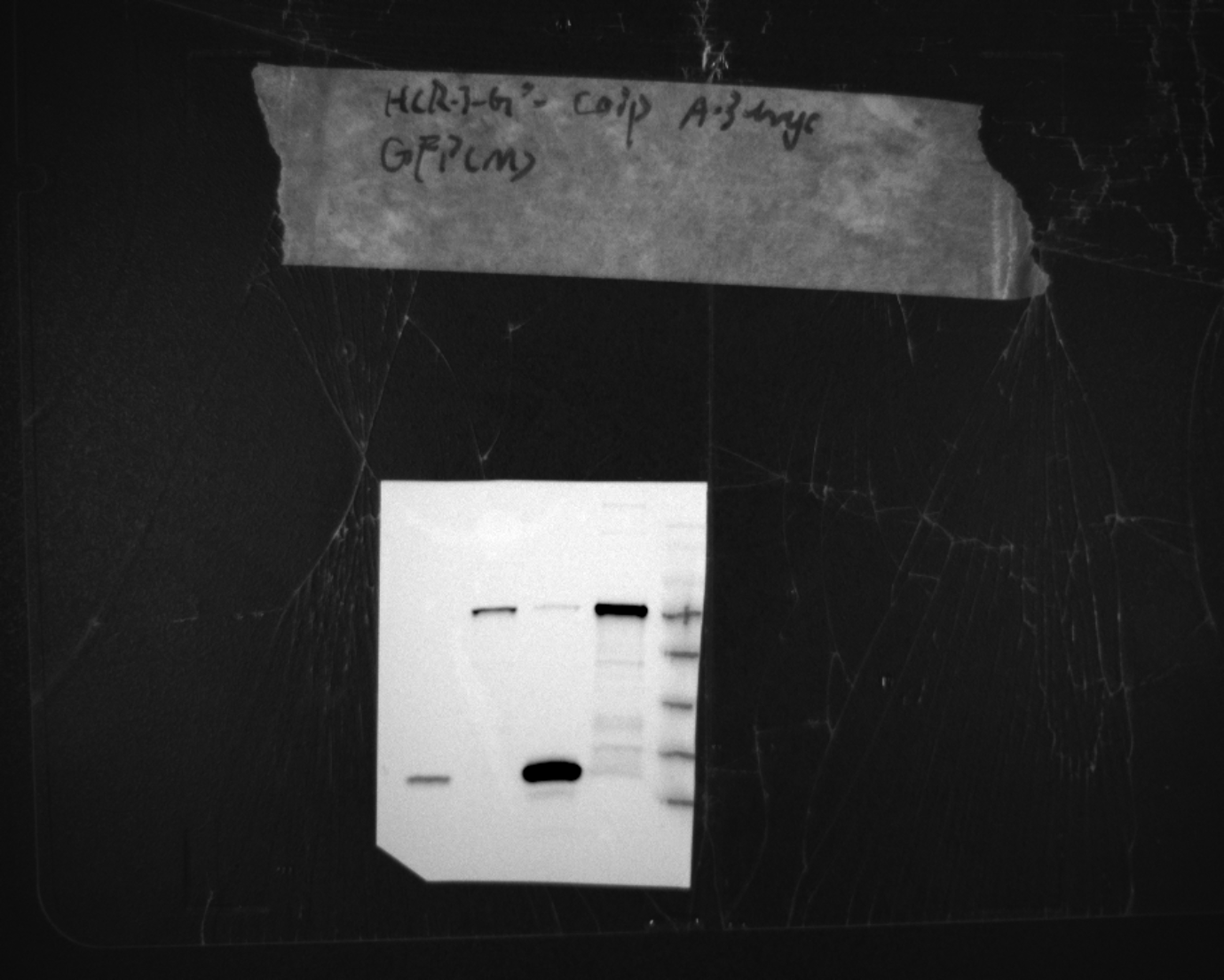

Supplement: Supplementary file 10 — Additional file 10. All Original and uncropped blots images used in manuscript. [file 12915_2022_1437_MOESM10_ESM.zip › blots images/Fig 1/Fig 1 F/HCR-CC3-GFP-coIP-astrin-CC2-myc-anti-GFP-marker.tif]

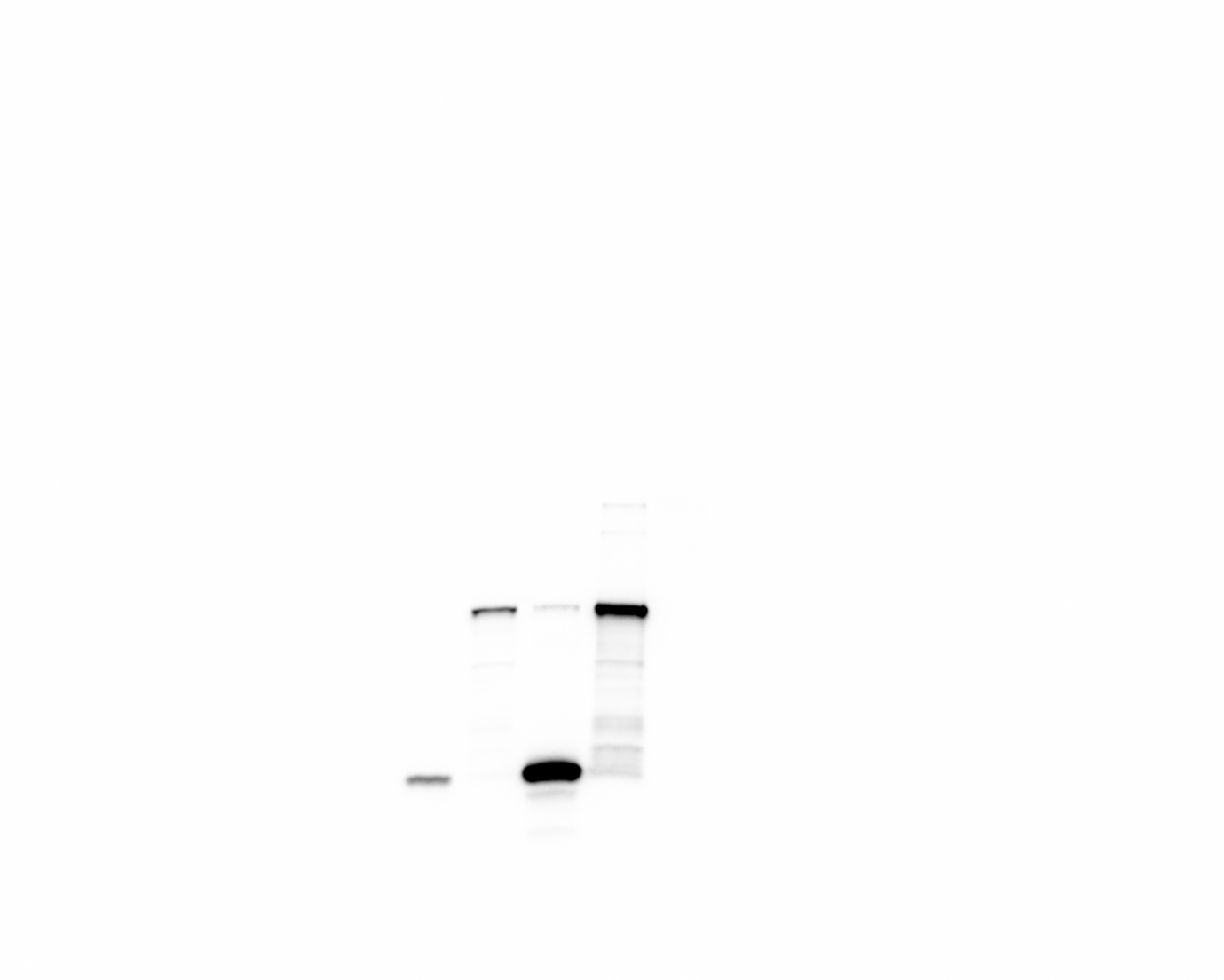

Supplement: Supplementary file 10 — Additional file 10. All Original and uncropped blots images used in manuscript. [file 12915_2022_1437_MOESM10_ESM.zip › blots images/Fig 1/Fig 1 F/HCR-CC3-GFP-coIP-astrin-CC2-myc-anti-GFP.tif]

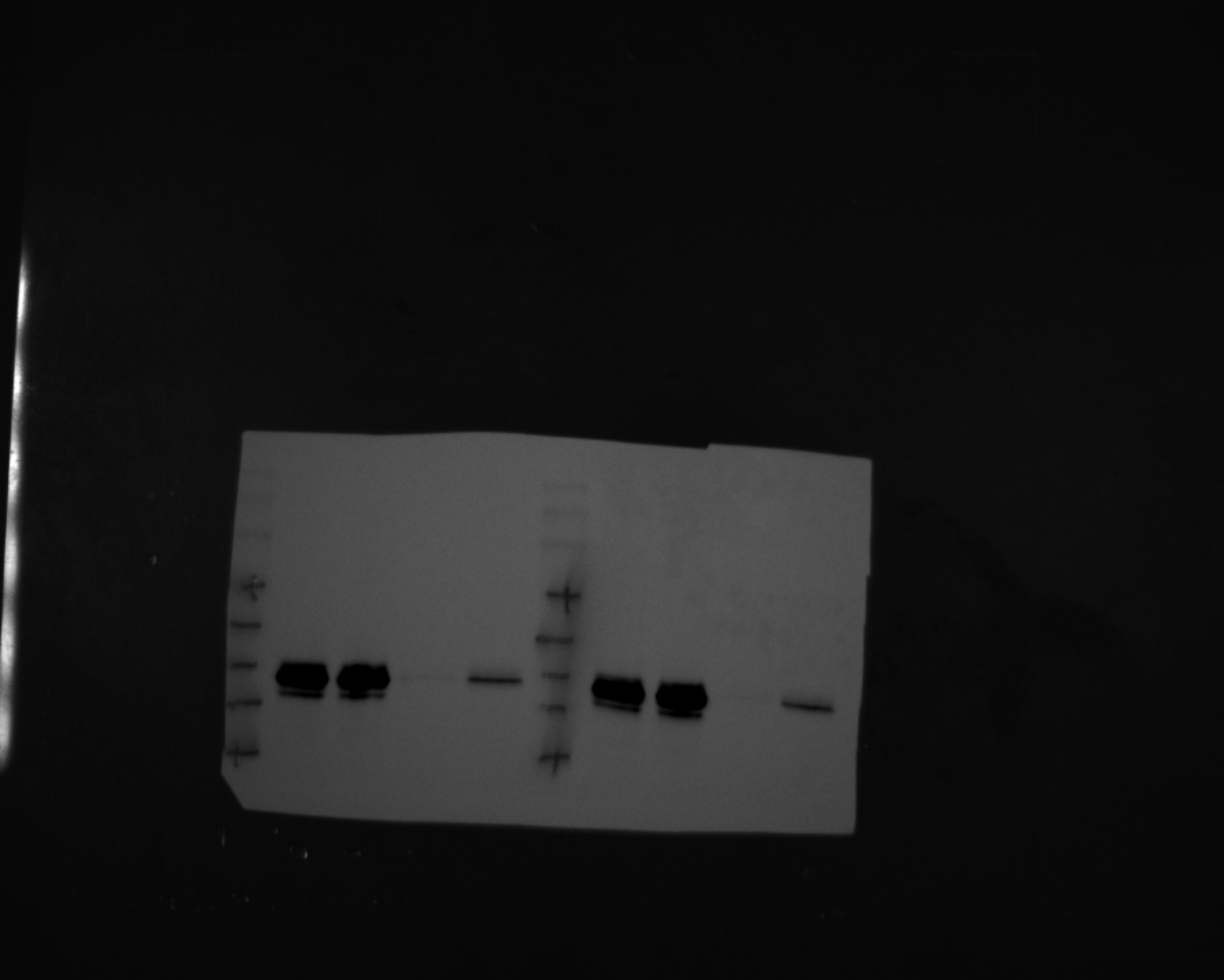

Supplement: Supplementary file 10 — Additional file 10. All Original and uncropped blots images used in manuscript. [file 12915_2022_1437_MOESM10_ESM.zip › blots images/Fig 1/Fig 1 F/left-3-lanes-HCR-CC3-GFP-coIP-astrin-CC2-myc-anti-myc-marker.tif]

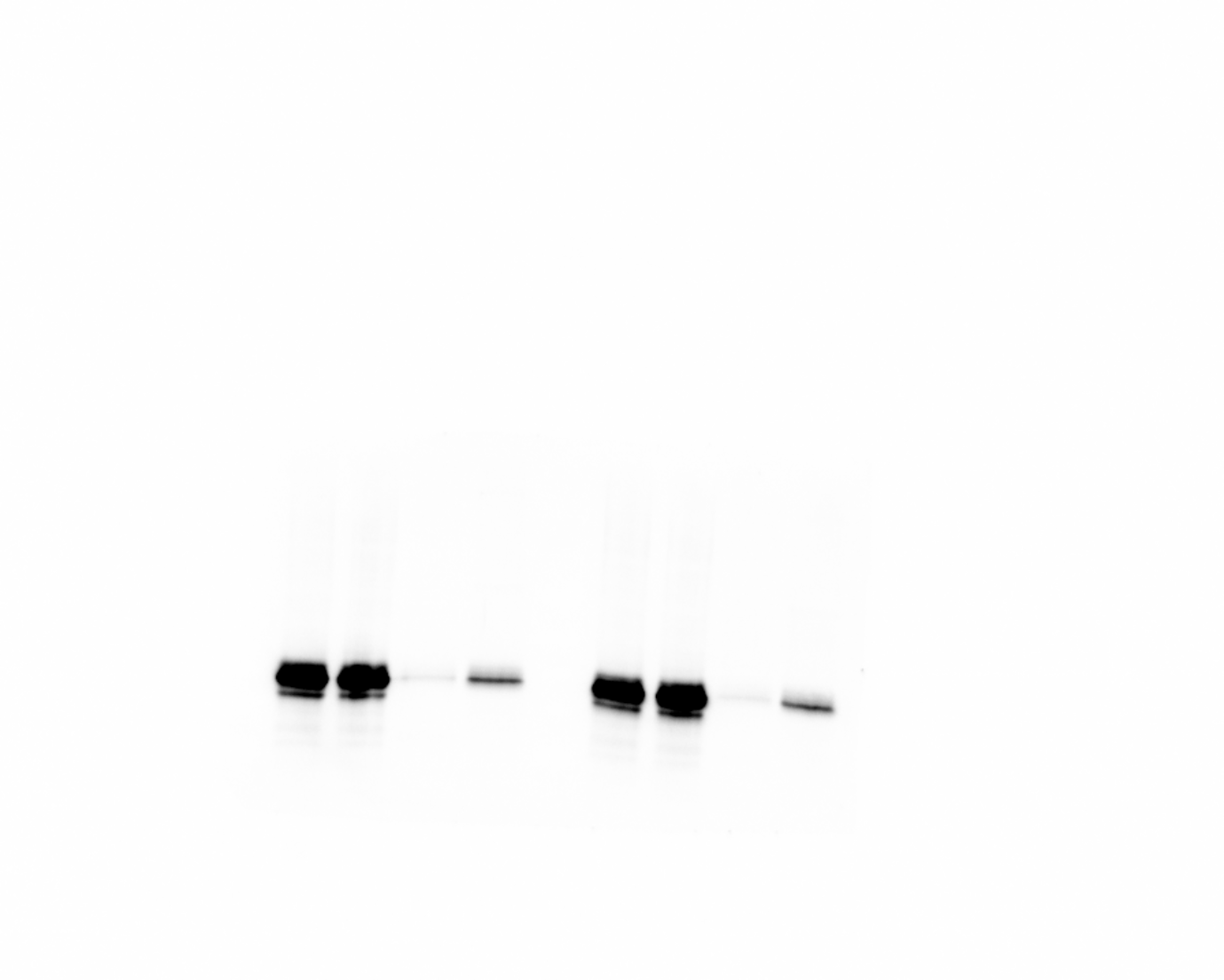

Supplement: Supplementary file 10 — Additional file 10. All Original and uncropped blots images used in manuscript. [file 12915_2022_1437_MOESM10_ESM.zip › blots images/Fig 1/Fig 1 F/left-3-lanes-HCR-CC3-GFP-coIP-astrin-CC2-myc-anti-myc.tif]

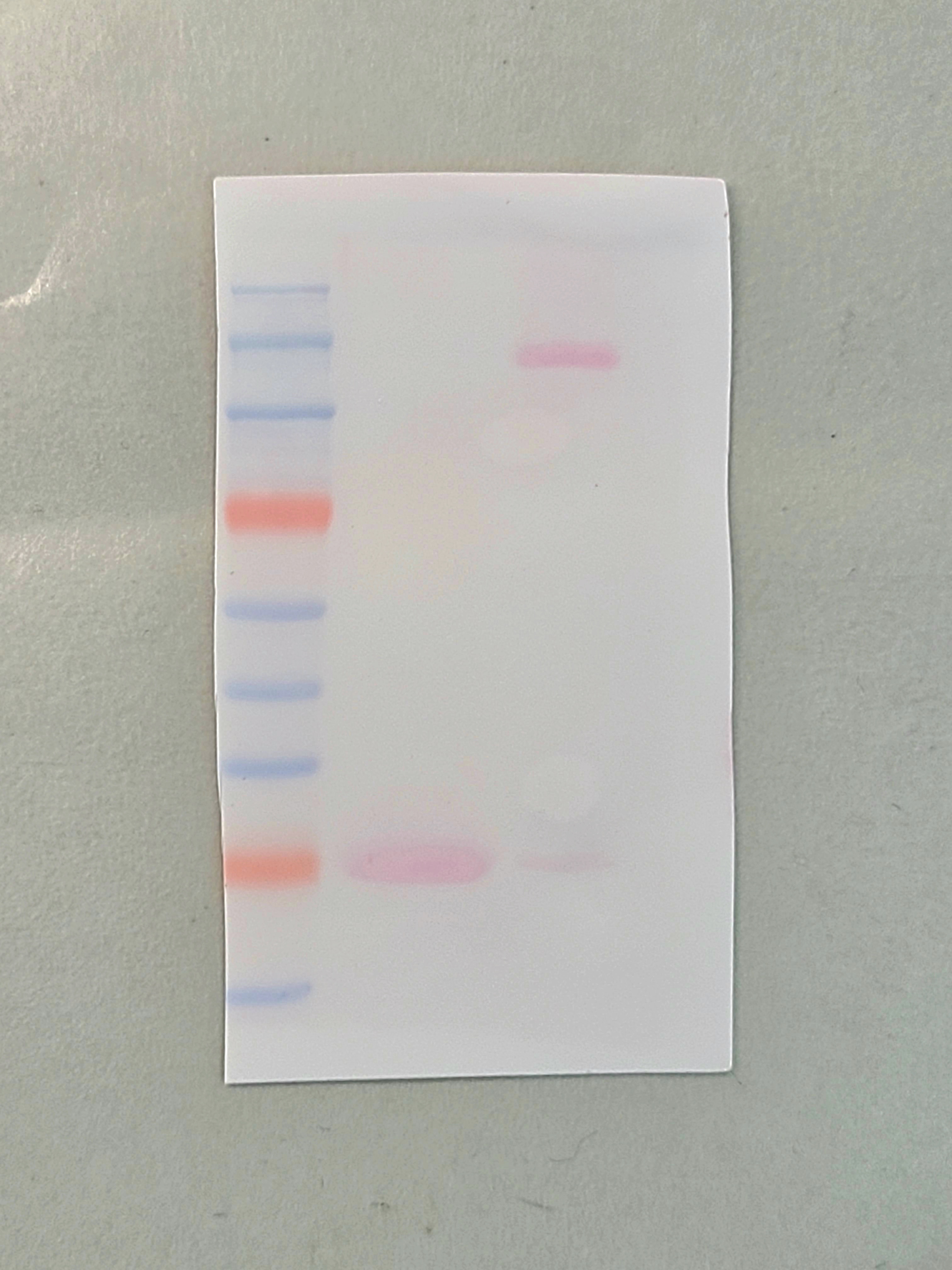

Supplement: Supplementary file 10 — Additional file 10. All Original and uncropped blots images used in manuscript. [file 12915_2022_1437_MOESM10_ESM.zip › blots images/Fig 1/Fig 1 G/GST-and-GST-astrin.jpg]

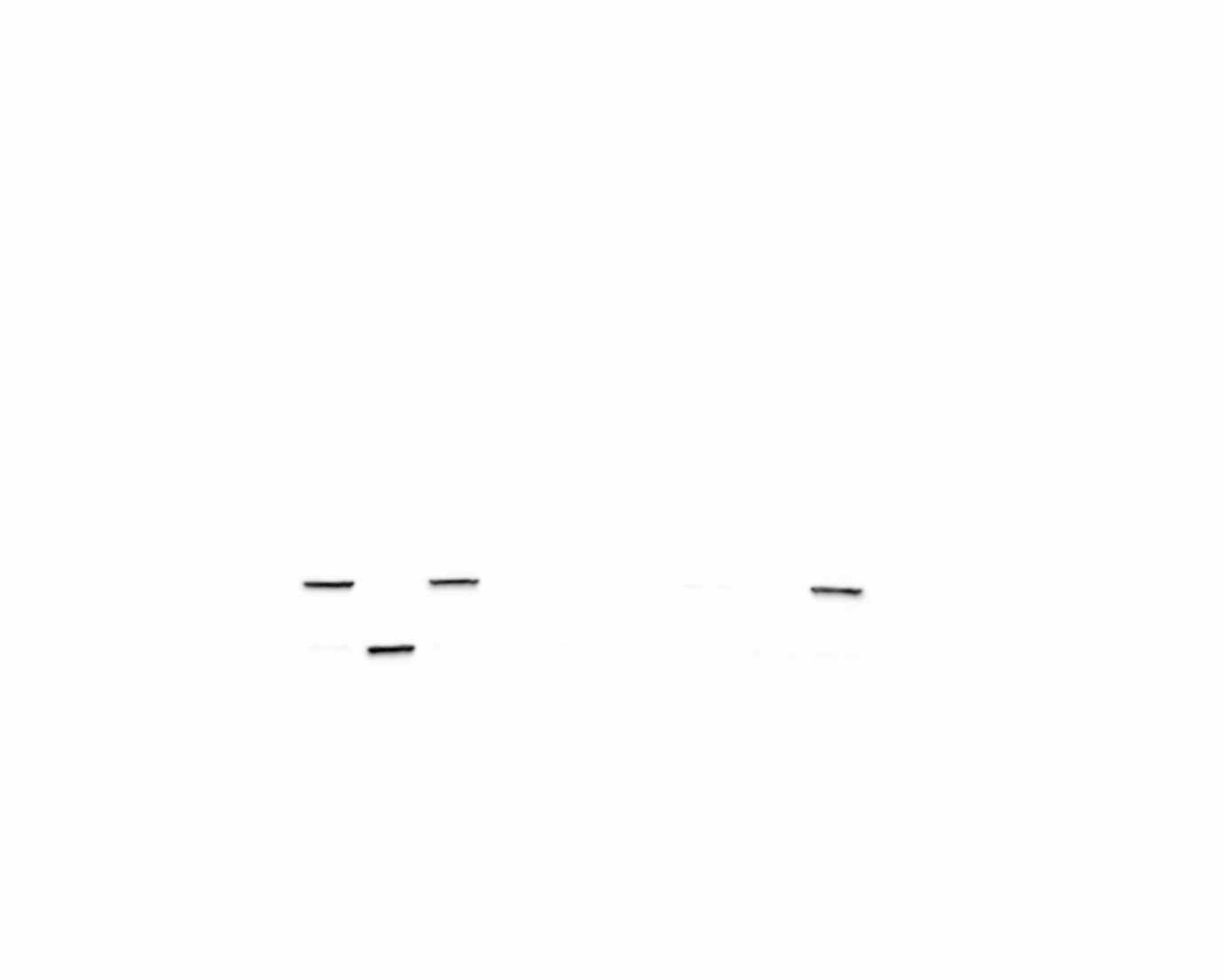

Supplement: Supplementary file 10 — Additional file 10. All Original and uncropped blots images used in manuscript. [file 12915_2022_1437_MOESM10_ESM.zip › blots images/Fig 1/Fig 1 G/GST-astrin-pull-down-HCR-1-2-3-hi.tif]

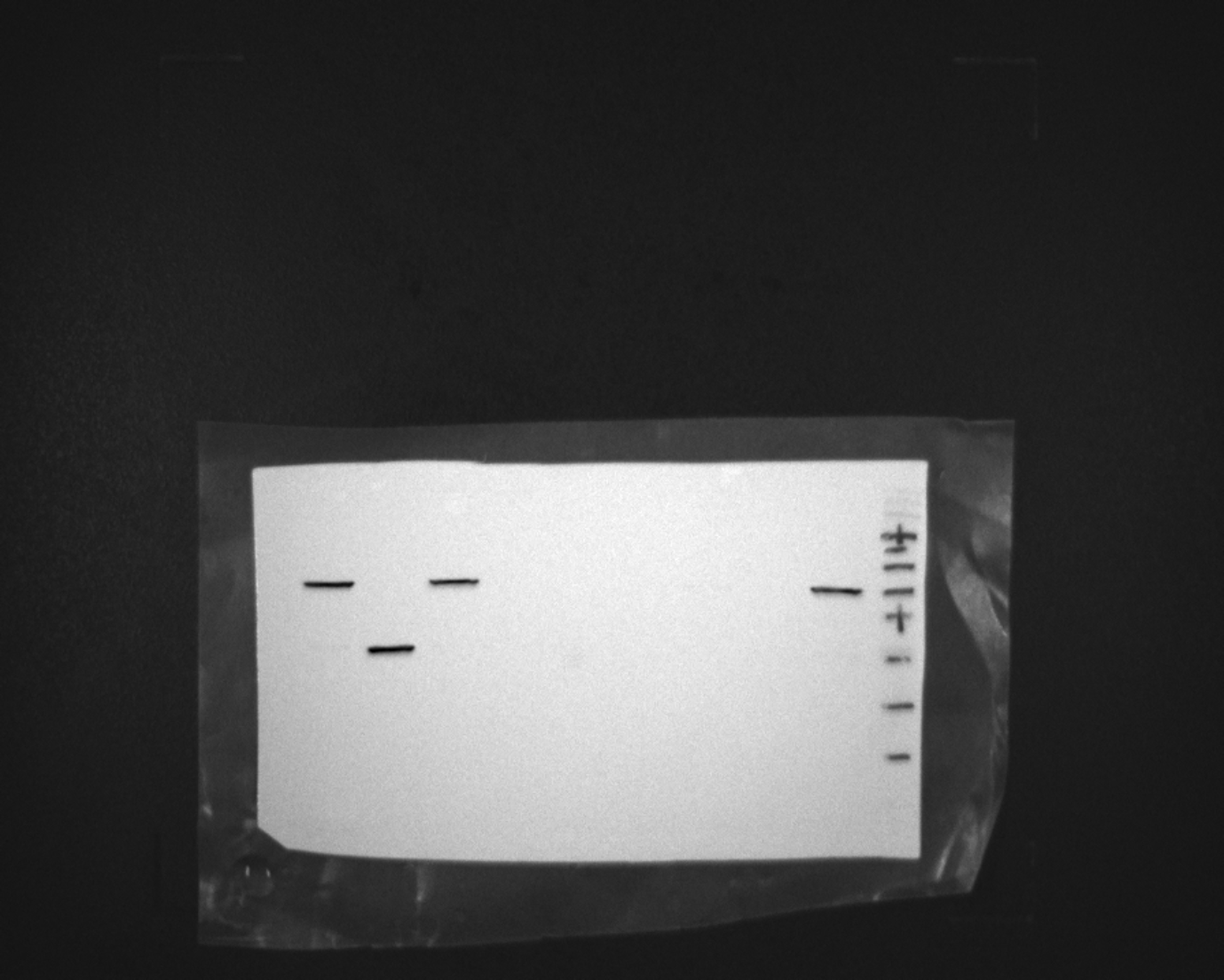

Supplement: Supplementary file 10 — Additional file 10. All Original and uncropped blots images used in manuscript. [file 12915_2022_1437_MOESM10_ESM.zip › blots images/Fig 1/Fig 1 G/GST-astrin-pull-down-HCR-1-2-3-his-marker.tif]

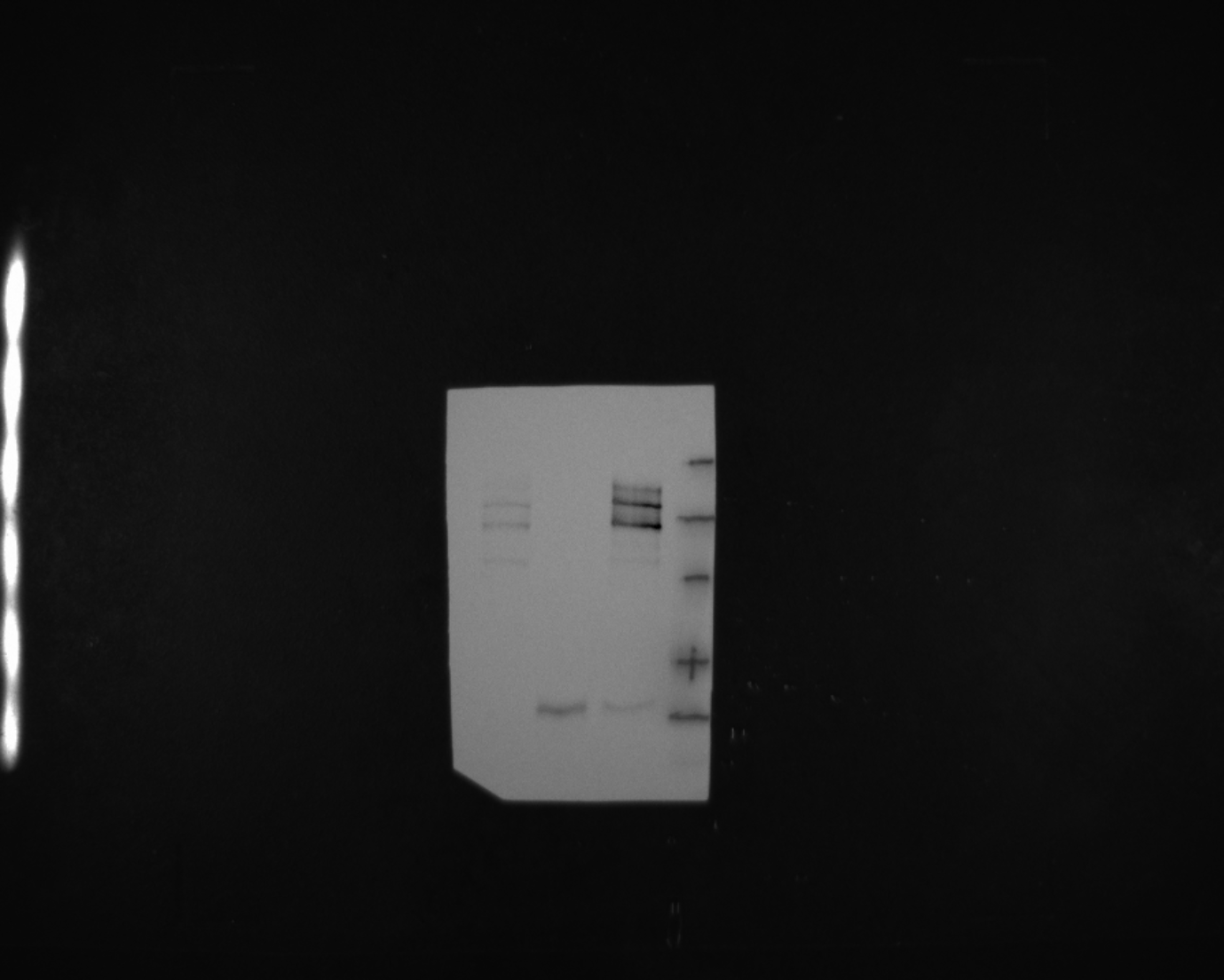

Supplement: Supplementary file 10 — Additional file 10. All Original and uncropped blots images used in manuscript. [file 12915_2022_1437_MOESM10_ESM.zip › blots images/Fig 2/Fig 2 C/IP-astrin/astrin-coIP-PCM1-anti-astrin-marker.tif]

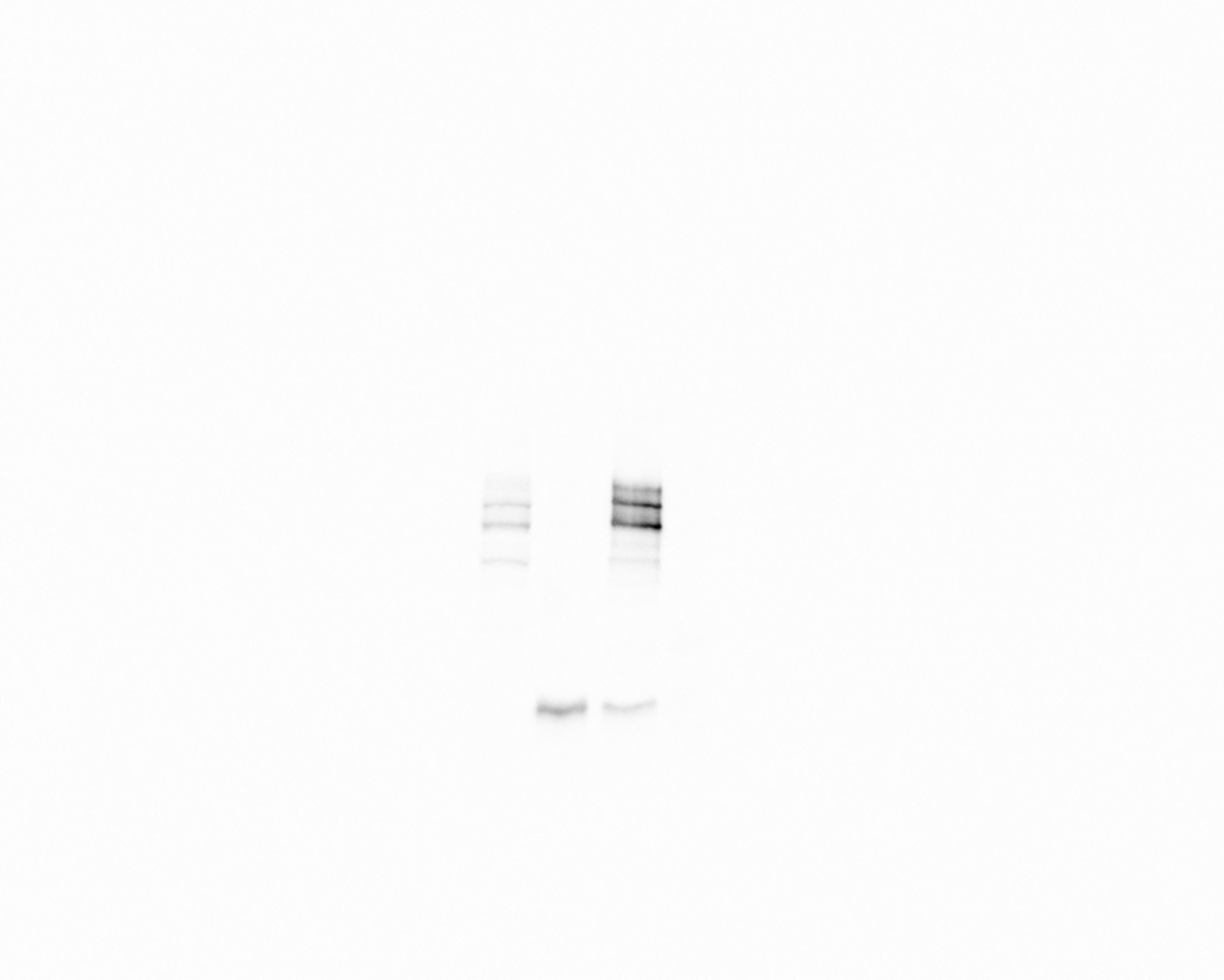

Supplement: Supplementary file 10 — Additional file 10. All Original and uncropped blots images used in manuscript. [file 12915_2022_1437_MOESM10_ESM.zip › blots images/Fig 2/Fig 2 C/IP-astrin/astrin-coIP-PCM1-anti-astrin.tif]

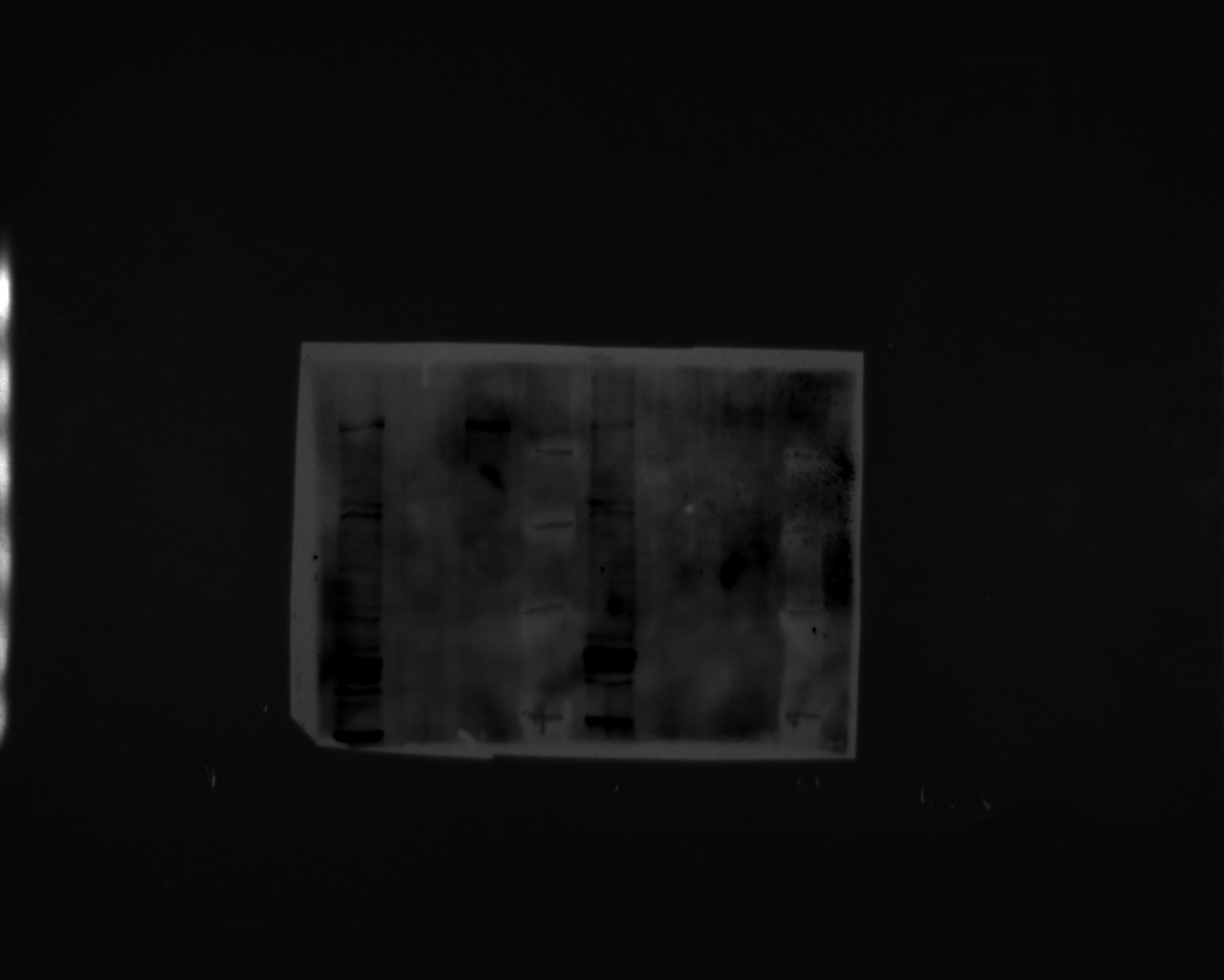

Supplement: Supplementary file 10 — Additional file 10. All Original and uncropped blots images used in manuscript. [file 12915_2022_1437_MOESM10_ESM.zip › blots images/Fig 2/Fig 2 C/IP-astrin/left-three-lanes-astrin-coIP-PCM1-anti-PCM1-marker.tif]

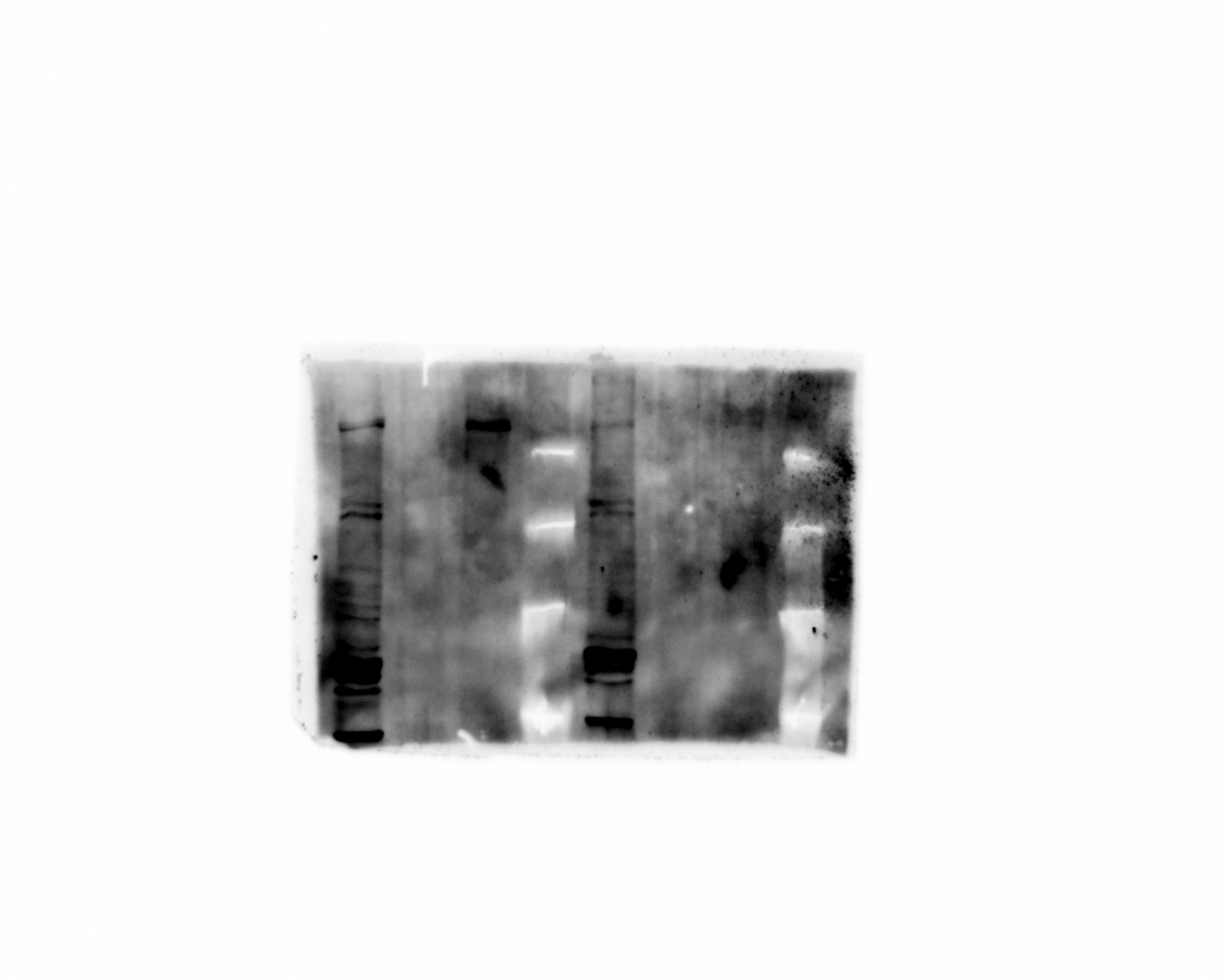

Supplement: Supplementary file 10 — Additional file 10. All Original and uncropped blots images used in manuscript. [file 12915_2022_1437_MOESM10_ESM.zip › blots images/Fig 2/Fig 2 C/IP-astrin/left-three-lanes-astrin-coIP-PCM1-anti-PCM1.tif]

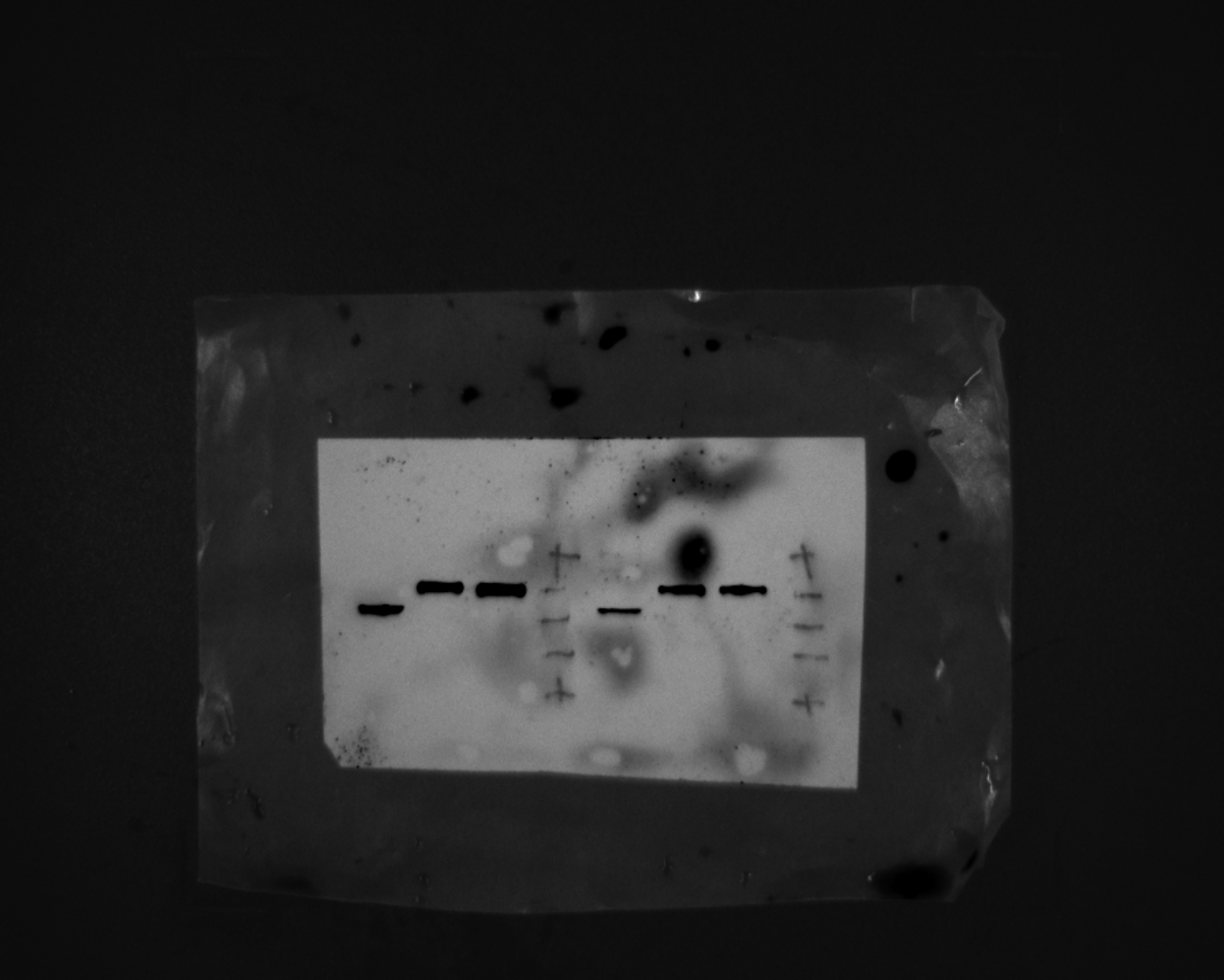

Supplement: Supplementary file 10 — Additional file 10. All Original and uncropped blots images used in manuscript. [file 12915_2022_1437_MOESM10_ESM.zip › blots images/Fig 2/Fig 2 C/IP-astrin/right-three-lanes-IP-astrin-beta-actin-marker.tif]

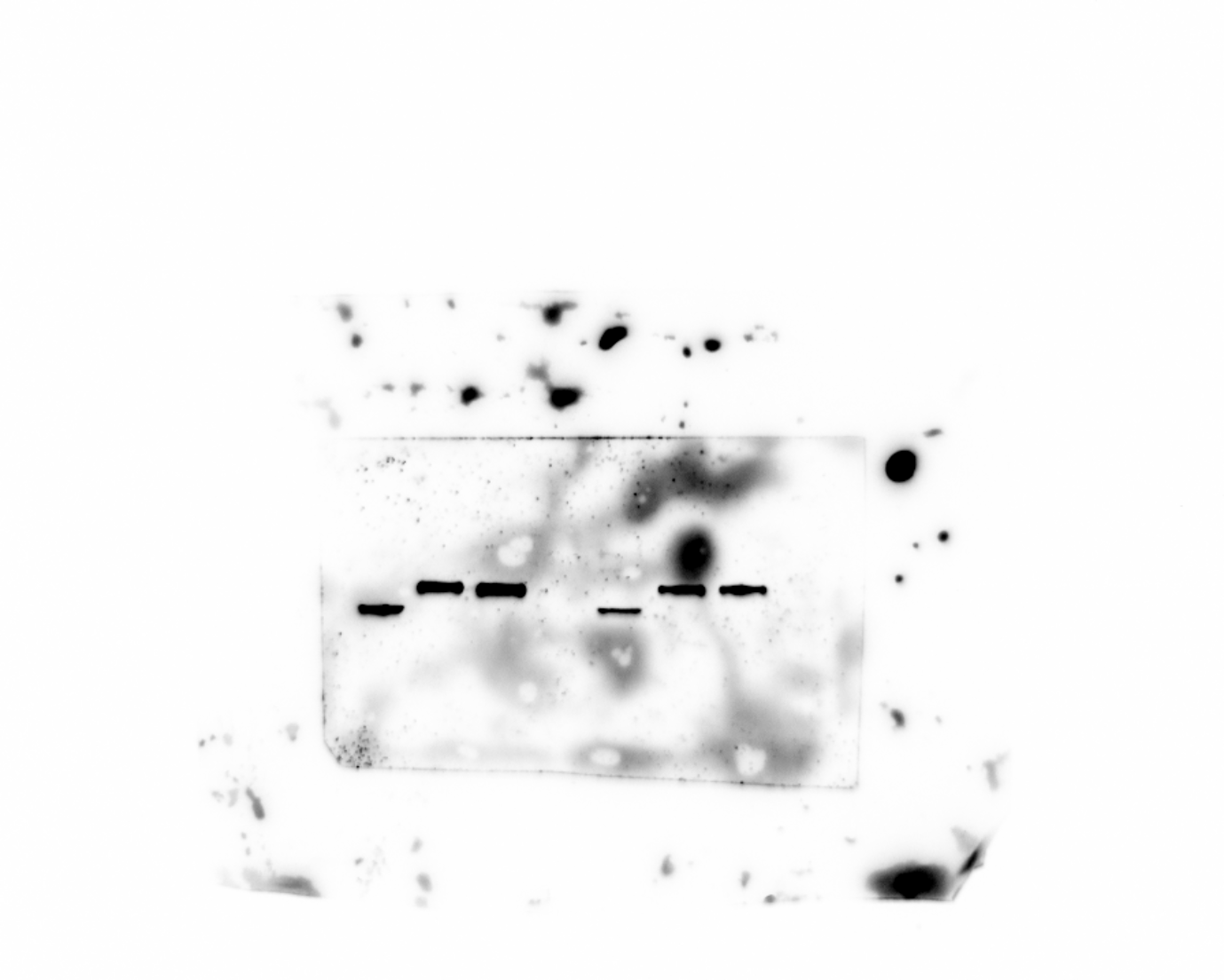

Supplement: Supplementary file 10 — Additional file 10. All Original and uncropped blots images used in manuscript. [file 12915_2022_1437_MOESM10_ESM.zip › blots images/Fig 2/Fig 2 C/IP-astrin/right-three-lanes-IP-astrin-beta-actin.tif]

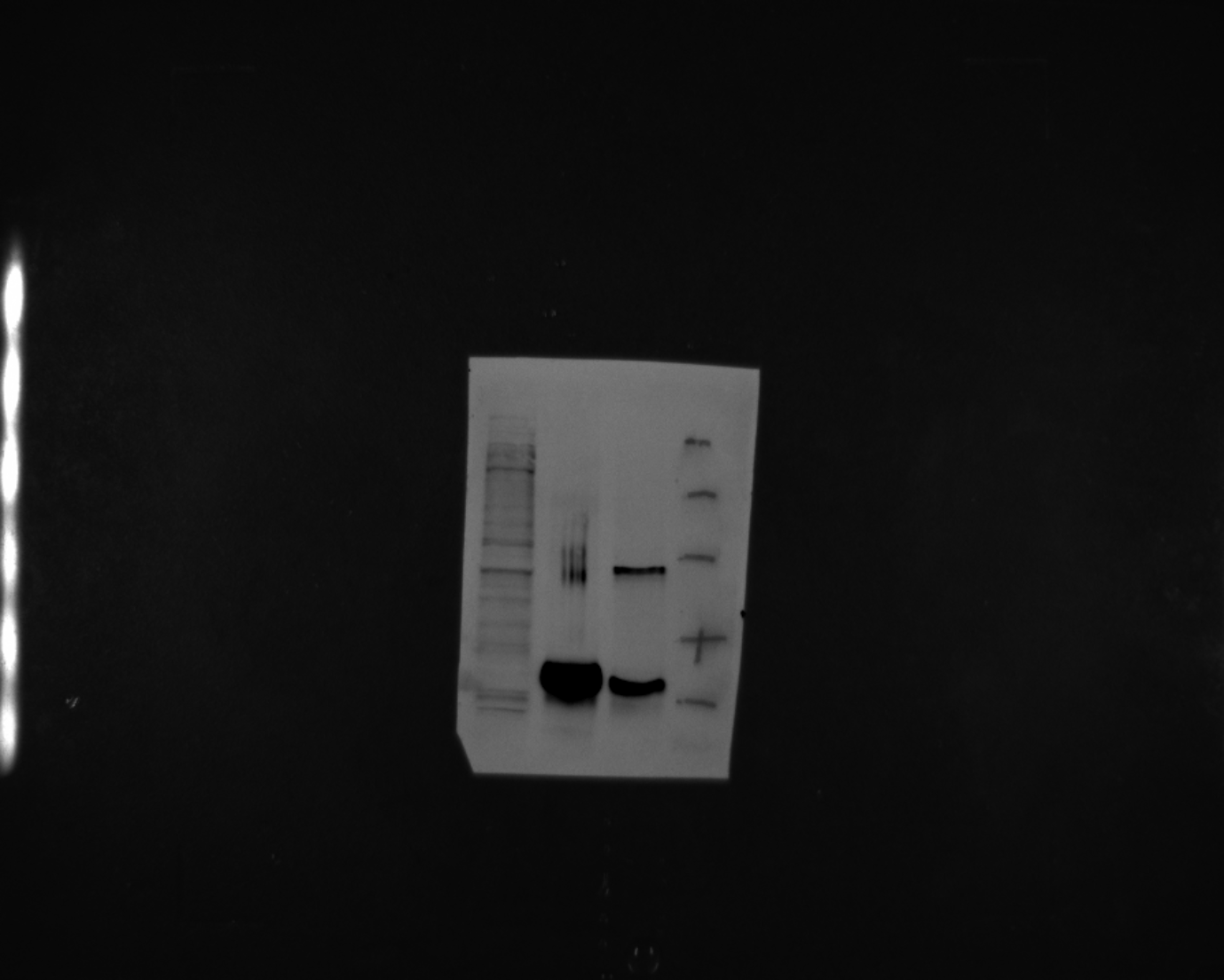

Supplement: Supplementary file 10 — Additional file 10. All Original and uncropped blots images used in manuscript. [file 12915_2022_1437_MOESM10_ESM.zip › blots images/Fig 2/Fig 2 C/IP-HCR/HCR-coIP-PCM1-anti-HCR-marker.tif]

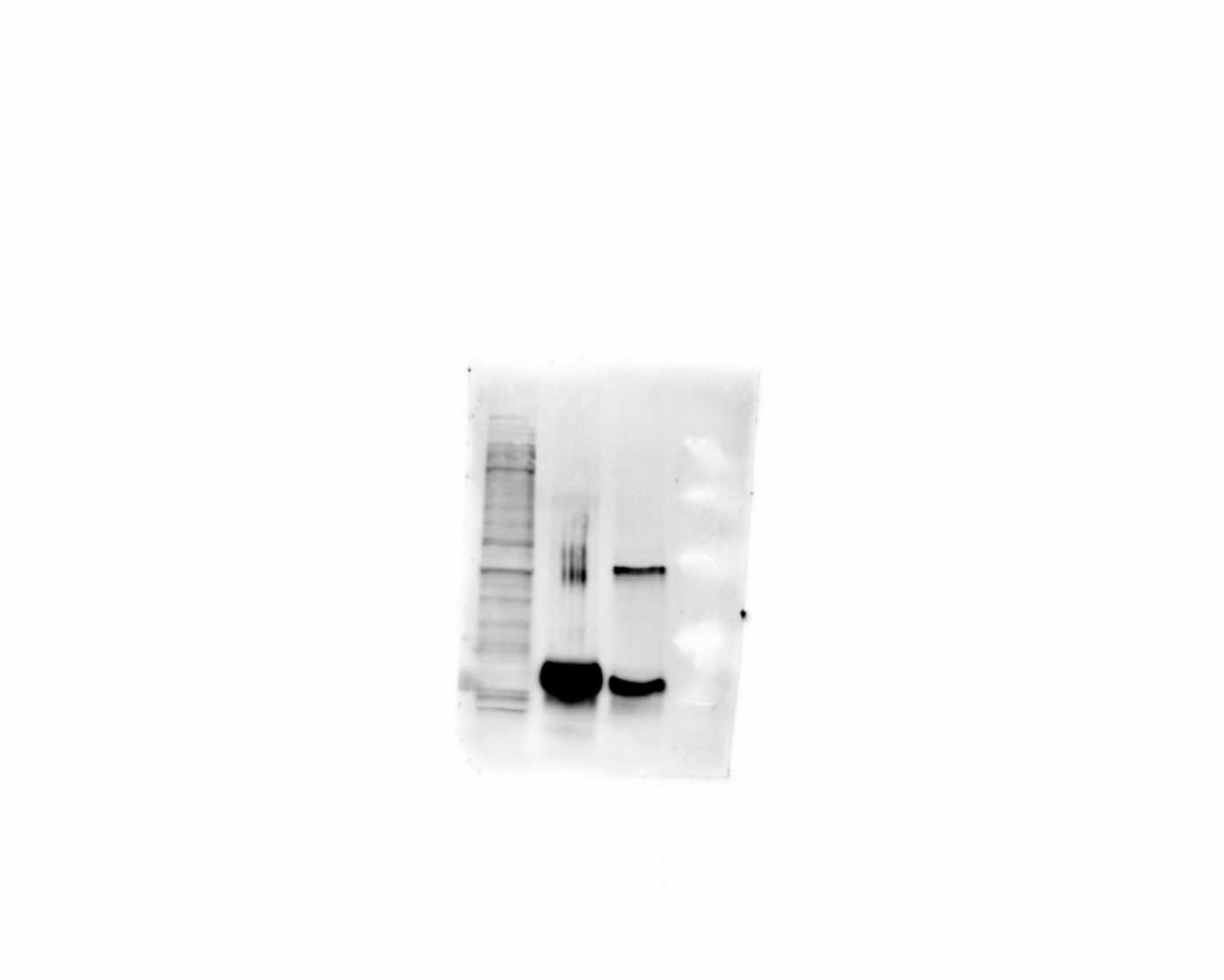

Supplement: Supplementary file 10 — Additional file 10. All Original and uncropped blots images used in manuscript. [file 12915_2022_1437_MOESM10_ESM.zip › blots images/Fig 2/Fig 2 C/IP-HCR/HCR-coIP-PCM1-anti-HCR.tif]

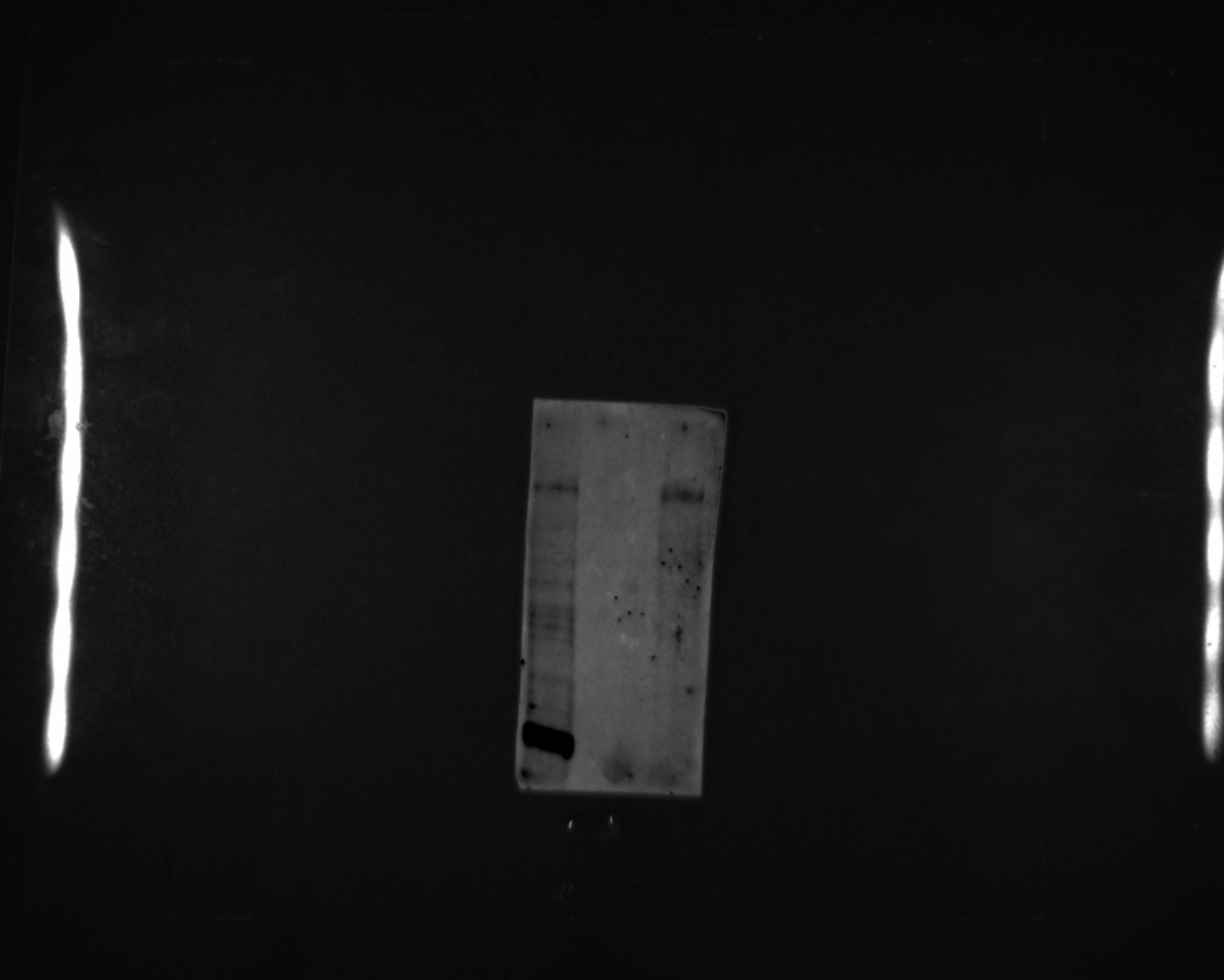

Supplement: Supplementary file 10 — Additional file 10. All Original and uncropped blots images used in manuscript. [file 12915_2022_1437_MOESM10_ESM.zip › blots images/Fig 2/Fig 2 C/IP-HCR/HCR-coIP-PCM1-anti-PCM1-marker.tif]

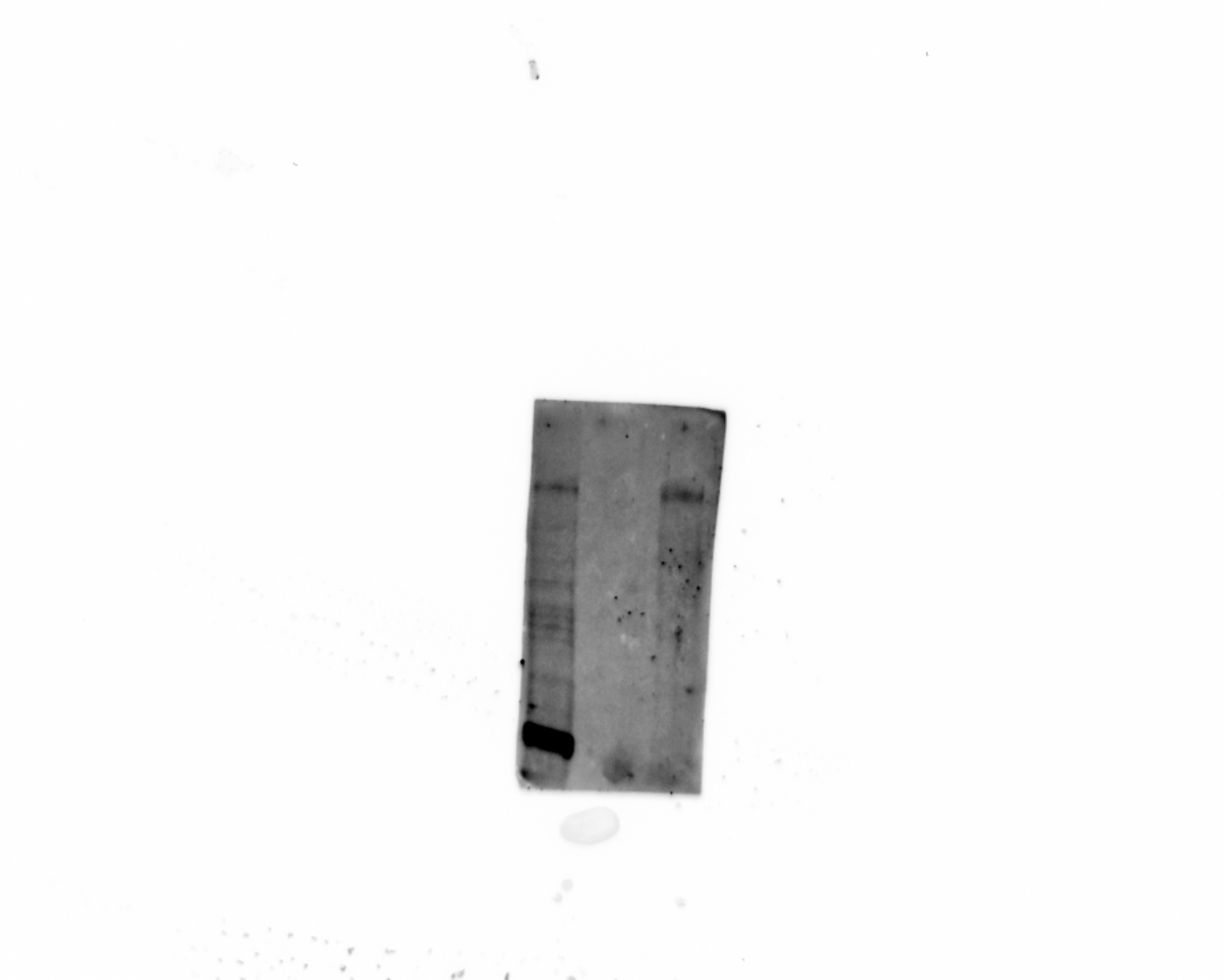

Supplement: Supplementary file 10 — Additional file 10. All Original and uncropped blots images used in manuscript. [file 12915_2022_1437_MOESM10_ESM.zip › blots images/Fig 2/Fig 2 C/IP-HCR/HCR-coIP-PCM1-anti-PCM1.tif]

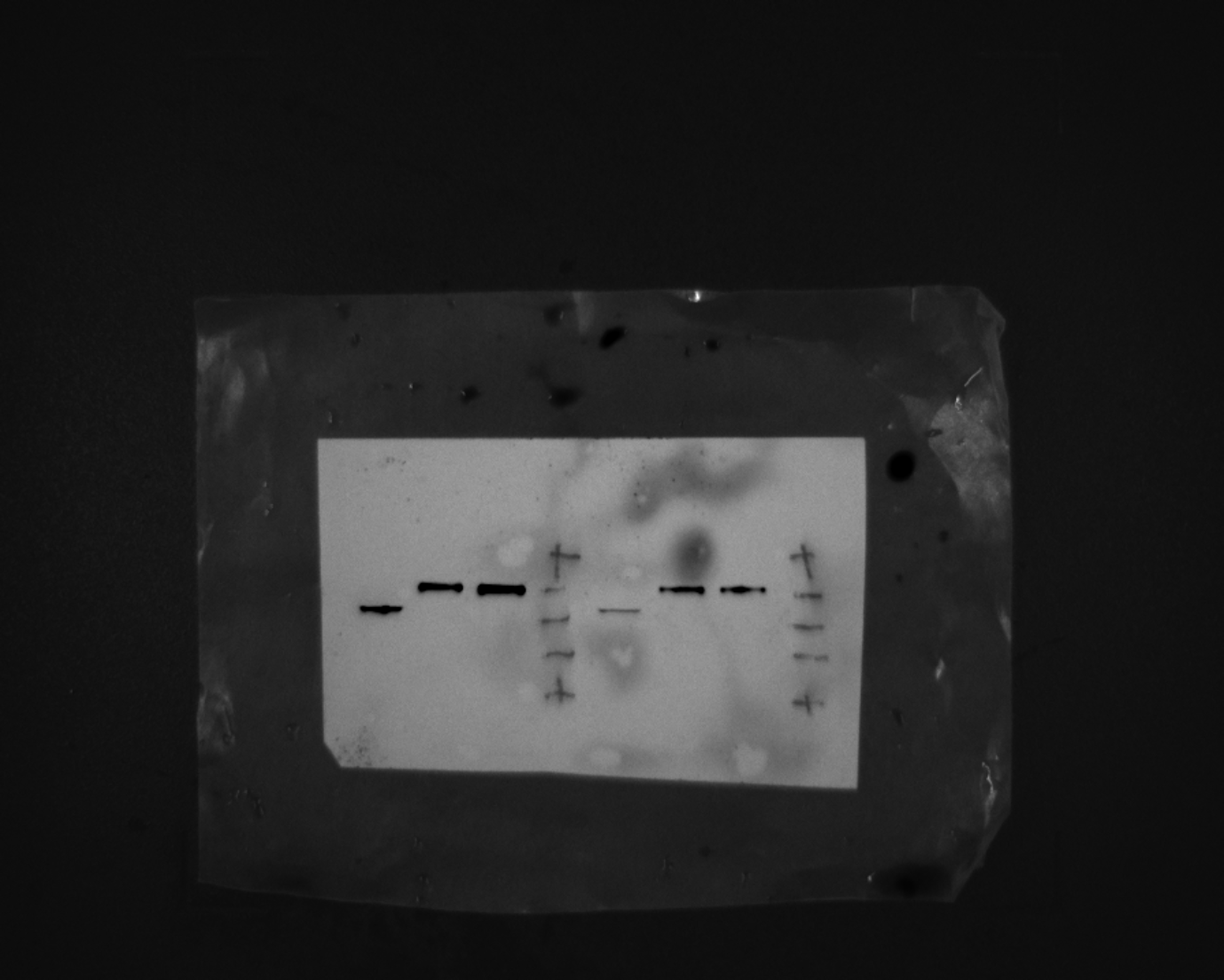

Supplement: Supplementary file 10 — Additional file 10. All Original and uncropped blots images used in manuscript. [file 12915_2022_1437_MOESM10_ESM.zip › blots images/Fig 2/Fig 2 C/IP-HCR/left-three-lanes-IP-HCR-beta-actin-marker.tif]

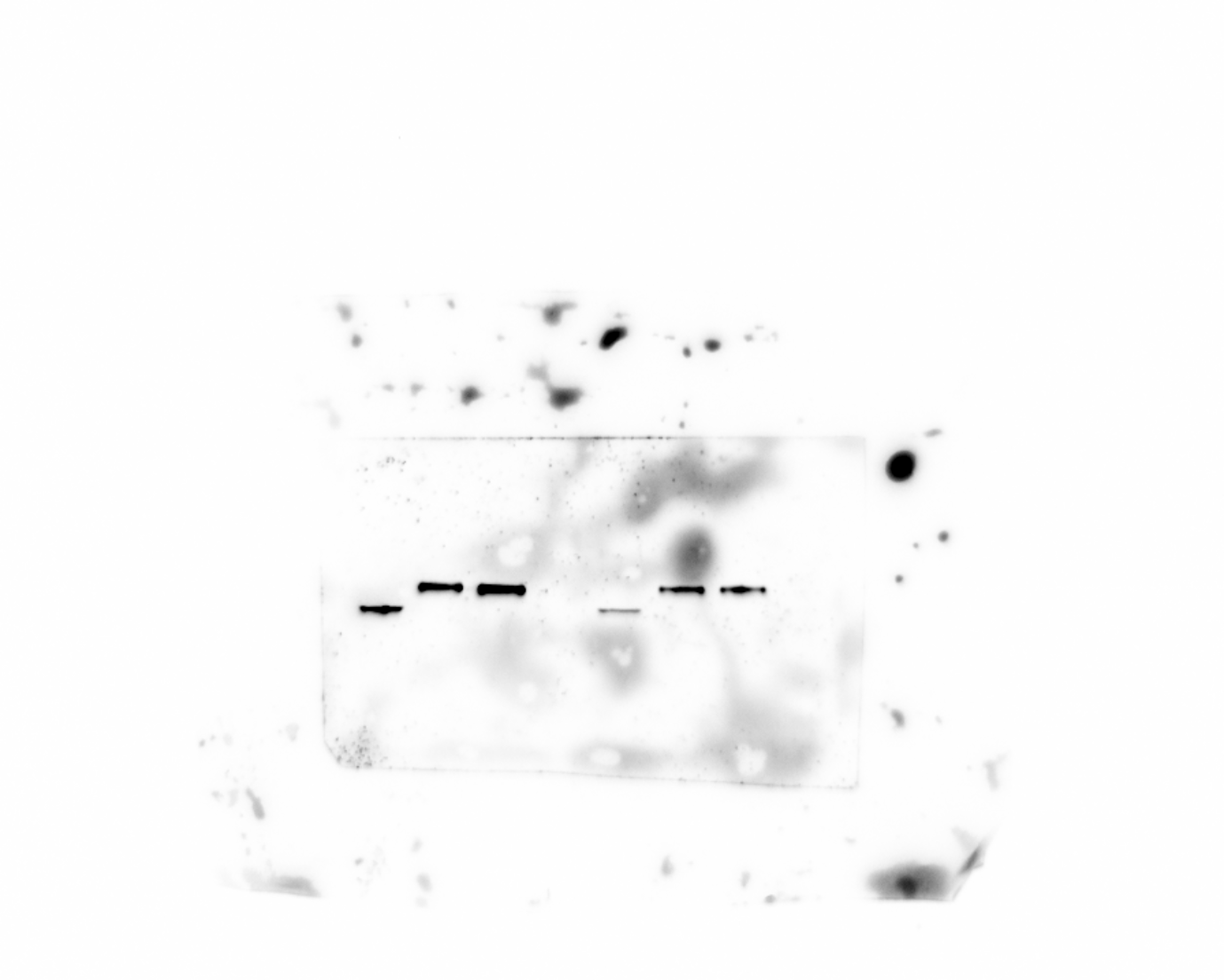

Supplement: Supplementary file 10 — Additional file 10. All Original and uncropped blots images used in manuscript. [file 12915_2022_1437_MOESM10_ESM.zip › blots images/Fig 2/Fig 2 C/IP-HCR/left-three-lanes-IP-HCR-beta-actin.tif]

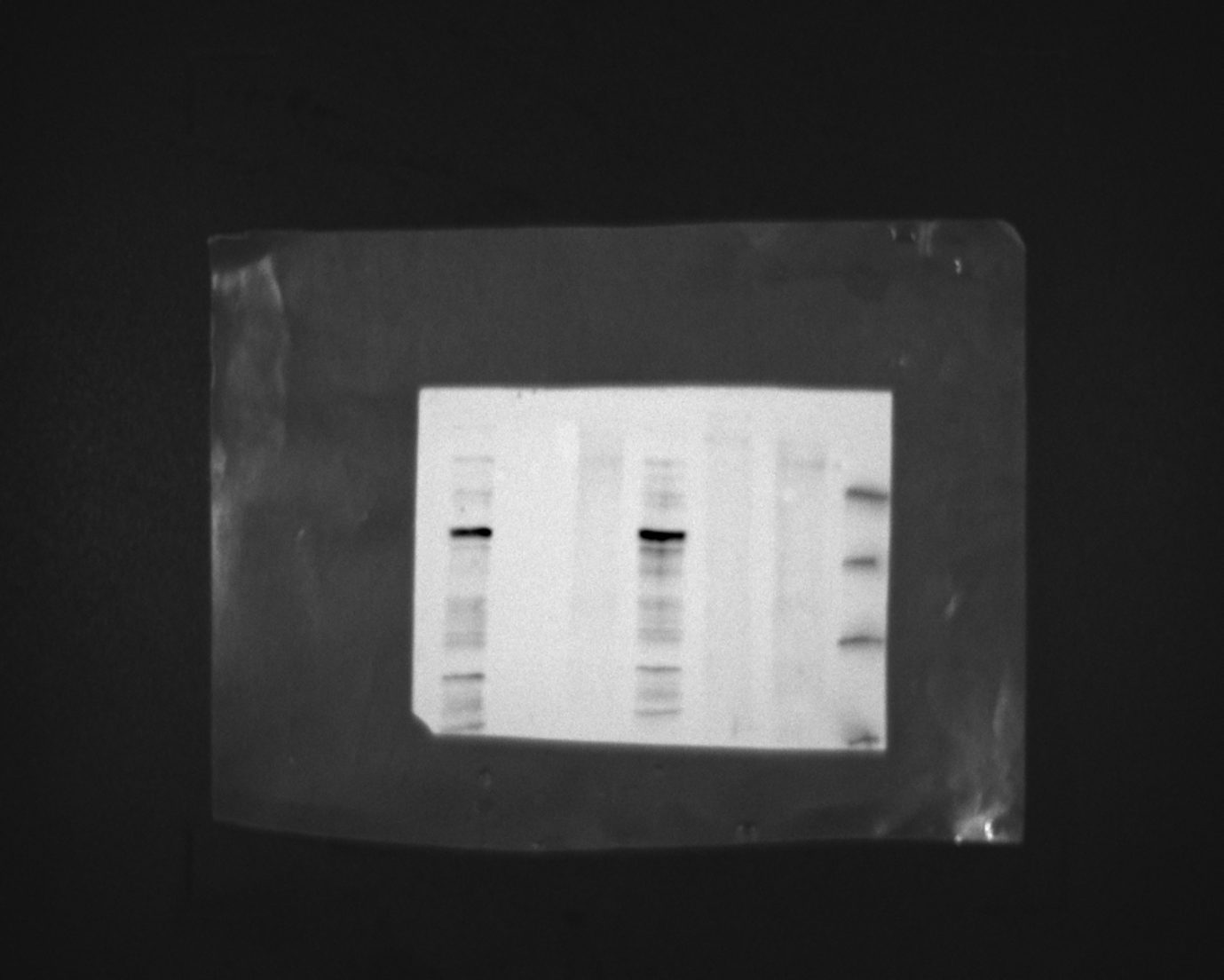

Supplement: Supplementary file 10 — Additional file 10. All Original and uncropped blots images used in manuscript. [file 12915_2022_1437_MOESM10_ESM.zip › blots images/Fig 2/Fig 2 C/left-three-lanes-IP-HCR-right-three-lanes-IP-astrin-anti-GM130-marker.tif]

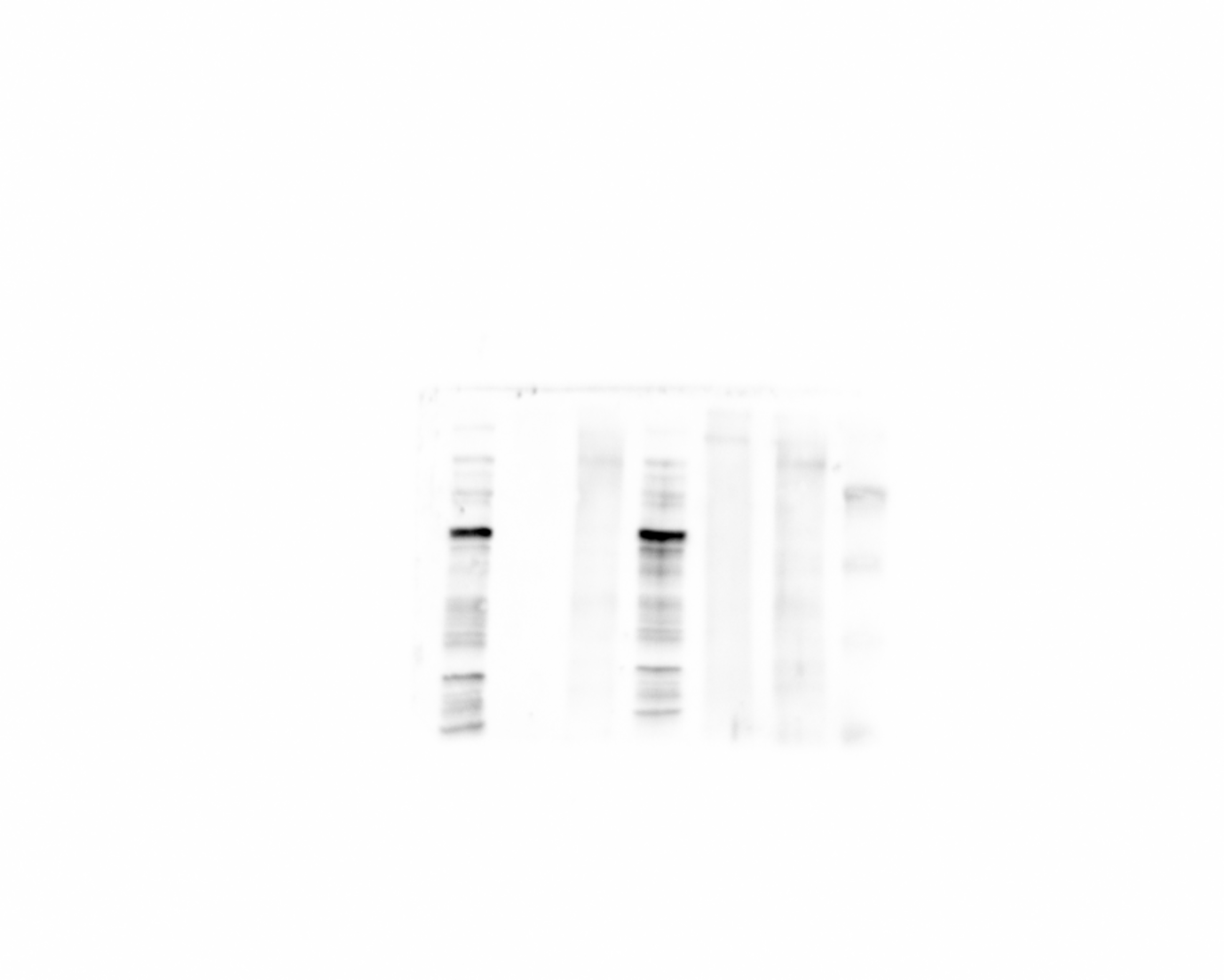

Supplement: Supplementary file 10 — Additional file 10. All Original and uncropped blots images used in manuscript. [file 12915_2022_1437_MOESM10_ESM.zip › blots images/Fig 2/Fig 2 C/left-three-lanes-IP-HCR-right-three-lanes-IP-astrin-anti-GM130.tif]

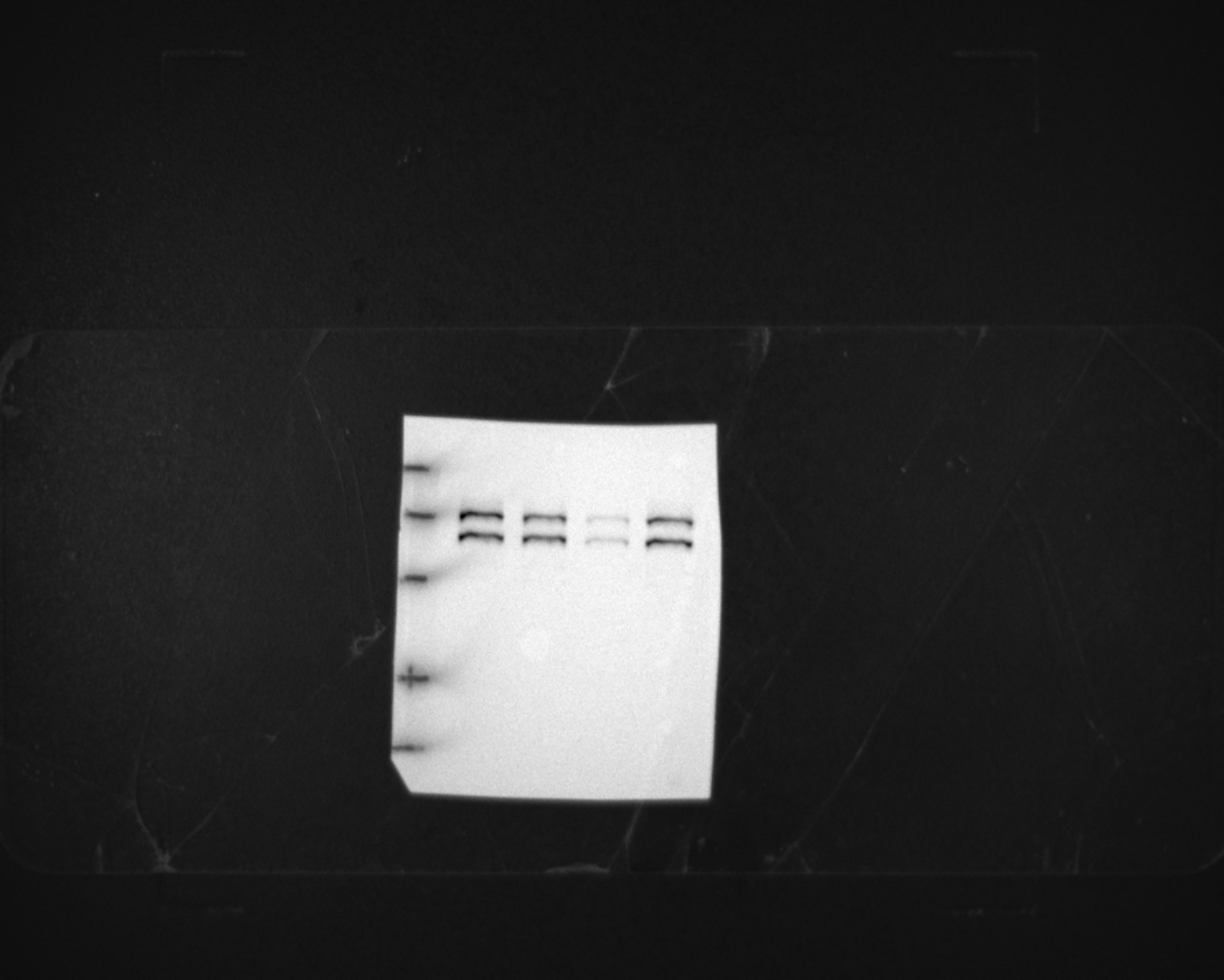

Supplement: Supplementary file 10 — Additional file 10. All Original and uncropped blots images used in manuscript. [file 12915_2022_1437_MOESM10_ESM.zip › blots images/Fig 3/Fig 3 A/Fig3A-anti-astrin-marker.tif]

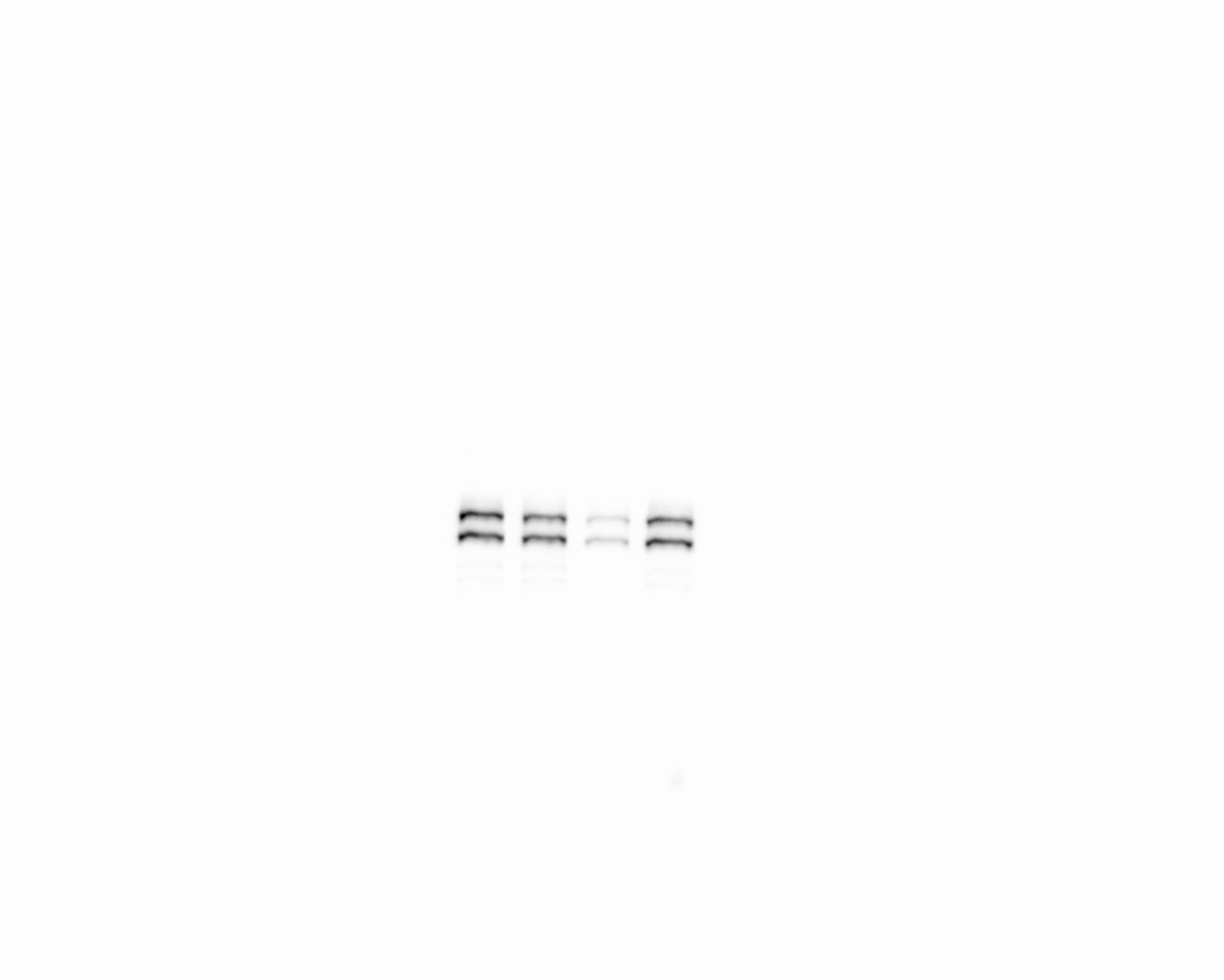

Supplement: Supplementary file 10 — Additional file 10. All Original and uncropped blots images used in manuscript. [file 12915_2022_1437_MOESM10_ESM.zip › blots images/Fig 3/Fig 3 A/Fig3A-anti-astrin.tif]

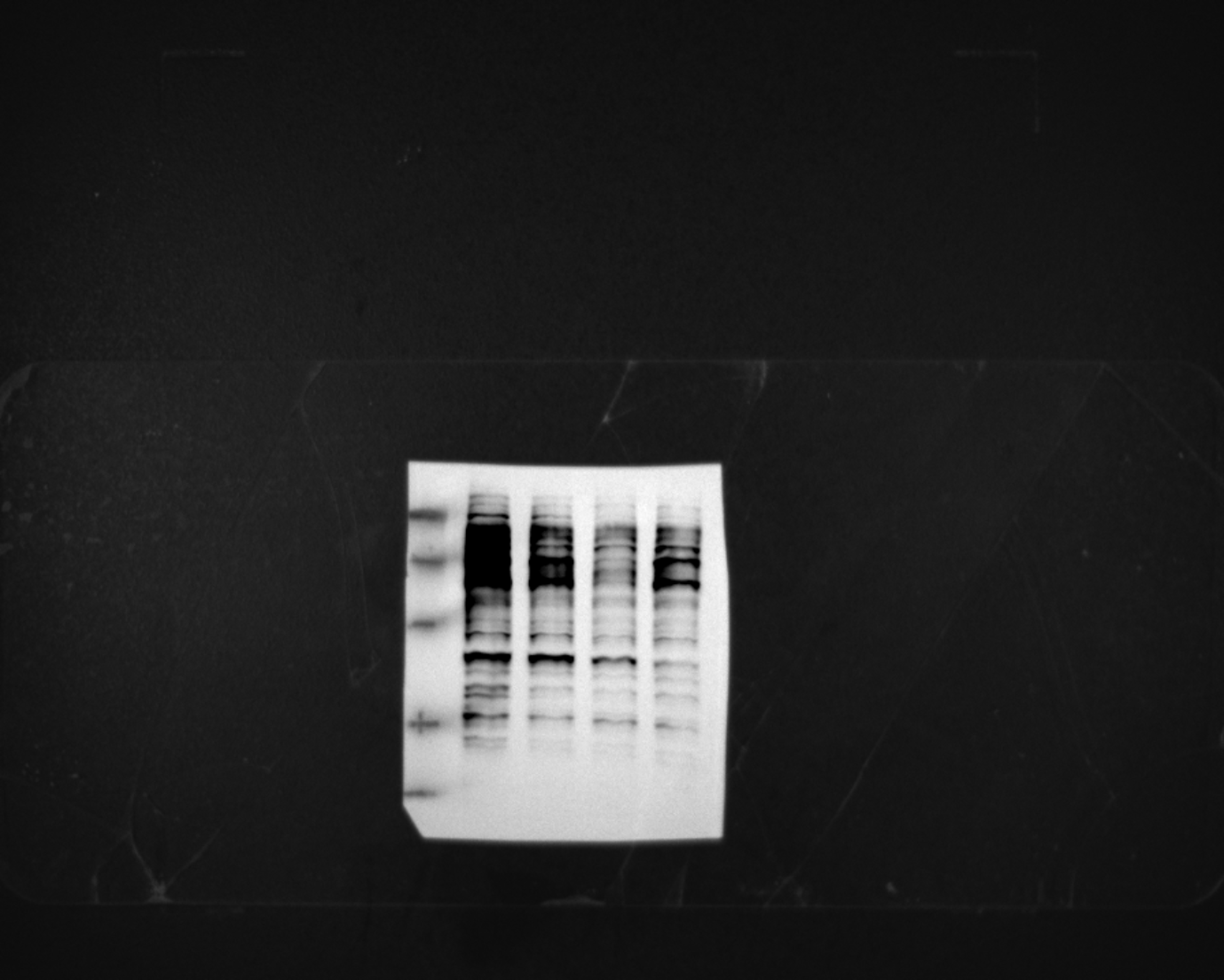

Supplement: Supplementary file 10 — Additional file 10. All Original and uncropped blots images used in manuscript. [file 12915_2022_1437_MOESM10_ESM.zip › blots images/Fig 3/Fig 3 A/Fig3A-anti-HCR-marker.tif]

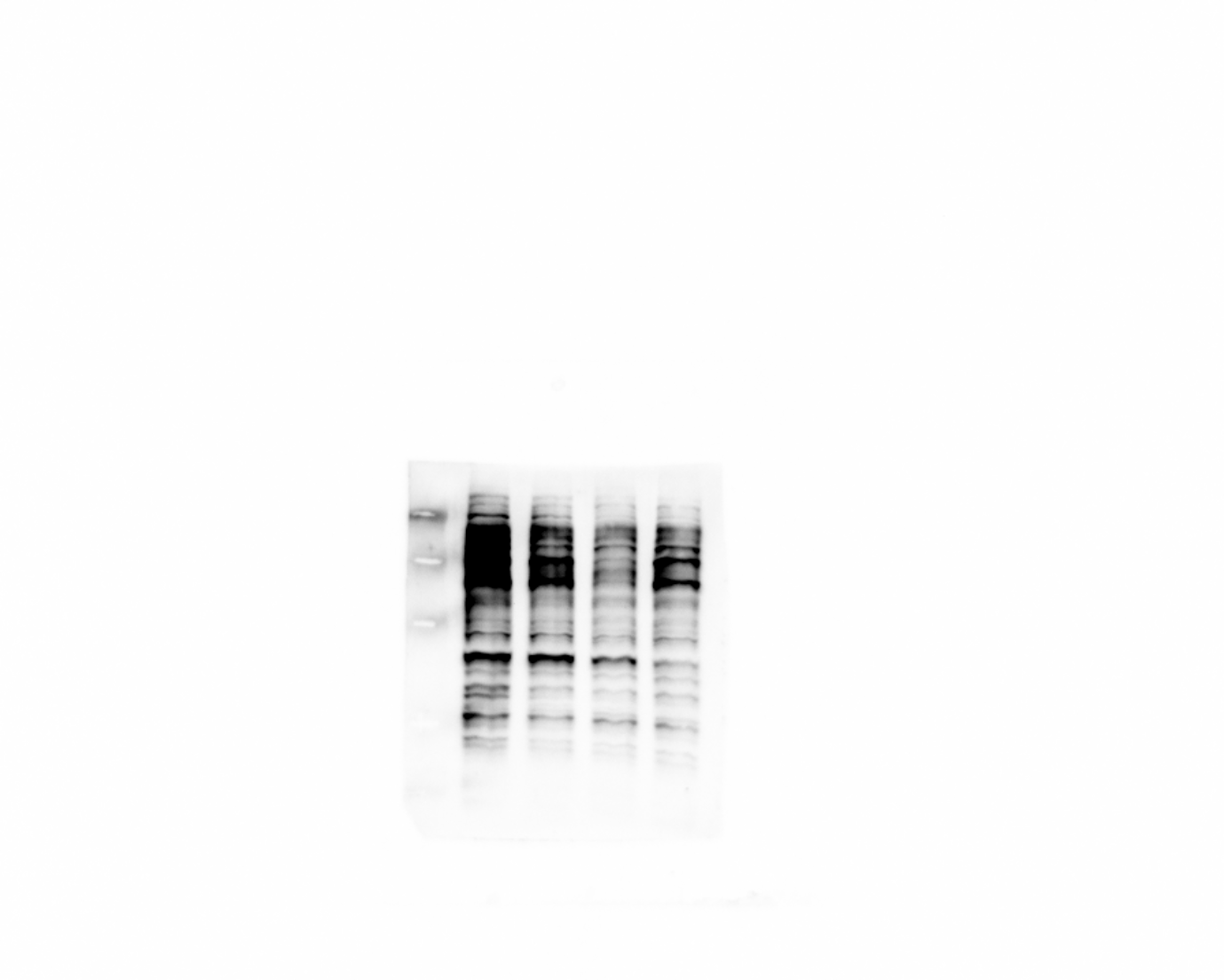

Supplement: Supplementary file 10 — Additional file 10. All Original and uncropped blots images used in manuscript. [file 12915_2022_1437_MOESM10_ESM.zip › blots images/Fig 3/Fig 3 A/Fig3A-anti-HCR.tif]

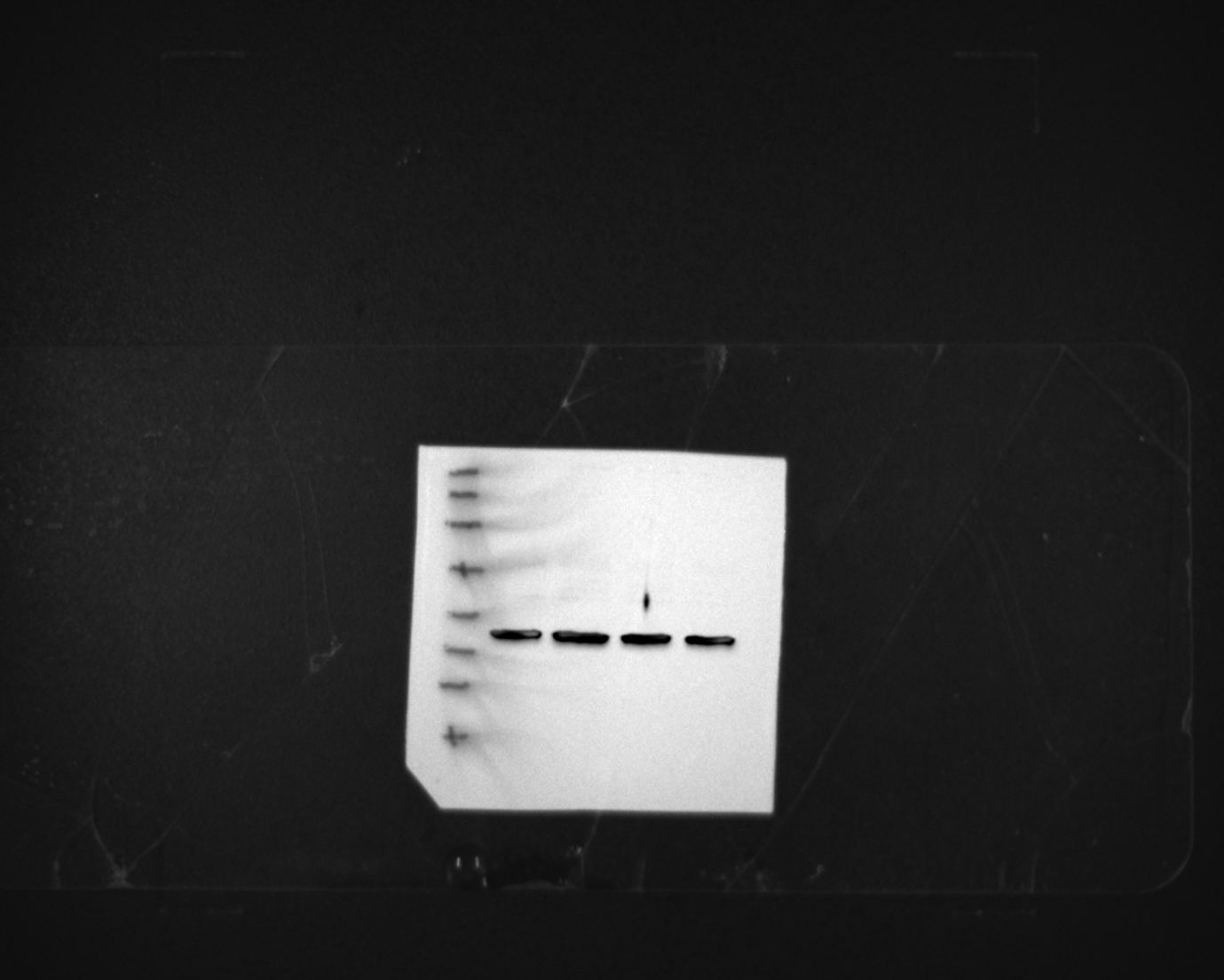

Supplement: Supplementary file 10 — Additional file 10. All Original and uncropped blots images used in manuscript. [file 12915_2022_1437_MOESM10_ESM.zip › blots images/Fig 3/Fig 3 A/Fig3A-beta-actin-marker.tif]

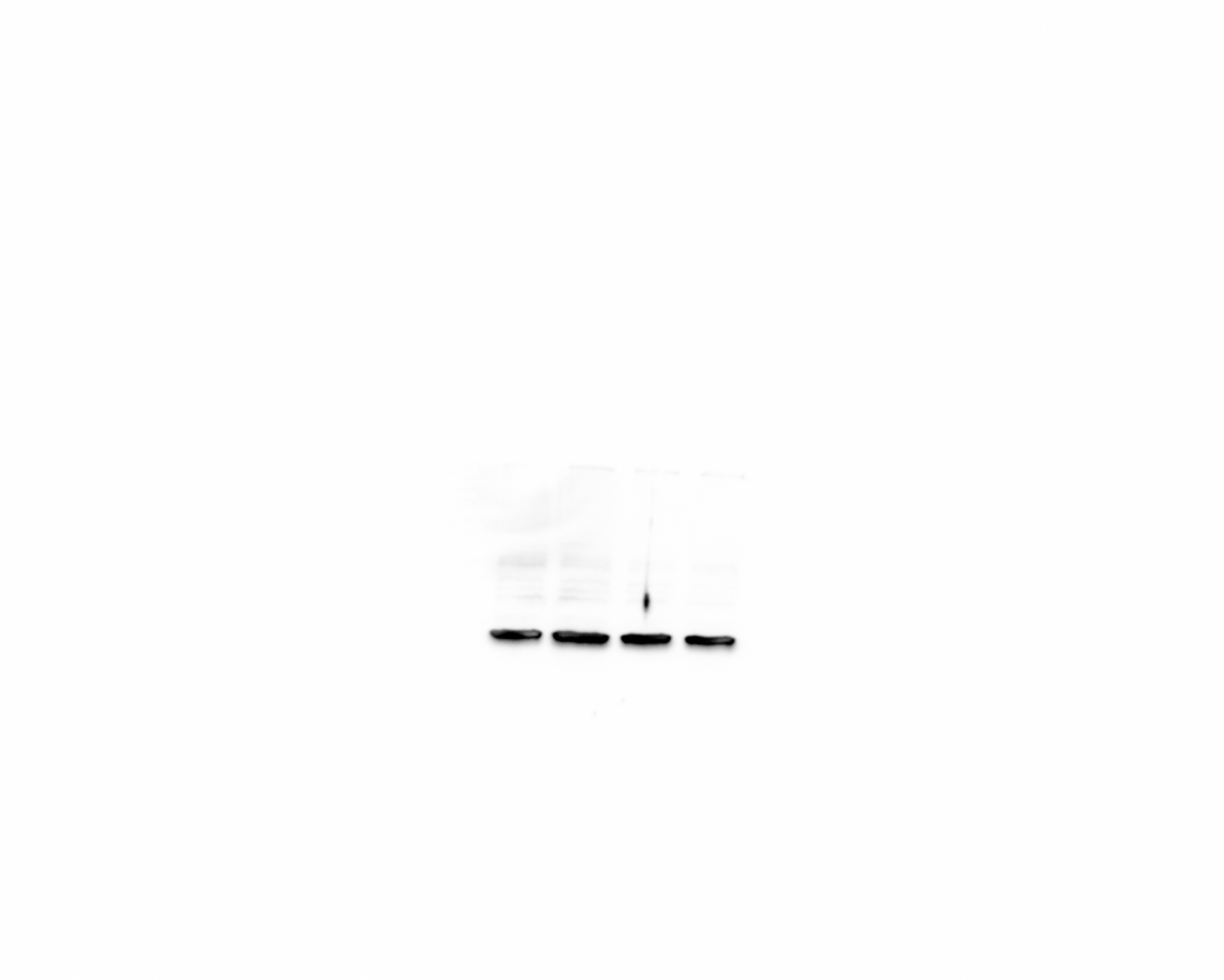

Supplement: Supplementary file 10 — Additional file 10. All Original and uncropped blots images used in manuscript. [file 12915_2022_1437_MOESM10_ESM.zip › blots images/Fig 3/Fig 3 A/Fig3A-beta-actin.tif]

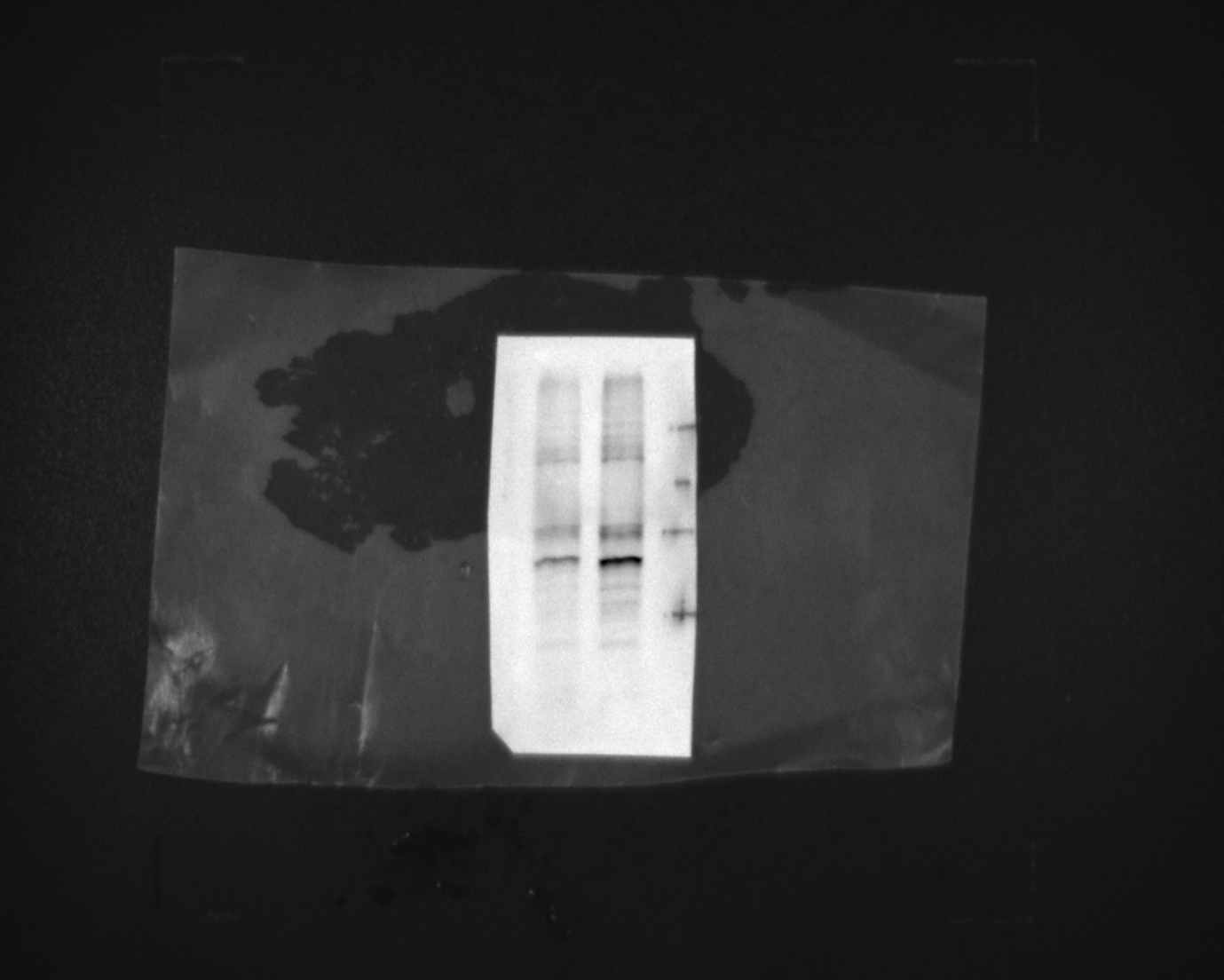

Supplement: Supplementary file 10 — Additional file 10. All Original and uncropped blots images used in manuscript. [file 12915_2022_1437_MOESM10_ESM.zip › blots images/Fig 3/Fig 3 B/anti-HCR-marker.tif]

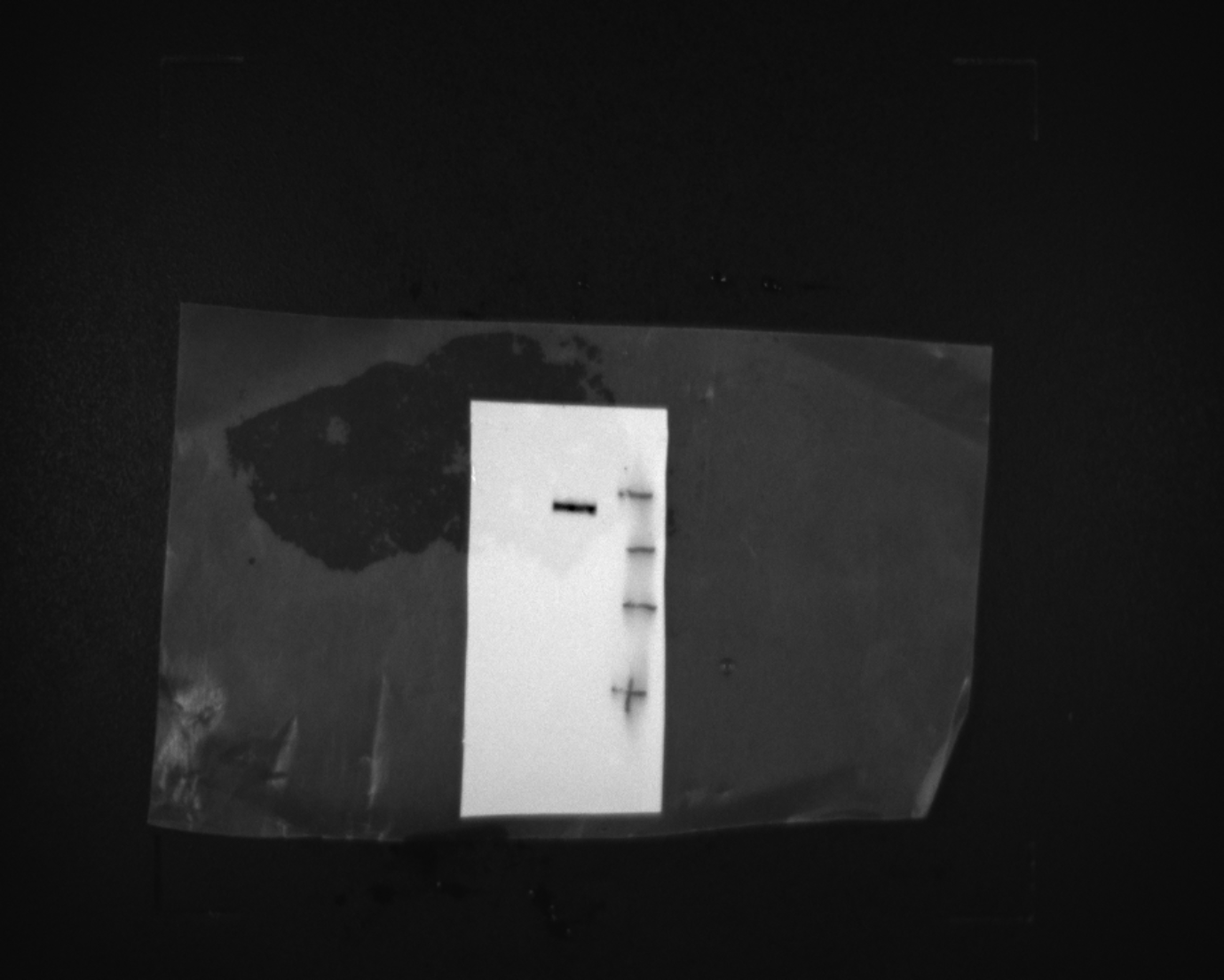

Supplement: Supplementary file 10 — Additional file 10. All Original and uncropped blots images used in manuscript. [file 12915_2022_1437_MOESM10_ESM.zip › blots images/Fig 3/Fig 3 B/Fig3B-anti-GFP-marker.tif]

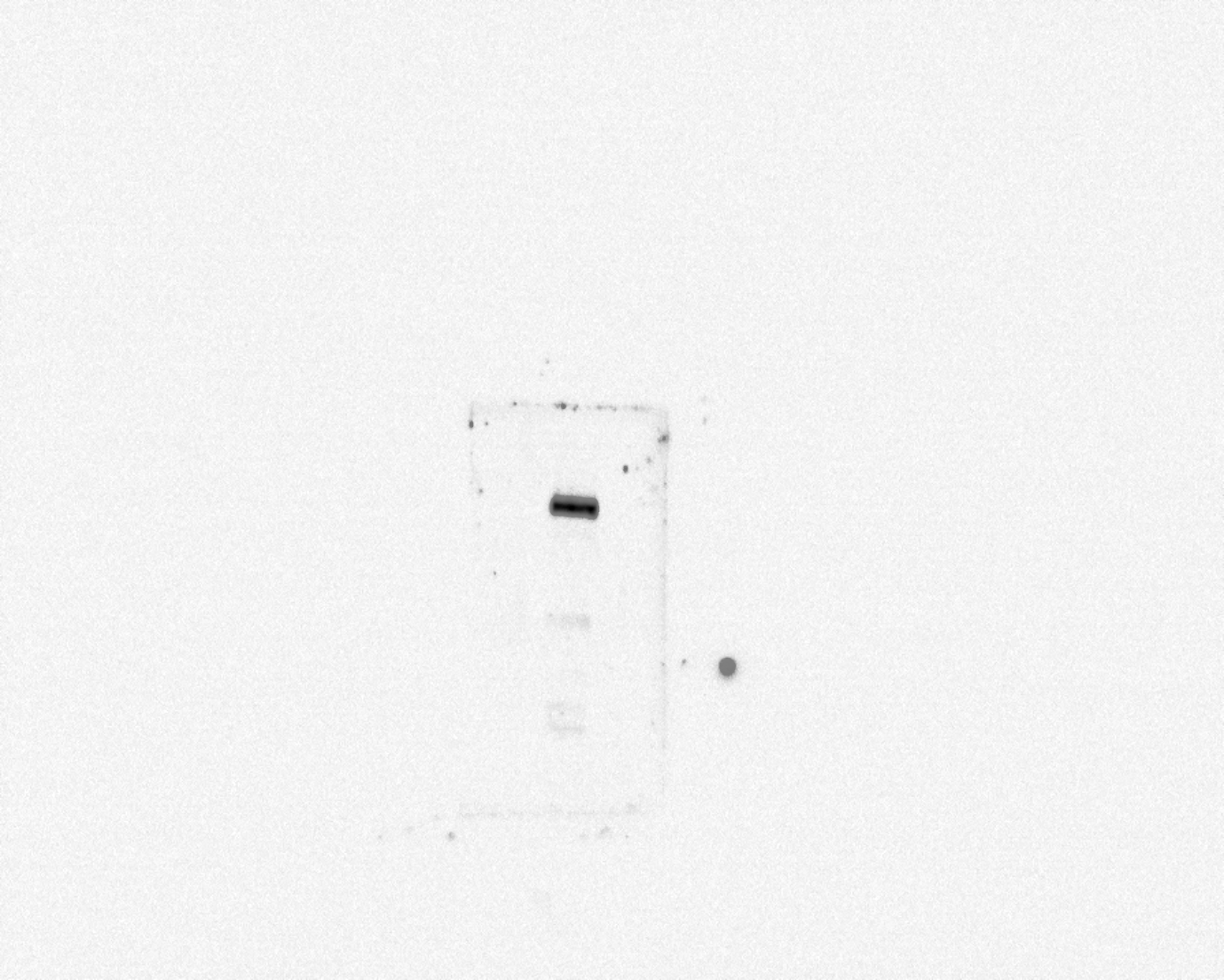

Supplement: Supplementary file 10 — Additional file 10. All Original and uncropped blots images used in manuscript. [file 12915_2022_1437_MOESM10_ESM.zip › blots images/Fig 3/Fig 3 B/Fig3B-anti-GFP.tif]

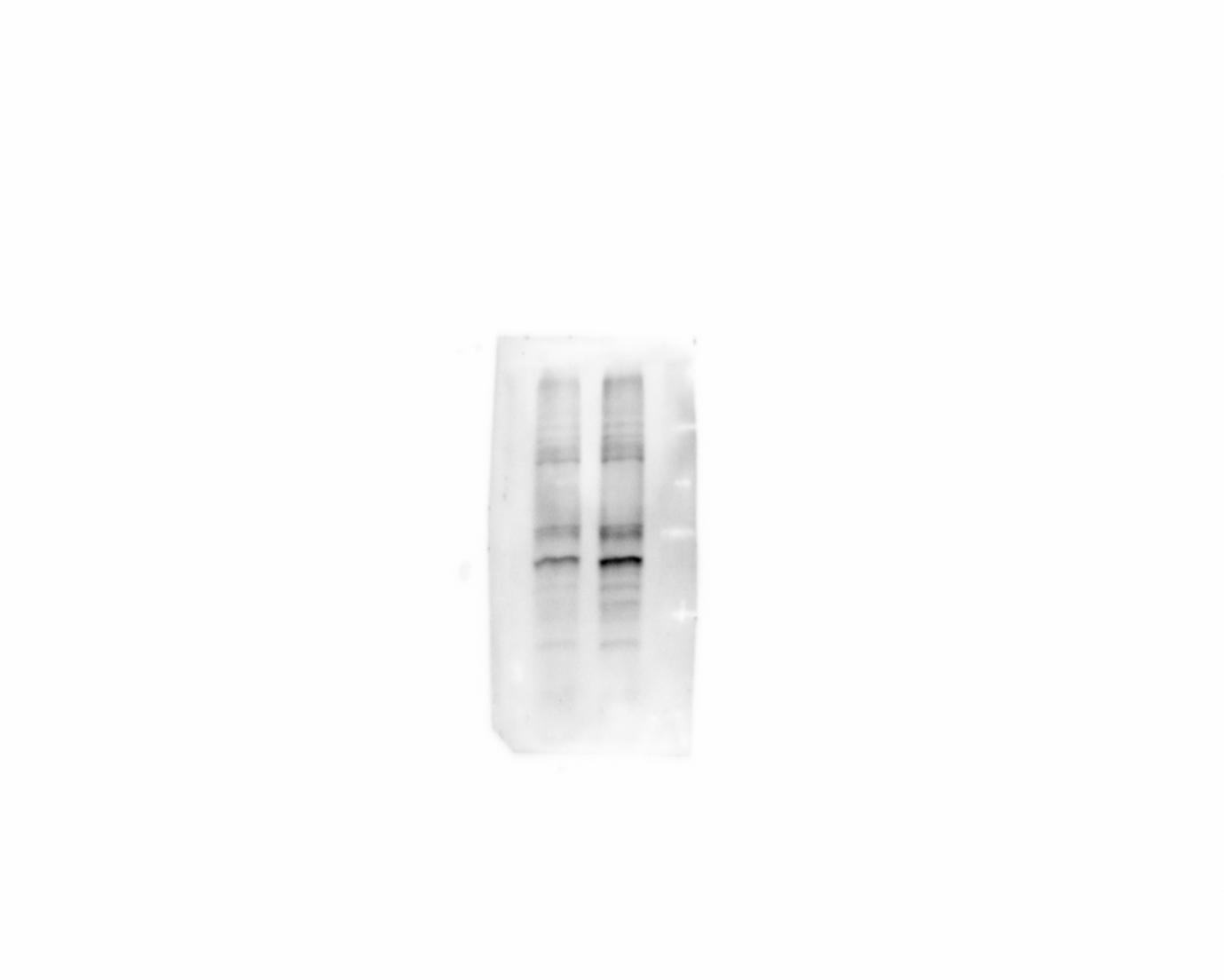

Supplement: Supplementary file 10 — Additional file 10. All Original and uncropped blots images used in manuscript. [file 12915_2022_1437_MOESM10_ESM.zip › blots images/Fig 3/Fig 3 B/Fig3B-anti-HCR.tif]

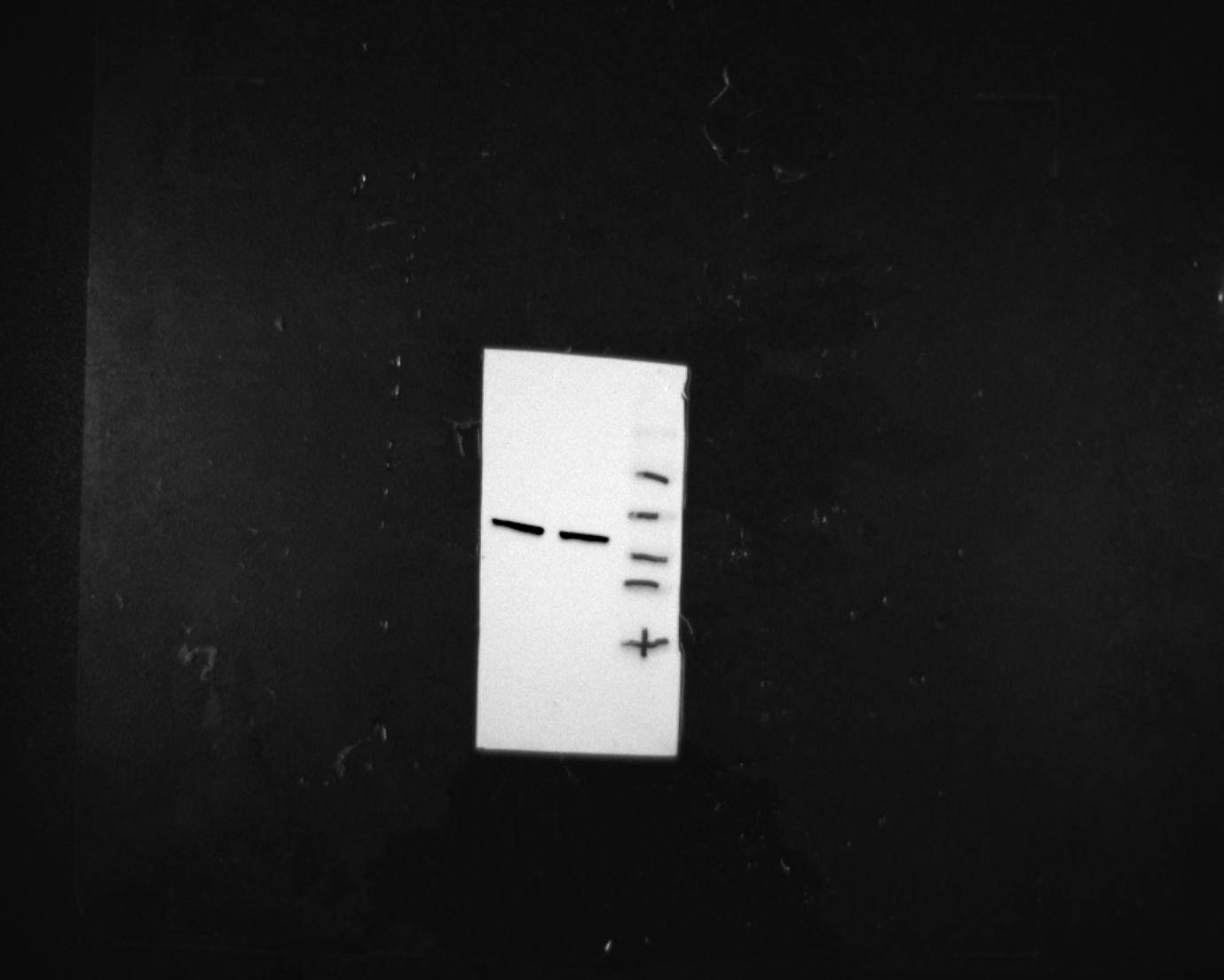

Supplement: Supplementary file 10 — Additional file 10. All Original and uncropped blots images used in manuscript. [file 12915_2022_1437_MOESM10_ESM.zip › blots images/Fig 3/Fig 3 B/Fig3B-beta-actin-marker.tif]

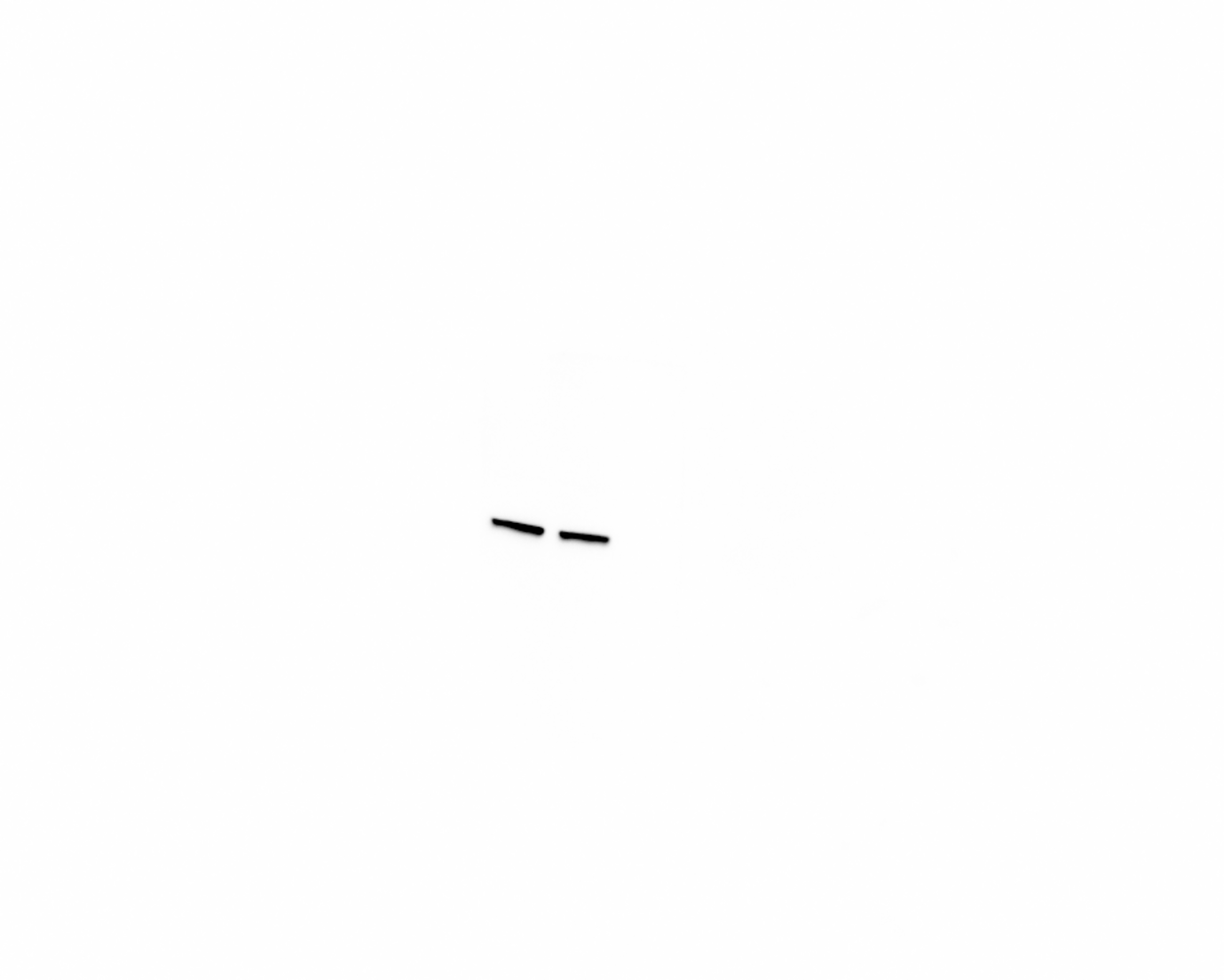

Supplement: Supplementary file 10 — Additional file 10. All Original and uncropped blots images used in manuscript. [file 12915_2022_1437_MOESM10_ESM.zip › blots images/Fig 3/Fig 3 B/Fig3B-beta-actin.tif]

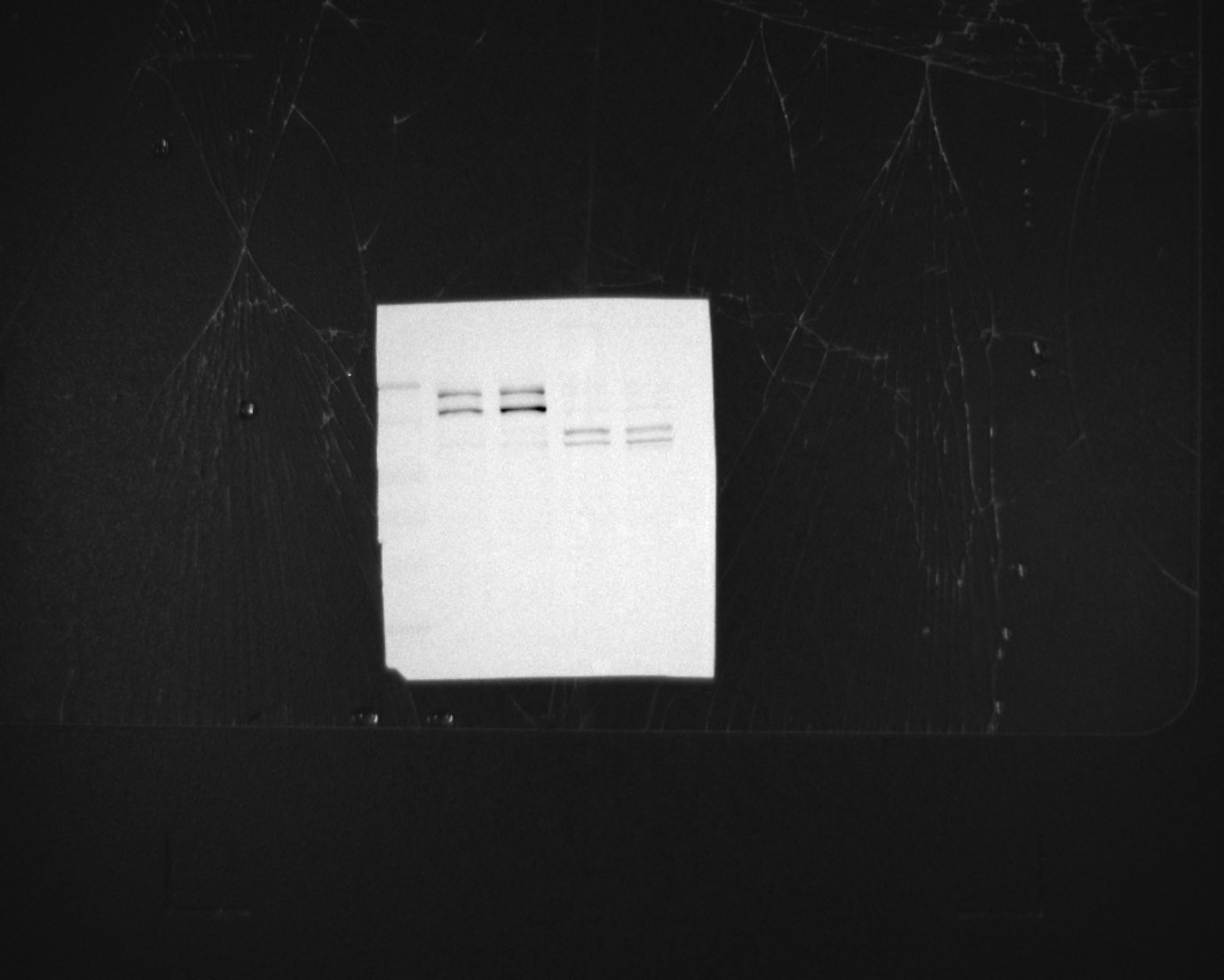

Supplement: Supplementary file 10 — Additional file 10. All Original and uncropped blots images used in manuscript. [file 12915_2022_1437_MOESM10_ESM.zip › blots images/Fig 3/Fig 3 G/Fig3G-astrin-marker.tif]

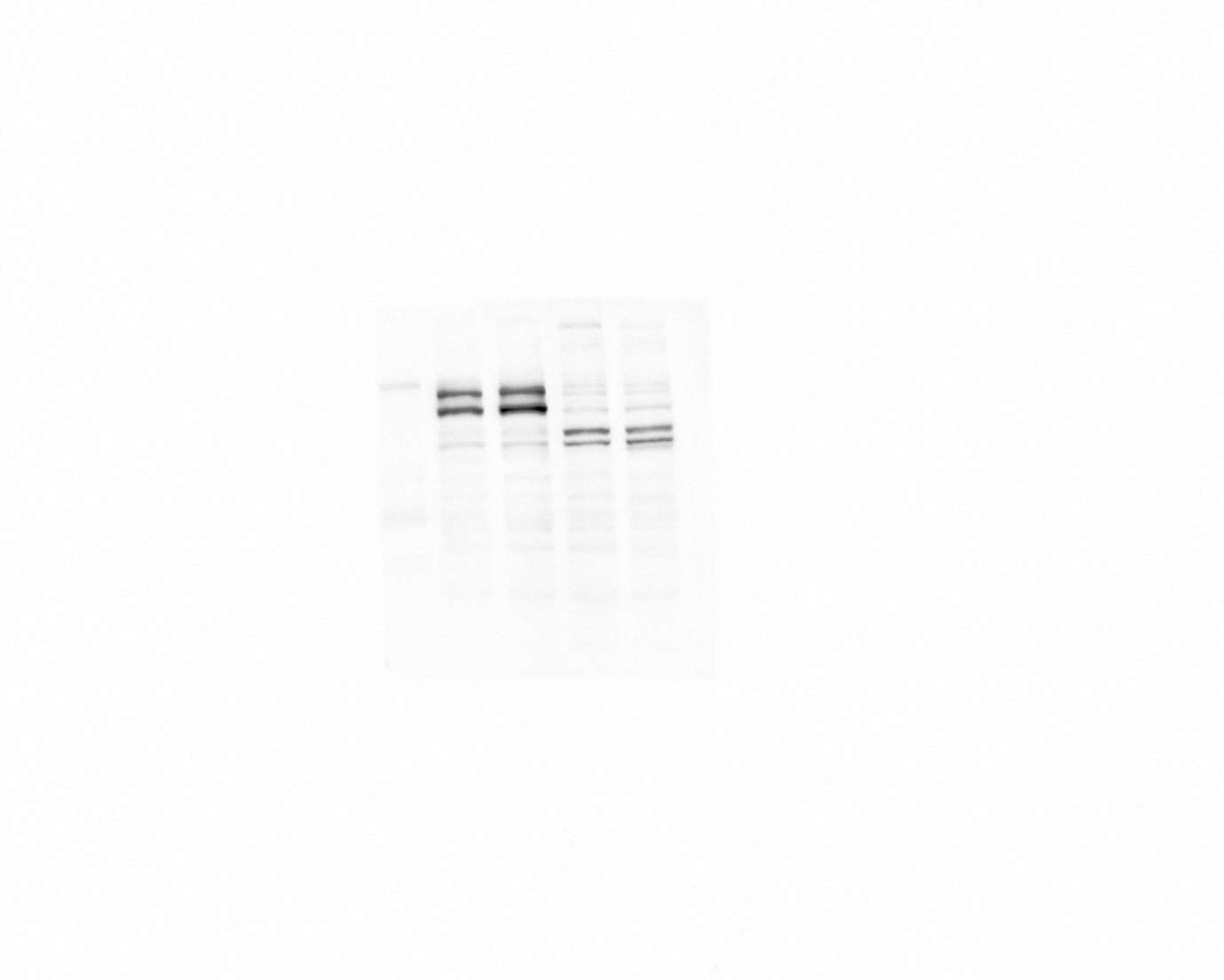

Supplement: Supplementary file 10 — Additional file 10. All Original and uncropped blots images used in manuscript. [file 12915_2022_1437_MOESM10_ESM.zip › blots images/Fig 3/Fig 3 G/Fig3G-astrin.tif]

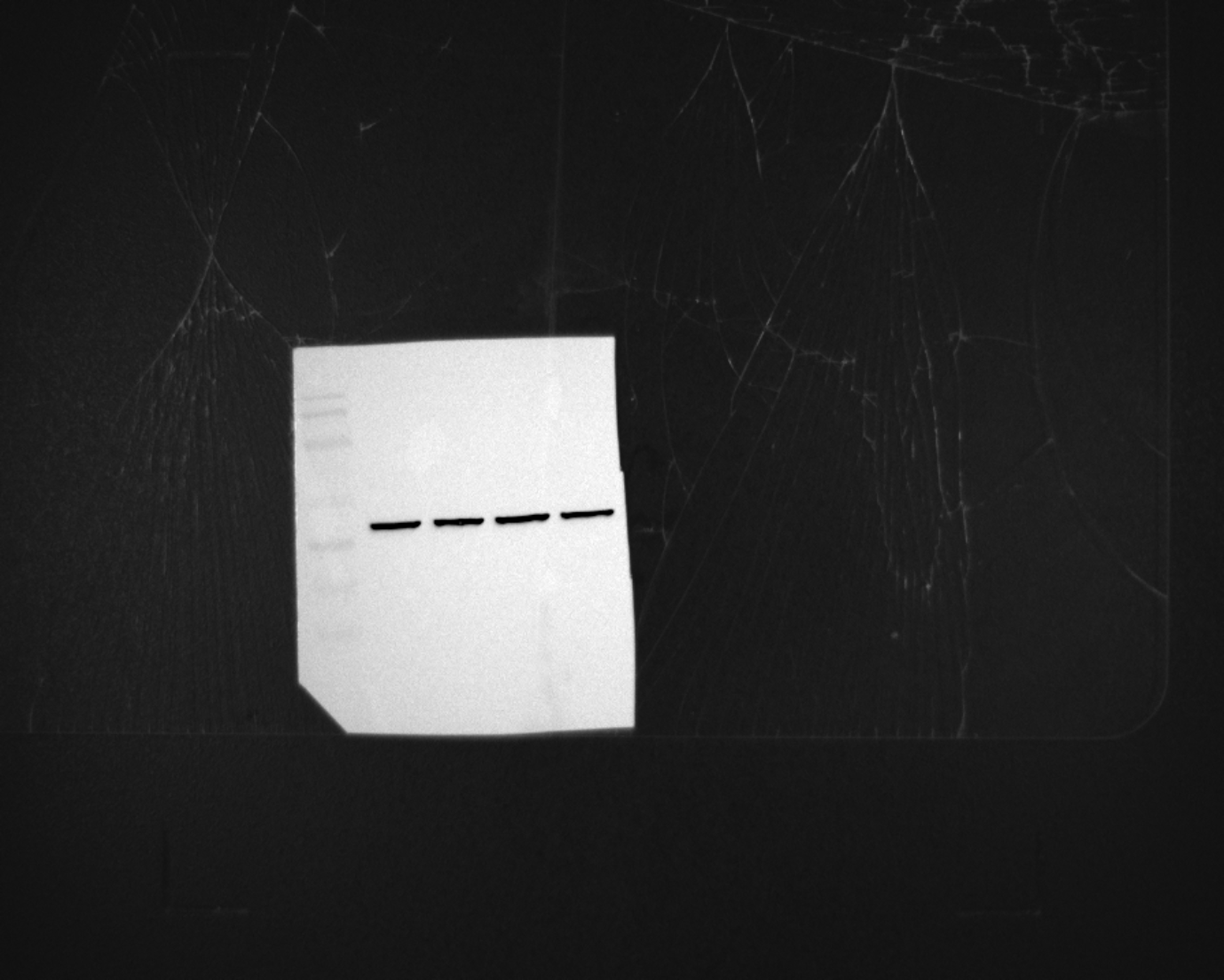

Supplement: Supplementary file 10 — Additional file 10. All Original and uncropped blots images used in manuscript. [file 12915_2022_1437_MOESM10_ESM.zip › blots images/Fig 3/Fig 3 G/Fig3G-beta-actin-marker.tif]

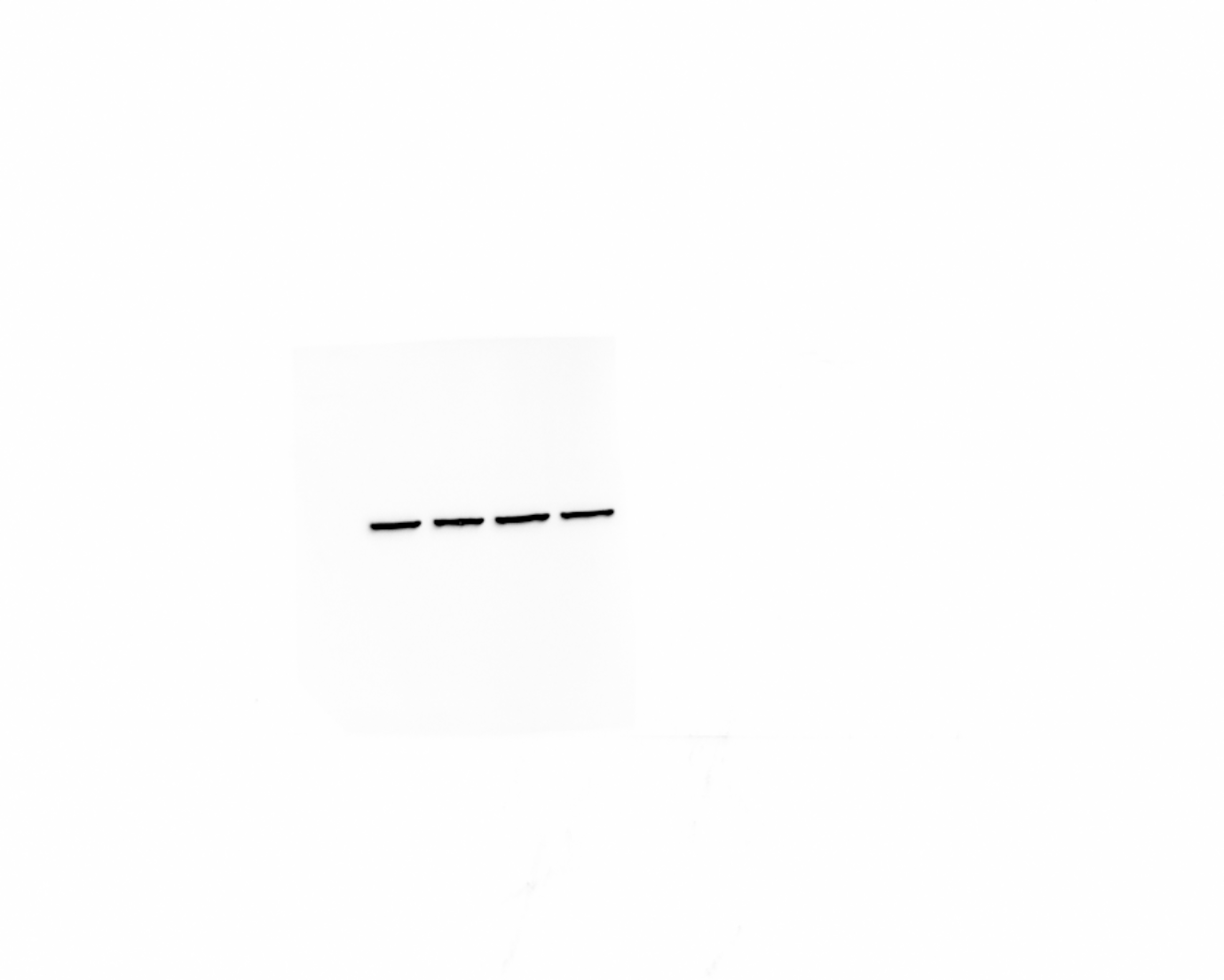

Supplement: Supplementary file 10 — Additional file 10. All Original and uncropped blots images used in manuscript. [file 12915_2022_1437_MOESM10_ESM.zip › blots images/Fig 3/Fig 3 G/Fig3G-beta-actin.tif]

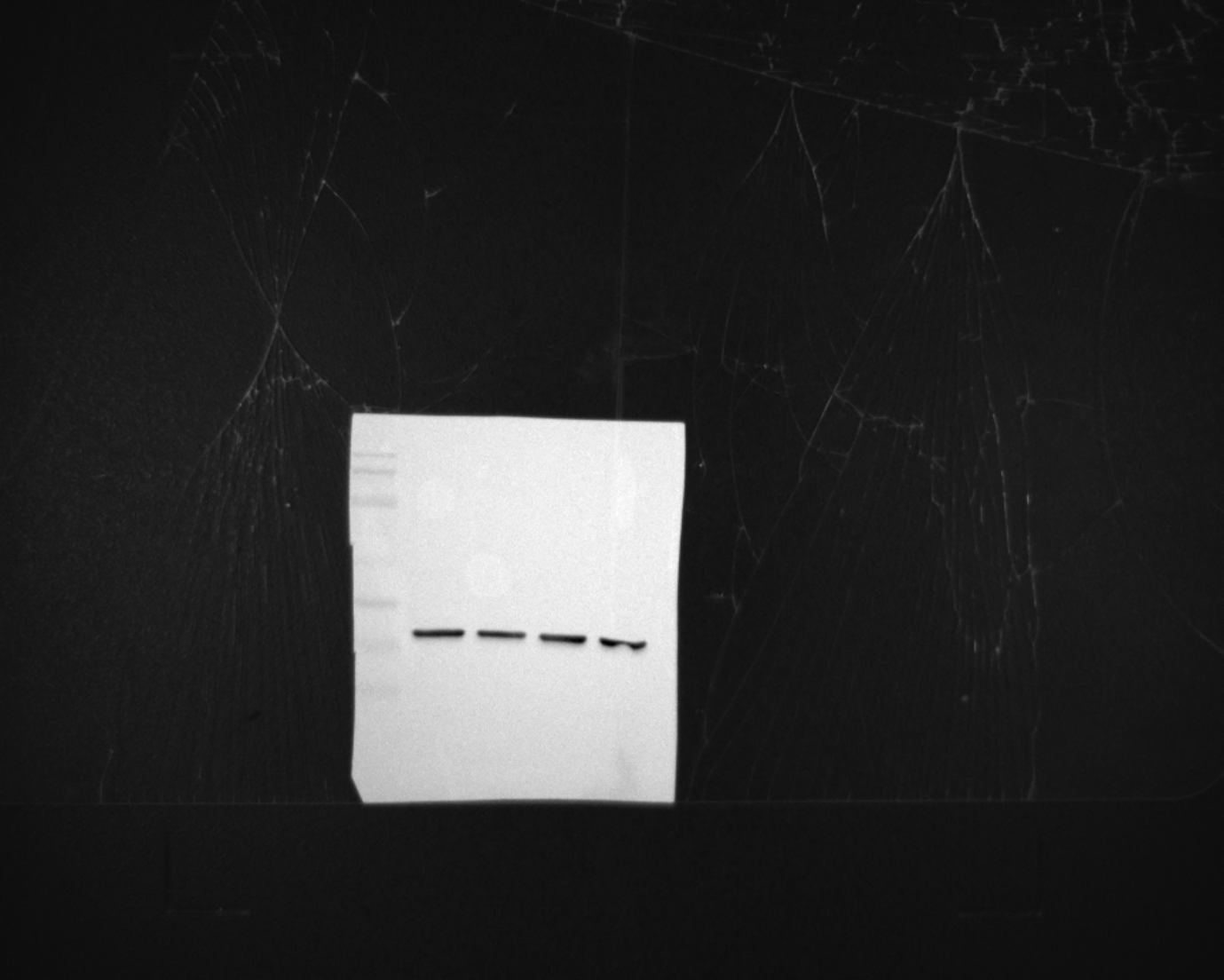

Supplement: Supplementary file 10 — Additional file 10. All Original and uncropped blots images used in manuscript. [file 12915_2022_1437_MOESM10_ESM.zip › blots images/Fig 3/Fig 3 G/Fig3G-GAPDH-marker.tif]

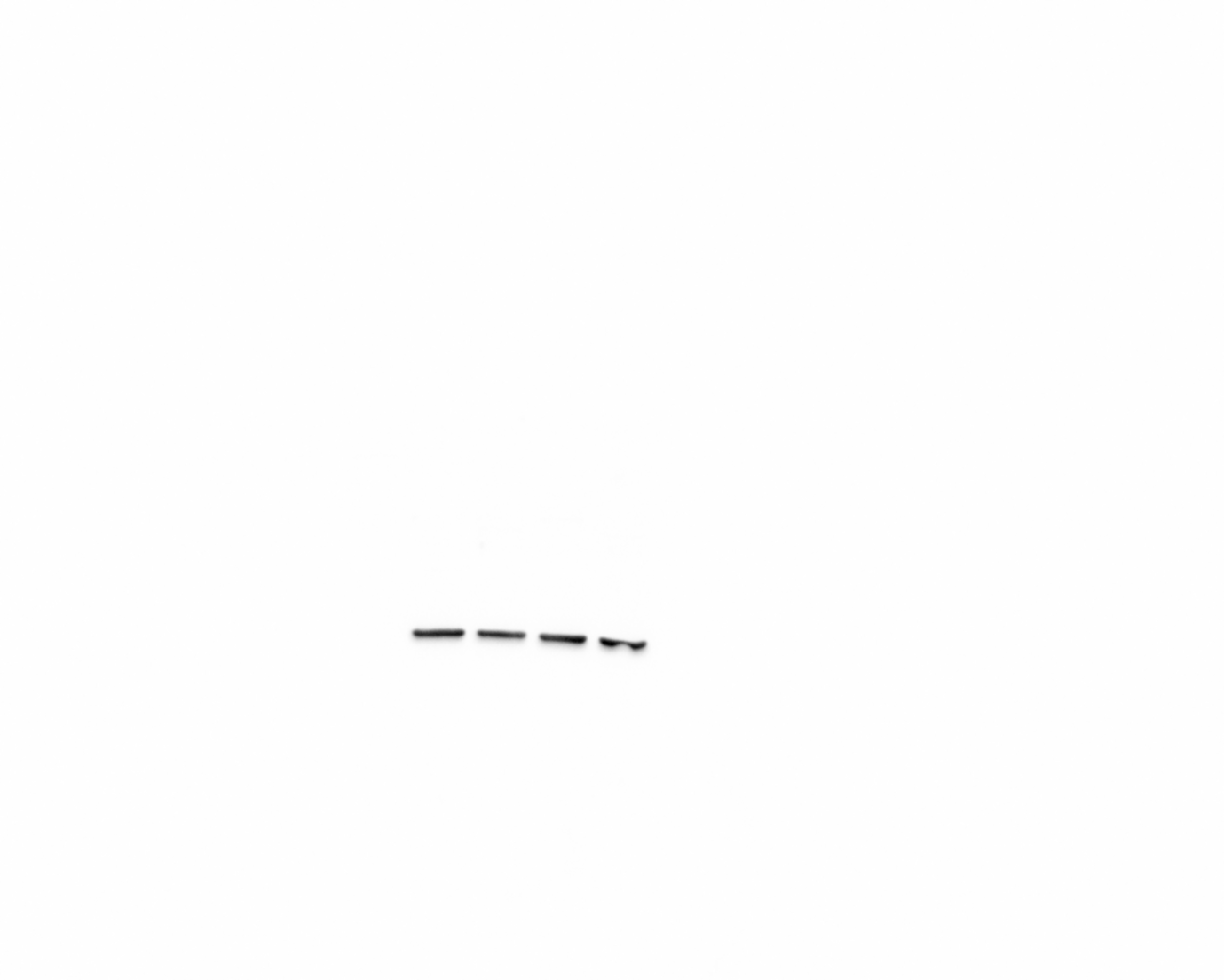

Supplement: Supplementary file 10 — Additional file 10. All Original and uncropped blots images used in manuscript. [file 12915_2022_1437_MOESM10_ESM.zip › blots images/Fig 3/Fig 3 G/Fig3G-GAPDH.tif]

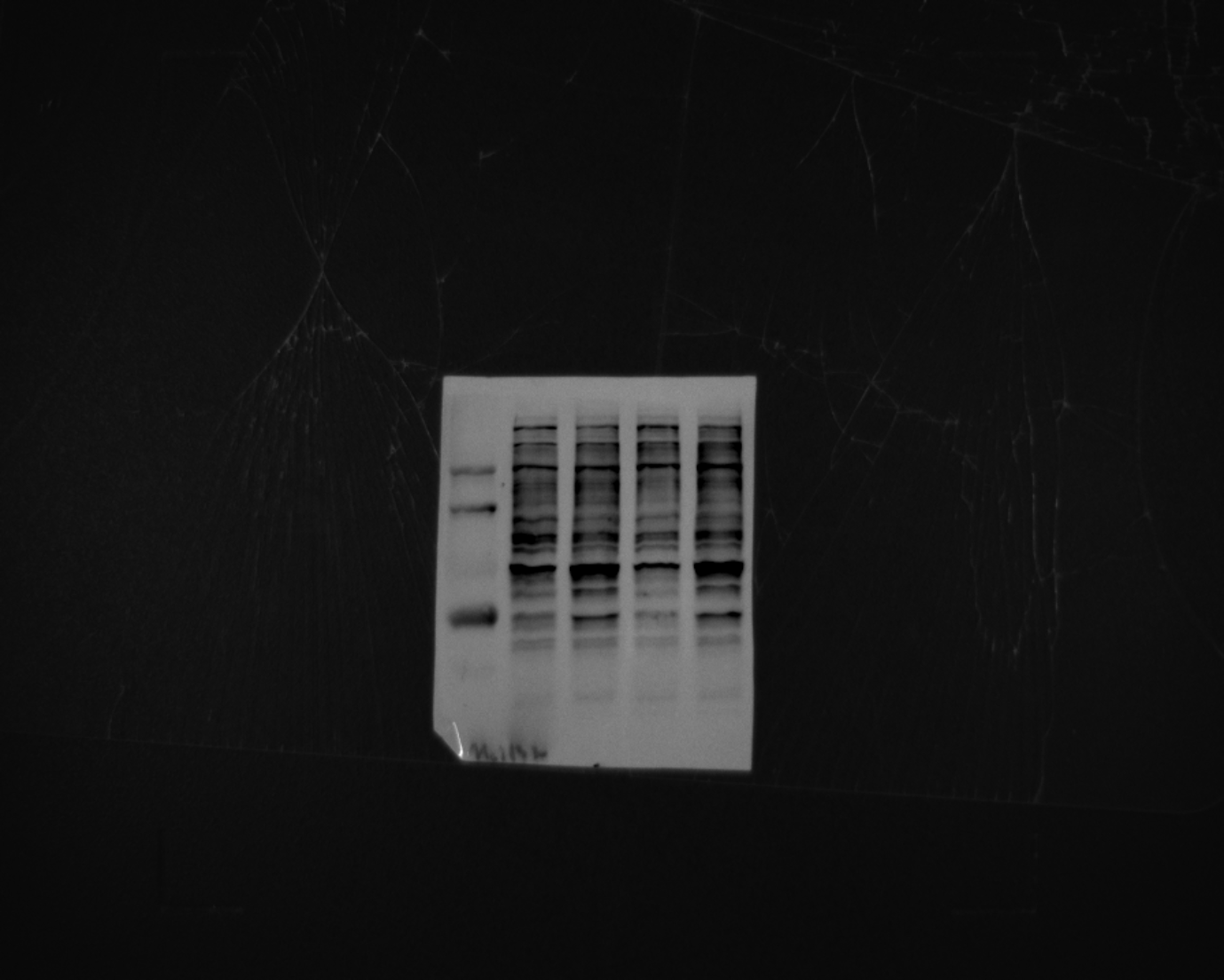

Supplement: Supplementary file 10 — Additional file 10. All Original and uncropped blots images used in manuscript. [file 12915_2022_1437_MOESM10_ESM.zip › blots images/Fig 3/Fig 3 G/Fig3G-HCR-marker.tif]

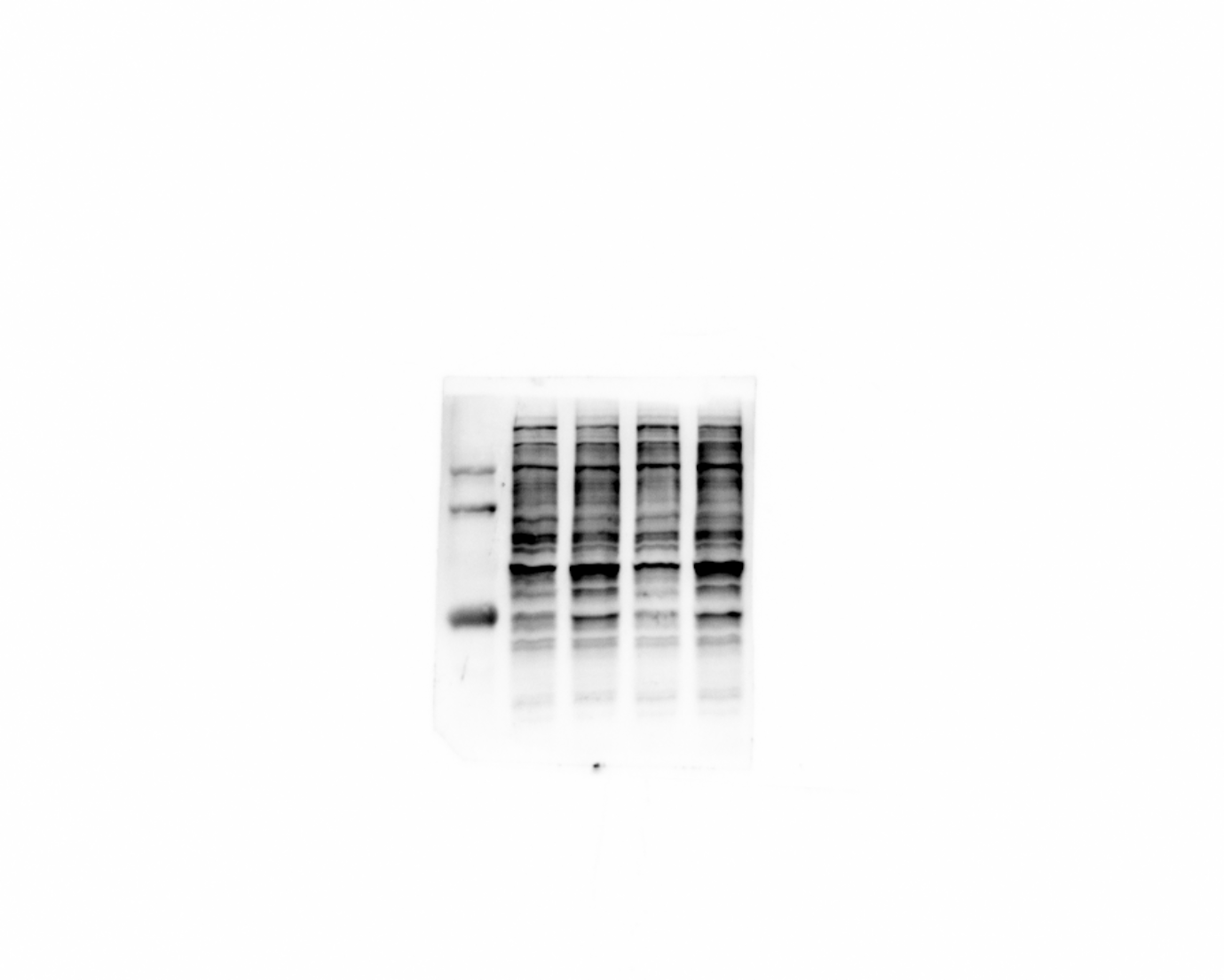

Supplement: Supplementary file 10 — Additional file 10. All Original and uncropped blots images used in manuscript. [file 12915_2022_1437_MOESM10_ESM.zip › blots images/Fig 3/Fig 3 G/Fig3G-HCR.tif]

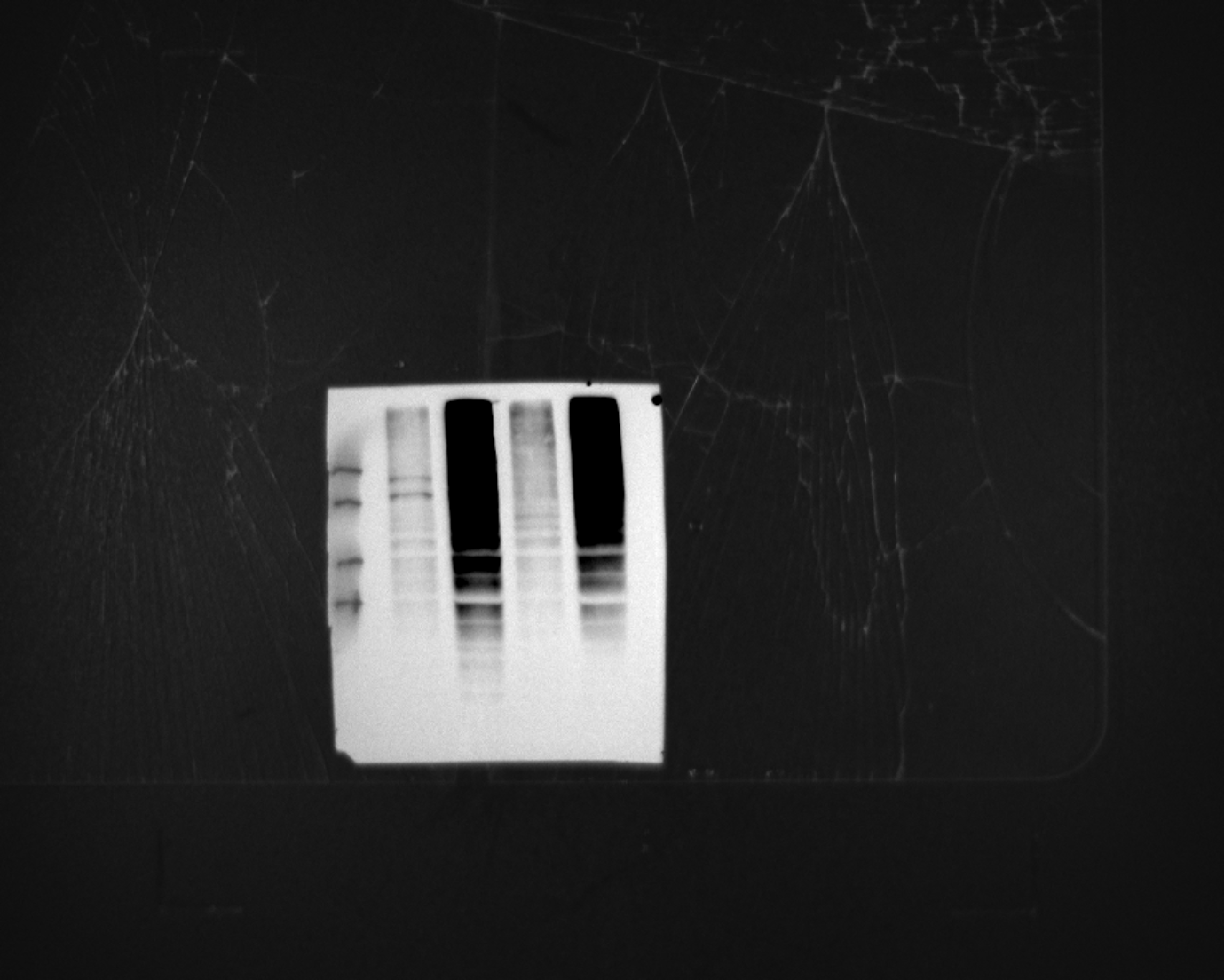

Supplement: Supplementary file 10 — Additional file 10. All Original and uncropped blots images used in manuscript. [file 12915_2022_1437_MOESM10_ESM.zip › blots images/Fig 3/Fig 3 G/Fig3G-Ub-marker.tif]

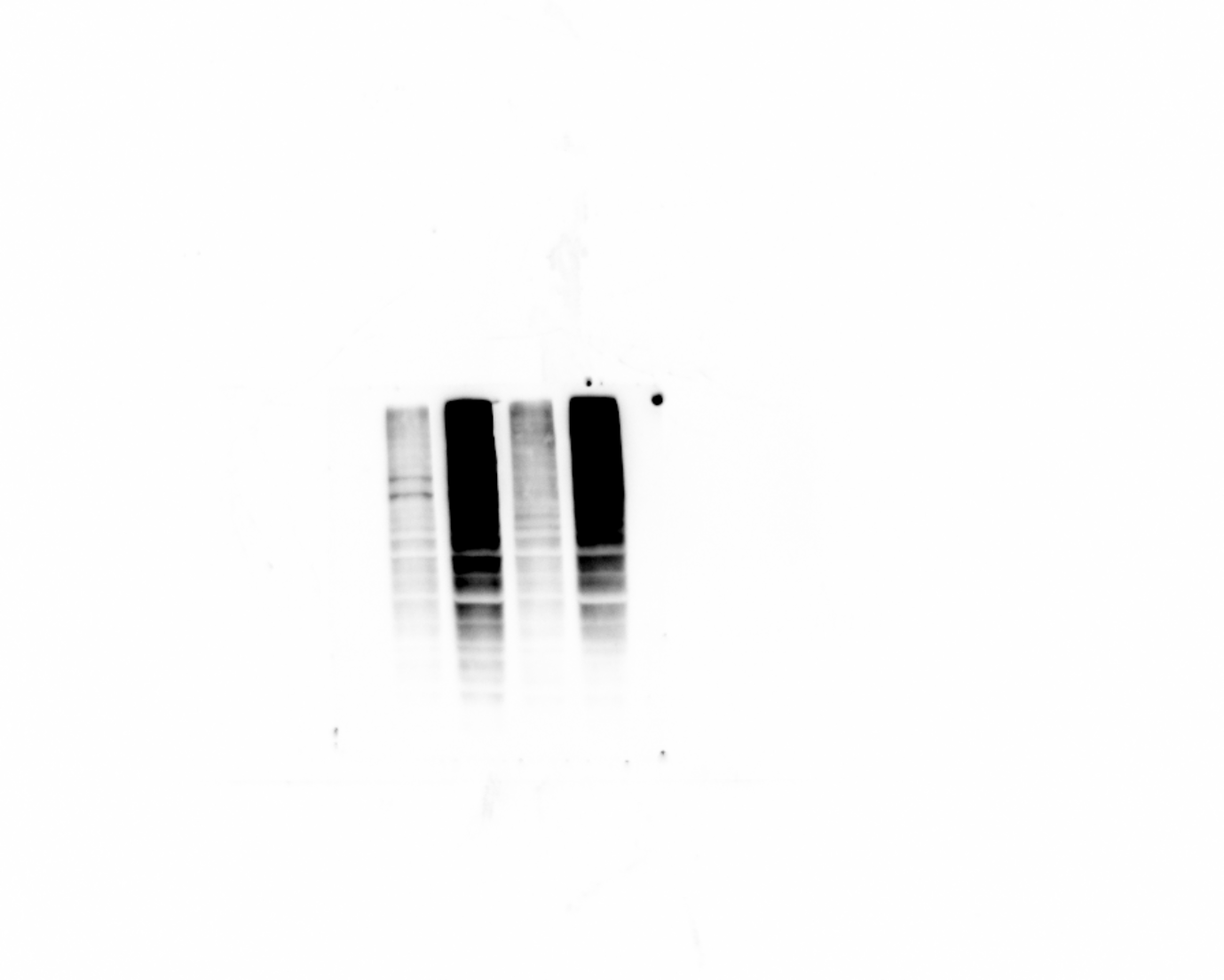

Supplement: Supplementary file 10 — Additional file 10. All Original and uncropped blots images used in manuscript. [file 12915_2022_1437_MOESM10_ESM.zip › blots images/Fig 3/Fig 3 G/Fig3G-Ub.tif]

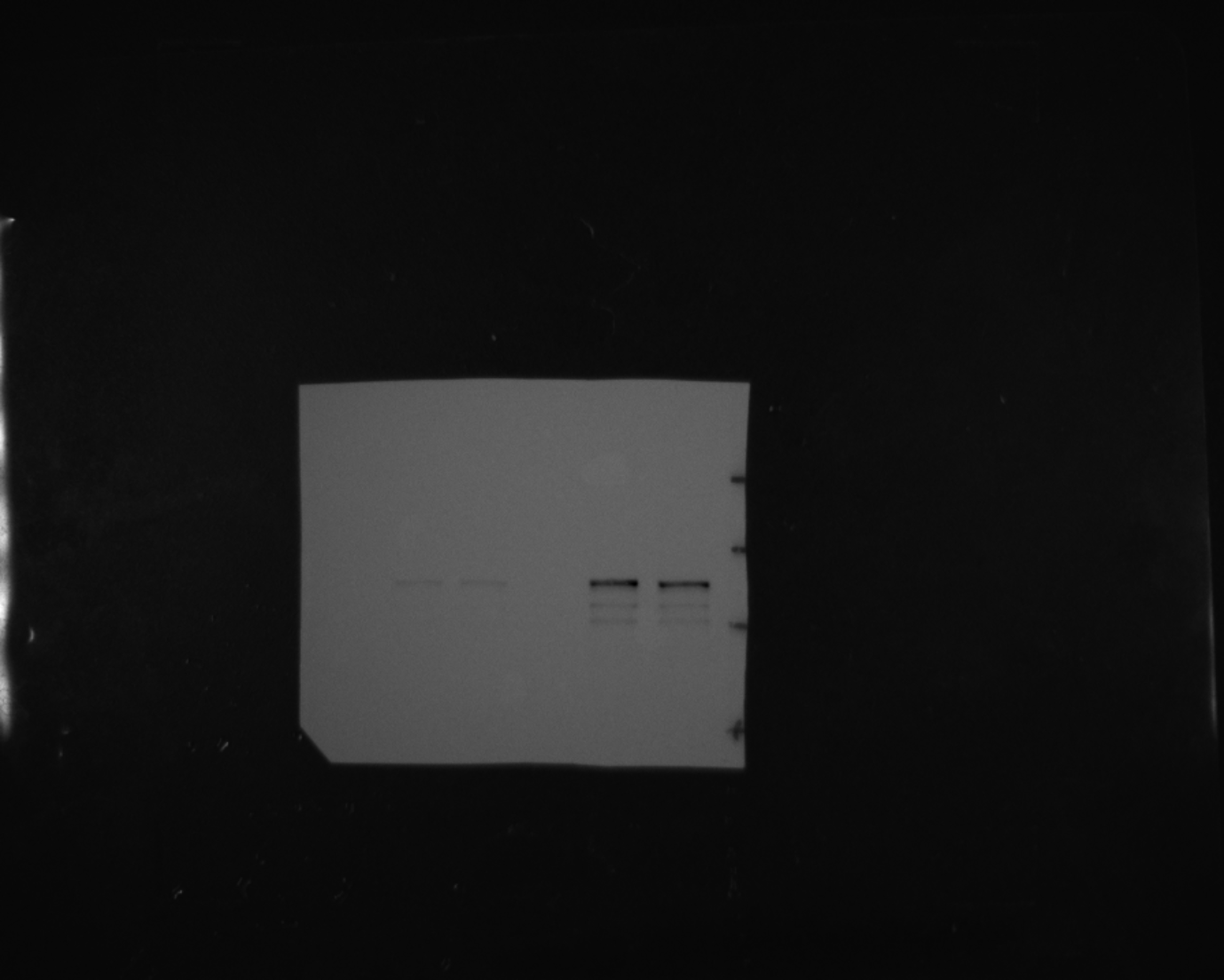

Supplement: Supplementary file 10 — Additional file 10. All Original and uncropped blots images used in manuscript. [file 12915_2022_1437_MOESM10_ESM.zip › blots images/Fig 3/Fig 3 H/Fig3H-left-3-lanes-input-right-3-IP-anti-GFP-marker.tif]

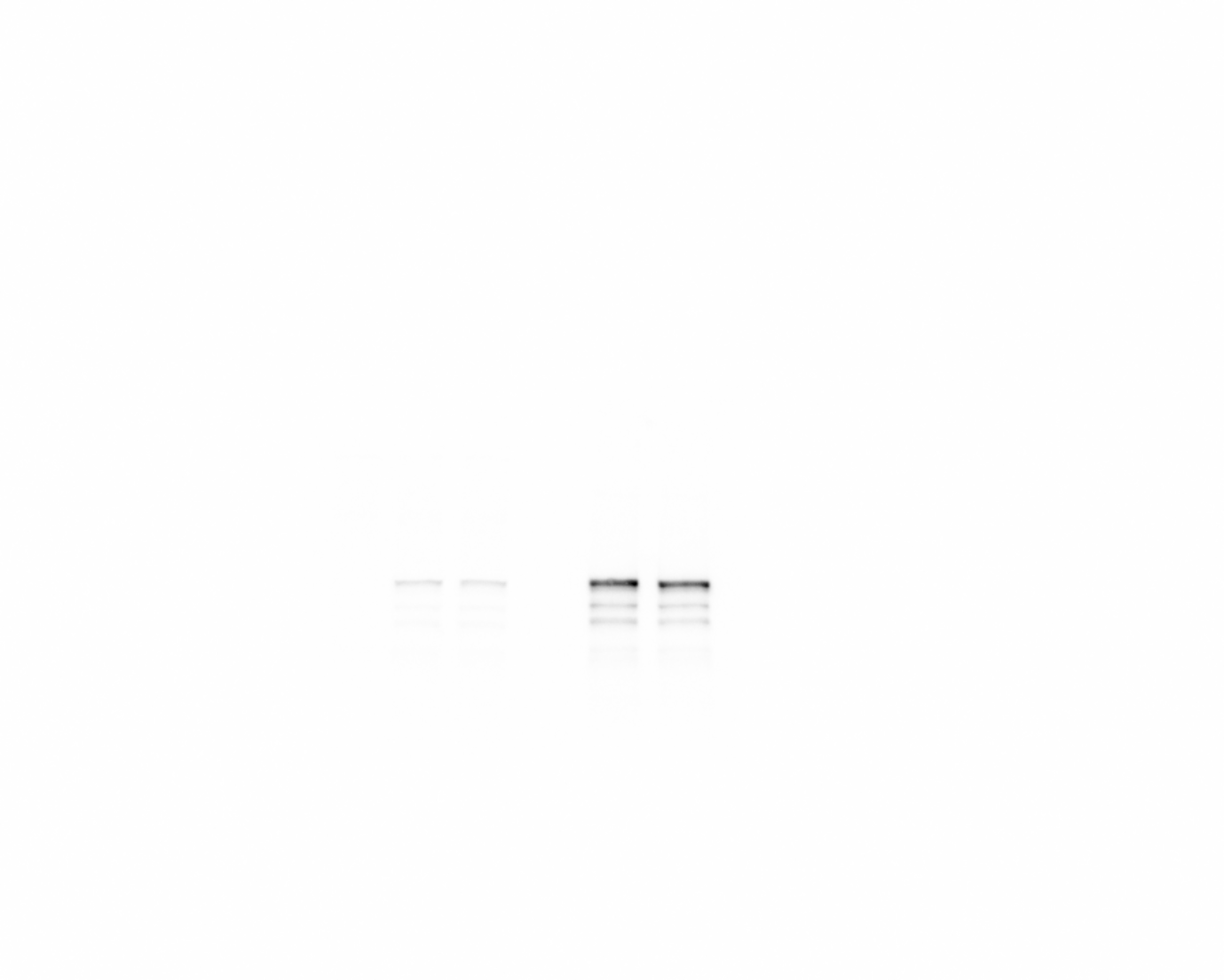

Supplement: Supplementary file 10 — Additional file 10. All Original and uncropped blots images used in manuscript. [file 12915_2022_1437_MOESM10_ESM.zip › blots images/Fig 3/Fig 3 H/Fig3H-left-3-lanes-input-right-3-IP-anti-GFP.tif]

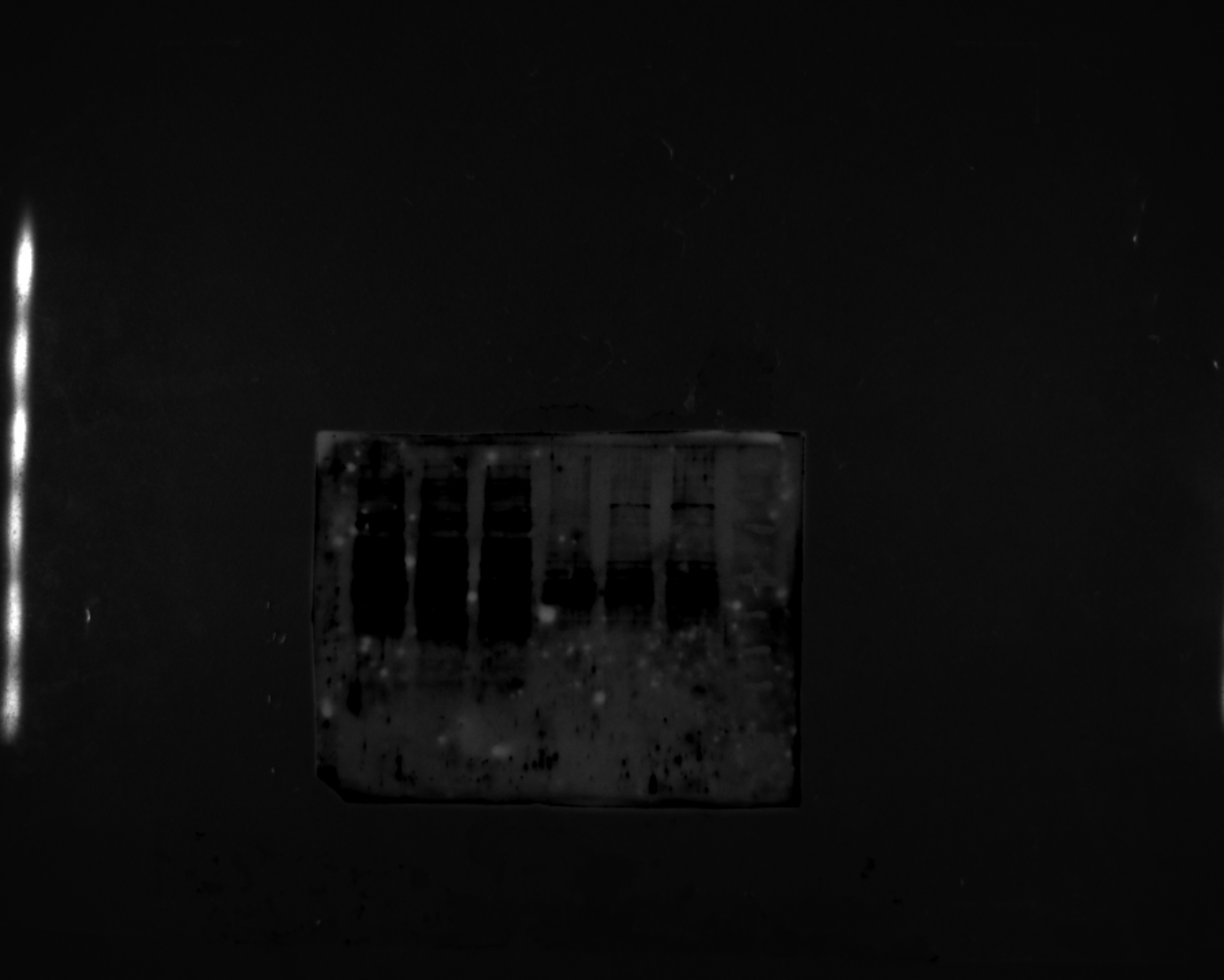

Supplement: Supplementary file 10 — Additional file 10. All Original and uncropped blots images used in manuscript. [file 12915_2022_1437_MOESM10_ESM.zip › blots images/Fig 3/Fig 3 H/Fig3H-left-3-lanes-input-right-3-IP-anti-HA-2-marker.tif]

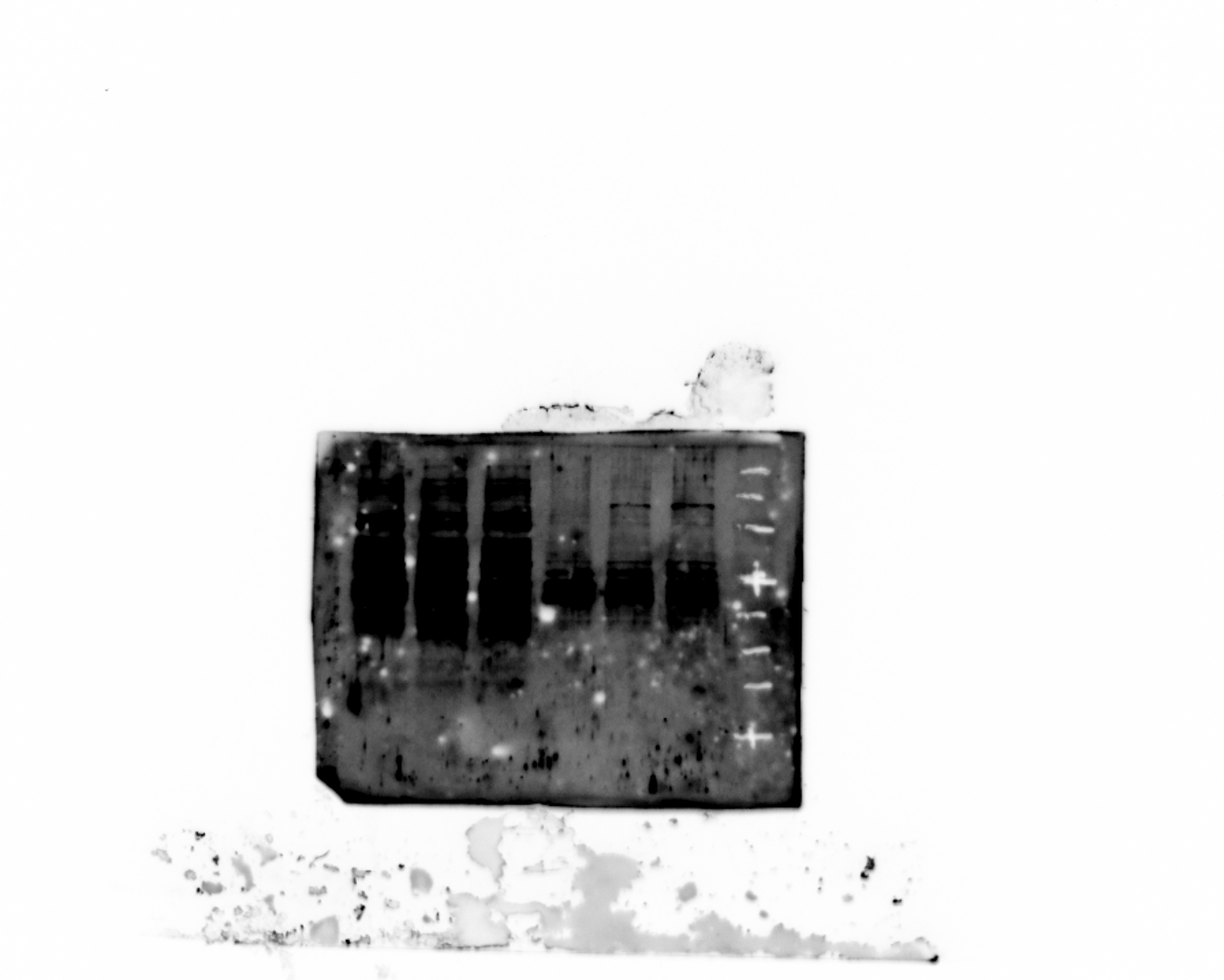

Supplement: Supplementary file 10 — Additional file 10. All Original and uncropped blots images used in manuscript. [file 12915_2022_1437_MOESM10_ESM.zip › blots images/Fig 3/Fig 3 H/Fig3H-left-3-lanes-input-right-3-IP-anti-HA-2.tif]

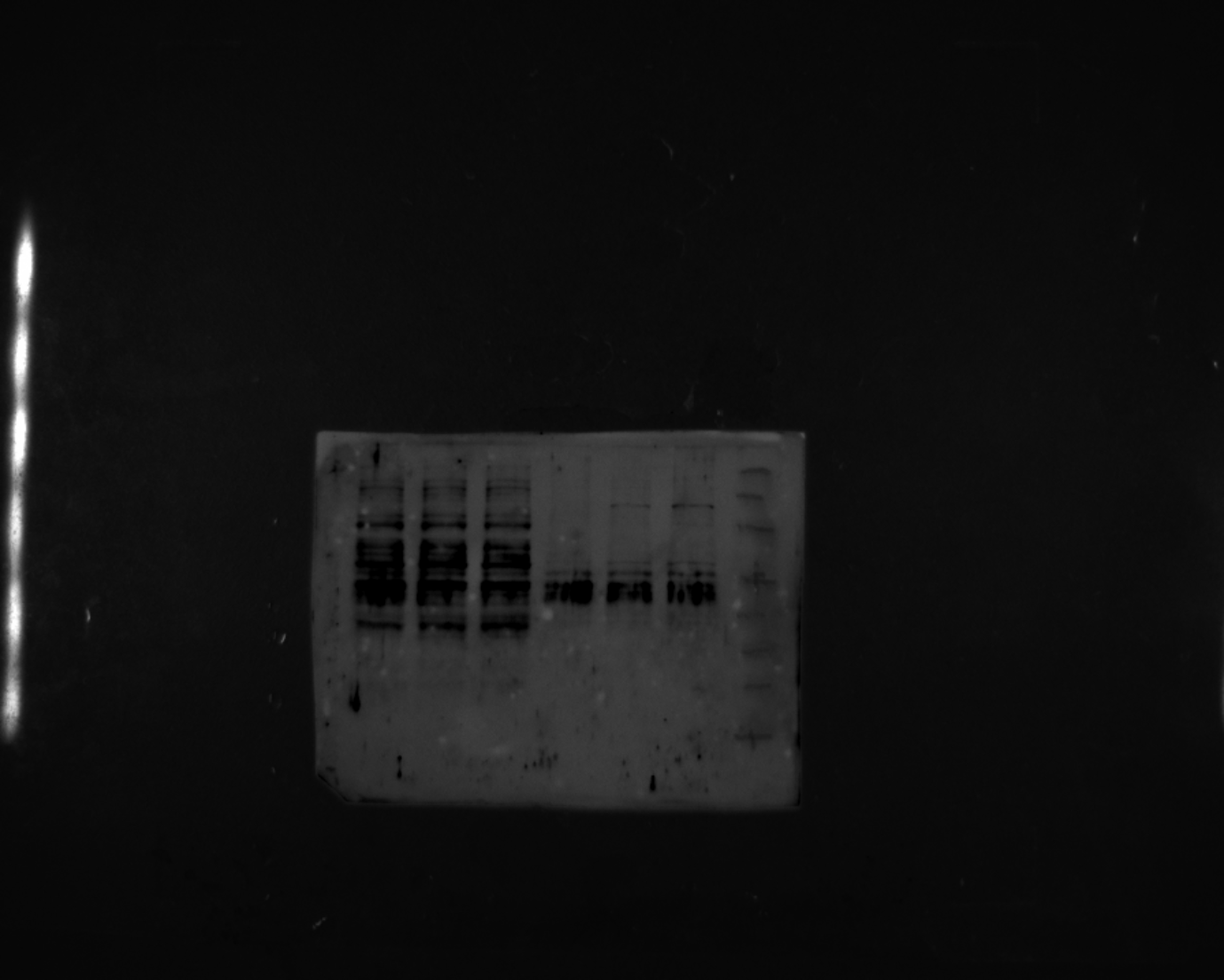

Supplement: Supplementary file 10 — Additional file 10. All Original and uncropped blots images used in manuscript. [file 12915_2022_1437_MOESM10_ESM.zip › blots images/Fig 3/Fig 3 H/Fig3H-left-3-lanes-input-right-3-IP-anti-HA-marker.tif]

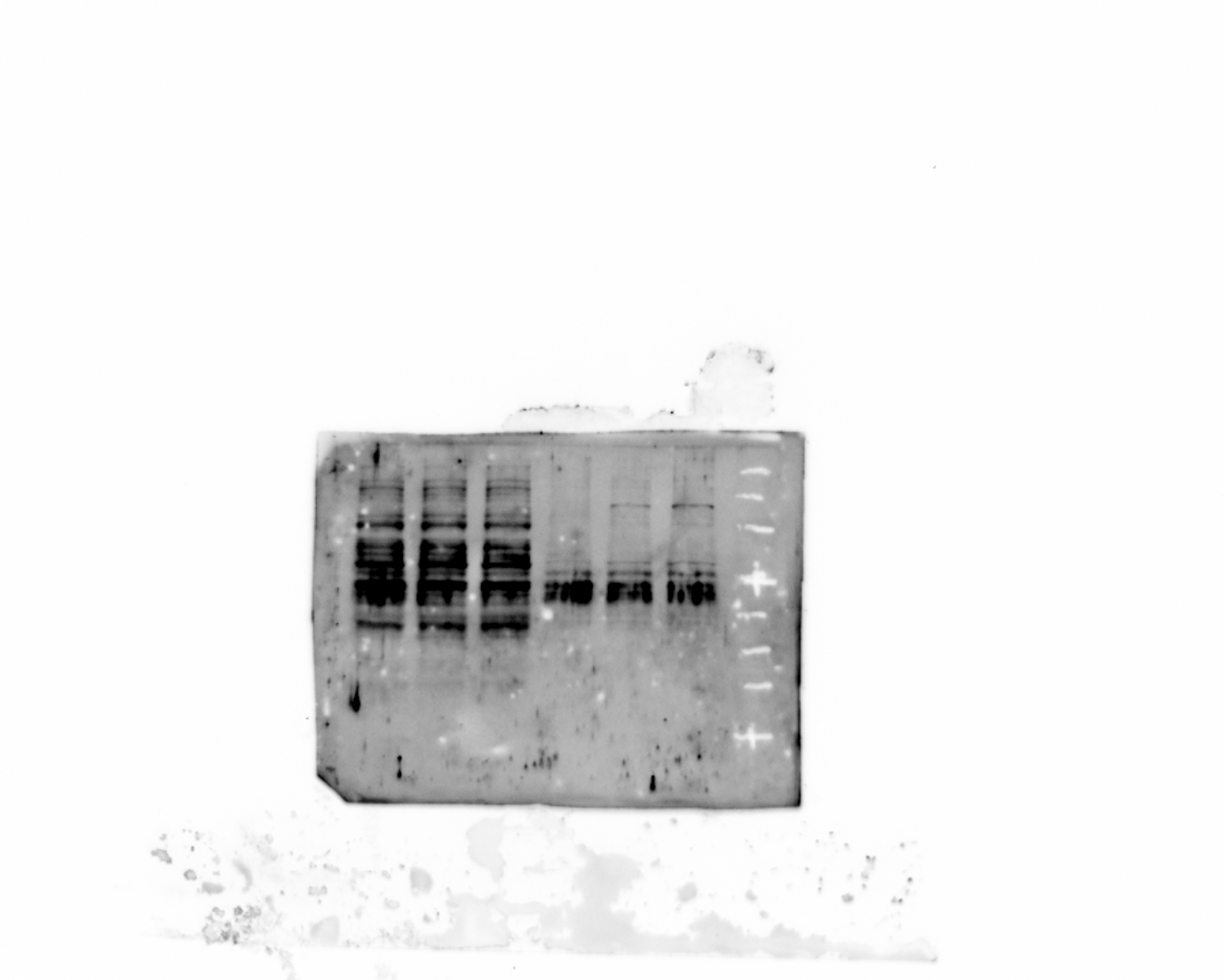

Supplement: Supplementary file 10 — Additional file 10. All Original and uncropped blots images used in manuscript. [file 12915_2022_1437_MOESM10_ESM.zip › blots images/Fig 3/Fig 3 H/Fig3H-left-3-lanes-input-right-3-IP-anti-HA.tif]

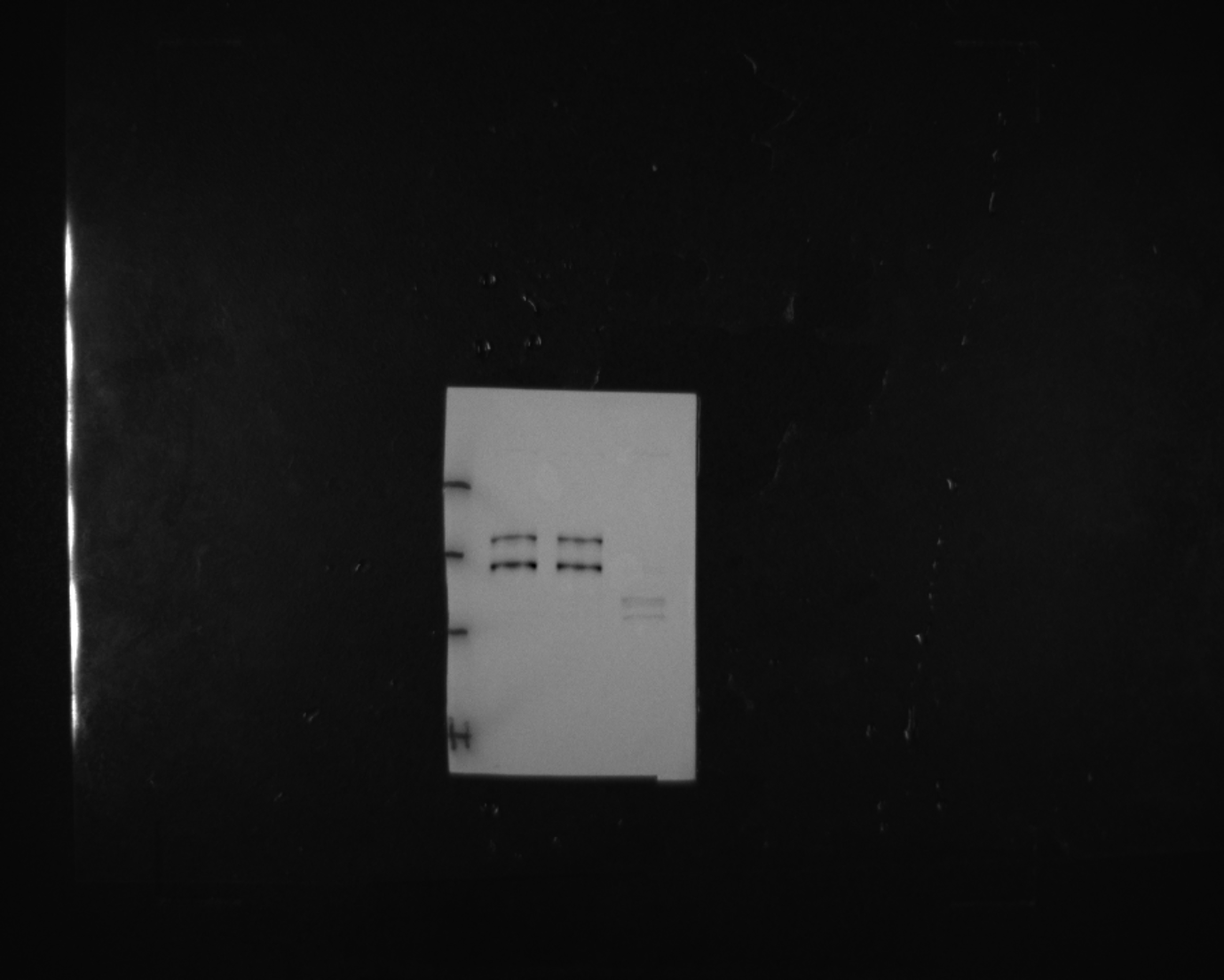

Supplement: Supplementary file 10 — Additional file 10. All Original and uncropped blots images used in manuscript. [file 12915_2022_1437_MOESM10_ESM.zip › blots images/Fig 3/Fig 3 H/input-astrin-marker.tif]

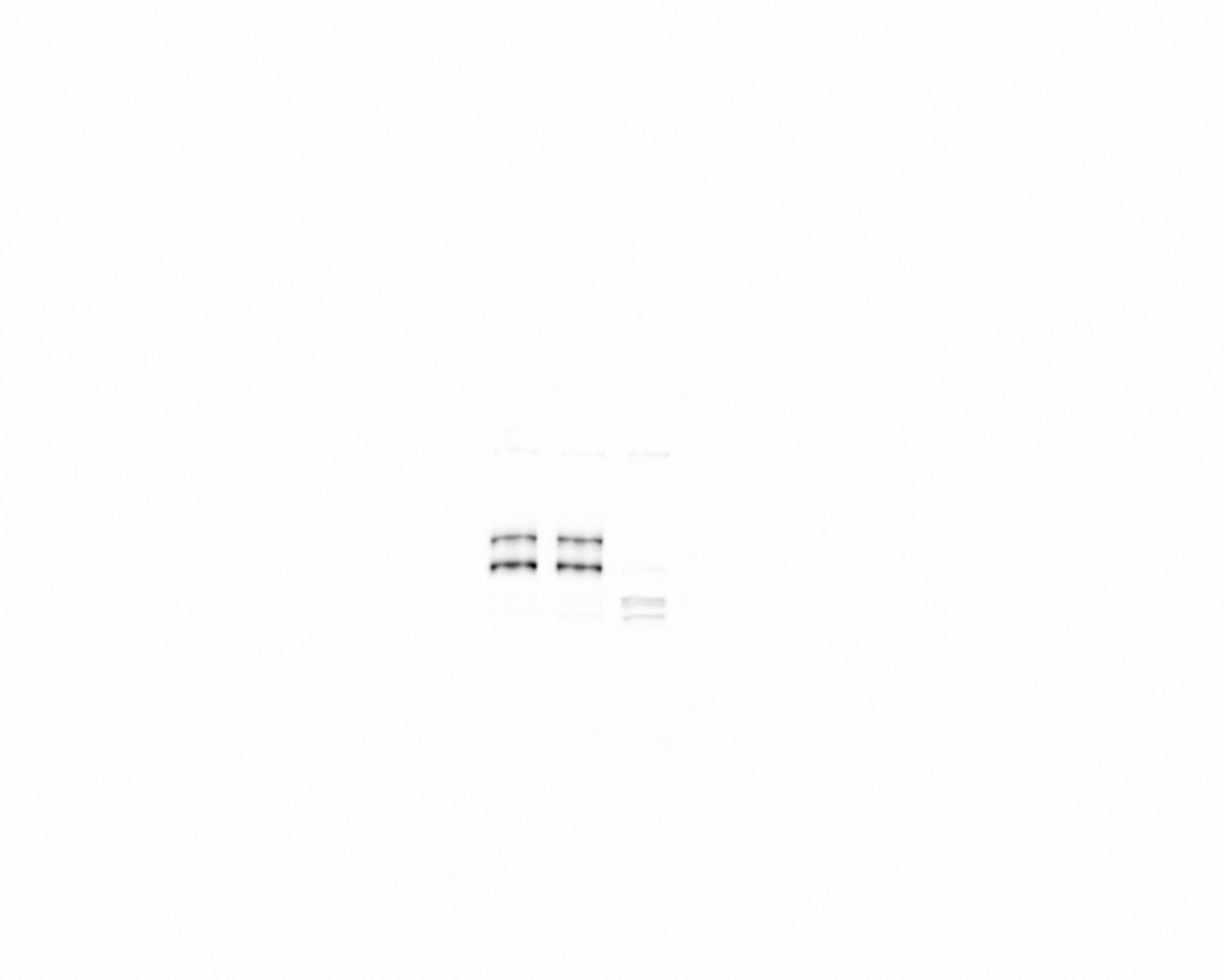

Supplement: Supplementary file 10 — Additional file 10. All Original and uncropped blots images used in manuscript. [file 12915_2022_1437_MOESM10_ESM.zip › blots images/Fig 3/Fig 3 H/Input-astrin.tif]

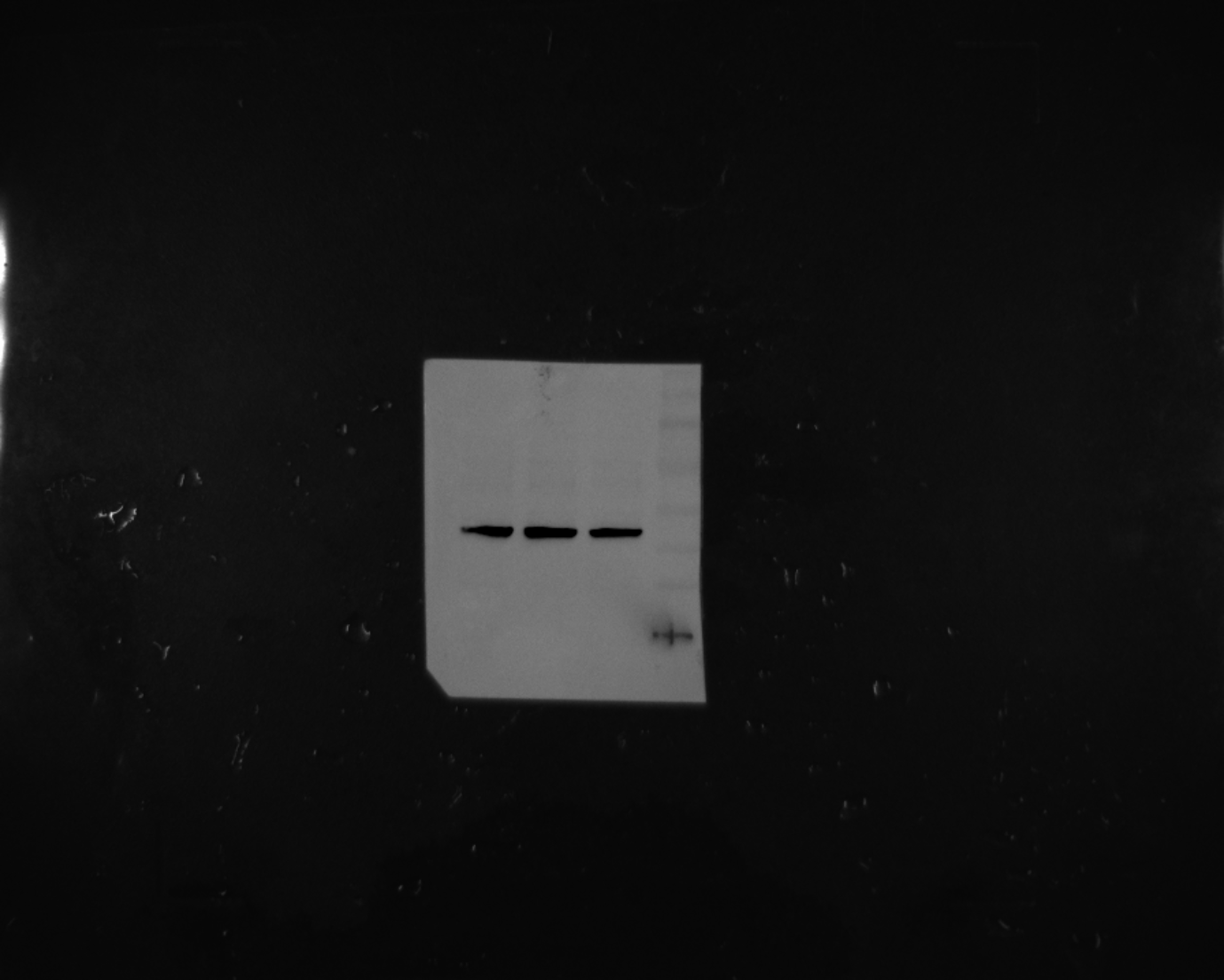

Supplement: Supplementary file 10 — Additional file 10. All Original and uncropped blots images used in manuscript. [file 12915_2022_1437_MOESM10_ESM.zip › blots images/Fig 3/Fig 3 H/Input-beta-actin-marker.tif]

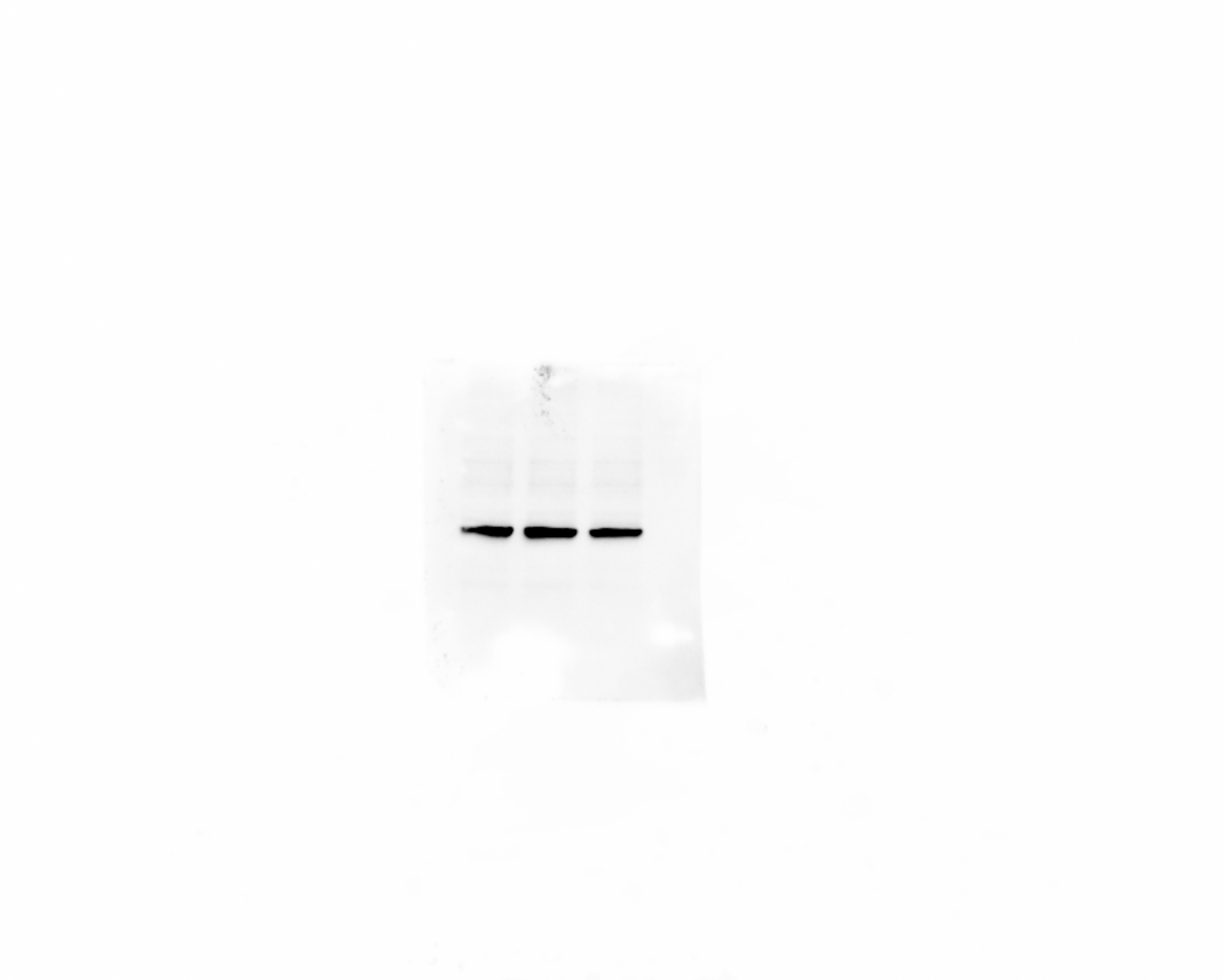

Supplement: Supplementary file 10 — Additional file 10. All Original and uncropped blots images used in manuscript. [file 12915_2022_1437_MOESM10_ESM.zip › blots images/Fig 3/Fig 3 H/Input-beta-actin.tif]

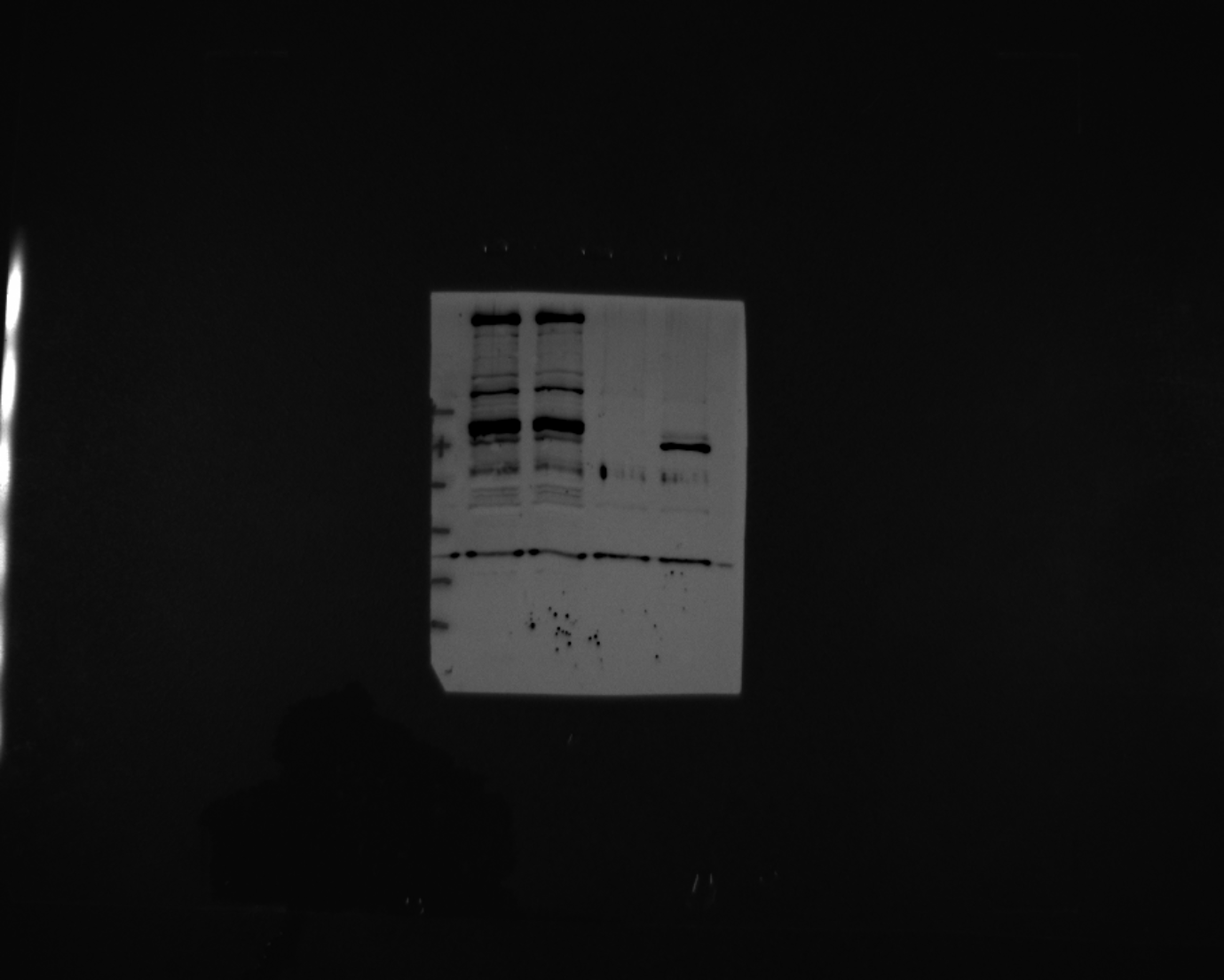

Supplement: Supplementary file 10 — Additional file 10. All Original and uncropped blots images used in manuscript. [file 12915_2022_1437_MOESM10_ESM.zip › blots images/Fig 4/Fig 4 A/HCR-CC3-coIP-CEP72/HCR-CC3-GFP-coIP-CEP72-antiCEP72-marker.tif]

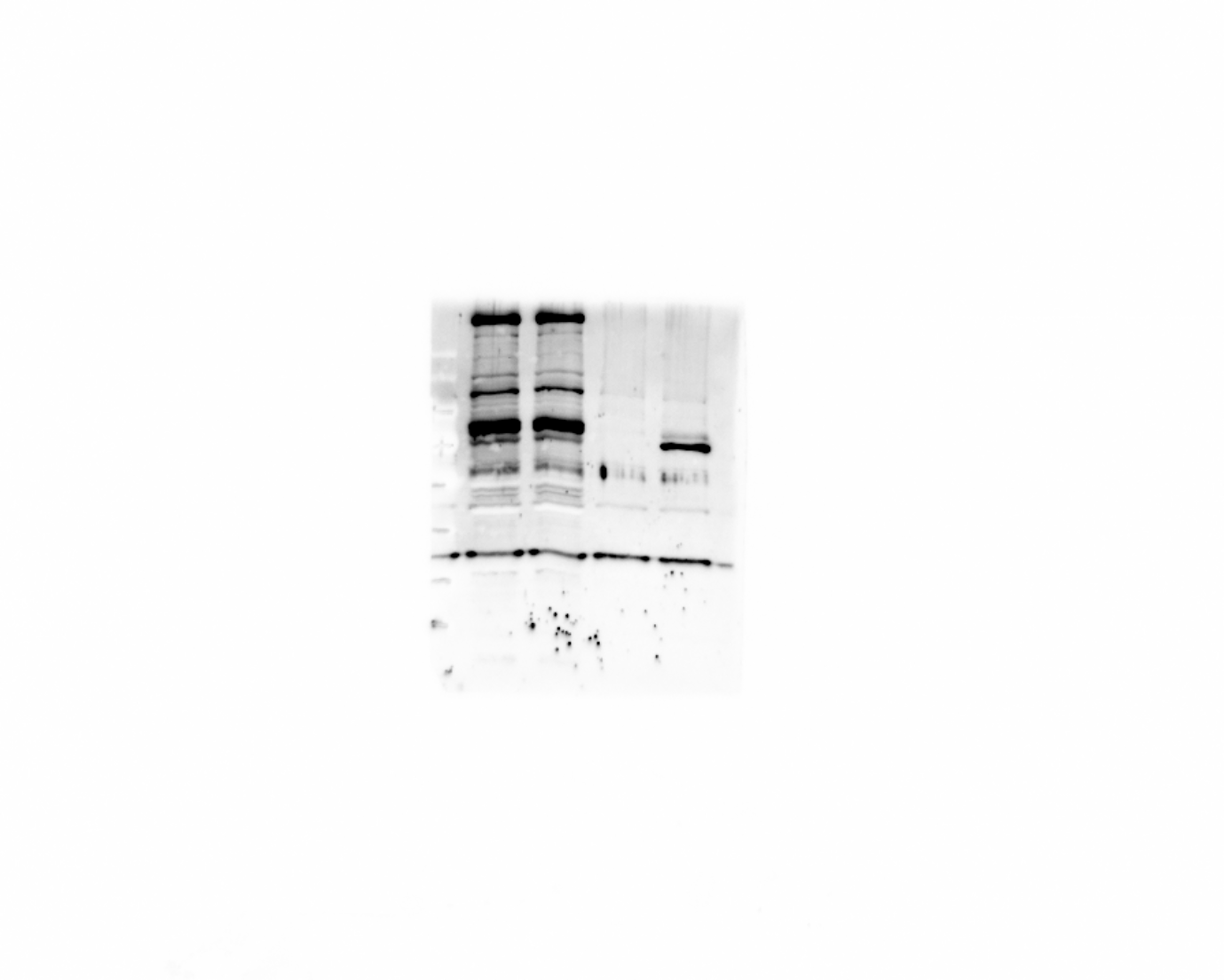

Supplement: Supplementary file 10 — Additional file 10. All Original and uncropped blots images used in manuscript. [file 12915_2022_1437_MOESM10_ESM.zip › blots images/Fig 4/Fig 4 A/HCR-CC3-coIP-CEP72/HCR-CC3-GFP-coIP-CEP72-antiCEP72.tif]

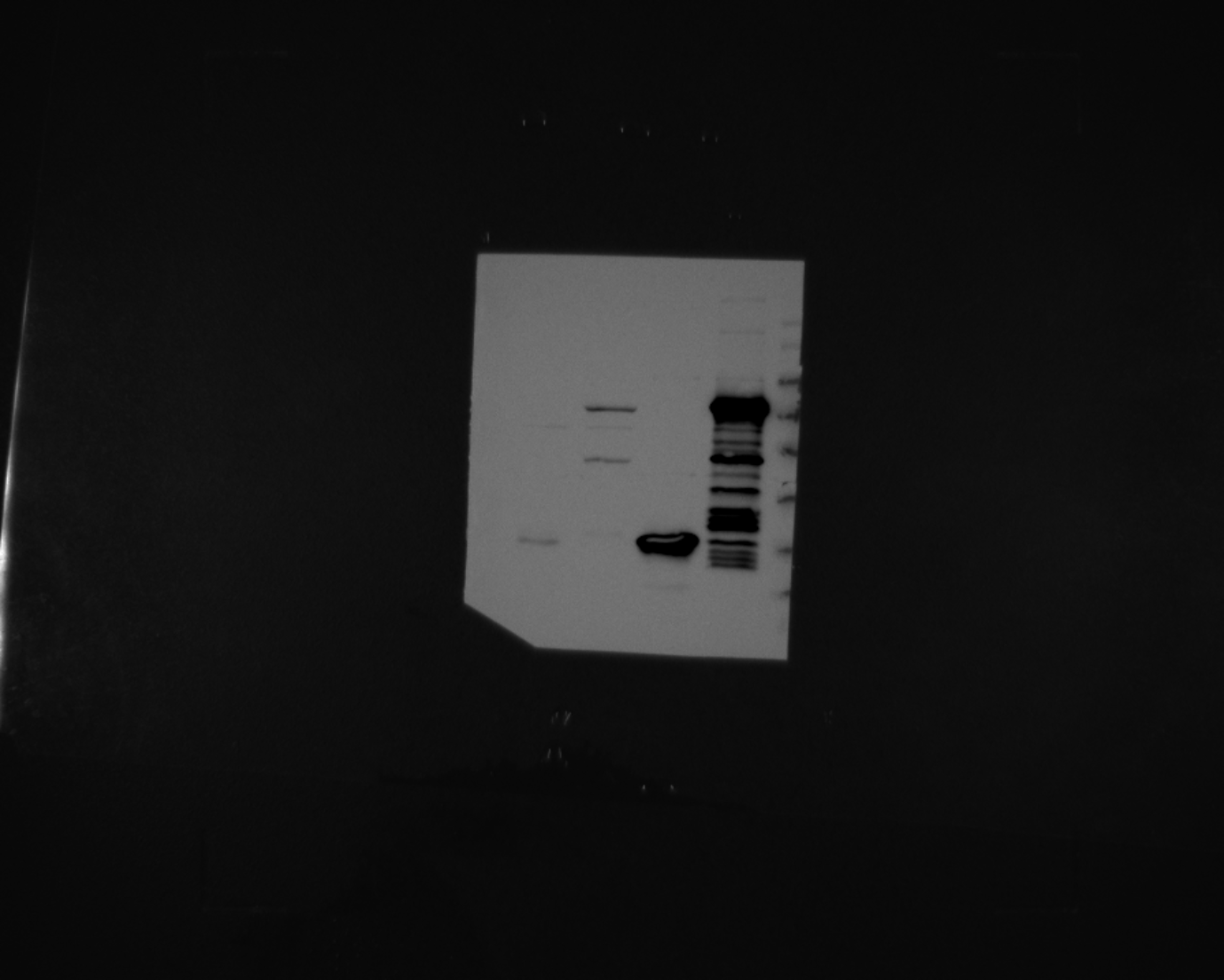

Supplement: Supplementary file 10 — Additional file 10. All Original and uncropped blots images used in manuscript. [file 12915_2022_1437_MOESM10_ESM.zip › blots images/Fig 4/Fig 4 A/HCR-CC3-coIP-CEP72/HCR-CC3-GFP-coIP-CEP72-antiGFP-marker.tif]

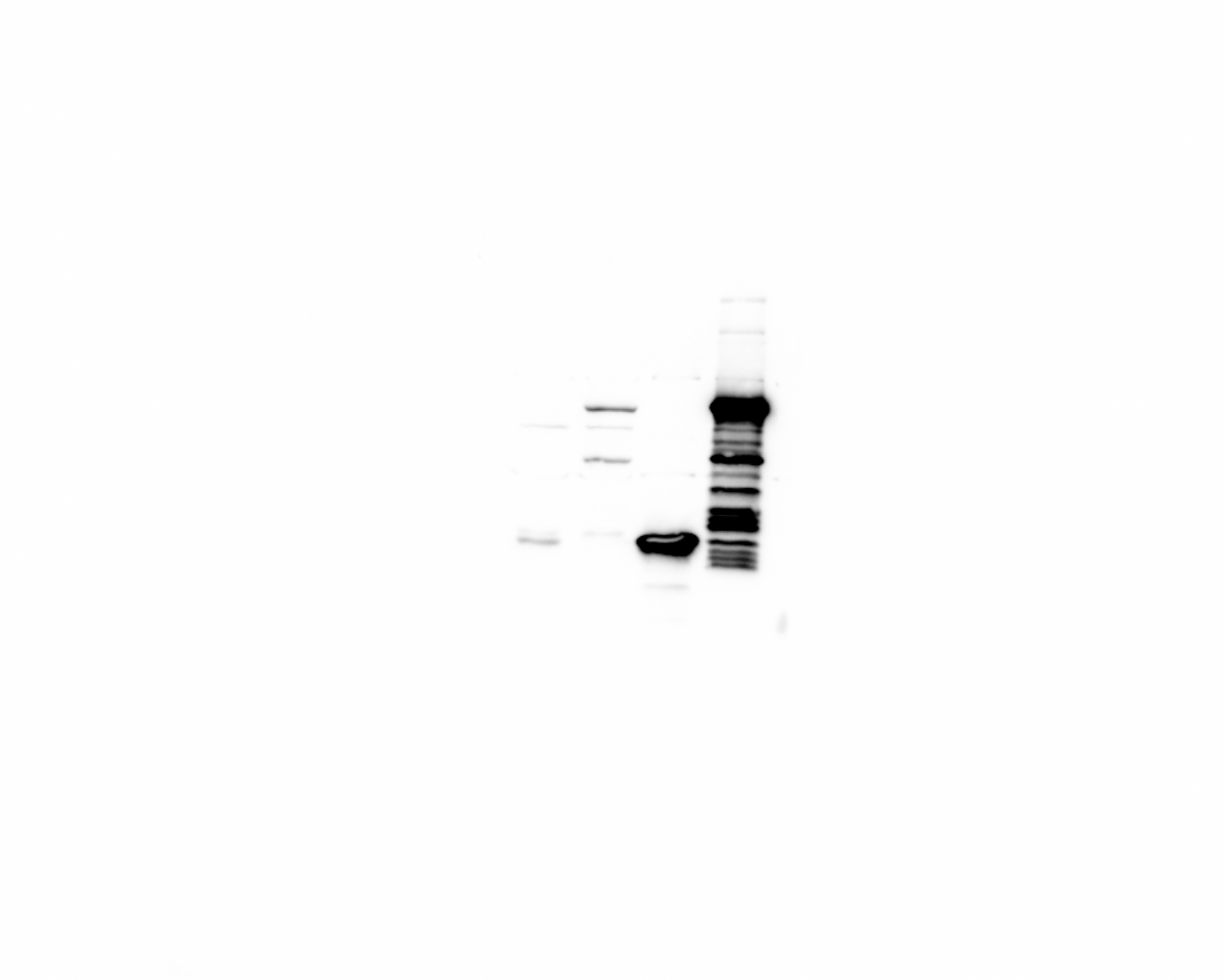

Supplement: Supplementary file 10 — Additional file 10. All Original and uncropped blots images used in manuscript. [file 12915_2022_1437_MOESM10_ESM.zip › blots images/Fig 4/Fig 4 A/HCR-CC3-coIP-CEP72/HCR-CC3-GFP-coIP-CEP72-antiGFP.tif]

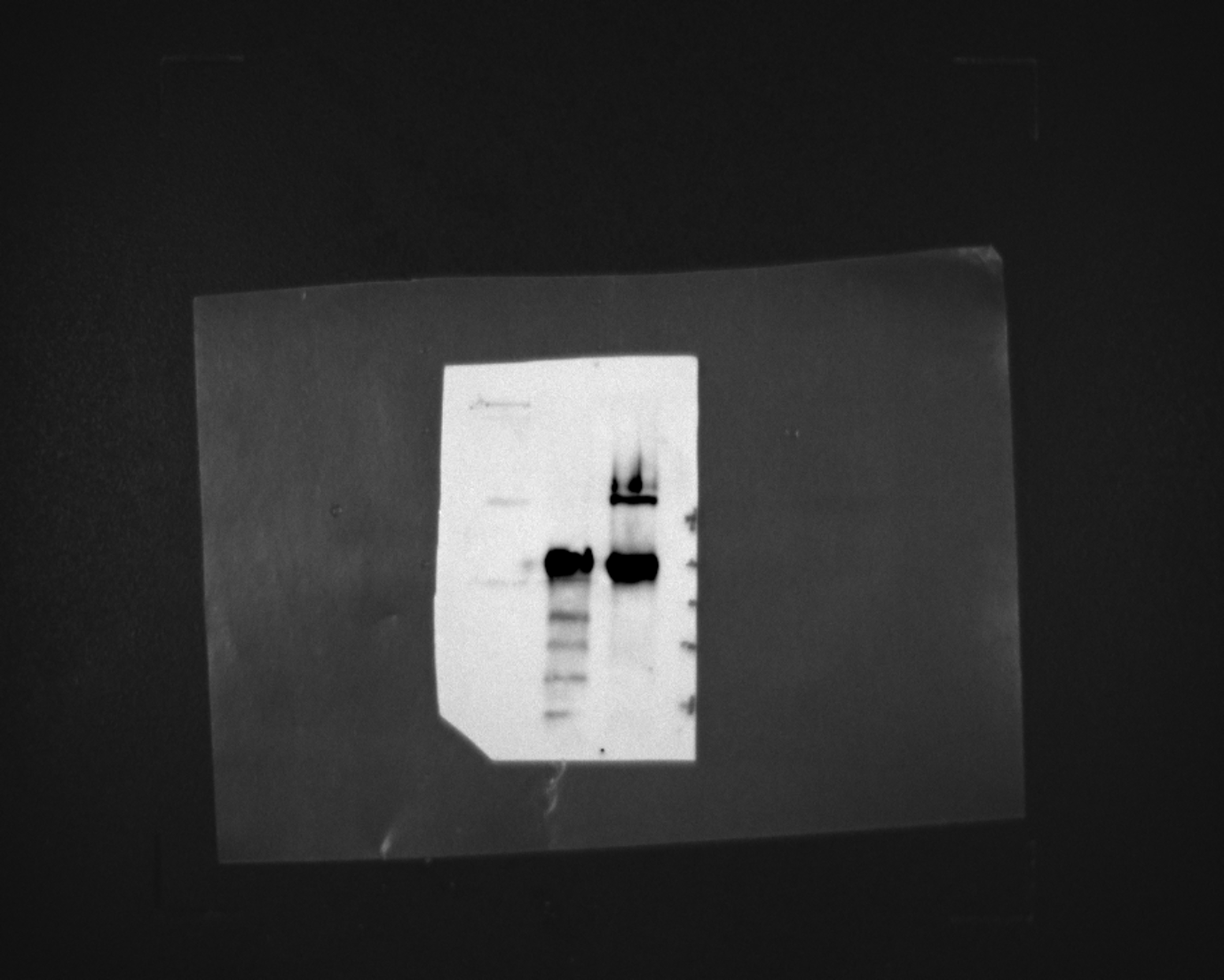

Supplement: Supplementary file 10 — Additional file 10. All Original and uncropped blots images used in manuscript. [file 12915_2022_1437_MOESM10_ESM.zip › blots images/Fig 4/Fig 4 A/IP-CEP72/CEP72-coIP-HCR-anti-CEP72-marker.tif]

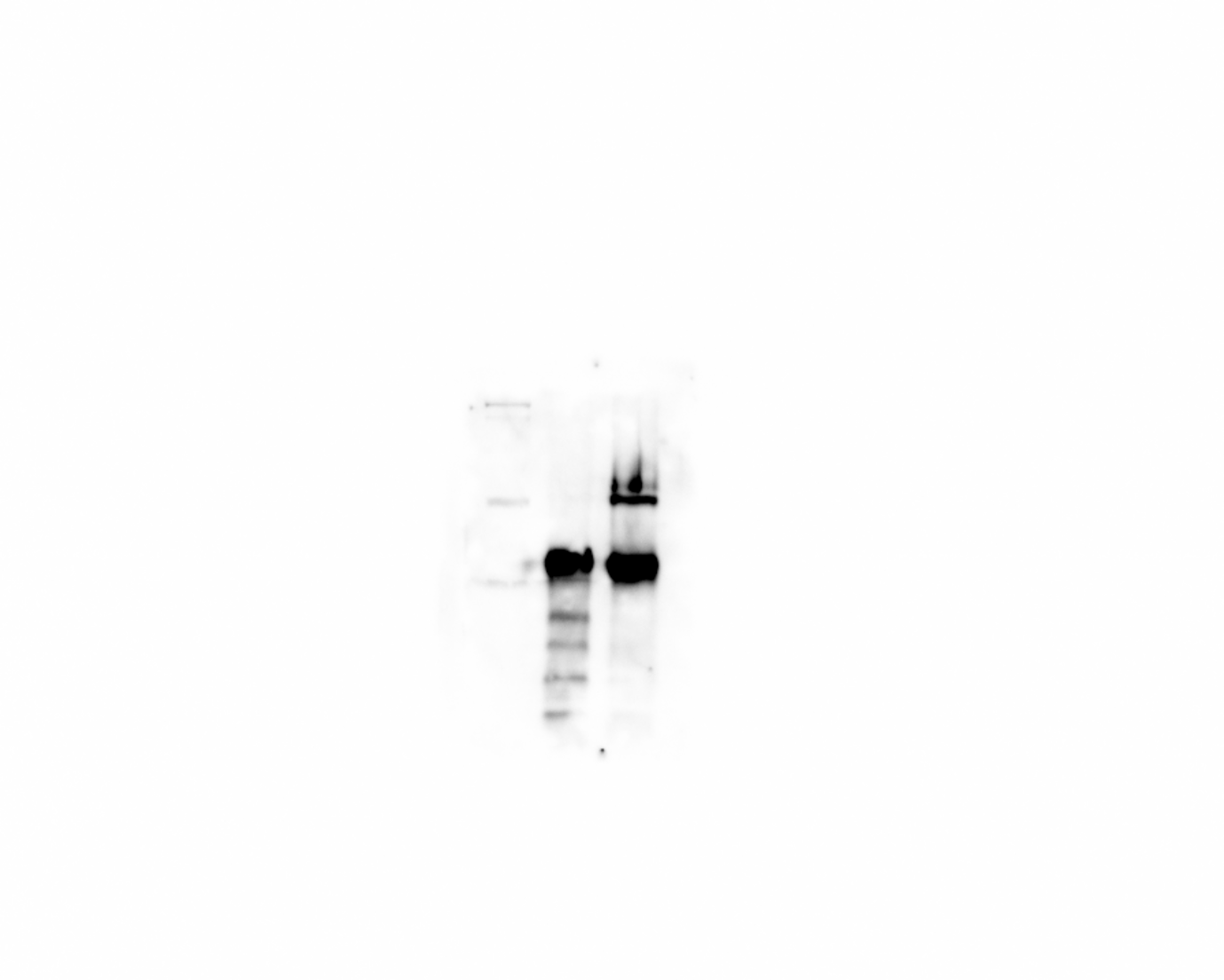

Supplement: Supplementary file 10 — Additional file 10. All Original and uncropped blots images used in manuscript. [file 12915_2022_1437_MOESM10_ESM.zip › blots images/Fig 4/Fig 4 A/IP-CEP72/CEP72-coIP-HCR-anti-CEP72.tif]

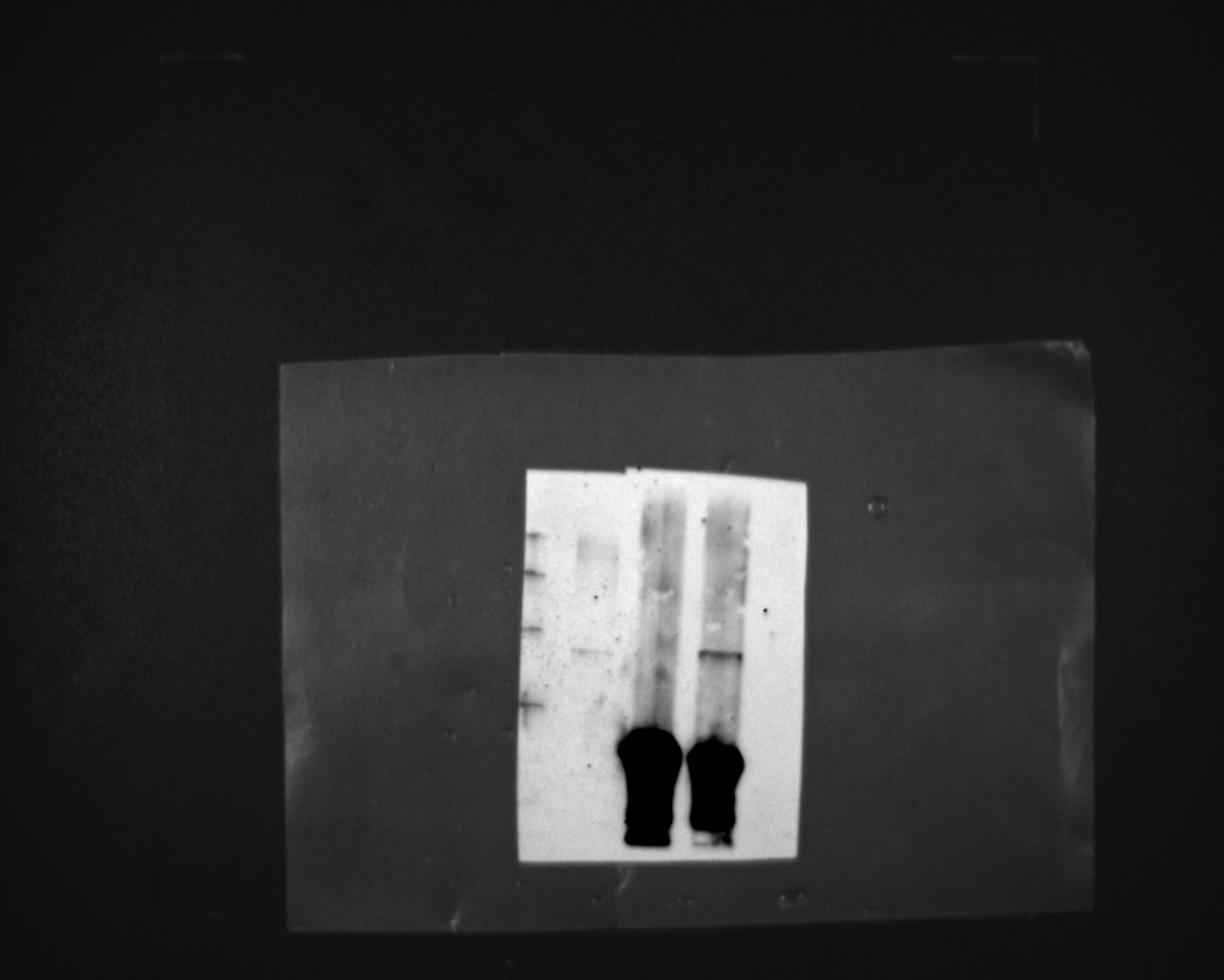

Supplement: Supplementary file 10 — Additional file 10. All Original and uncropped blots images used in manuscript. [file 12915_2022_1437_MOESM10_ESM.zip › blots images/Fig 4/Fig 4 A/IP-CEP72/CEP72-coIP-HCR-anti-HCR-marker.tif]

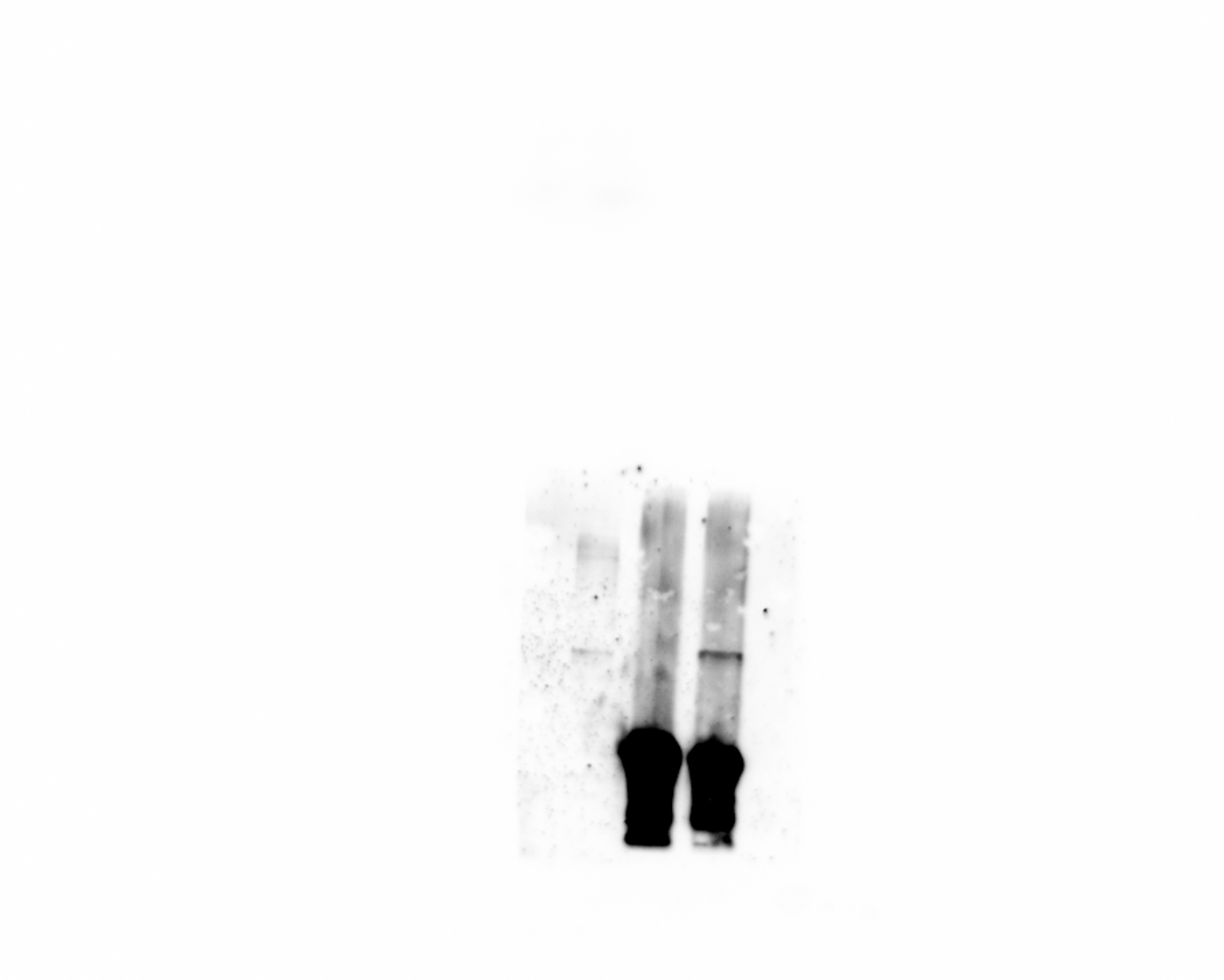

Supplement: Supplementary file 10 — Additional file 10. All Original and uncropped blots images used in manuscript. [file 12915_2022_1437_MOESM10_ESM.zip › blots images/Fig 4/Fig 4 A/IP-CEP72/CEP72-coIP-HCR-anti-HCR.tif]

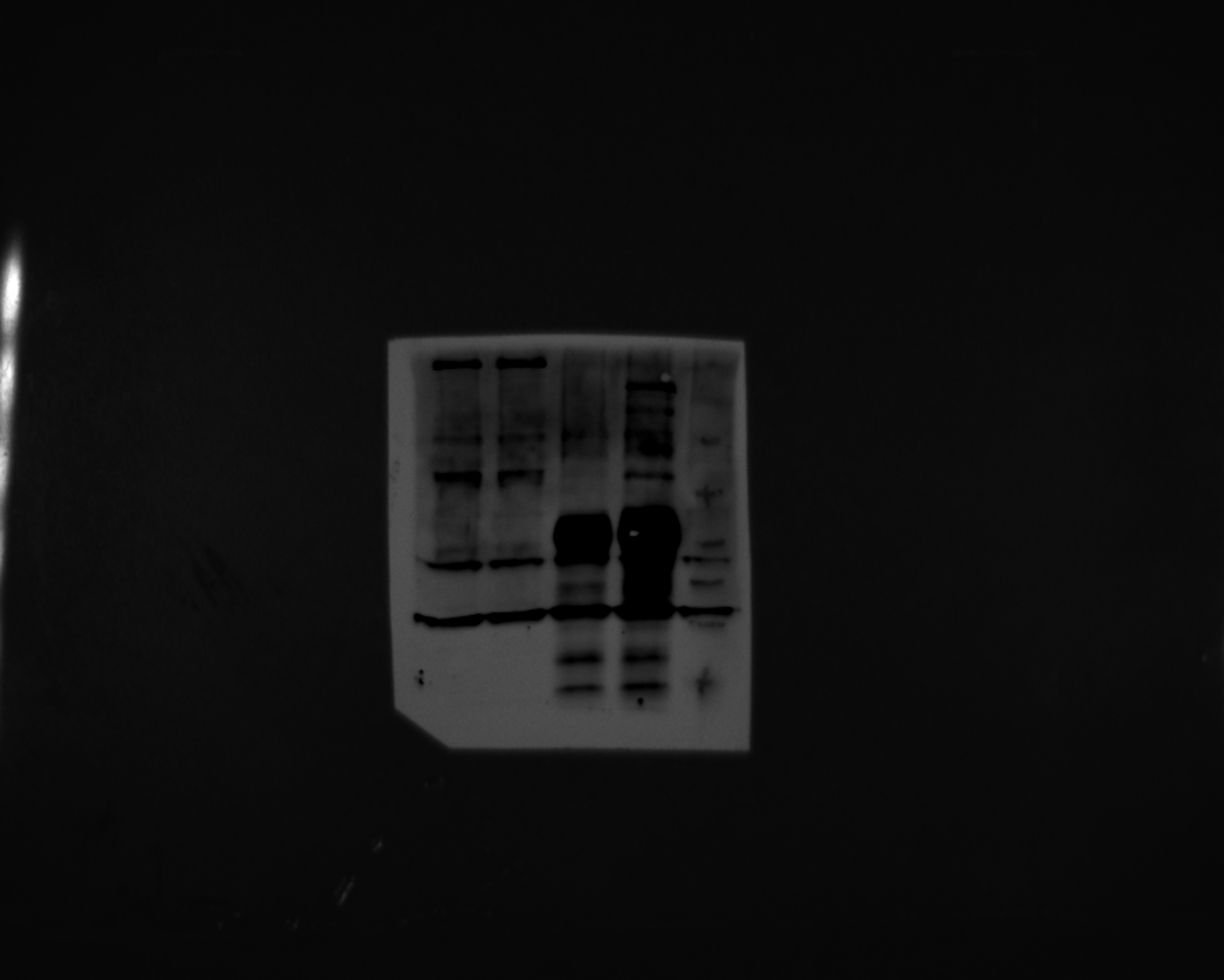

Supplement: Supplementary file 10 — Additional file 10. All Original and uncropped blots images used in manuscript. [file 12915_2022_1437_MOESM10_ESM.zip › blots images/Fig 4/Fig 4 A/IP-HCR/HCR-coIP-CEP72-anti CEP72 with marker.tif]

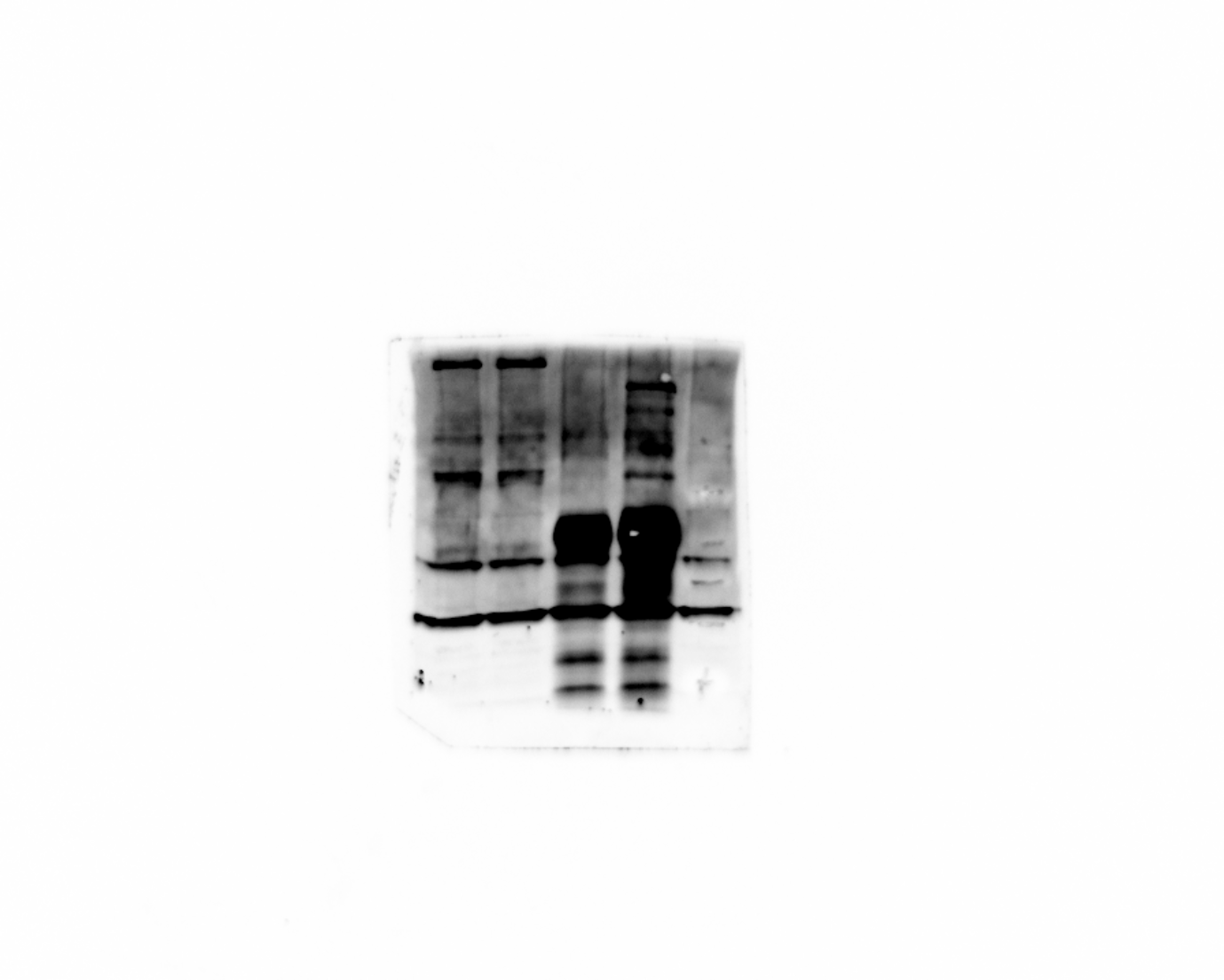

Supplement: Supplementary file 10 — Additional file 10. All Original and uncropped blots images used in manuscript. [file 12915_2022_1437_MOESM10_ESM.zip › blots images/Fig 4/Fig 4 A/IP-HCR/HCR-coIP-CEP72-anti CEP72-first-lane-is-superfluous sampling.tif]

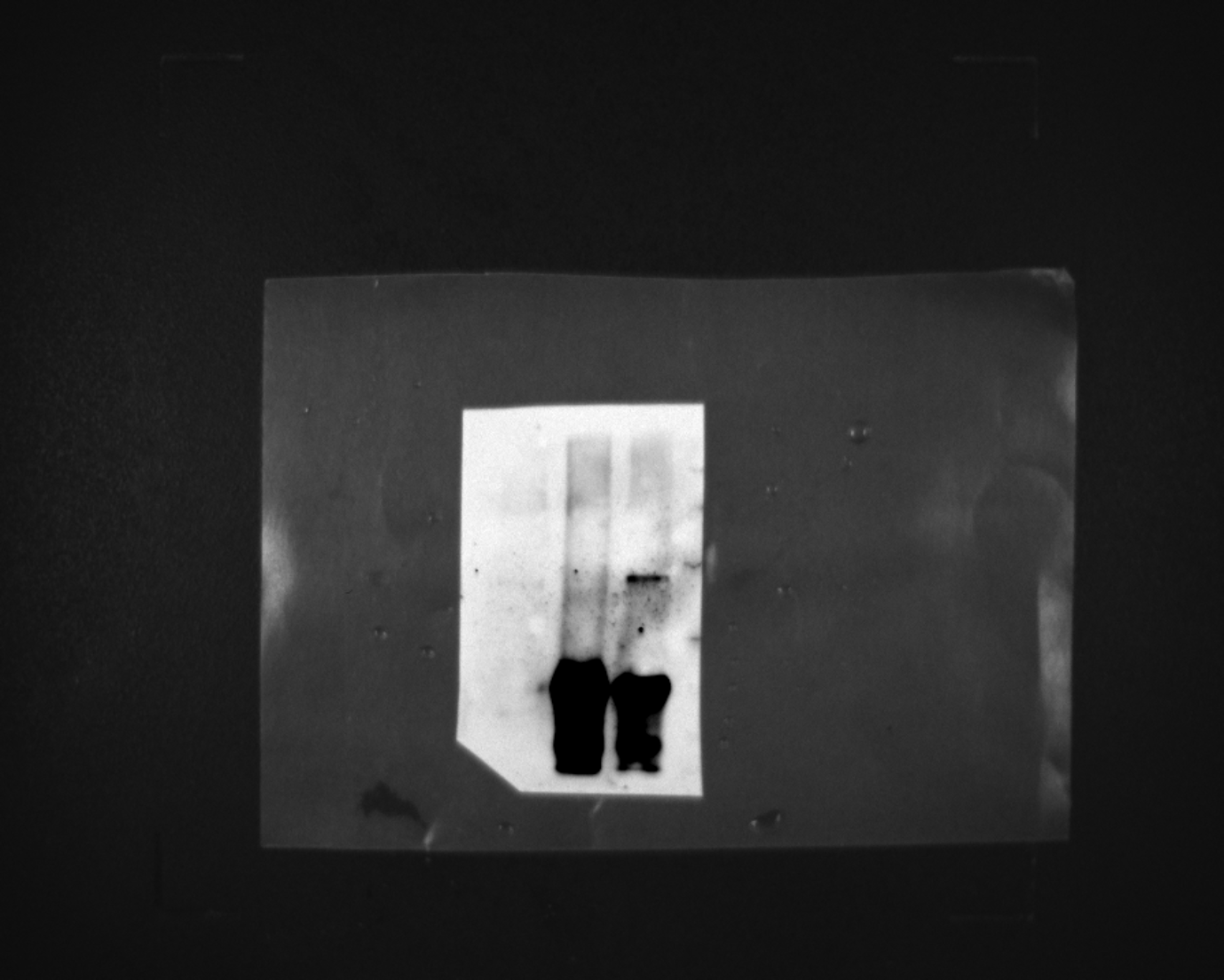

Supplement: Supplementary file 10 — Additional file 10. All Original and uncropped blots images used in manuscript. [file 12915_2022_1437_MOESM10_ESM.zip › blots images/Fig 4/Fig 4 A/IP-HCR/HCR-coIP-CEP72-anti HCR-marker.tif]

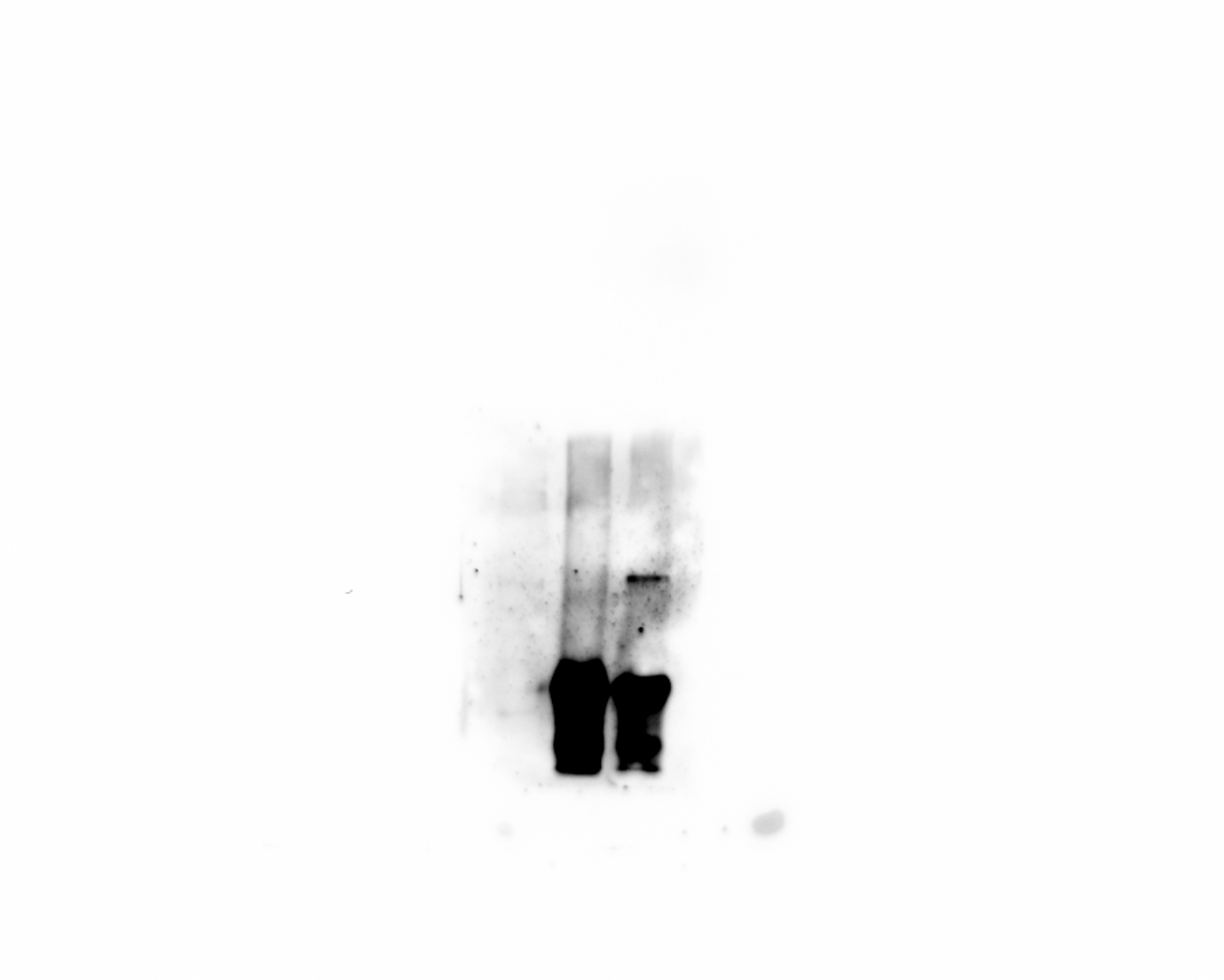

Supplement: Supplementary file 10 — Additional file 10. All Original and uncropped blots images used in manuscript. [file 12915_2022_1437_MOESM10_ESM.zip › blots images/Fig 4/Fig 4 A/IP-HCR/HCR-coIP-CEP72-anti HCR.tif]

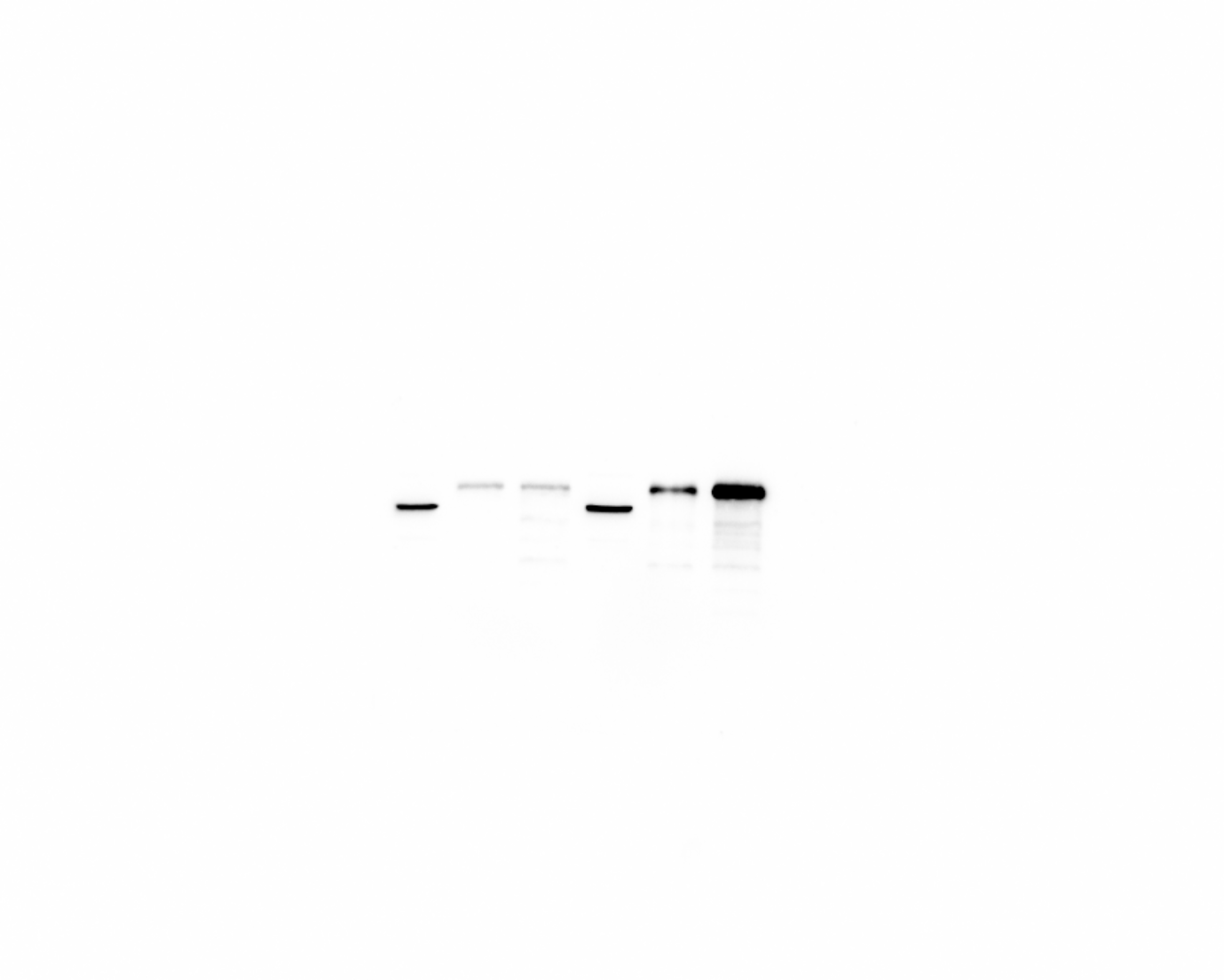

Supplement: Supplementary file 10 — Additional file 10. All Original and uncropped blots images used in manuscript. [file 12915_2022_1437_MOESM10_ESM.zip › blots images/Fig 4/Fig 4 A/left-three-HCR-coip-CEP72-right-three-CEP72-coIP-HCR-anti-beta-actin.tif]

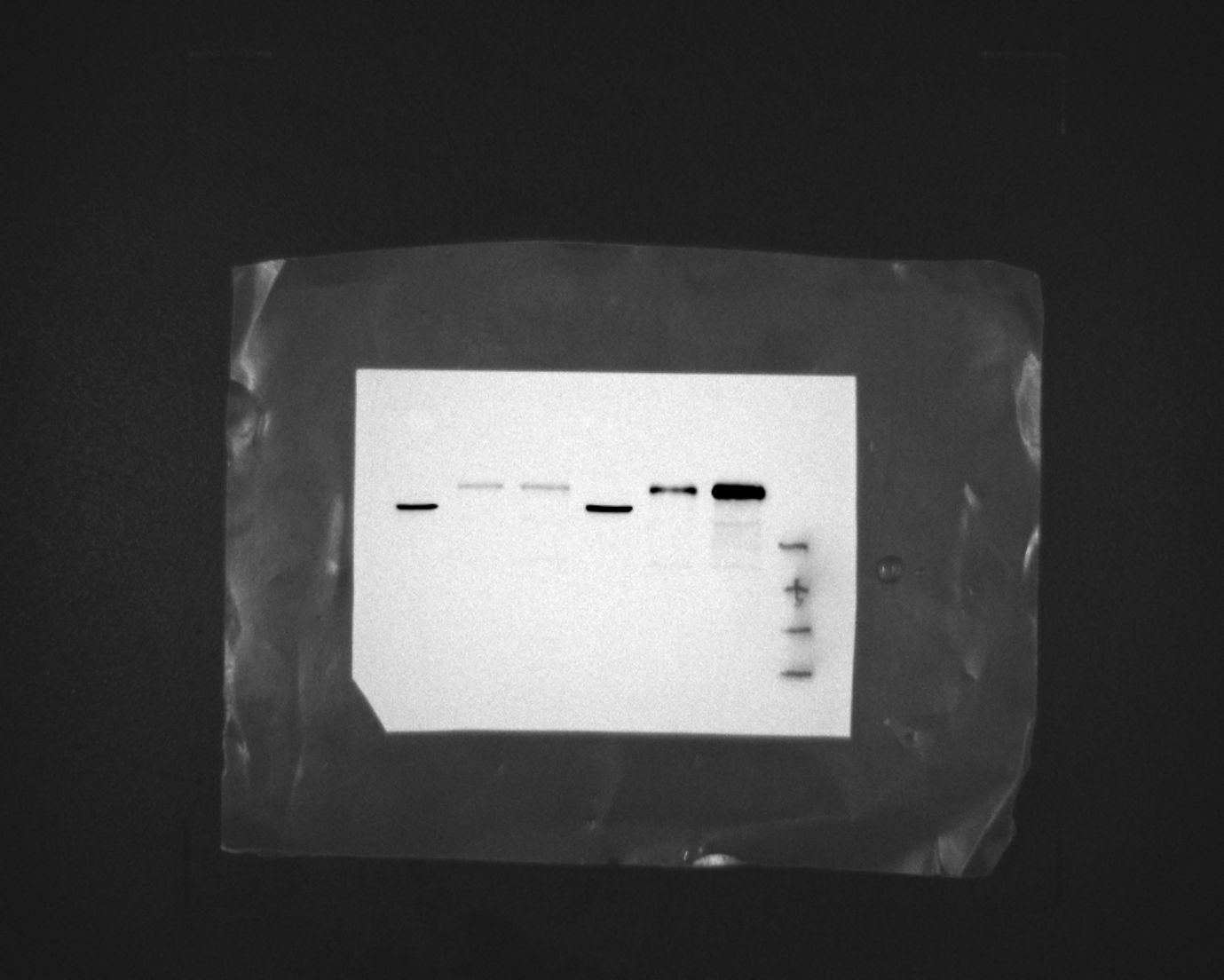

Supplement: Supplementary file 10 — Additional file 10. All Original and uncropped blots images used in manuscript. [file 12915_2022_1437_MOESM10_ESM.zip › blots images/Fig 4/Fig 4 A/left-three-HCR-coip-CEP72-right-three-CEP72-coIP-HCR-anti-beta-actin_marker.tif]

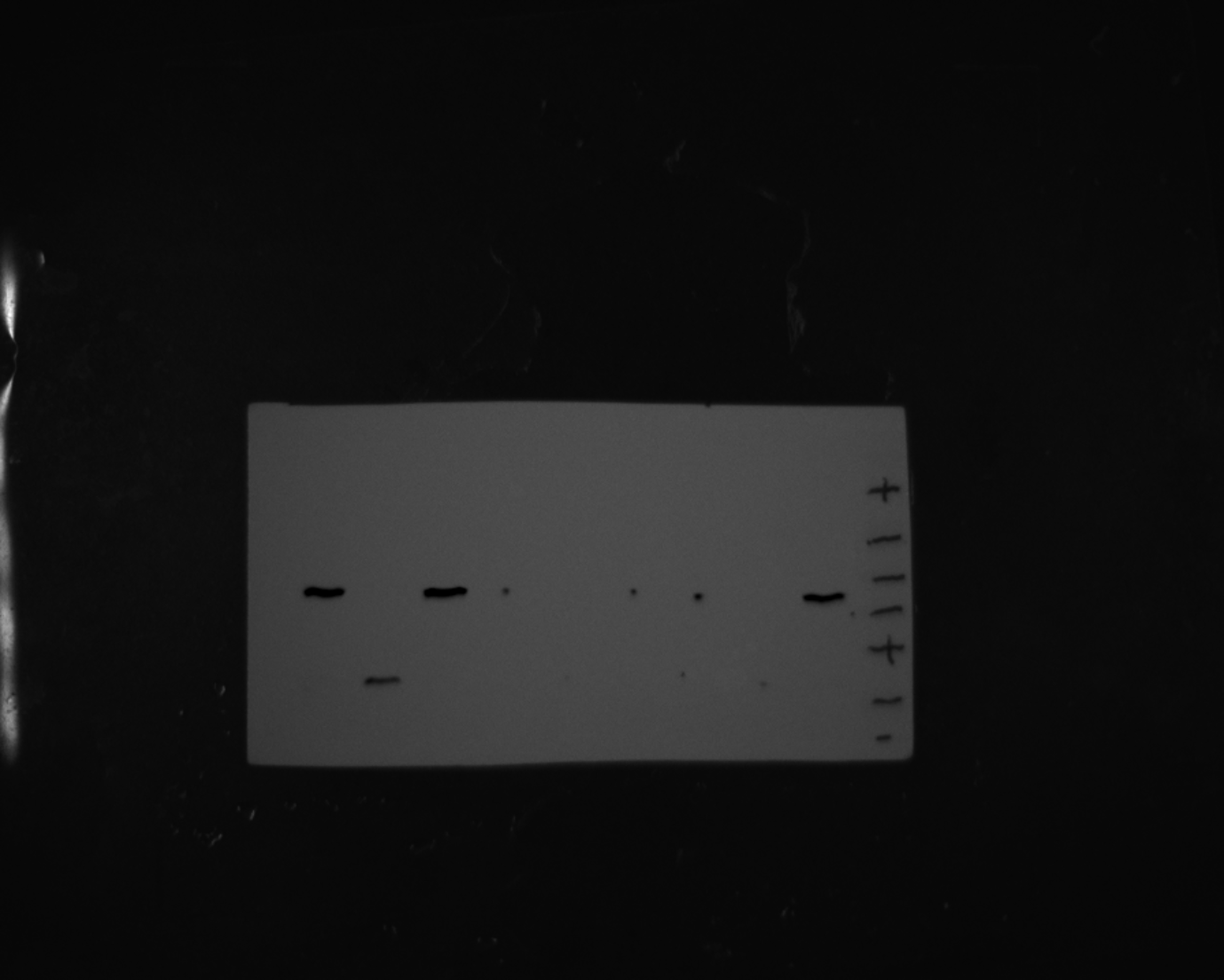

Supplement: Supplementary file 10 — Additional file 10. All Original and uncropped blots images used in manuscript. [file 12915_2022_1437_MOESM10_ESM.zip › blots images/Fig 4/FIg 4 B/Fig4B-GST-pull-down-His-HCR-1-2-3-marker.tif]

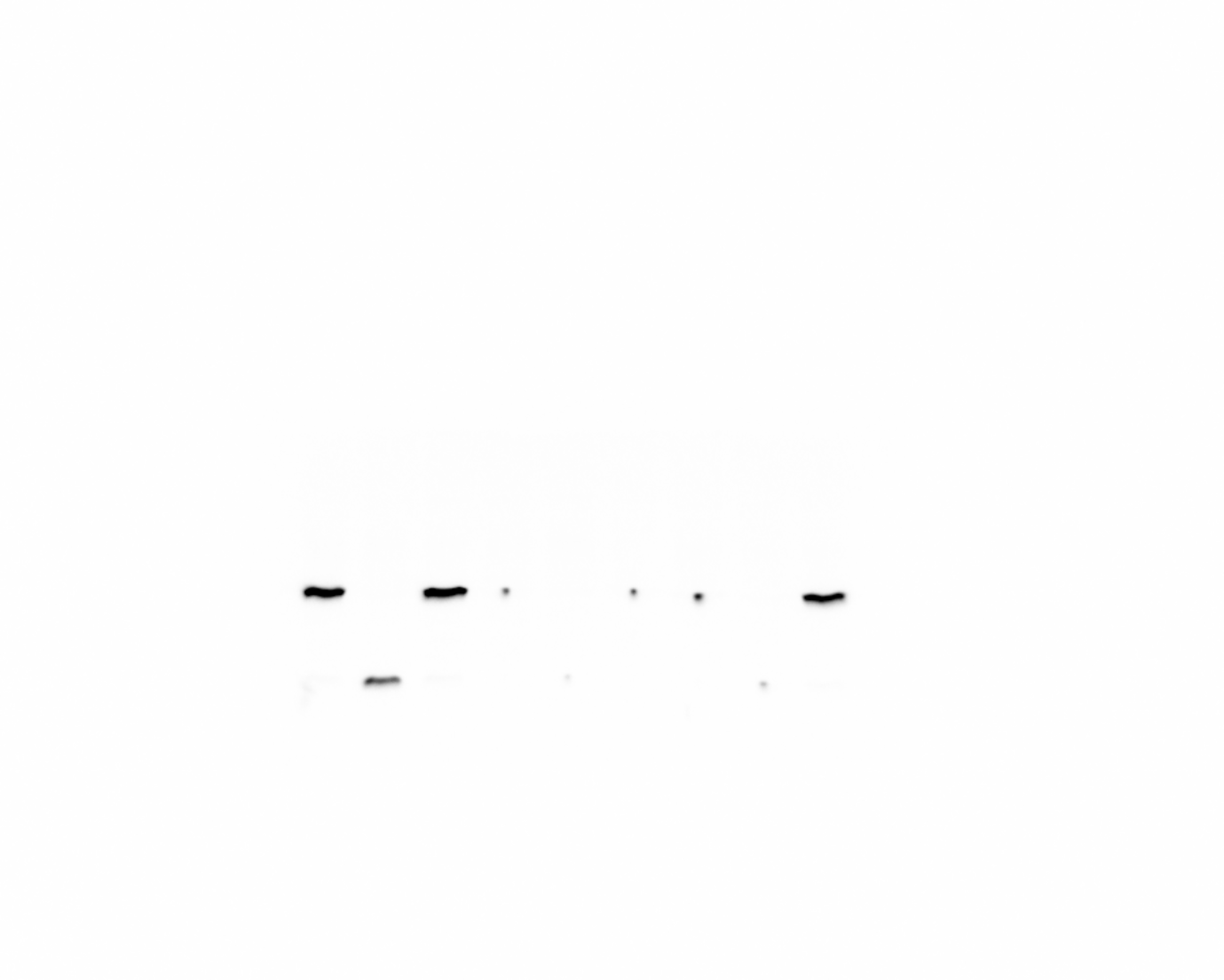

Supplement: Supplementary file 10 — Additional file 10. All Original and uncropped blots images used in manuscript. [file 12915_2022_1437_MOESM10_ESM.zip › blots images/Fig 4/FIg 4 B/Fig4B-GST-pull-down-His-HCR-1-2-3.tif]

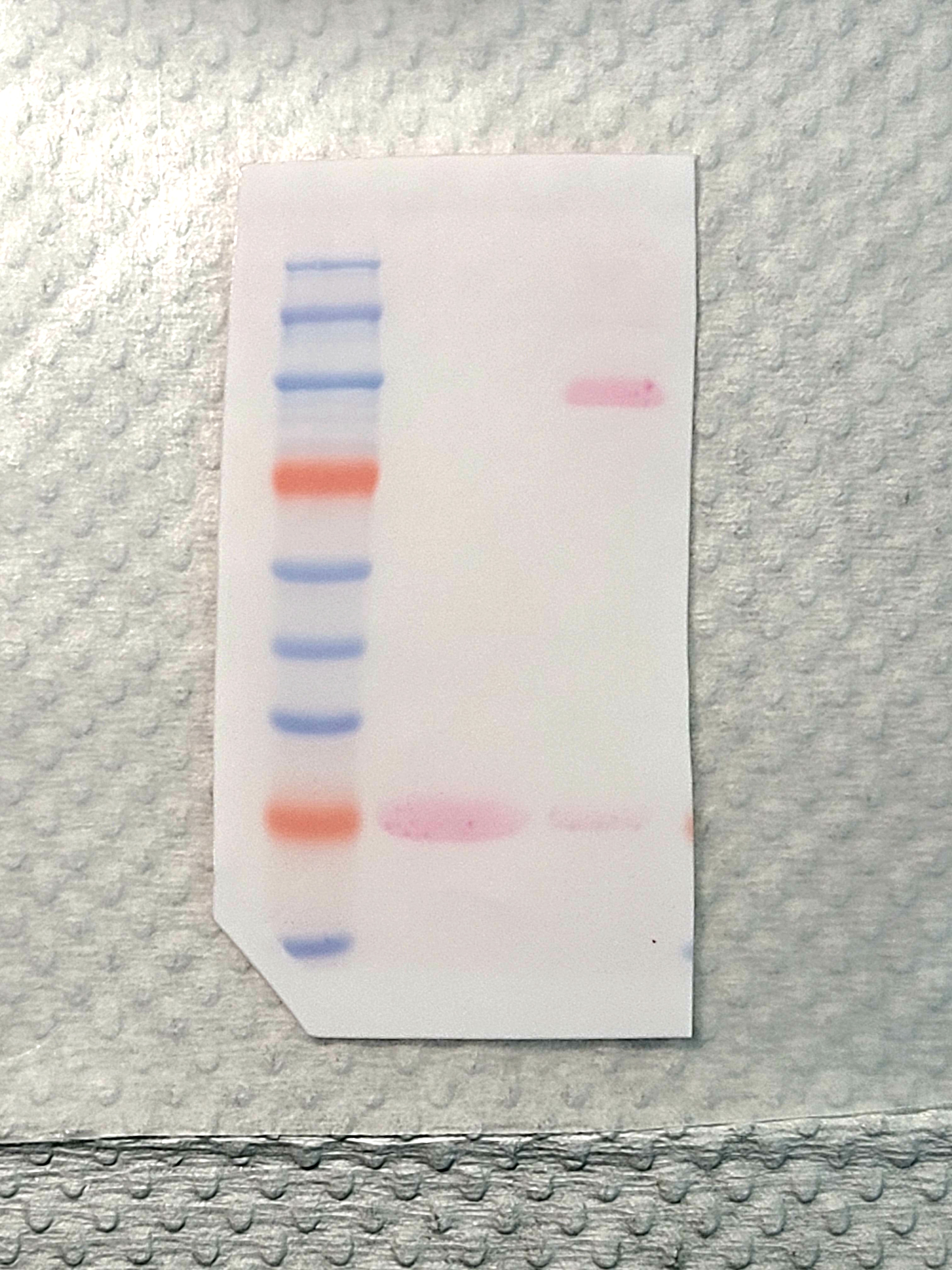

Supplement: Supplementary file 10 — Additional file 10. All Original and uncropped blots images used in manuscript. [file 12915_2022_1437_MOESM10_ESM.zip › blots images/Fig 4/FIg 4 B/GST-CEP72.jpg]

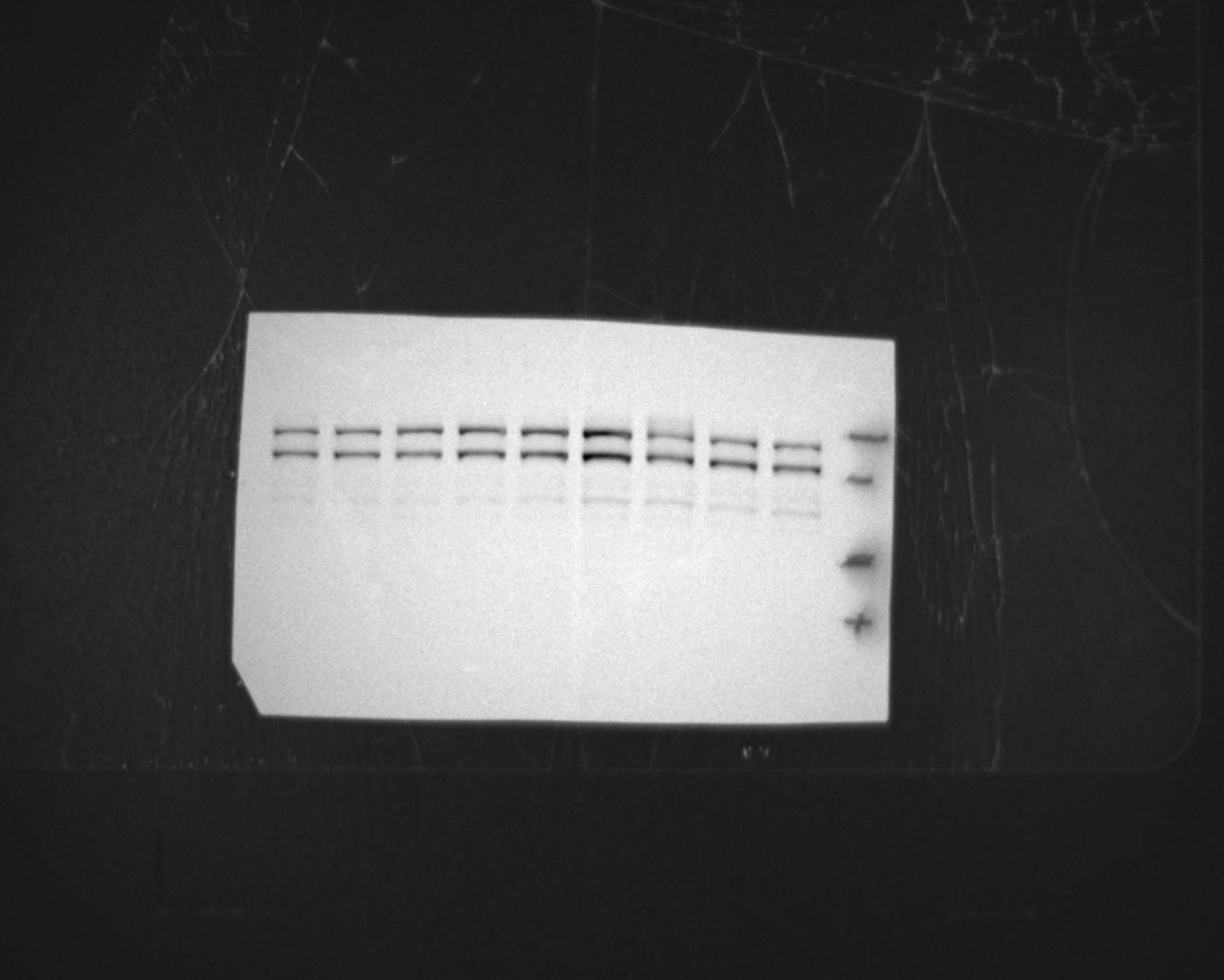

Supplement: Supplementary file 10 — Additional file 10. All Original and uncropped blots images used in manuscript. [file 12915_2022_1437_MOESM10_ESM.zip › blots images/Fig 4/Fig 4 C/astrin/Fig4C-anti-astrin-arker.tif]

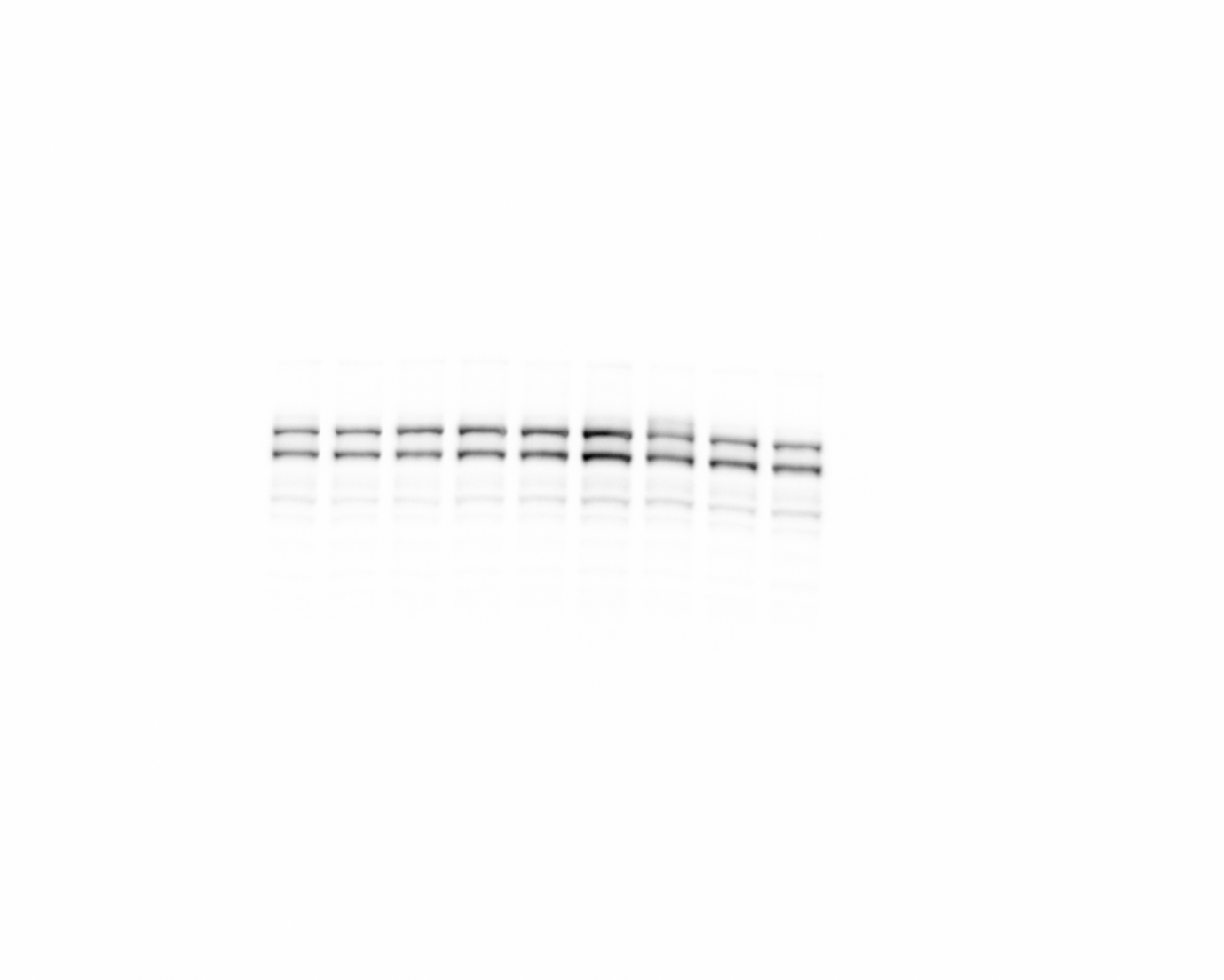

Supplement: Supplementary file 10 — Additional file 10. All Original and uncropped blots images used in manuscript. [file 12915_2022_1437_MOESM10_ESM.zip › blots images/Fig 4/Fig 4 C/astrin/Fig4C-antiastrin.tif]

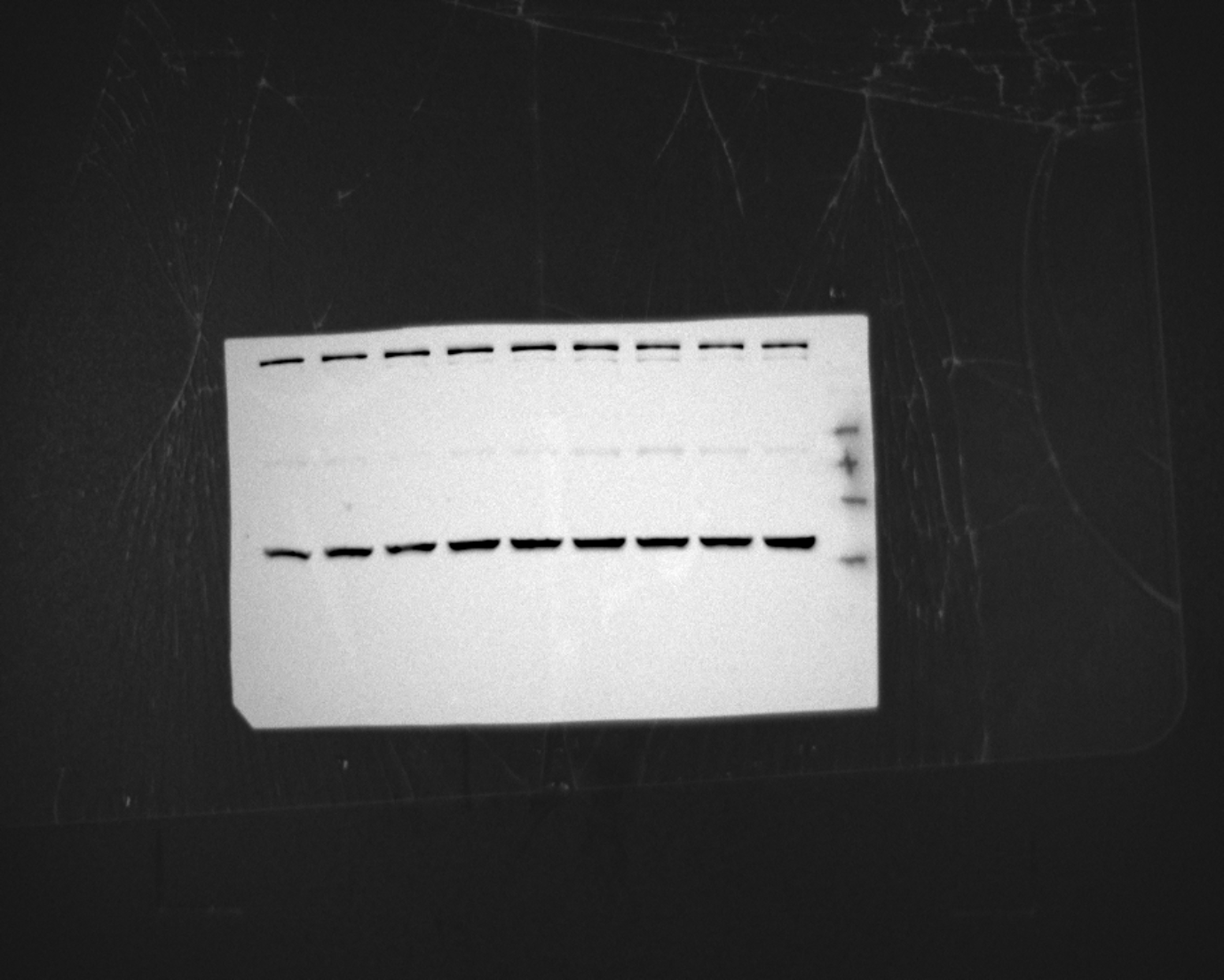

Supplement: Supplementary file 10 — Additional file 10. All Original and uncropped blots images used in manuscript. [file 12915_2022_1437_MOESM10_ESM.zip › blots images/Fig 4/Fig 4 C/beta-actin/Fig4C-beta-actin-marker.tif]

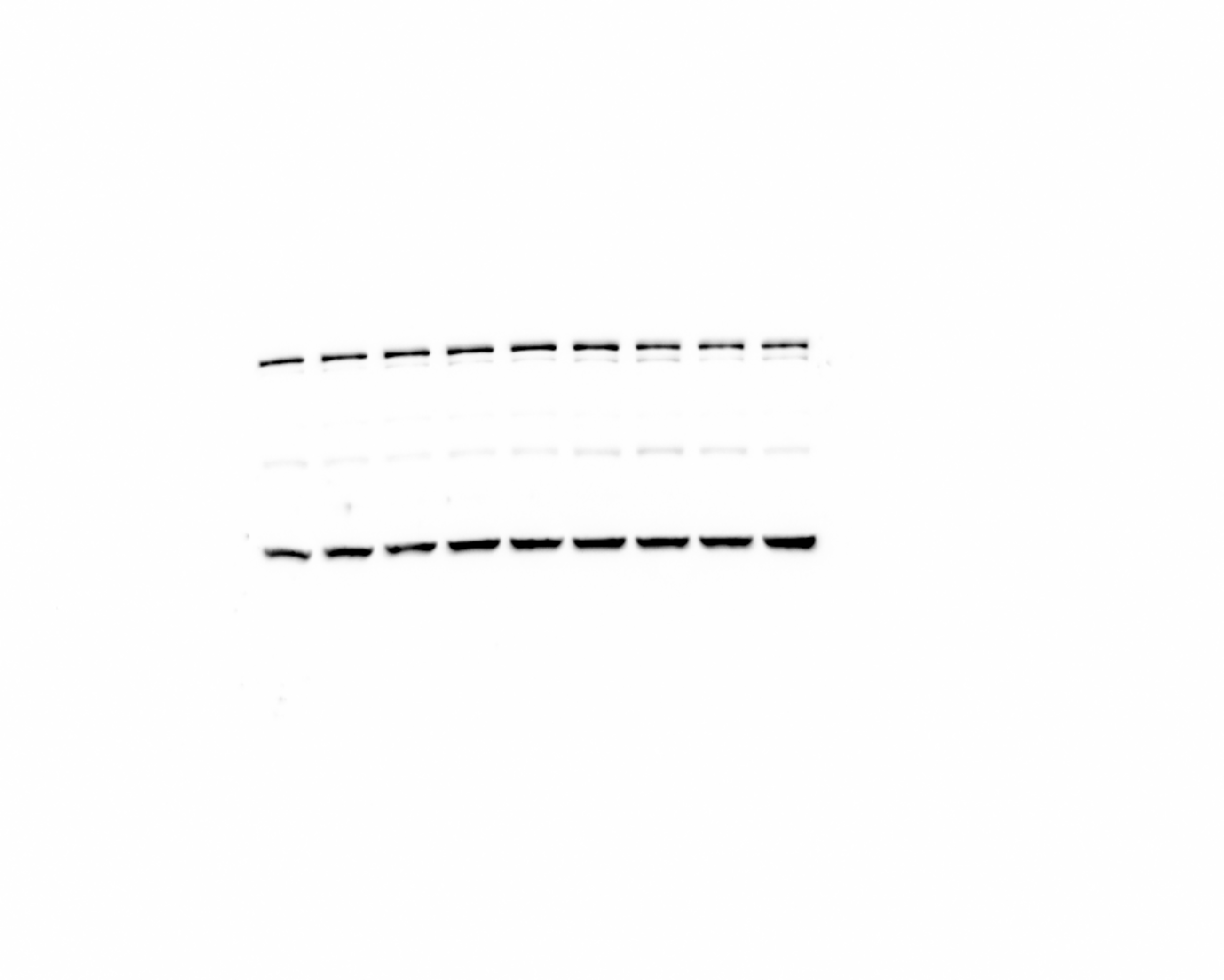

Supplement: Supplementary file 10 — Additional file 10. All Original and uncropped blots images used in manuscript. [file 12915_2022_1437_MOESM10_ESM.zip › blots images/Fig 4/Fig 4 C/beta-actin/Fig4C-beta-actin.tif]
