# Supplementary material for: CCHCR1-astrin interaction promotes centriole duplication through recruitment of CEP72
Source: BMC Biol. 2022 Oct 24;20:240. doi: 10.1186/s12915-022-01437-6 (PMC9590400; doi:10.1186/s12915-022-01437-6)
Supplement: Supplementary file 8 — Additional file 8. Verification reports of HCR-KO cell line. [file 12915_2022_1437_MOESM8_ESM.pdf]

Product brochure

Project information

Knock out the gene name	Human CCHCR1 gene
sgRNA sequence	sgRNA1: CCCGAATGGTGTGGACCTTG<br>sgRNA2: GCGGGAAGAACGGAACCGCC<br>sgRNA3: AACGGGATGTTTCCAGTGAC<br>sgRNA4: TGAGGTTGTCCGGAAGAACT
Cell lines	HELA
PCR primers	PCR-F1： AGGGGAACATAGATGGCTGGAG<br>PCR-R1: CTCATCCCTAGCACTTAGCACA<br>PCR-F2: ATGTTCCACGATCTCAGCCAGAC<br>PCR-R2: TTCACCAAAGGTCTCATCACTC<br>PCR-R3: CAGACGGGGCATATCAGCA
specification	Human CCHCR1 gene knockout HELA monoclonal (heterozygote) cell line: 2 *10^6 2 branches
Mycoplasma testing	feminine

Cell white light map	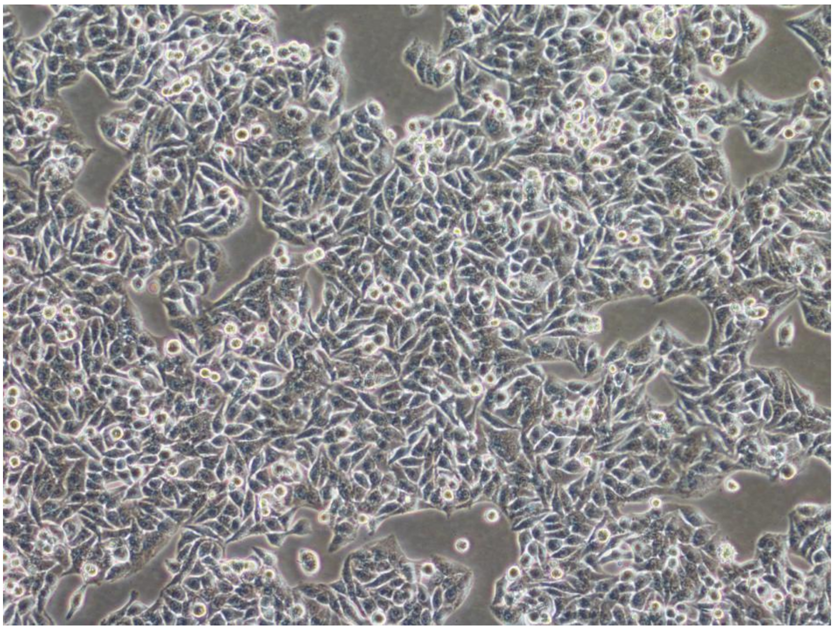

Experimental steps

1. GRNA vector construction

We designed the sgRNA sequence for the CCHCR1 gene, constructing the sgRNA sequence into an sgRNA vector with the spCas9 protein gene and the purinemycin resistance gene;

2. Seed the cells of interest

Inoculate about 4×105 cells to 2 Petri dishes respectively, divided into experimental group and control group;

3. Plasmid transfection

2 μg of endotoxin DNA transfection of experimental group cells; The control group did not do any transfection;

4. Antibiotics screen cells

After transfection 48h, the experimental group added puromycin to screen positive cells with a final concentration of 3 μg/ml, and the control group also added the same concentration of puromycin;

5. Cell enrichment

After 48 h of antibiotic screening, the cells in the control group basically died, the cells in the experimental group partially survived, and the cells in the experimental group continued to be cultured.

6. Collect cell pull genomes

To be cultured to a certain number of cells in the experimental group, part of the cells are collected to extract the genome;

7. Hybrid clone PCR sequencing verification

We designed PCR primers at approximately 500 bp each upstream and downstream of the gRNA target location, amplified the DNA of interest using the extracted genome as a template, and sequenced to confirm the knockout effect;

8. Single cloning screening and verification

The mixed clones with knockout effect were screened by limited dilution method, and part of the cell pull genome was collected after the monoclonal cells were cultured to a certain number, and the target strip was expanded by PCR for glue running identification and sequencing to verify the knockout effect.

Results of the monoclonal 4 experiment

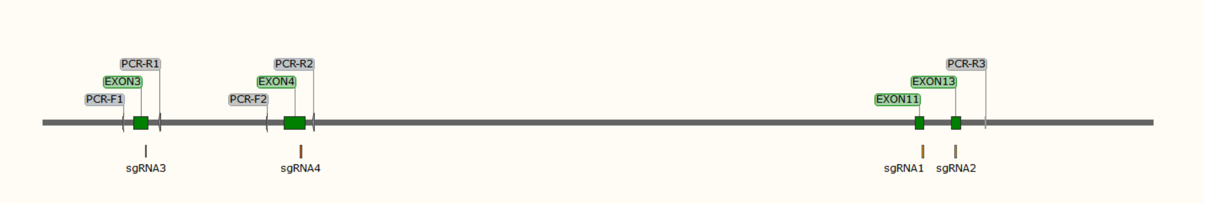

The figure above is a map of the position of 4 gRNA and PCR primers in the genome

We mixed 4 gRNAs (sgRNA1-4) with knockout effects into HELA cells, theoretically all 4 gRNAs will be cut on the genome, and we can theoretically get cell lines that knock out large fragments between exon3-exon13. We divide the verification method into two steps:

Validation one: We used the primers PCR-F1 and PCR-R3 to verify whether the cell line knocked out large fragments, if after knocking out part of the fragment between exon3-exon13, the primers PCR-F1 and PCR-R3 are less than 1500bp apart on the genome, so we use the primers PCR-F1 and PCR-R3 to amplify the knockout cell line genome, under the set PCR amplification conditions, When a single band below 1500 can be amplified, it means that the cell line has been knocked out of some fragments between exon3-exon13, and if the amplification does not expand the band, it is not knocked out, and we sequence the PCR product to verify.

Validation two: Identify knockout of exon4 with primers PCR-F1 and PCR-R1 (538bp expandable), knockout of exen3, pcR-F2 and PCR-R2 (684bp expandable), thereby verifying whether the cell line is a homozygote that knocks out large fragments. If PCR-F1 and PCR-R1, PCR-F2 and PCR-R2 cannot amplify the band, it means that the cell line is a homozygote that knocks out the fragment between exon3-exon13, and if the band can be amplified, it means that it is a heterozygote, and we sequence the PCR product to verify.

Verify a result as shown in the following table:

<table>
<tr><td colspan="4">First, the results of electrophoresis</td></tr>
<tr><td rowspan="2">Analysis and interpretation of results</td><td>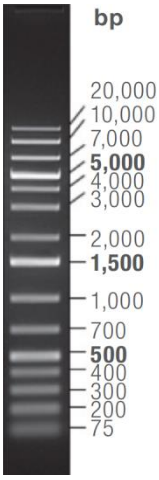
</td><td>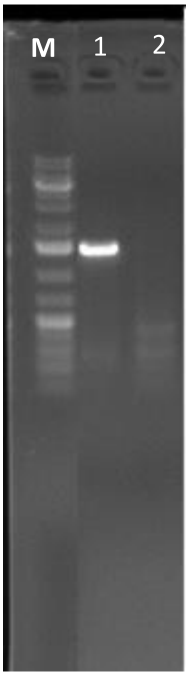
</td><td>No. 1 is a monoclonal cell
No. 2 is the control group without transfection of cells</td></tr>
<tr><td colspan="3">The above figure shows the results of the monoclonal cell genome and the control cell genome after PCR, and the PCR conditions we set are theoretically that only the cell genome that knocks out the large fragment between exon3 and exon13 can amplify the band, the single clone obtains a single band, and the control cell does not get the band, indicating that the cell line knocks out the large fragment between exon3 and exon13</td></tr>
<tr><td colspan="4">Second, the sequencing results</td></tr>
</table>

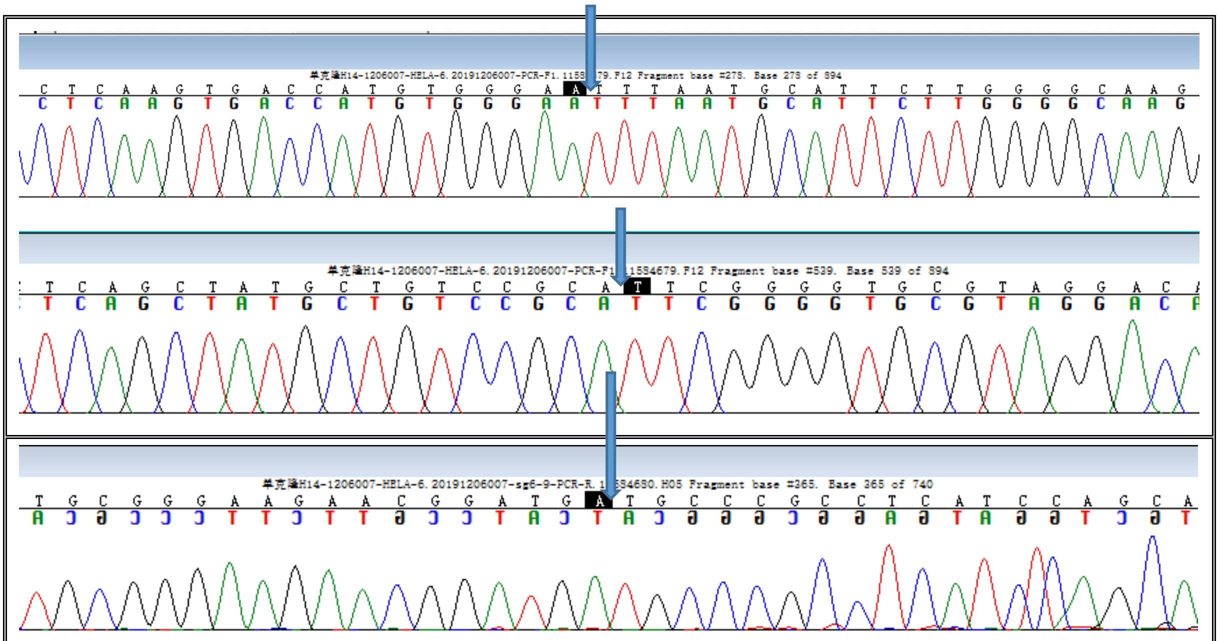

The above figure is the location of each gRNA knockout, and the sequencing peak diagram at both ends of the knockout position is single, indicating that the cell is a CCHCR1 gene knockout monoclonal cell line.

3. Knock out the sequence

CcHCR1 **gene cDNA partial sequence (marked red is knockout part, green is the mutant base).**

TCGGCCCCTTTCAACTCTGCCAAGAATGGCTCCCACCTGGCTCTCAGACATTCCCCTGGTCCAACCCCCAGGCCAT

CAAGATGTCTCAGAGAGGCGGCTAGACACCCAGAGACCTCAAGTGACCATGTGGGA

ACGGGATGTTTCCAGTGACAGGCAGGAGCCAGGGCGGAGAGGCAGGTCCTGGGGGCTGGAGGGGTCACAGGC

CCTGAGCCAGCAGGCTGAGGTGATCGTTCGGCAGCTGCAAGAGCTGCGGCGGCTGGAGGAGGAGGTCCGGCTC

CTGCGGGAGACCTCGCTGCAGCAGAAGATGAGGCTAGAGGCCCAGGCCATGGAGCTAGAGGCT

CTGGCACGGGCGGAGAAGGCCGGCCGAGCTGAGGCTGAGGGCCTGCGTGCTGCTTTGGCTGGGGCTGAGGTTG

TCCGGAAGAACTTGGAAGAGGGGAGCCAGCGGGAGCTGGAAGAGGTTCAGAGGCTGCACCAAGAGCAGCTGT

CCTCTTTGACACAGGCTCACGAGGAGGCTCTTTCCAGTTTGACCAGCAAGGCTGAGGGCTTGG

AGAAGTCTCTGAGTAGTCTGGAAACCAGAAGAGCAGGGGAAGCCAAGGAGCTGGCCGAGGCTCAGAGGGAGG

CCGAGCTGCTTCGGAAGCAGCTGAGCAAGACCCAGGAAGACTTGGAGGCTCAGGTGACCCTGGTTGAGAATCTA

AGAAAATATGTTGGGGAACAAGTGCCTTCTGAGGTCCACAGCCAGACATGGGAACTGGAGCG

ACAGAAGCTTCTGGAAACCATGCAGCACTTGCAG<br>GAGGACCGGGACAGCCTGCATGCCACCGCGGAGCTGCTGCAGGTGCGGGTGCAGAGCCTCACACACATCCTCG<br>CCCTGCAGGAGGAGGAGCTGACCAGGAAGGTTCAACCTTCAGATTCCCTGGAGCCTGAGTTTACCAGGAAGTGC<br>CAGTCCCTGCTGAACCGCTGGCGGGAGAAGGTGTTTGCCCTCATGGTGCAGCTAAAGGCCC<br>AGGAGCTGGAACACAGTGACTCTGTTAAGCAGCTGAAGGGACAGGTGGCCTCACTCCAGGAAAAAGTGACATCC<br>CAGAGCCAGGAGCAGGCCATCCTGCAGCGATCCCTGCAGGACAAAGCCGCAGAGGTGGAGGTGGAGCGTATGG<br>GTGCCAAGGGCCTGCAGTTGGAGCTGAGCCGTGCTCAGGAGGCCAGGCGTCGGTGGCAGCA<br>GCAGACAGCCTCAGCCGAGGAGCAGCTGAGGCTTGTGGTCAATGCTGTCAGCAGCTCTCAGATCTGGCTCGAGA<br>CCACCATGGCTAAGGTGGAAGGGGCTGCCGCCCAGCTTCCCAGCCTCAACAACCGACTCAGCTATGCTGTCCGCA<br>AGGTCCACACCATTCGGGGCCTGATTGCTCGAAAGCTTGCCCTTGCTCAGCTGCGCCAGGAGAGCTGTCCCCTAC<br>CACCACCGGTCACAGACGTGAGCCTTGAGTTGCAGCAGTTGCGGGAAGAACGGATGA<br>GCCTGGATGCAGAACTGCAGCTGAG<br>TGCCCGCCTCATCCAGCAGGAGGTGGGCCGGGCTCGGGAGCAAGGGGAGGCAGAGCGGCAGCAGCTGAGCAA<br>GGTGGCCCAGCAGCTGGAGCAGGAGCTGCAGCAGACCCAGGAGTCCCTGGCTAGCTTGGGGCTGCAGCTGGAG<br>GTAGCACGCCAGGGCCAGCAGGAGAGCACAGAGGAGGCTGCCAGTCTGCGGCAGGAGCTGACC<br>CAGCAGCAGGAACTCTACGGGCAAGCCCTGCAAGAAAAGG
CCHCR1 **genome partial sequence (marked in red as knockout part) (complete knockout of exon4-exon10 of NM_001105564.1, knockout of exon3, exon11, exon13 part).**<br>GAGCCTTCAAATAATGTGGAGATGTTTCCACCTTCAGGTCAGTGGGACCAGACAAGGGGAGTGGTGGTTGTCTC<br>TGCCTGGGAAACTGACCATCTTTGTTTTTGTAATTCTTCAGGTTCCACTGGGCTGATTCCCCCCTCCCACTTTCAAG<br>CTCGGCCCCTTTCAACTCTGCCAAGAATGGCTCCCAC<br>CTGGCTCTCAGACATTCCCCTGGTCCAACCCCCAGGCCATCAAGATGTCTCAGAGAGGCGGCTAGACACCCAGAG<br>ACCTCAAGTGACCATGTGGGAA<br>CGGGATGTTTCCAGTGACAGGCAGGAGCCAGGGCGGAGAGGCAGGTAGGGATCCATCCACGCCGTTTTCTCAG<br>GCTTGCTTGCTAGTGACCCTTCCTCACTGGAATAAACTCCTTACCCTATTTCGGCCCTAGTTTCCAGAACGTACTCA<br>TTTTTATGTAAGCAATTAGTTCCTACAGTGCATAAATAATAAAGCAGTGTGCTAAGTG<br>CTAGGGATGAGGATAACAATAGTGAATAAGACAAATCCTGACTTAAATTTGTAATTAATAAGGAATCTAAAATCC<br>TAAGAGGGGAGATAGATTGACCACCAGCTGATGGTAAAGCATGGTATGAGGCAAGATGAAGAAGTGCAAACTA

GAGGTAGAGACCCAGCACTGTGGCAGTGTGGGTTCATTCTGATGGAAGAAAGCCTTTACA

GAGGACATGCTGTGGGAGCTGGCCATGCTGTCGTATTATCTGTAAACATTCTTATTCTTGTTTCATGTGTCTTTTCC

CAGATGTACTGGGATATCATTCTGCCATTTTGCTCCAGAATAGGATTACATTAGAAGAAGCAGCATGATGAAGAA

GGAATACTAGACTGGGTGTCAGGAGACAGAGACTCTATTTCTAGTTTGGCTACCAG

CTAGAGGACATTTGGCAAGTCACTTAATTTCCCTAGCCTACAGATCGCTGCTACTAAAGAAAGAAAGAAAGAGCG

AGCCAGATATGGCGGCTCACACCTATAATCCTAGCACTTTGGGAGCTGAGGCAGGAGGATCACTTGAGCCCAGG

AGTTCATGACCAGCGTGGGCAACAAAGCGAGACTCCGTCTCCACAGAAAATAATTAGCT

GGCTGTGGTGGCATGCATTTGTAGTCCTAGCTACTCAGGAGGCTAAGGTGGTAGGACCCCTTGAGCTCAGAAGT

TGAAGACTGCAGTGACCTATGATCCAGCCACTGCATTGAGGCCTGGGTGACACAGTGATACCCTGTCTCTAAAAA

CGACAACAACAACAATCTCTTATAGTCCTGGGTCTCAGAGAGCTGCCTCAGGAGCCATG

TTCCAAGCTGGATTAAACTTCACGTGACATTGGTAGACGATTTCTCTAAAGGCTGGCACTGTGTTATTTATGTACT

GTTCTCTCAGACTCCTCATAAAGTATACCTAACACTCAATAAATGCCTTTTTTTTGAGATGGAGTCTCACTCTGTCG

CCCAGGCTGGAGTG

CACTGGGGAGATCTTGGCTCACTGCAAGCTCTGCCTCCCAGGTTGACGCCATTCTCCTGCCTCAGCCTCCCAAGCA

GCTGGGACTACAGGCACCTGCCACCATGCCCAGCTAGTTTTTTATATTTTTAGTAGAGATGGGGTTTCACCGTGTT

AACCAGGATGATCTCGATCTCCTGACCTCGTGATCCGCCCGCCTCGGCCTCCCAAA

GTGCTGGGATTACAGGCATGAGCCACCGCGCCCAGCCAATAAATGCCTTTTAACTAGCACCTGGCCTCACCATAT

TGATACTGGAAGCTTACGACCTCTCTATGCCCATTCCTCCCTCAAACTTCTTGCCCTTAAATTAGAATTGAGAAGTC

CCTGTGTGTTCTTCCAACCTTTCCACTTAAAATGTGTGGCCTAAAAATAAAAAAAT

AAATAAGACAAAAAAACCACCAAAAAACAAAAAGAATGTGTGGCCTAATGATATACACATTTGATGTTGAATCCT

CCTGTATGTAGCTCTCTCTAGGGAGATCATGTTCCACGATCTCAGCCAGACTTTAGGTCCTGTGAGTCCAGGCACT

GGACTCAACATGCTCAATAGGGCTTTGATGAATGATGATGATGTCAATGCAGACATC

CCCATACCCCAGCTTCAGCACCCCCTTCACCTCCCCACACGGAAGCAGAGGGGTCCTCTTTTCCTTCTCCTGGCTAT

GTTTATGCCCTCAACTATCCTTCCAGCACTGGAGACAAGTCTCACCTGCACTAACCTGTCTTTGAAGGTCCTGGGG

GCTGGAGGGGTCACAGGCCCTGAGCCAGCAGGCTGAGGTGATCGTTCGGCAGCTG

CAAGAGCTGCGGCGGCTGGAGGAGGAGGTCCGGCTCCTGCGGGAGACCTCGCTGCAGCAGAAGATGAGGCTA

GAGGCCCAGGCCATGGAGCTAGAGGCTCTGGCACGGGCGGAGAAGGCCGGCCGAGCTGAGGCTGAGGGCCTG

CGTGCTGCTTTGGCTGGGGCTGAGGTTGTCCGGAAGAACTTGGAAGAGGGGAGCCAGCGGGAGC

TGGAAGAGGTTCAGAGGCTGCACCAAGAGCAGGTGAATGCAGGGGTAGAAAGGATTCAAATTCATAACGGAGA
GCTGGGCAGTAGCTTCCAAGCAAAGAACAGGTATTGCAGAAAAGACCCTCCATGAGTAGTGAGTAGTAGAGTGA
TGAGACCTTTGGTGAAAATAAACACACATGGCCTAGAAAGATGGAGAATTTGGGTACTTTT
ATCTTAATTCAGGTTGCACTTTTCCCCCAAGACTCAGGTGGCCTCCCAC
CACTTGAAGCCCTGCTTCCCTTTCTAGTAGGAAATAGTTTGCCTCCCTACTTTACTCCAGGTACCAATTCATCAGGG
GTATTGTGGAGGGCAGTGAGGAAGGTGGGTATAAGGGGGCTATGGTGGACTGGGAGAGAGAGATTATTCAGT
CCTCAAACTCAGTACTTACTCTTTGCCATCTCTAAGACTCAGTGACCAAATTAGCCTGA
GTCCTGCCTTCGTGGATGGAACTGACAGCCTCGTGGGGGATATACACATCCACATTTAATTTAATCATAATTAATT
GCAATCAGGAGGAATGCCTTGAAGGAGAAGGACTAGAAAGCACTAACTTAGTCTGGGGTTCAGGGAAGGCCAC
TGTAACCAGTTGACATGTCAAGGAAAGAAACCATAGCCCTGGGCGCAGTGGCTCACGCC
TGTAATCCCAGCATTTTGGGAGGCCGAAGCAGGCAGATCACAAGGTCAGGAGTTCAAGACCAGCCTGGCCAACA
TGGCGAAACCCTGTCTCTACTAAAAATACAAAAATTCACCAGGCGTGGTGGTGCGCACCTGTAATCCCAGCTACT
TGGGAGGCCGAGGCAGGAGAATCACTTGAACCTGGGCAGTGGAGGTTGCAGTGAGCCGA
GACTGTGCCATTGCACTCCAGCCTGGGCGACAGAGCAAGACTCTGTCTCAAAAAATAAAATAAAAAAGGAACTG
GGAGAAAACAAGGGAGAATTCCTTTATAACCTTGTAGTGGGCAAGGCCTTTCTACCTGTGAGTCAAAATCCAAAA
TCTAGAAGCCATAAAGGAAAAAATTGATCCATTGACTTTATAACATGAACATTAGGAAT
AGCCAAAAAGAAAAAAAAAAGCTATATTTATAGCTCAGATCACAAGAAAAGGGTAATATCCCTAATATAAAATGT
GTGCCTAGAAATTGGTAAGGGAAAGACCAGCAATCCAATCAGAAAATGGACAAAGGAGATTTATGAAAGAAAC
TTAGAAACAAGAAGCTAGGCCAGGCACAGTGGCTCATGCCTGTAATCCCAGCACTTTGGG
AGGCCGAGGTGGGCGGATCACTTGAGGCCAGGAGTTAAAGACCAACCTGGCCAACATGAAGAACTCTACAAAA
AAATACAAAAATTAGCCGGGTATAGTCGTGGGCGCCTGTAATCCCCCAGCTTCTTGGGAGGCTGAGGCAGGAGA
ATTGCTTGAACCTGGGAGACAGAGGTTACAGTGAGCTGACATCACACTCCAGCCTGGGCAG
CAGAGCGAGACTAAAAAAACAACAAGCTACCGTTTGTGCTGAATAG
GAGTTGGCCAGTGAAGAGGCGTGTGAAGTCCAGTGGTAGCTGGAAGACACTTGGTGGGACAACAGGTGAAGG
CGGGGACAGGAGGCCAGAAGGCTGGGGCACAGAGATGAGGGGCACTGAGTGTGCTGCAGAGCCCAGGGCCCA
GGGCACAAGGCTTTGGCCACTTCAGAACTTGCTACTTTCCCATAAGAGCAATGAGCAGGCTGGG
CACAGTGGCTCATACCTGTAATCCTAGCACTTTGGGAGGCCAAGGTGGAAGGATCATTTGAGCCCAGGAGTTTG
AGACCAGCCTGGGCAACAAAGCGAGACCCCCATCTCTATTTTATGGAAGAAATTAGGGCTGGGCATGGTTGCTC

ACATGTGTAATCCTAGCACTTTGGGAAGCTGAGGCGGGTGGATCACTTGAGG

TCAGGAGTTCGAGACCAGCCTGGCCAACATGGTAAAACCTCATCTCTACTAAAAATACAAAAATTAGCTGGGCGT

GGTGGCTCATGCCTGTAATCTCAGCTACTCAGGAGGGTGAGGCAGGAGAATCGCTTGAACCTGGGAGGCAGCG

TTTGCAGTGAGCTGAGATCGTGCCATTGCACTCCAGTCTAGGCAACAAAGTGAAACTCCA

TCTCCAAAAAAACAAACAAAAAAAAATTGTTTTTTCAAGTAATAAGCAACCGTTGAAAGGTTGTTTTTTTTTTTTT

TTGAGATGGAGTCTCGCTCTGTCGCCCAGGCTGGAGTGCAGTGGCGCGATCTTGGCTCACTGCCAGCTCCGCCTC

CCGGGTTCAAGTCATTCTTCCGAGTAGCTGGGACTACAGGCGCCCGCCACCACGCC

CAGCTAATTTTTTGTATTTTAATAGAGACGGGGTTTCACCATGTTAGCCAGGATGATCTCGATCTCCTGACCTTGT

GATCCACCCACCTCGGCCTCCCAAAGTGCTGGAATTACAGGAATGAGCCACTGCGCCCGGCCTGTTGAAAGGTTT

TAAGCAGGGAAATAACATGATTAGATTTGTATTTTATGTCTAAAAAATTTTGTCATT

TATGTCCCCCAAATTAATTTTATTGTTGTATGGAGACAGGGCTAGAGGAGGCAGACCAGGAAGCAGGGTGGGCA

CTTTGCCCTCCTTTCCAGTCCATCCCATGACTCTTGGTGGCTCTGACACCCCTGCAACCCTTTGAGGTGCCATGAGC

AAAAGACACAAAATTCCTCCTTTCCTGGAGCTTTCCTTCCAGTGTGGTCCGACAGAT

AGTAACACATACACATAAGCAAGATATGGTCAGTGCTAAGTGCTCAGGAGGACGTGAACAG

CTGATGGGGCAGAGTAGGGTGGGGAGGGACGGTATTAGAGGGCCCAGTGAAGCCACCCTGAGGAGGGGCTAT

CGCCTGGGGTCTGTGGAGCAAGGAGGGGCCGCTGTCTGGTTCTCAGCAGACTCCCCGTGGCCGGAGCGGGGAG

CAGTGGGAGAGCCTCCAGGGTGAGCTCAGGAGGTAGGCAGAGGCCGGGTCCCCTGGCCTGCAG

GTGTGGAGAGACACCTGGGTTTTGTTGTGAATGCTGTGAGAAGCCACTGAGGGTTTGTAAAGACTAGTTAGGAG

ATGGTCGCTGTTGCCCAGGCAAAAGATGAGGGTTGGTGGCAGTGGAGACGGAGACAGAGAGGTGAAGATATG

TTTTGGGGGAGATCGGACAAGAACTCCTGATGGGTTGTGGGGCAGCTGCGGAGAGTGAGTTG

CCAGCTCTCCATTTGCTGTGCACAGTTGGCTGATTGGTTGGGTCATTCTCTAAGGTCACAGAAAGTGGGAGTGAA

GGGAACAAGGAAGGCCTCCATGTGGGGTCGAGCCTCTGCTGAGCCCCCTCTTCTTTCCGCAGCTGTCCTCTTTGA

CACAGGCTCACGAGGAGGCTCTTTCCAGTTTGACCAGCAAGGCTGAGGGCTTGGAGAA

GTCTCTGAGTAGTCTGGAAACCAGAAGAGCAGGGGAAGCCAAGGAGCTGGCCGAGGCTCAGAGGGAGGCCGA

GCTGCTTCGGAAGCAGCTGAGGTAGGTGGGCGGACGCCGACGGGAGCCCAGCAATTAGTGATGTGGTGGATCT

GCAGGGCGCCCCACTGATGGCTGTCCCATTCCCACCCCAACCCTAGCAAGACCCAGGAAGACT

TGGAGGCTCAGGTGACCCTGGTTGAGAATCTAAGAAAATATGTTGGGGAACAAGTGCCTTCTGAGGTCCACAGC

CAGACATGGGAACTGGAGCGACAGAAGCTTCTGGAAACCATGCAGGTGAGGGTGCAGGAATGTATCTGTGTGC

AGACTTAGGGATCAGGTTGGGAGGCAAGCGTGGCCCTTGGAGGAGCGTGTAGAGCACAGCC

TCCGGGAGAGAAGGTGGTACCTAAGGCGGCATGGAGGCCCTACAGAGGGGCTGCTTTCCTCTGCCCGCAGCACT

TGCAGGAGGACCGGGACAGCCTGCATGCCACCGCGGAGCTGCTGCAGGTGCGGGTGCAGAGCCTCACACACAT

CCTCGCCCTGCAGGAGGAGGAGCTGACCAGGAAGGTACAGCCCAACCCCCAGACCCCTCAC

CCTCAGCCGCATCCTGCATCTACTGTCCCCT

GCCTCCCTCCCTGTGGGCAGGAGGGGTCAATGTGCCCCAGAACCTGCTTAGATCTCCTTCCTGTGAACTCCTCTTG

CTGTAGCTCATGTTGCCCAGGCAGGACAGAGGAGAAACAAAGATGCCACCTCCTTCCTCTCCTCCCCCAGGAGCC

CACACTTTTCTCCCACTCCTTCTCCCTCAGGTTCAACCTTCAGATTCCCTGGAGCCT

GAGTTTACCAGGAAGTGCCAGTCCCTGCTGAACCGCTGGCGGGAGAAGGTGTTTGCCCTCATGGTGCAGCTAAA

GGCCCAGGAGCTGGAACACAGTGACTCTGTTAAGCAGCTGAAGGGACAGGTCACTGCACTCTCTTTTCTCCCGGT

ATTCCCTCCCAGCACCTTGCTCCTTCCATGAAGGTGGCATCCATTCAACCAGTGTTTAT

TGAGTGGTTGCCACATGCTGGGCACACAGCCCTGAACAAAACTAAAATGTGGAGCTTGCATTCTAGAACAGAGA

CACAGAACACGCAAGTAAACAGATAATGTTGGGTAATTATATGTGCGATAGAAAGATTGAAGCCAGGTGCAGTG

GCTCACACCTATAATGCGATCACTTTGGTCTTGAACTCCTGACCTCAGGTGATTCACCTG

CCTCAGCCTCCCAAAGTGATGGGATTACAGGTGTGAGCCACCGTGCCCAGTCAAGTAATGCCAACAGTTTGGGA

GACCGAGGCAGGTGGATCACTGGAGGTCAGGAGTTCGAGACCAGCCTGGGCAACATGTGAAATCCCGTCTCTAC

TAAAAATACAAAAAATTAGCCGGTCATAGTGGCTCATTCCTGTAGTCCCAGCTACTCTGG

AGGATGAGGTGGGAGGATCACCTGAGGCTGGGAGGTCGAGGCGAGGCCACAGTGAACTGTGATCCCATCACTG

CACTCTAGCCTGGGTGACAAAGCGAGATCTTTTCTCAAAAAAAAAGAAAGTAGTAAGAAAAATTCAAAAGATAA

TGTGACGGAGAGACTGTGGGGTGAGTCAGCCTCAGGTAGGATGCTCAGAGACAGCCTCTCT

GAGGAGGTGACAGCATCTGAGGAGAGTGGCATGGTCAGTTGGTGGGTCTTGTGGGGTGGGTCAAGGGCTATTC

CCATCTTTGAGTGGGCACATGGAATGTGGAACATGGAACACTGGGCTCAGATTCCATCCTCAGAACCTAAGCTTC

TGTCTCCCTGCGTGGCATTCATTCTTTTTCTTTTCTTTTTTTTTTTTTTTTTTTTTTGAG

AAGGAGTCTTGTTCTTGTCACCAGGCTGGAGTGCAGTGGCCTGATCTCAGCTCACT

GCAACCTCCGCCTCCCAGGTTCAAGTGATTCTCCTGCCTCAGCCTCCCGAGTAGCTGGGATTACAGGCACATGCC

ATCACGCTCAGCTAATTTTTGTATTTTTAGTAGAGACAGGCTTTCACCATGTTGGTCAGGCTGATCTTGAACCCCT

GACCTCAAGTGATCCATCTGCCTCGGCCTCCCAAAGTGCTGGGATTACAGGTGTGAG

CCACCGTGCTGCAACCCACCCCCGTCGCCCGCCCCCCCTCCCCCCAGCCCCTGCATGGCATTCTTACAGAGATCTC

TGCACCTGCCACTTTGCTTCCAGTGCCCCCCTCATCTTTTAGCTCTAGAGGGCCCTGCCTAGCTCTCTCTCCTCCCCC

AGGTGGCCTCACTCCAGGAAAAAGTGACATCCCAGAGCCAGGAGCAGGCCATCC

TGCAGCGATCCCTGCAGGACAAAGCCGCAGAGGTGGAGGTGGAGCGTATGGGTGCCAAGGTTGGTGTCAGCCT

ACTAGAGACTCGGGGAGGGCAAGGGAGCCCCTGTTCCGGGGCTGCAGCCAGGACTTAGGGAGGGACCCTGTCC

TTTGCTGCATCCTCCCCAGGGCCTGCAGTTGGAGCTGAGCCGTGCTCAGGAGGCCAGGCGTC

GGTGGCAGCAGCAGACAGCCTCAGCCGAGGAGCAGCTGAGGCTTGTGGTCAATGCTGTCAGCAGGTATCAGGG

ATGGAGGGGTGGGTGGAGTAGTGTTTCTGCCACCTCAGGTTCCTGGGCACCTTGTTGCTGAGGATCCTCAGGCA

AGAGGGGCTGGAAAGTGGCCACTGGAGGCTACAGGGCTGGGCAGATTTAGCTCTATCAATG

TTCCTGTGTTCGTTTCTTTTCCTGGGGAAGCCCCTTCTGCATTCATACCTGATTGCTTGTTATGAATTTCCCGTTGCA

TGTTTGGCTGGAGGTGAGGCCTTGCTTCCTCCTGCAGTTCAGTCTAGTAATGGCTTGAGCTAAATAGAGCACCCG

GGAGGATCTTTACTTGCAGTATTGTTCAAG

GATGGAGAGTGTAGACACTTCATCTTCCTTTTTTTTTCTAAAATTTTACGGGCAATCCGTTTCACTGGAGAAAAATT

TAGTCTATTTATTTATTTATTTTGAGACAAAGTCTCGCTCTGTCACCCAGGCTGGAGTGCAATGGCGCAATCTTGG

CTCACTGCAACCTCACCTCCCTGGTTCAAGTGATTCTCCTGCCTCAGCCTCCCGA

GTAGCTGGATTACAGGCATCCTCCACCAGTGTCCTCCACTACACCCGGCTAATTTTTGCATTTTTAGTAGGGACGG

GGTTTCAC

CATGTTGGCCAGGCTGGTCTTGAACTCCTGACCTCAGGTGATCCACCCACCTCAGCCTCCCAAAGTGCTGGAATTA

TAGGTGTGAGCCACTGCACCTGGCCTAGTCTATTTATTTAAAGCTATATACTTACTTGCTTATTATATACTTAACTT

GCTTACTATTCCATCTAAAATGTAAGCCAGTTAGTTTCCTTCTAAATCAATTGCC

AGCCTTTGTCTCTCCTACCAACTTCCTAGTTGTTTCATTACCTACAATTGTTGTATGACCTTCAGAAAAACCTCTAAG

AAAACAGCAAAGCTTCTTTGTGCTGGTGATGACTTCCCCTCAGCCTTAGACACTGAGGTACCCAAGGCAGGTAGT

TCTTTTTTTTTTTTTTTTTTTTTTGAGACAGAGTCTCGCACTGTCACCCAGGCT

GGAGTGCAATGGCACGATCTCAGCTCACTGCAACCTCTGCCTCCCGGGTTCACACGATTTTCCTGCCTCAGCCTCC

TGAGTAGCTGGGATTACAGGTGCACACCACCACACCCGGCTACTTTTTTGTATTTTTAGTAGAGACAGGGTTTCAC

TGTGTTGGCCAGGCTGGTCTCAAACTTCTGACCTCGTGATCCGCCCGCCTCGGCCT

CCCAAAGTGCTGGGATTACAGGCTTGAGCCACCGTGCCTGGCCGGCAGGTAGTTCTTAGCACAGTCTCTGGCTTG

TAAATGTTTATTGTTATCGTGAGGCTCTTCTTGATGGGTTAATTTAGATAAAGATAATTTTTGGTTTAGCGAAATTA

AGATGCAGGATGAGTCCTTGCCCATCACTTCCTTCTCTTGGATTTGTACCTTAGGG

ACTAATTTAGCTTTAAAAATTATGAAAAATTTCAAACTTGCACGGAATTAAACTAGTTATAATGACCTACCACCCA
GGTTCAATCCATCGCGGGTGCCCCCTACCTCCAGGAGAACAGAAAGATGCACTGTGGACAGGGTTTGACTTTGG
TACCAGGCGATCACAGAAATGGTCTGTGTAATGATGCATTTGCCGAAAGTCCTCCAGG
CTTAAAGCAGTCCAAACTCTGATTGTTGCTGGGTTTATTAGATTGTCTCTAGGTAATTGGAGACTTTAATAAGGGC
TGTGGTAGGTGTTGGAAGCATCTACTGGAACAATTTCCAGATCAAAGTGAACTTTGCTGTGCTGCTGGGGATGCA
GCTGCAAGCCTTCATCATCATGTGTTTTTCTGTGGGTGCAGACCTGGACTCTCCTGA
GGAAACCCCAGCCCGGCCCCAGACACTCCTTGGCCTCCTCCTGGGGCCTGTTTAAGCTGCTCAGTTTTCATGAGC
CAGGTTGGTCCTACTTCTGGCACAGCCAGCTGGTAAAGCATGTGGACCTGCCCGTCATTGGTGCTAGATCGACAC
TCCTGGGCTTGGAGAGGATAACTTTGTTTTCTTTTGTTTTTTTGAGATGGAGTCTCGCTCTGTCACCCAGTCTTGAG
GGCAGGGGTGCGATCTTGGCTCACTGGAACCTCCACCTCCTAGGTTCAAGTGATTC
TCGTGCCTCAGCTTCTGGAGTAGCTGGGATTACAGGCATGAGCCACCATGCCCGGCTAATTTTTGTGTTTTTAAGT
AGAGAGAGTTTCACCATGTTGGCCAGGCTAGTCTCCAATTCCTGACTTCAGGTGATCCGCCCGCCTCGGCCTCCCA
AAGTGCTGGGATTACAGGCAGGAGCCACTGCCCCTGATCAGAGAGGATAACTTTAC
TCTTTGATACACGATAGTGAGCAAAACACAGTTGTGAGAAATAAGCTTAACAGGTTGCTTAAAAAGATAGTCA
TTTAATGCATTCTTGGGGCAAGGGTCCTTTAGATAATTGACGGAAGCTGTGCGTTCTGTACTTGTATAATGGGAC
AGGATTAGAGGGAGTTGTCTATACAAGGCACAGCAAGTCCTTTGGGAATGAGGGGAGGCATGGAGGATCAGTG
ACTTGTGCCCTCTCCAGCTCTCAGATCTGGCTCGAGACCACCATGGCTAAGGTGGAAGGG
GCTGCCGCCCAGCTTCCCAGCCTCAACAACCGACTCAGCTATGCTGTCCGCAAGGTCCACACCA
TTCGGGGTGCGTAGGACAACTGCGAGCCACGTCCTGCCCCCACCCCACCAGCTCGGACTTTCTTCTTCCTGACCCA
GCTCTCTCTGATCCCACATCCATTCACCTTCCTCCTTTCACCAGTCCTTGCATCTCTTTTTCCCTTACTCCCTGTCCCC
ACTTTCTCCCATGCAAACTTCATCTCTTTTTCTCCCTGCTTTTTCCCCTCCAG
GCCTGATTGCTCGAAAGCTTGCCCTTGCTCAGCTGCGCCAGGAGAGGTGAAGTTTGGGCACTTTGAGGTGGATG
GGGCTTTAGGGCATTGGCTGCTGGGACCCCCAAAACCATGAGGACTGAGGTGGGATGGGGGCTTTGGGATCAG
GCAGCTGGGTGATTTCTTCTGACTCTTTCTCTTCCCCGTCCCAGCTGTCCCCTACCACCAC
CGGTCACAGACGTGAGCCTTGAGTTGCAGCAGTTGCGGGAAGAACGGATGAGCCTGGATGCAGAACTGCAGCT
GAGTGCCCGCCTCATCCAGCAGGAGGTGGGCCGGGCTCGGGAGCAAGGTACACCTGGT
TGCCAGGGGGTGGAGAGGATGAGGAAAAACCCGGTGTCTAGGGTGCTGGGAGAGGCCTGACCCAGCACCCCCT
CCTTTTAGGGGAGGCAGAGCGGCAGCAGCTGAGCAAGGTGGCCCAGCAGCTGGAGCAGGAGCTGCAGCAGAC

CCAGGAGTCCCTGGCTAGCTTGGGGCTGCAGCTGGAGGTAGCACGCCAGGGCCAGCAGGAGAG<br>CACAGAGGAGGCTGCCAGTCTGCGGCAGGAGCTGACCCAGCAGCAGGAACTCTACGGGCAAGGTGTCGAGAGG<br>GAAATGGGTGCTTCCCTTGGAGGGTGGGGTGGGAACTGCGAATCAAAGCTCCTGCTGATATGCCCCGTCTGCAC<br>TTTCACCCCAGCCCTGCAAGAAAAGGTGGCTGAAGTGGAAACTCGGCTGCGGGAGCAACTC<br>TCAGACACAGAGAGGAGGCTGAA

Judging from the results of verification one, the cell line we verified knocked out the large fragment between exon3-exon11, which made the CCHCR1 gene we want to knock out not express normally

The results of the verification two **are as follows:**

First, the results of electrophoresis

<table>
<tr>
<td rowspan="2">Analysis and illustration of the results (knockout of sg3 and sg4 with two pairs of primers).</td>
<td>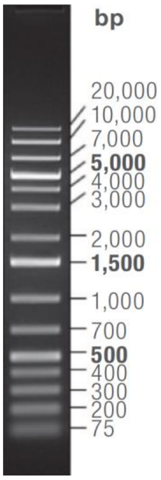
</td>
<td>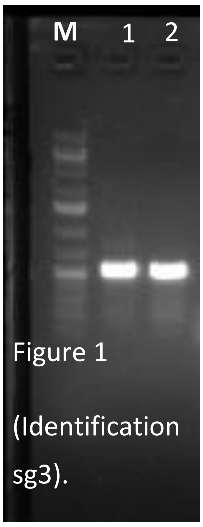

No. 1 is a monoclonal cell
No. 2 is the control group without transfection of cells</td>
<td>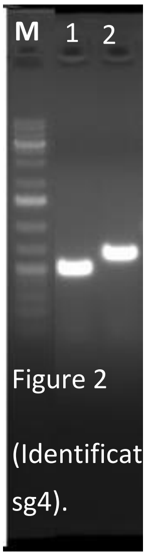

No. 1 is a monoclonal cell
No. 2 is the control group without transfection of cells</td>
</tr>
<tr>
<td colspan="3">The above figure shows the results of the monoclonal cell genome PCR after running glue, from the electrophoresis figure 1, the size of the monoclonal genome PCR band and the control group is not significantly different, from electrophoresis figure 2 it can be seen that the genome PCR band of the monoclonal cell is smaller than the PCR band of the control group, indicating that a part of the fragment is knocked out, and the results of the two indicate that the cell line is a heterozygote</td>
</tr>
<tr>
<td colspan="4">Second, the sequencing results</td>
</tr>
<tr>
<td colspan="4">Correspond
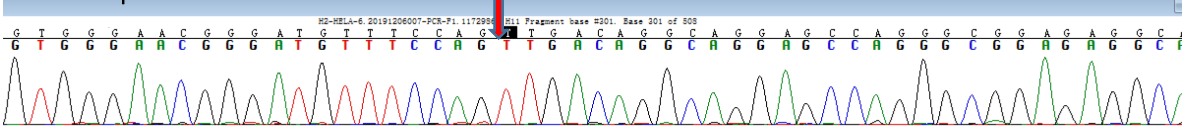
Corresponds to Figure 2
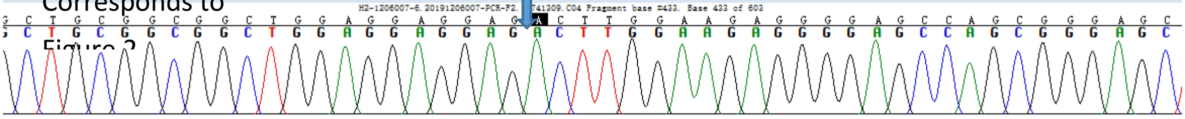</td>
</tr>
</table>

The figure above shows the sequencing results of monoclonal cells after CCHCR1 gene knockout, the red arrow indicates the insertion location, and the blue arrow indicates the gRNA knockout location

3. Knock out the sequence

CcHCR1 **gene cDNA part sequence (marked red is the knockout part, green is the insertion part).**

gRNA acts on Exon3

GTTCCACTGGGCTGATTCCCCCCTCCCACTTTCAAGCTCGGCCCCTTTCAACTCTGCCAAGAATGGCTCCCACCTGG

CTCTCAGACATTCCCCTGGTCCAACCCCCAGGCCATCAAGATGTCTCAGAGAGGCGGCTAGACACCCAGAGACCT

CAAGTGACCATGTGGGAACGGGATGTTTCCAG TTGACAGGCAGGAGCCAGGGCGGAGAGGCAG

gRNA acts on Exon4

CCTGGGGGCTGGAGGGGTCACAGGCCCTGAGCCAGCAGGCTGAGGTGATCGTTCGGCAGCTGCAAGAGCTGCG

GCGGCTGGAGGAGGAG

GTCCGGCTCCTGCGGGAGACCTCGCTGCAGCAGAAGATGAGGCTAGAGGCCCAGGCCATGGAGCTAGAGGCTC

TGGCACGGGCGGAGAAGGCCGGCCGAGCTGAGGCTGAGGGCCTGCGTGCTGCTTTGGCTGGGGCTGAGGTTGT

CCGGAAGA ACTTGGAAGAGGGGAGCCAGCGGGAGCTGGAAGAGGTTCAGAGGCTGCACCAAGAGCAG

CCHCR1 **genome partial sequence (marked in red is knockout part, knockout genome between 3526-3679bp sequence, green is inserted part).**

gRNA acts on Exon3

GTTCCACTGGGCTGATTCCCCCCTCCCACTTTCAAGCTCGGCCCCTTTCAACTCTGCCAAGAATGGCTCCCACCTGG

CTCTCAGACATTCCCCTGGTCCAACCCCCAGGCCATCAAGATGTCTCA

GAGAGGCGGCTAGACACCCAGAGACCTCAAGTGACCATGTGGGAACGGGATGTTTCCAGTTGACAGGCAGGAG

CCAGGGCGGAGAGGCAG

gRNA acts on Exon4

CTCAGCCAGACTTTAGGTCCTGTGAGTCCAGGCACTGGACTCAACATGCTCAATAGGGCTTTGATGAATGATGAT

GATGTCAATGCAGACATCCCCATACCCCAGCTTCAGCACCCCCTTCACCTCCCCACACGGAAGCAGAGGGGTCCT

CTTTTCCTTCTCCTGGCTATGTTTATGCCCTCAACTATCCTTCCAGCACTGGAGACAA

GTCTCACCTGCACTAACCTGTCTTTGAAGGTCCTGGGGGCTGGAGGGGTCACAGGCCCTGAGCCAGCAGGCTGA

GGTGATCGTTCGGCAGCTGCAAGAGCTGCGGCGGCTGGAGGAGGAG

GTCCGGCTCCTGCGGGAGACCTCGCTGCAGCAGAAGATGAGGCTAGAGGCCCAGGCCATGGAGCTAGAGGCTC

TGGCACGGGCGGAGAAGGCCGGCCGAGCTGAGGCTGAGGGCCTGCGTGCTGCTTTGGCTGGGGCTGAGGTTGT
CCGGAAGA
ACTTGGAAGAGGGGAGCCAGCGGGAGCTGGAAGAGGTTCAGAGGCTGCACCAAGAGCAGGTGAATGCAGGGG
TAGAAAGGATTCAAATTCATAACGGAGAGCTGGGCAGTAGCTTCCAAGCAAAGAACAGGTATTGCAGAAAAGAC
CCTCCATGAGTAGTGAGTAGTAGAGTGATGAG

From the results of the verification two, the cell line is heterozygote, but due to the transcoding of both exon3 and exon4, a terminator will appear, which will make the CCHCR1 gene to be knocked out unable to express normally.

conclusion

Combining the results of validating one and two can verify that the cell line is a heterozygote that the CCHCR1 gene cannot express properly

GRNA target sequence design description

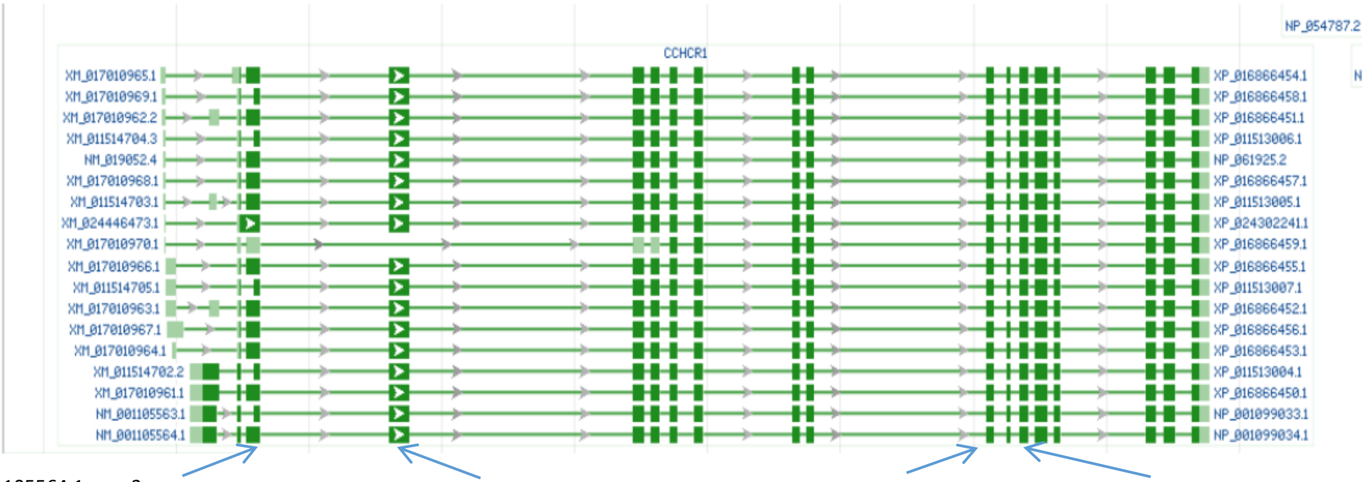

NM_001105564.1 exon3 NM_001105564.1 exon4 NM_001105564.1 exon11 NM_001105564.1 exon13

Let's take a transcript of this gene NM_001105564.1 as an example. We used sgRNA3 designed for exon 3 and sgRNA4 for exon 4, sgRNA1 for exon 11, and sgRNA2 for exon 13 for building knockout cell lines.

Shipping & Storage Conditions

Full dry ice transportation. When receiving the cell line package, please check the cell line cryovial immediately for thawing, and if so, take a photo immediately and notify Party B. Carry out experiments as soon as possible after the cell line is received, and transfer to liquid nitrogen if you want long-term preservation.

Ordering Instructions

ViGene Biosciences products are for research purposes only and may not be used for other purposes, including in vitro diagnosis and treatment in humans. **ViGene Biosciences**' products may not be modified or resold to any third party and may not be used to provide services to other third parties or to manufacture commercialized products without the written approval of **ViGene Biosciences**.

The full-length cDNA clone of ViGene Biosciences comes from human cDNA. 70% of our clones are identical to sequences in the RefSeq and GenBank databases, and some of the cDNA clone sequences contain SNPs, possibly with one or more nucleotide differences from the sequences in the RefSeq database. Buyers are advised to carefully check the clone sequence before purchasing. According to the sequence information in the GenBank 2013 database, each clone in the product contains a complete open reading box (ORF), and GenBank's data is constantly updated as the RefSeq sequence continues to

change. All of our clones have been validated by NestGen sequencing II full-length sequencing and are fully identical to the sequences available on our website.

Use of this product is subject to the above terms and the product regulations on http://www.vigenebio.c n of the website. You are responsible for reading, understanding and complying with any restrictions arising from these Terms.

Quality Control Officer:

Date:
