# Supplementary material for: CCHCR1-astrin interaction promotes centriole duplication through recruitment of CEP72
Source: BMC Biol. 2022 Oct 24;20:240. doi: 10.1186/s12915-022-01437-6 (PMC9590400; doi:10.1186/s12915-022-01437-6)
Supplement: Supplementary file 9 — Additional file 9. Verification reports of astrin-KO cell line. [file 12915_2022_1437_MOESM9_ESM.pdf]

Project report——knockout cell line (Electroporation)

1. Project Info

Contract ID：UBISCK200908JM1

Target Gene (ID)：SPAG5

Cell line：Hela

2. Pre-test results

Optimal Puromycin concentration for drug screening	2ug/ml
Electroporation parameters	1300v，30 ms，1 pulse
Single clone formation rate	☑ Level I(simple)　☐ Level II(intermediate)　☐ Level III(complex)

3.Gene knockout strategy

1）gRNA Sequence (target exon 3)

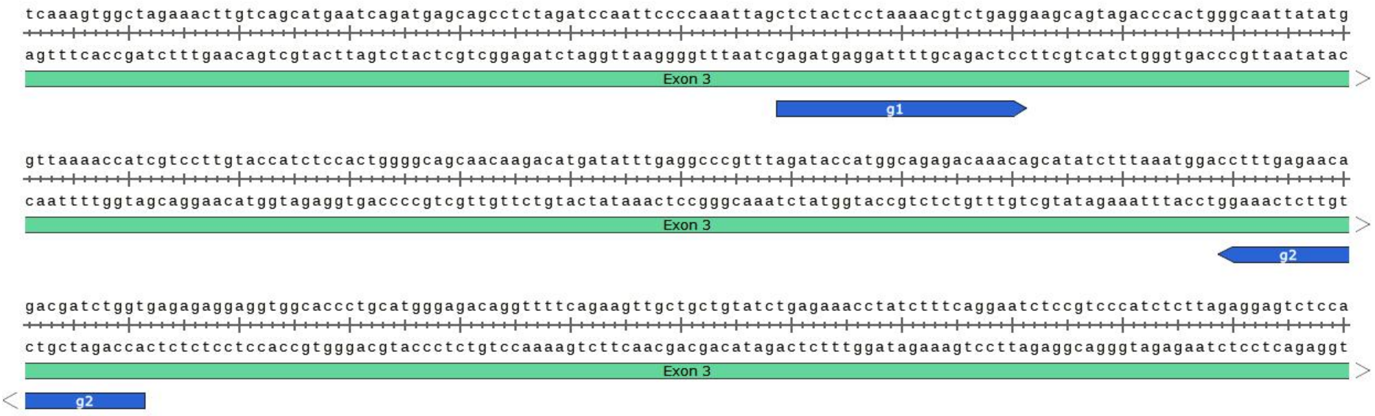

Sequence：

SPAG5-gRNA1: CTCTACTCCTAAAACGTCTG <u>AGG</u>
SPAG5-gRNA2:ACCAGATCGTCTGTTCTCAA <u>AGG</u>

4. Validation results

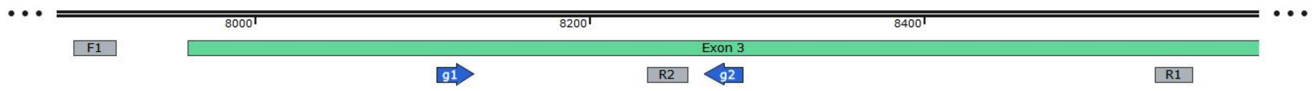

1) PCR Identification

Clone No	Lane	Primer	Fragment	Result
#A7	1	F1/R1	520 bp	√
#B8	2	F1/R1	520 bp	√
WT	3	F1/R1	669 bp	√
#A7	4	F1/R2	0 bp	√
#B8	5	F1/R2	0 bp	√
WT	6	F1/R2	367 bp	√
SPAG5-F1:GCACCTGTAATTGTATGTCGGGAAC				
SPAG5-R1:CACGGAAATCTGCTGCCAAGGC				
SPAG5-R2:GATATGCTGTTTGTCTCTGCCATG				

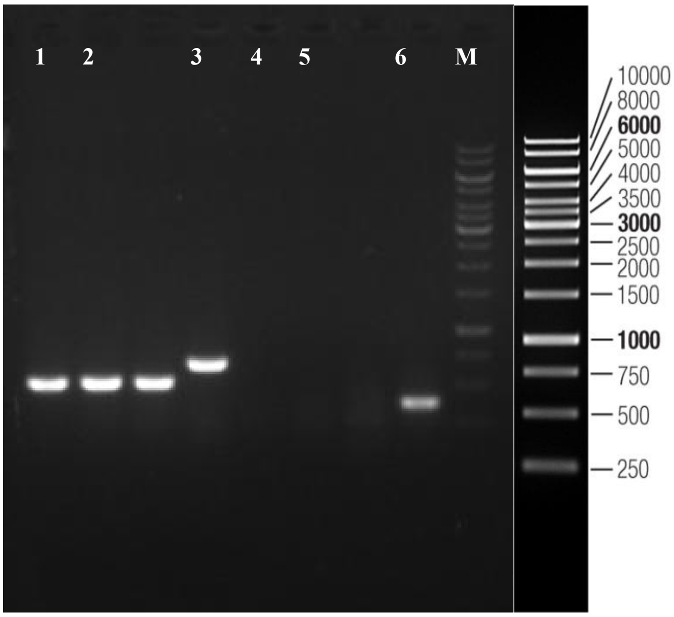

PCR Identification for positive clones

2) Sanger Sequencing (Detail result is attached as a zip document)

#A7 Clone:

Genotype of #A7 Clone(del 149 bp):

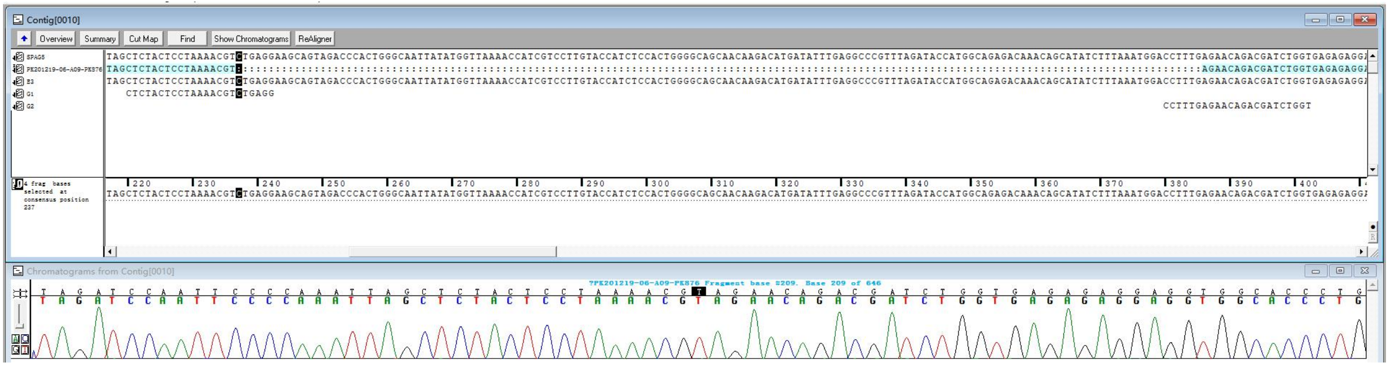

CAAATTAGCTCTACTCCTAAAACGT(~~CTGAGGAAGCAGTAGACCCACTGGGCAATTATATGGTTAAAACCATCG TCCTTGTACCATCTCCACTGGGGCAGCAACAAGACATGATATTTGAGGCCCGTTTAGATACCATGGCAGAGAC AAACAGCATATCTTTAAATGGACCTTTG~~)AGAACAGACGATCTGGTGAGAGAGGAGGTGGC

#B8 Clone:

Genotype of #B8 Clone(del 149 bp):

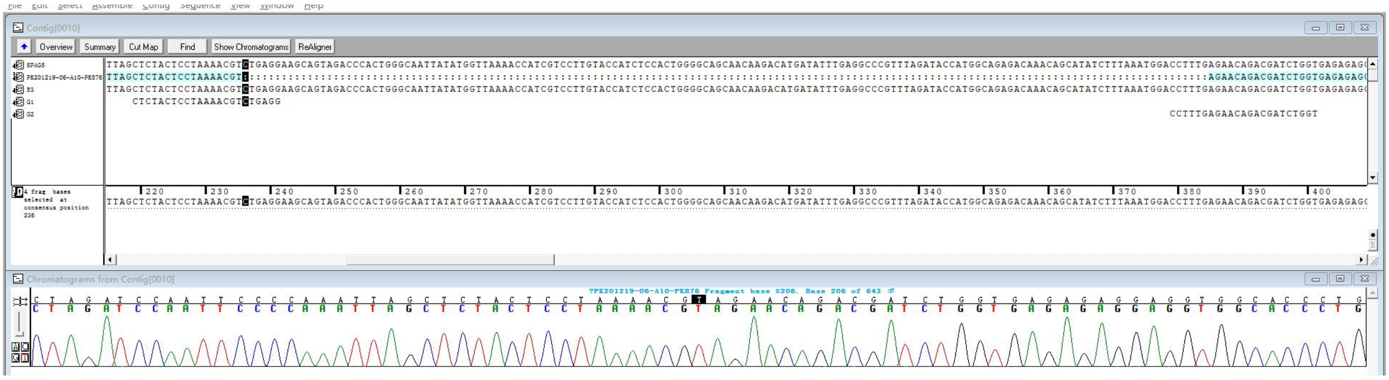

CAAATTAGCTCTACTCCTAAAACGT(~~CTGAGGAAGCAGTAGACCCACTGGGCAATTATATGGTTAAAACCATCG TCCTTGTACCATCTCCACTGGGGCAGCAACAAGACATGATATTTGAGGCCCGTTTAGATACCATGGCAGAGAC AAACAGCATATCTTTAAATGGACCTTTG~~)AGAACAGACGATCTGGTGAGAGAGGAGGTGGC

5.Positive clone image

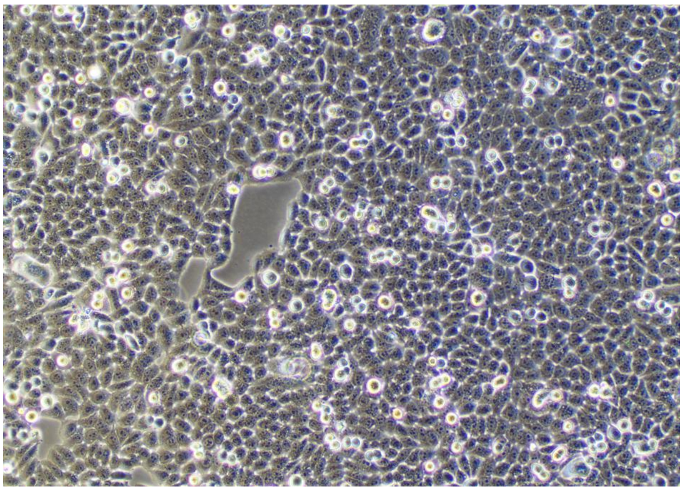	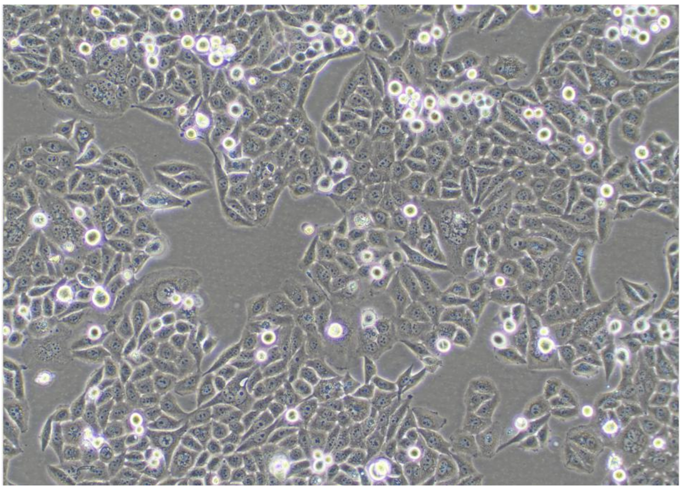
Hela-SPAG5-Clone#A7	Hela-SPAG5-Clone#B8
