# Supplementary figures and images for: CCHCR1-astrin interaction promotes centriole duplication through recruitment of CEP72 (part 2 of 2)
Source: BMC Biol. 2022 Oct 24;20:240. doi: 10.1186/s12915-022-01437-6 (PMC9590400; doi:10.1186/s12915-022-01437-6)

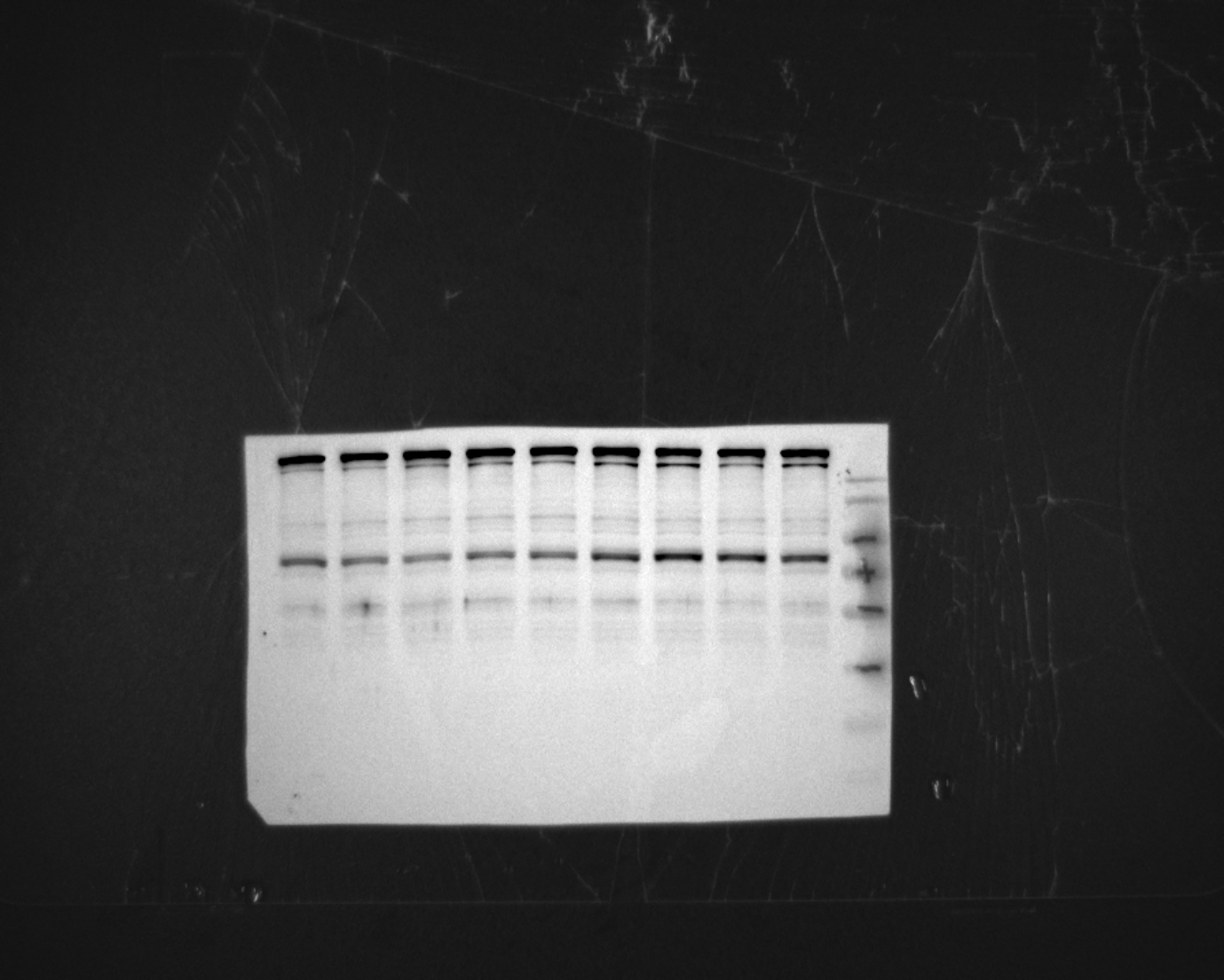

Supplement: Supplementary file 10 — Additional file 10. All Original and uncropped blots images used in manuscript. [file 12915_2022_1437_MOESM10_ESM.zip › blots images/Fig 4/Fig 4 C/CEP72/Fig4C-CEP72-marker.tif]

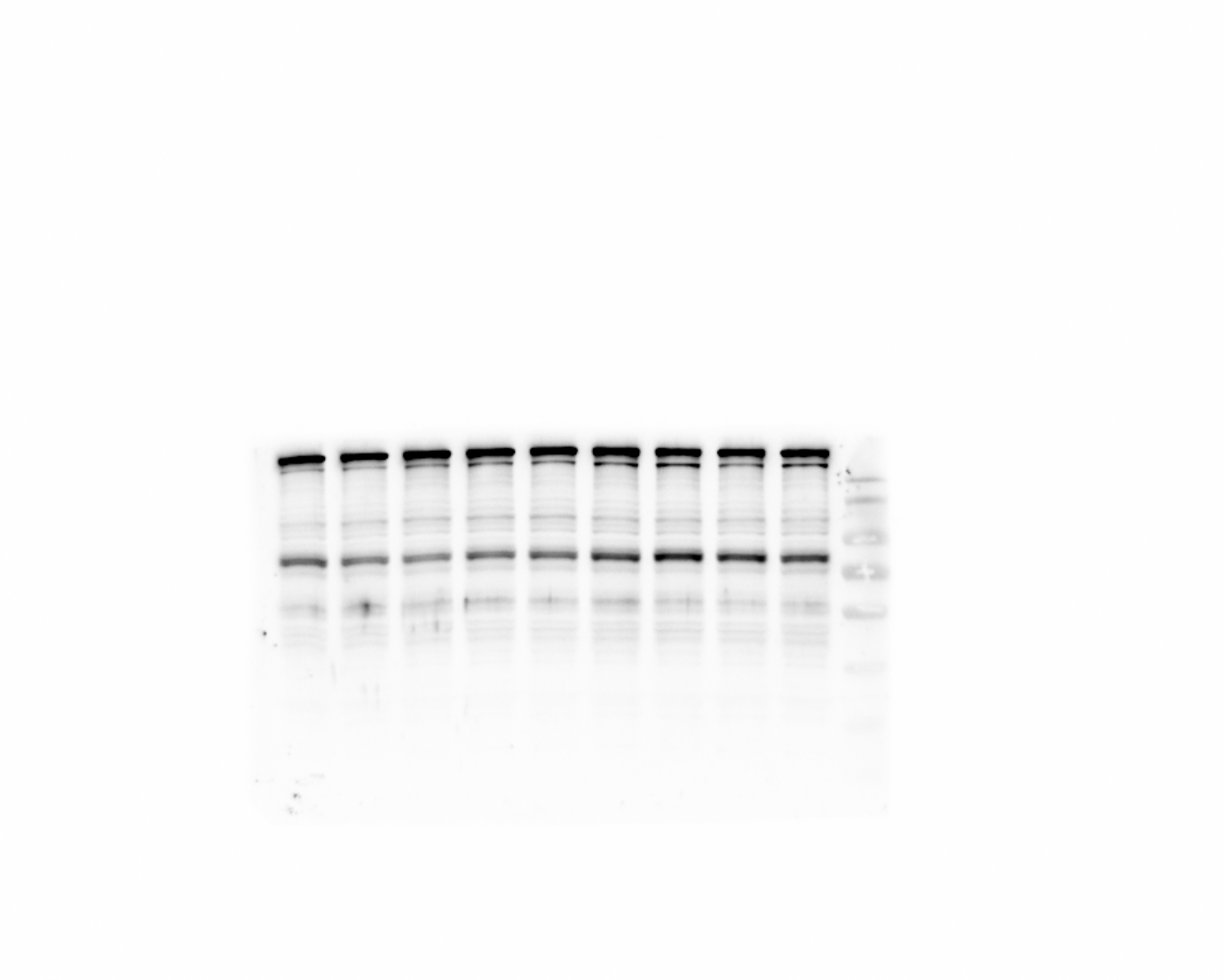

Supplement: Supplementary file 10 — Additional file 10. All Original and uncropped blots images used in manuscript. [file 12915_2022_1437_MOESM10_ESM.zip › blots images/Fig 4/Fig 4 C/CEP72/Fig4C-CEP72.tif]

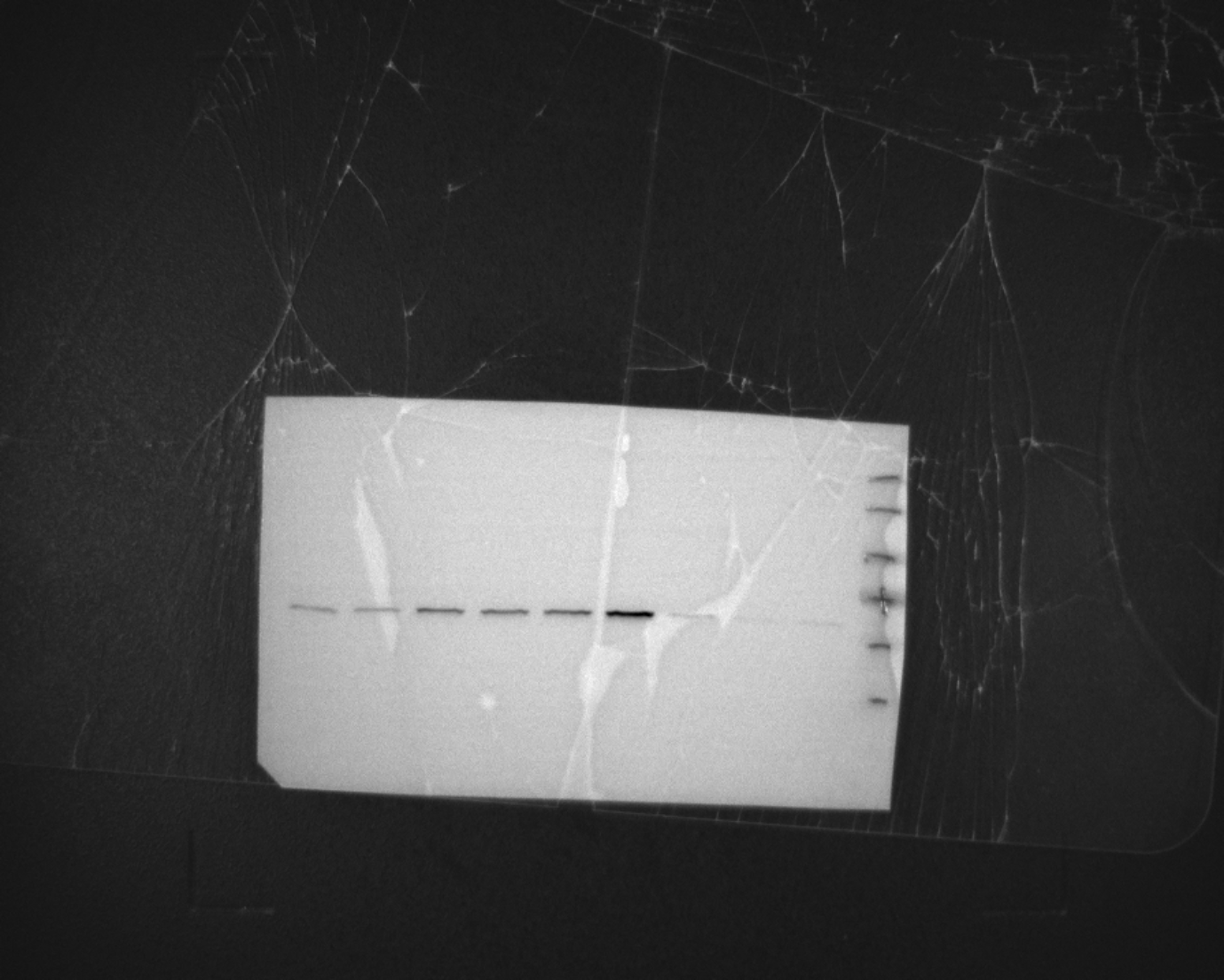

Supplement: Supplementary file 10 — Additional file 10. All Original and uncropped blots images used in manuscript. [file 12915_2022_1437_MOESM10_ESM.zip › blots images/Fig 4/Fig 4 C/cyclin B1/Fig4C-cyclinB1-marker.tif]

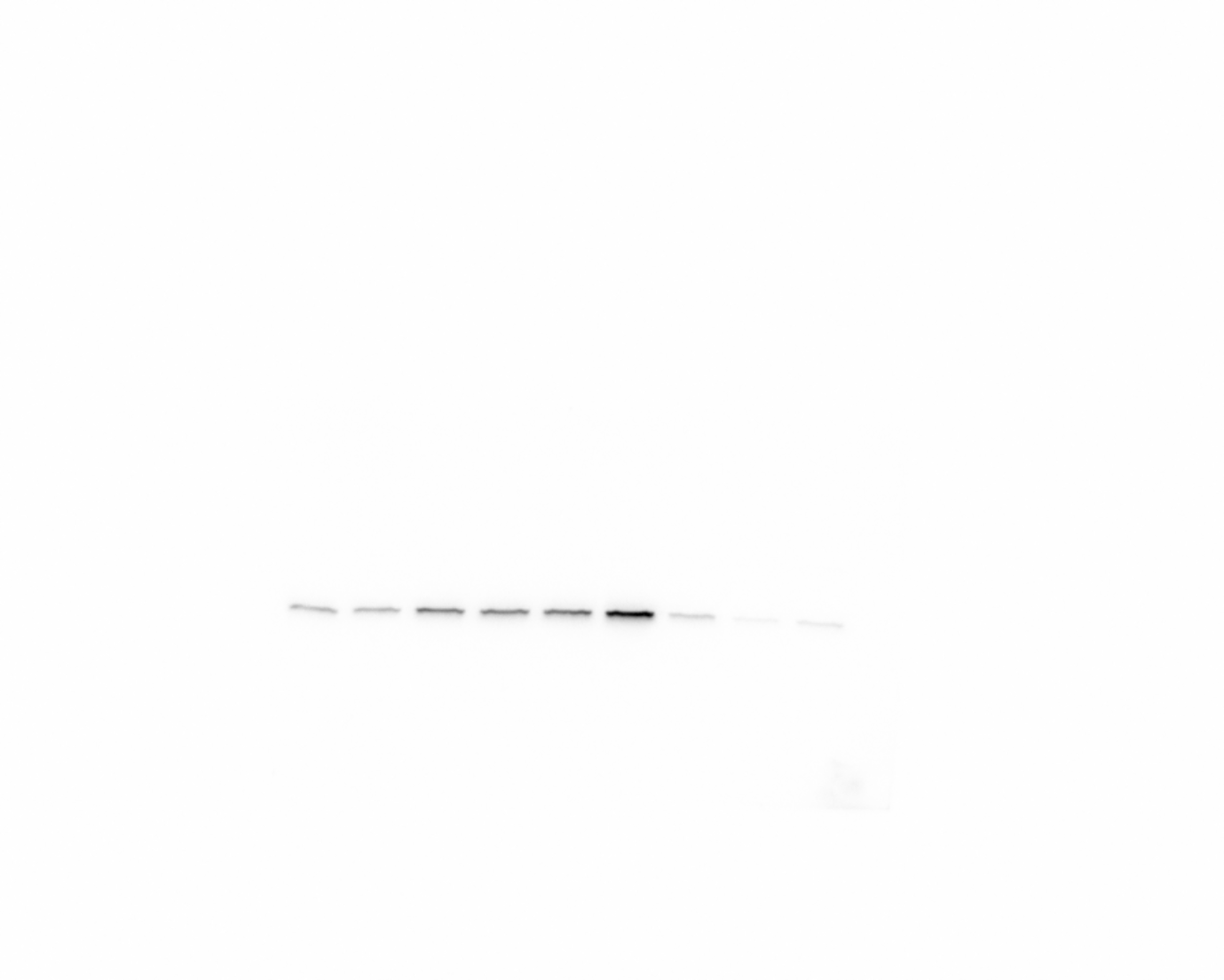

Supplement: Supplementary file 10 — Additional file 10. All Original and uncropped blots images used in manuscript. [file 12915_2022_1437_MOESM10_ESM.zip › blots images/Fig 4/Fig 4 C/cyclin B1/Fig4C-cyclinB1.tif]

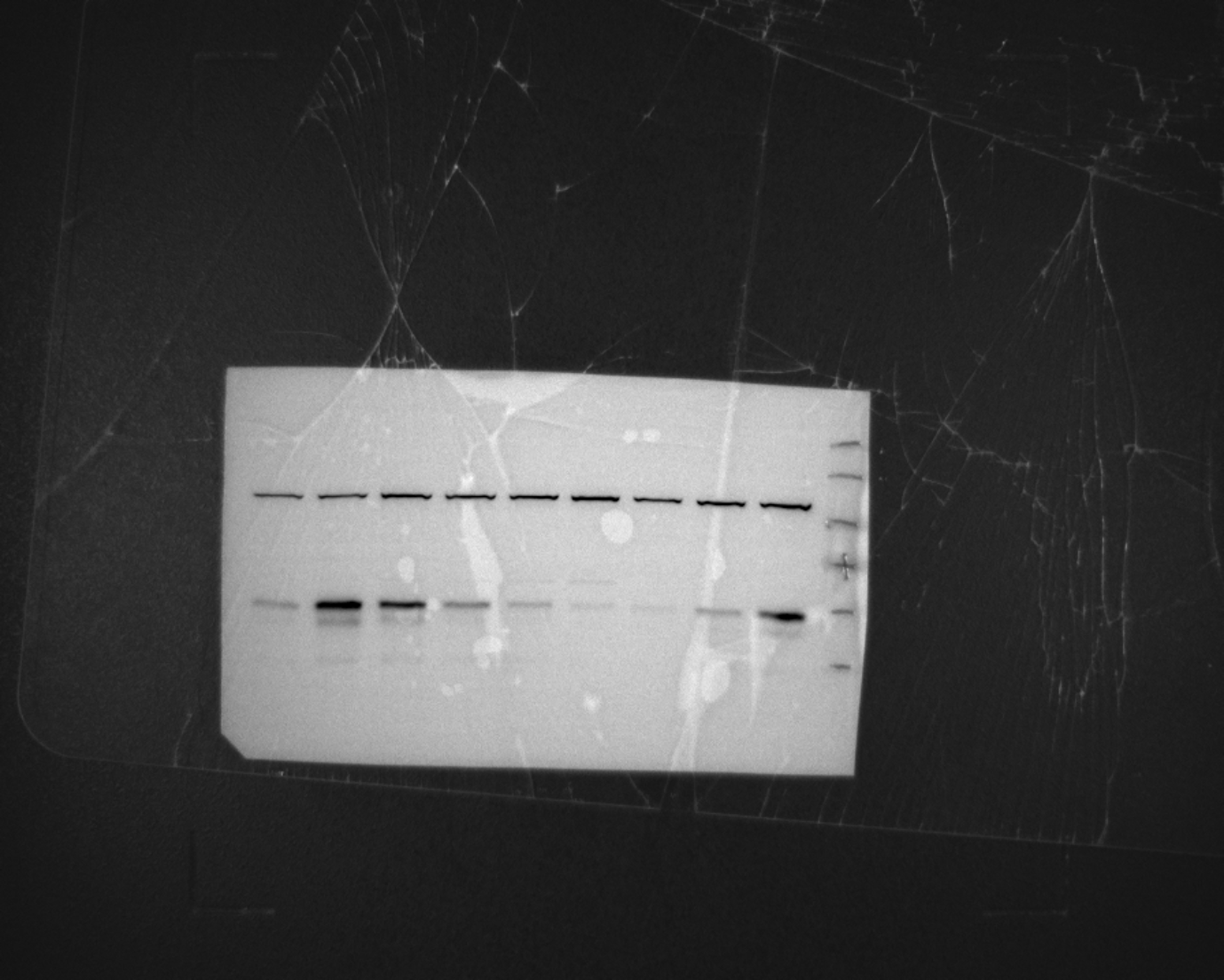

Supplement: Supplementary file 10 — Additional file 10. All Original and uncropped blots images used in manuscript. [file 12915_2022_1437_MOESM10_ESM.zip › blots images/Fig 4/Fig 4 C/cyclin E/Fig4C-cyclin-E-marker.tif]

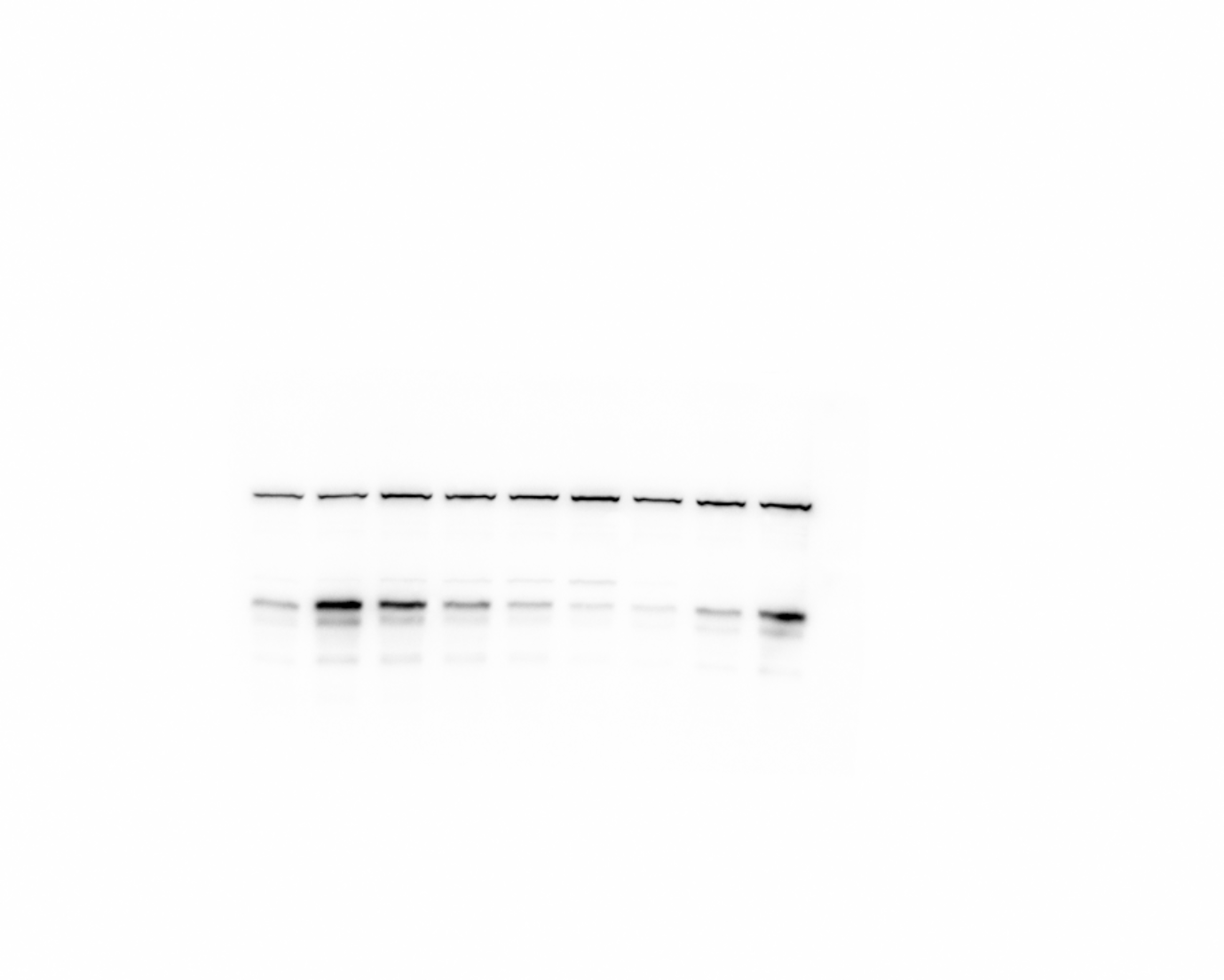

Supplement: Supplementary file 10 — Additional file 10. All Original and uncropped blots images used in manuscript. [file 12915_2022_1437_MOESM10_ESM.zip › blots images/Fig 4/Fig 4 C/cyclin E/Fig4C-cyclin-E.tif]

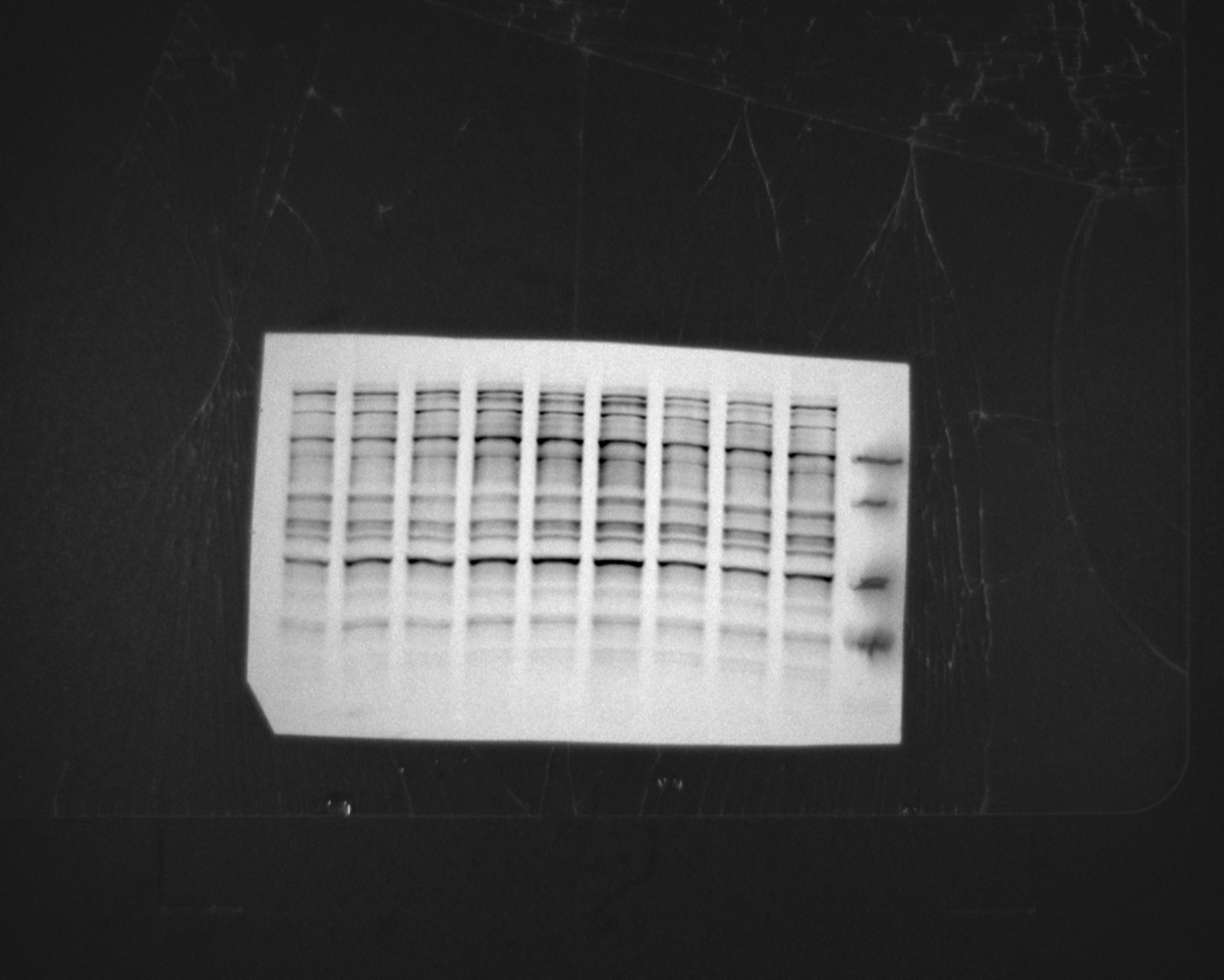

Supplement: Supplementary file 10 — Additional file 10. All Original and uncropped blots images used in manuscript. [file 12915_2022_1437_MOESM10_ESM.zip › blots images/Fig 4/Fig 4 C/HCR/Fig4C-HCR-marker.tif]

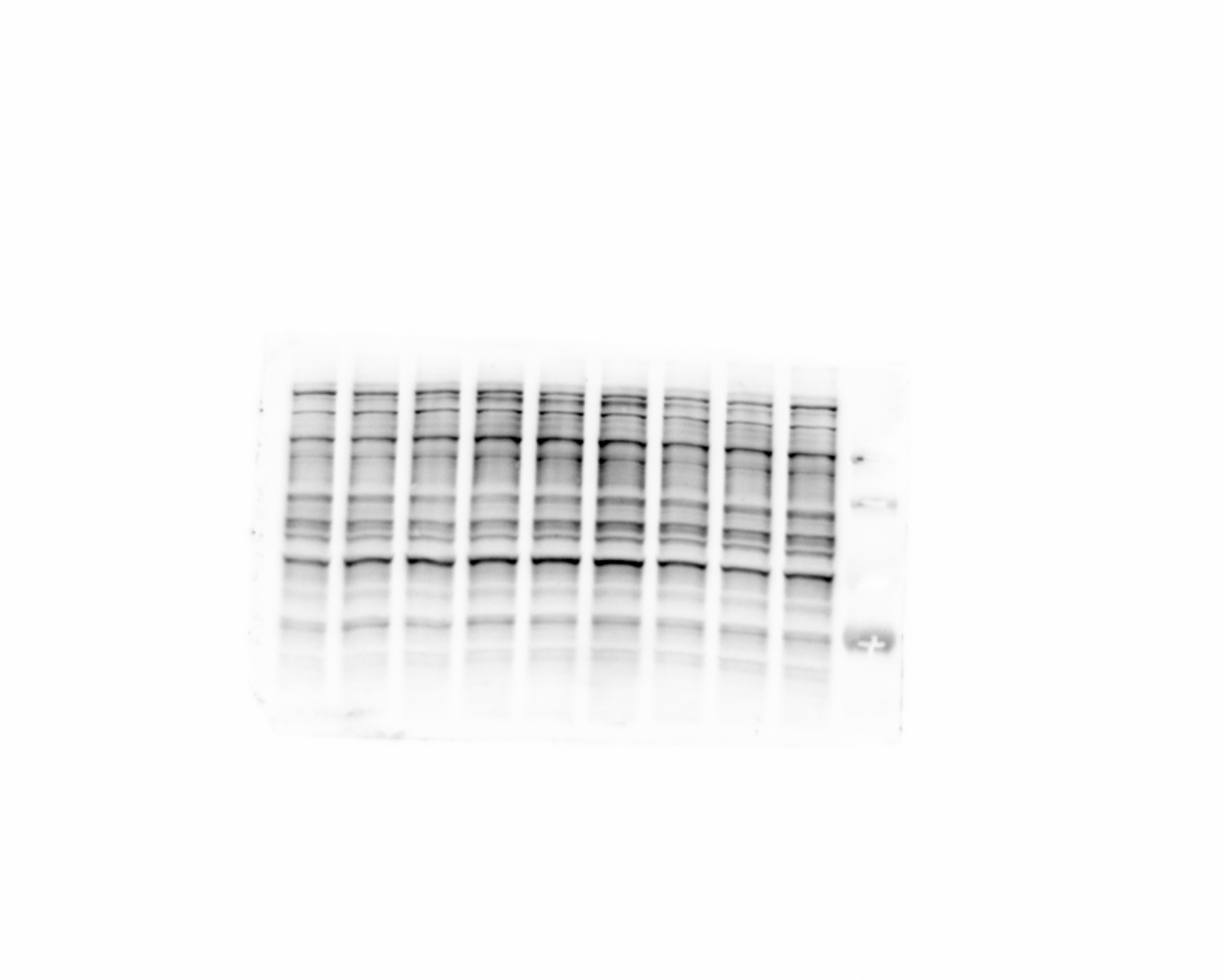

Supplement: Supplementary file 10 — Additional file 10. All Original and uncropped blots images used in manuscript. [file 12915_2022_1437_MOESM10_ESM.zip › blots images/Fig 4/Fig 4 C/HCR/Fig4C-HCR.tif]

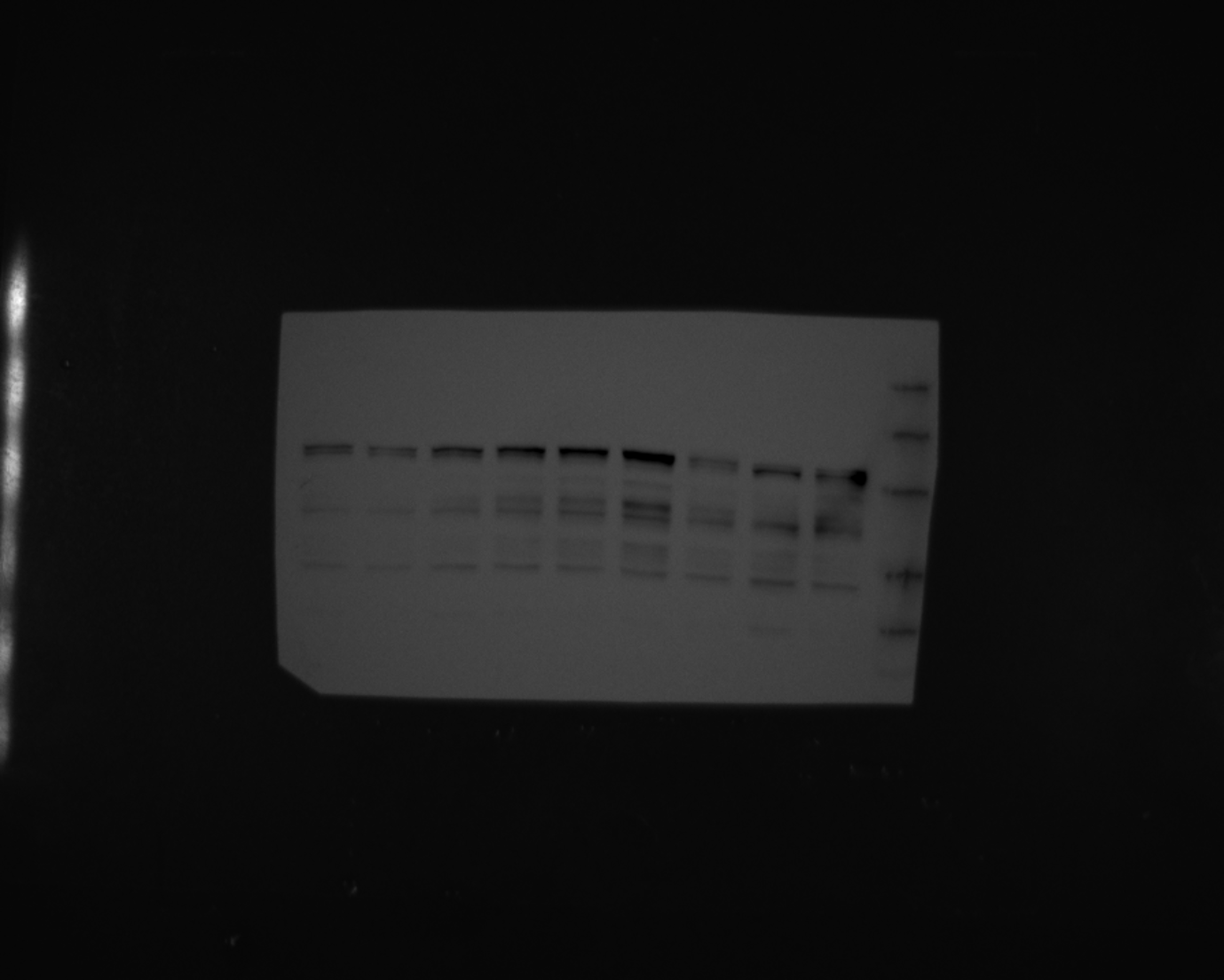

Supplement: Supplementary file 10 — Additional file 10. All Original and uncropped blots images used in manuscript. [file 12915_2022_1437_MOESM10_ESM.zip › blots images/Fig 4/Fig 4 C/HURP/Fig4C-HURP-marker.tif]

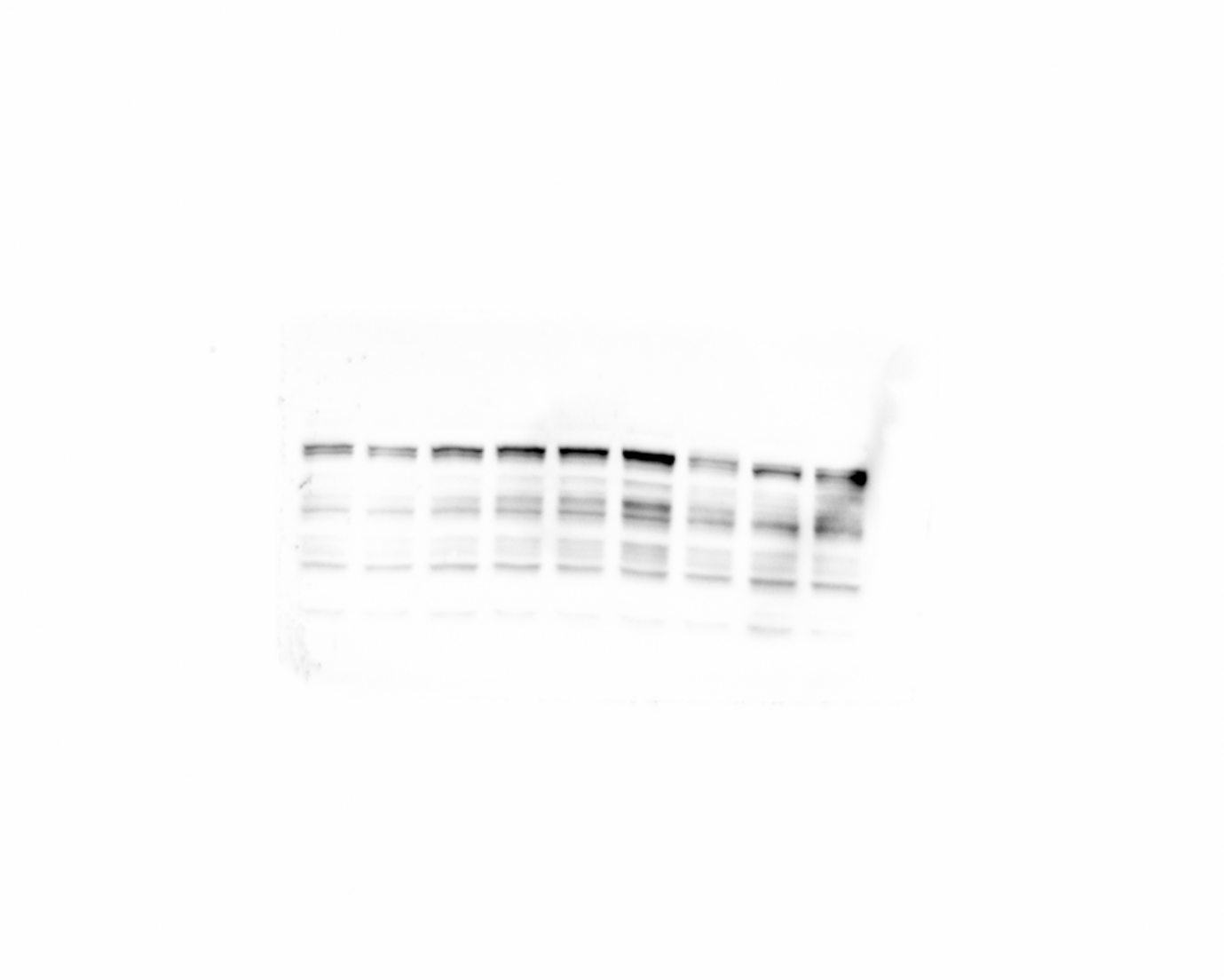

Supplement: Supplementary file 10 — Additional file 10. All Original and uncropped blots images used in manuscript. [file 12915_2022_1437_MOESM10_ESM.zip › blots images/Fig 4/Fig 4 C/HURP/Fig4C-HURP.tif]

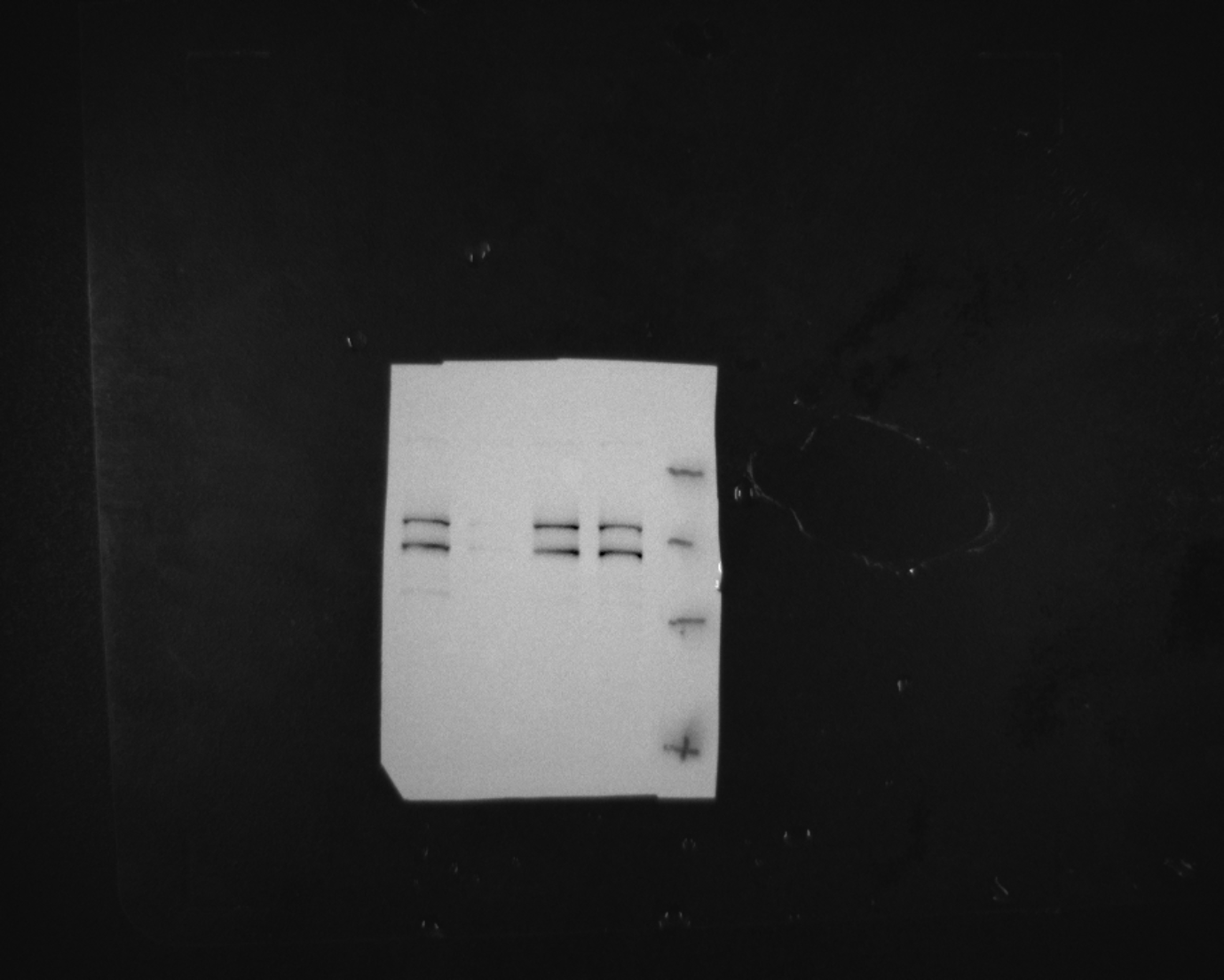

Supplement: Supplementary file 10 — Additional file 10. All Original and uncropped blots images used in manuscript. [file 12915_2022_1437_MOESM10_ESM.zip › blots images/Fig 5/Fig 5 C/astrin/Fig5C-astrin-marker.tif]

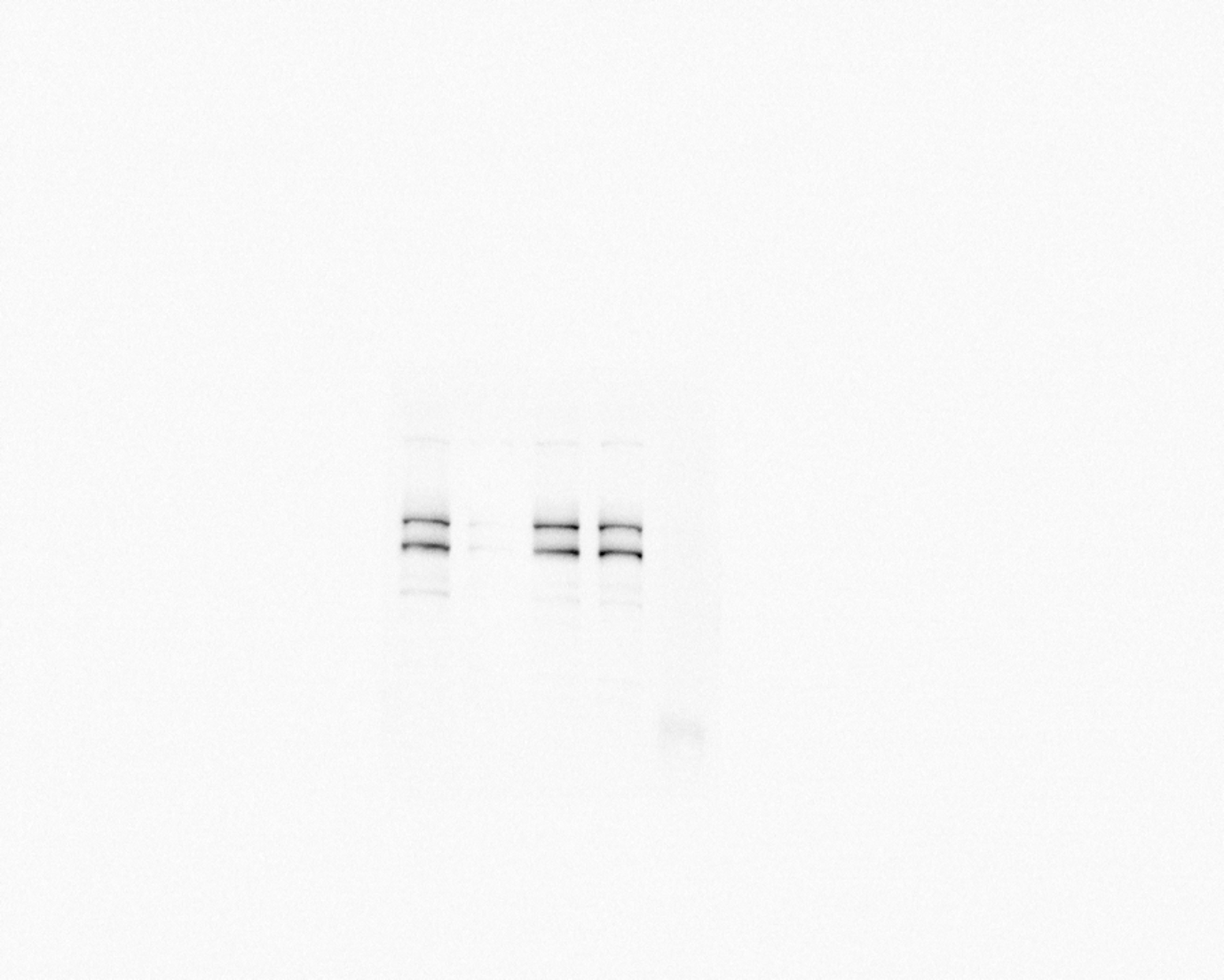

Supplement: Supplementary file 10 — Additional file 10. All Original and uncropped blots images used in manuscript. [file 12915_2022_1437_MOESM10_ESM.zip › blots images/Fig 5/Fig 5 C/astrin/Fig5C-astrin.tif]

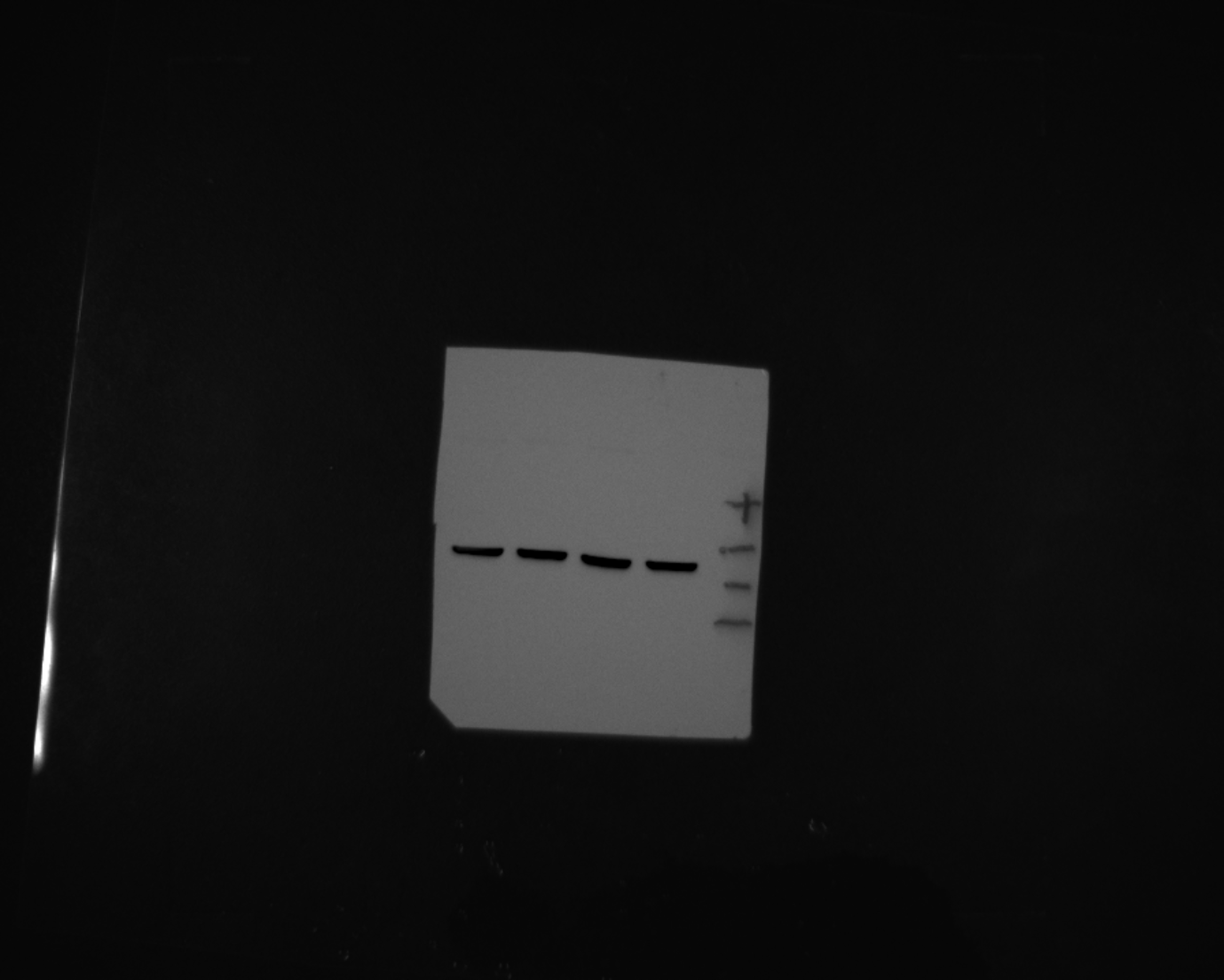

Supplement: Supplementary file 10 — Additional file 10. All Original and uncropped blots images used in manuscript. [file 12915_2022_1437_MOESM10_ESM.zip › blots images/Fig 5/Fig 5 C/beta-actin/Fig5C-beta-actin.-marker.tif]

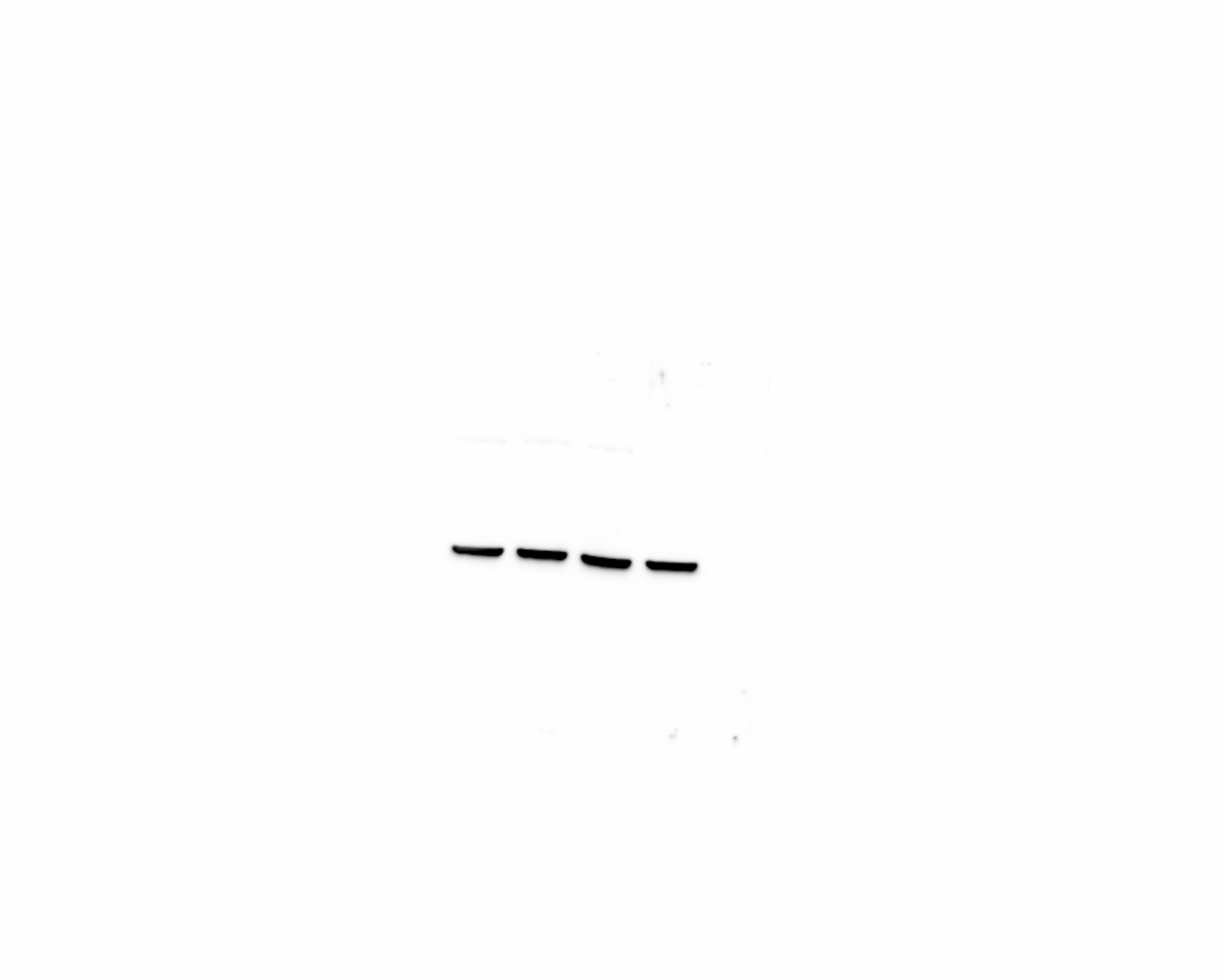

Supplement: Supplementary file 10 — Additional file 10. All Original and uncropped blots images used in manuscript. [file 12915_2022_1437_MOESM10_ESM.zip › blots images/Fig 5/Fig 5 C/beta-actin/Fig5C-beta-actin.tif]

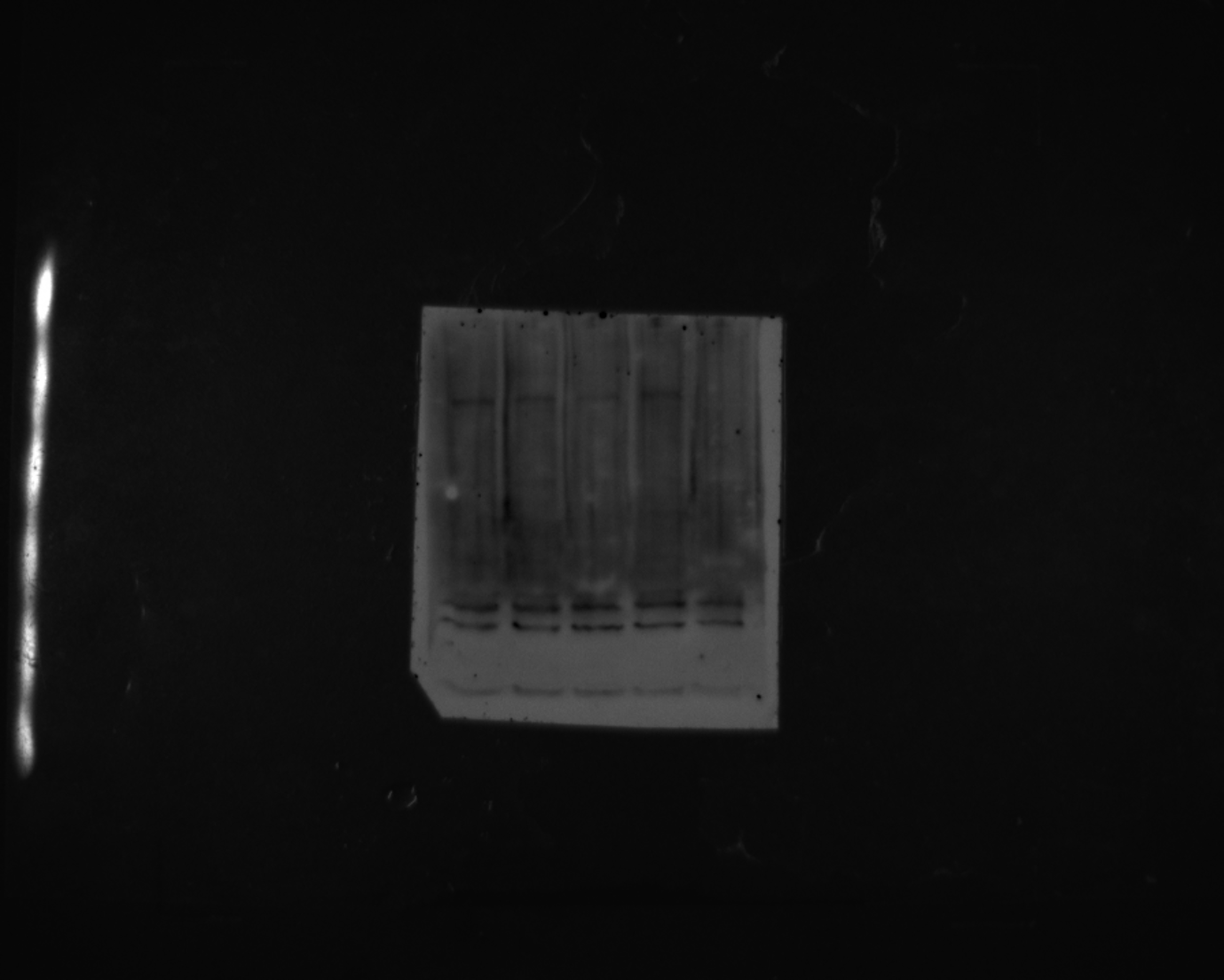

Supplement: Supplementary file 10 — Additional file 10. All Original and uncropped blots images used in manuscript. [file 12915_2022_1437_MOESM10_ESM.zip › blots images/Fig 5/Fig 5 C/CDK5RAP2/FIg5C-CDK5RAP2-marker.tif]

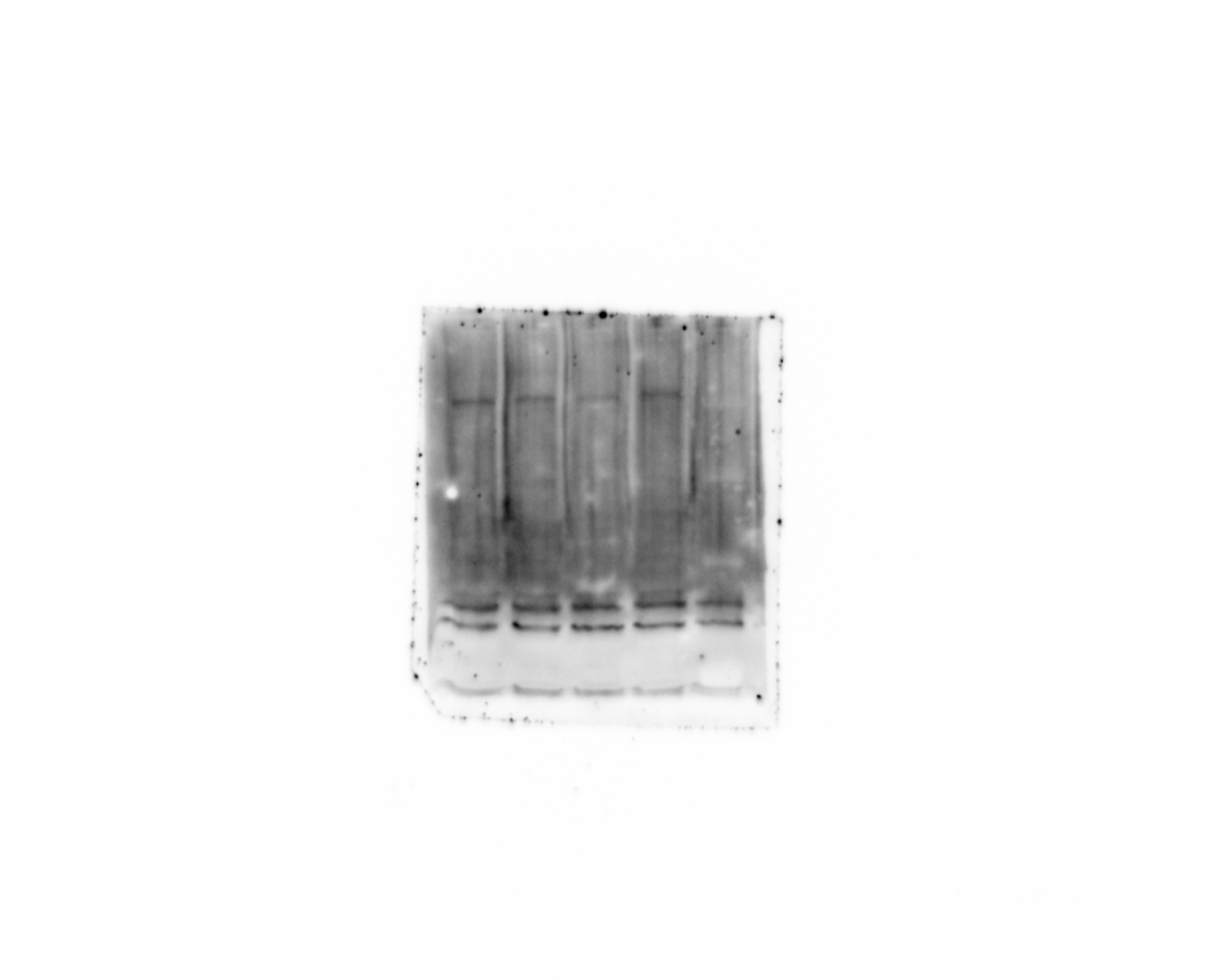

Supplement: Supplementary file 10 — Additional file 10. All Original and uncropped blots images used in manuscript. [file 12915_2022_1437_MOESM10_ESM.zip › blots images/Fig 5/Fig 5 C/CDK5RAP2/FIg5C-CDK5RAP2.tif]

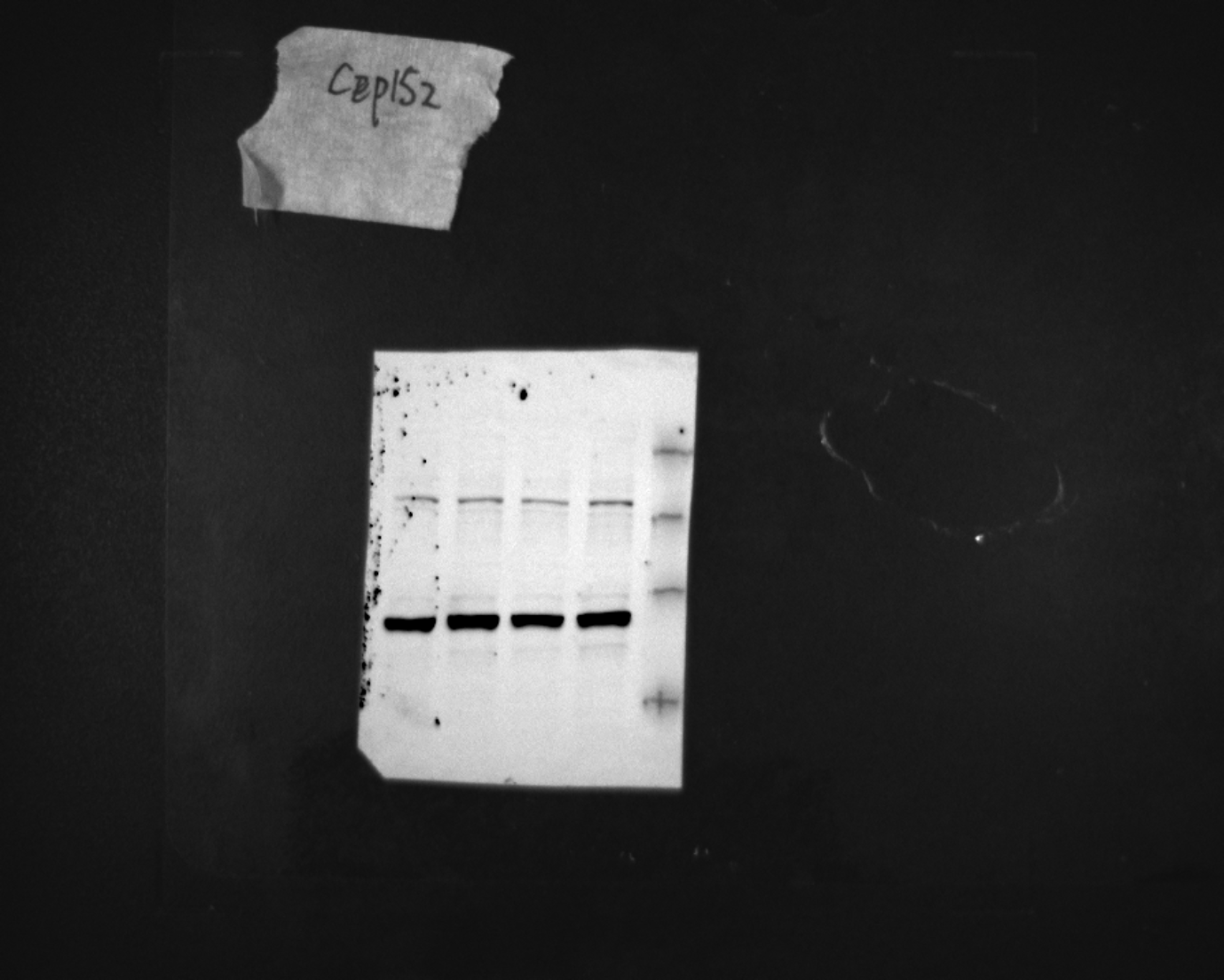

Supplement: Supplementary file 10 — Additional file 10. All Original and uncropped blots images used in manuscript. [file 12915_2022_1437_MOESM10_ESM.zip › blots images/Fig 5/Fig 5 C/CEP152/Fig5C-CEP152-marker.tif]

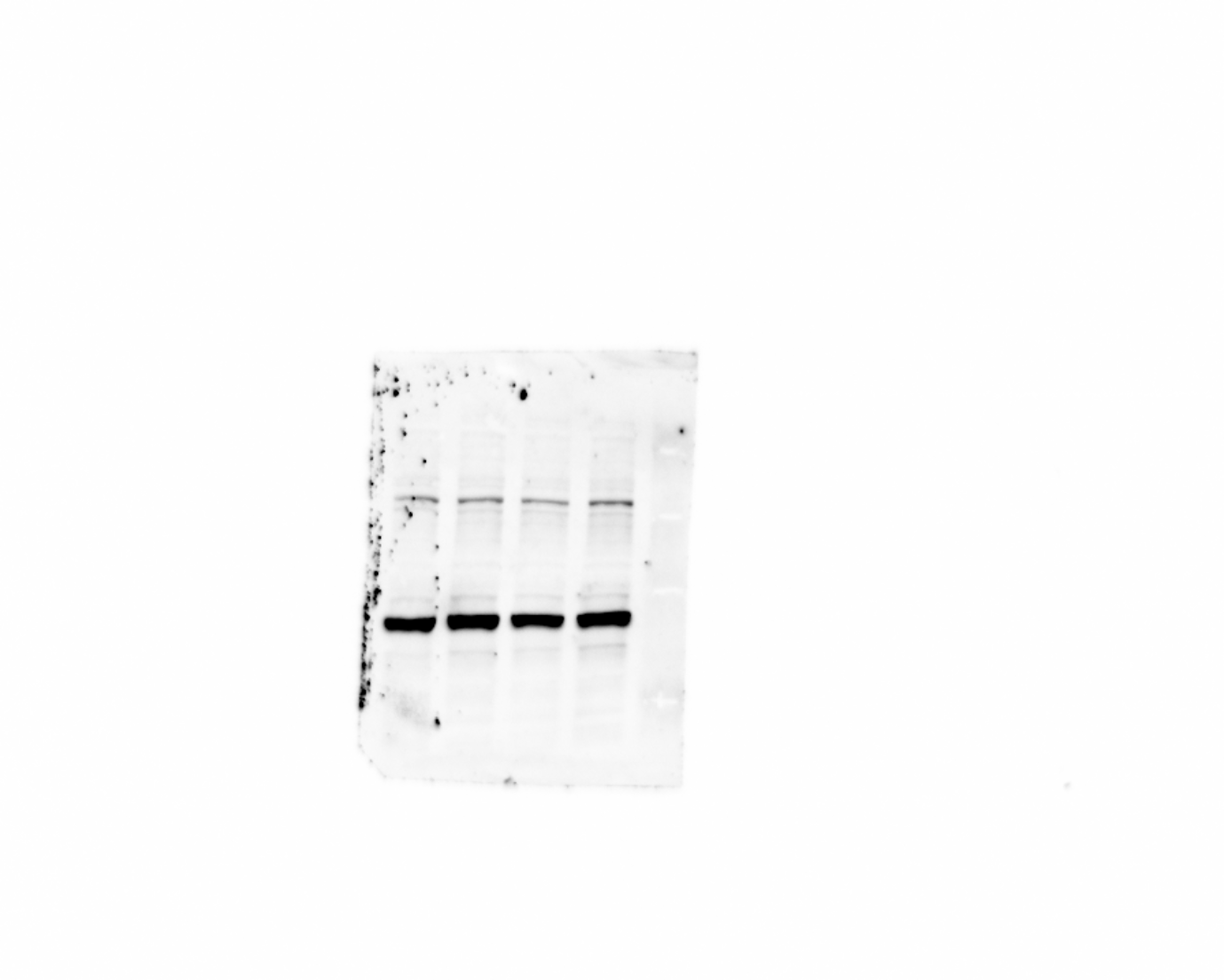

Supplement: Supplementary file 10 — Additional file 10. All Original and uncropped blots images used in manuscript. [file 12915_2022_1437_MOESM10_ESM.zip › blots images/Fig 5/Fig 5 C/CEP152/Fig5C-CEP152.tif]

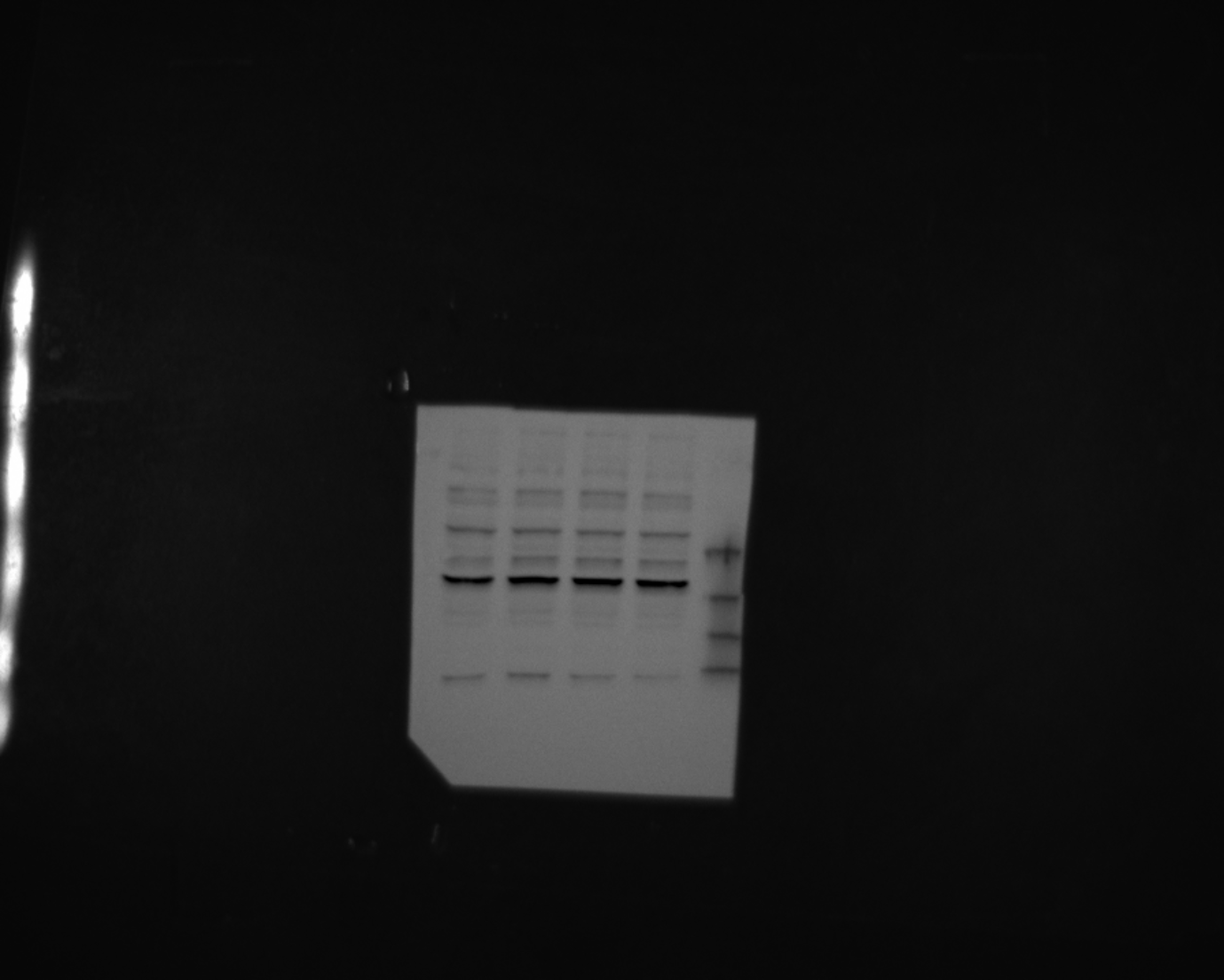

Supplement: Supplementary file 10 — Additional file 10. All Original and uncropped blots images used in manuscript. [file 12915_2022_1437_MOESM10_ESM.zip › blots images/Fig 5/Fig 5 C/CEP63/Fig5C-CEP63-marker.tif]

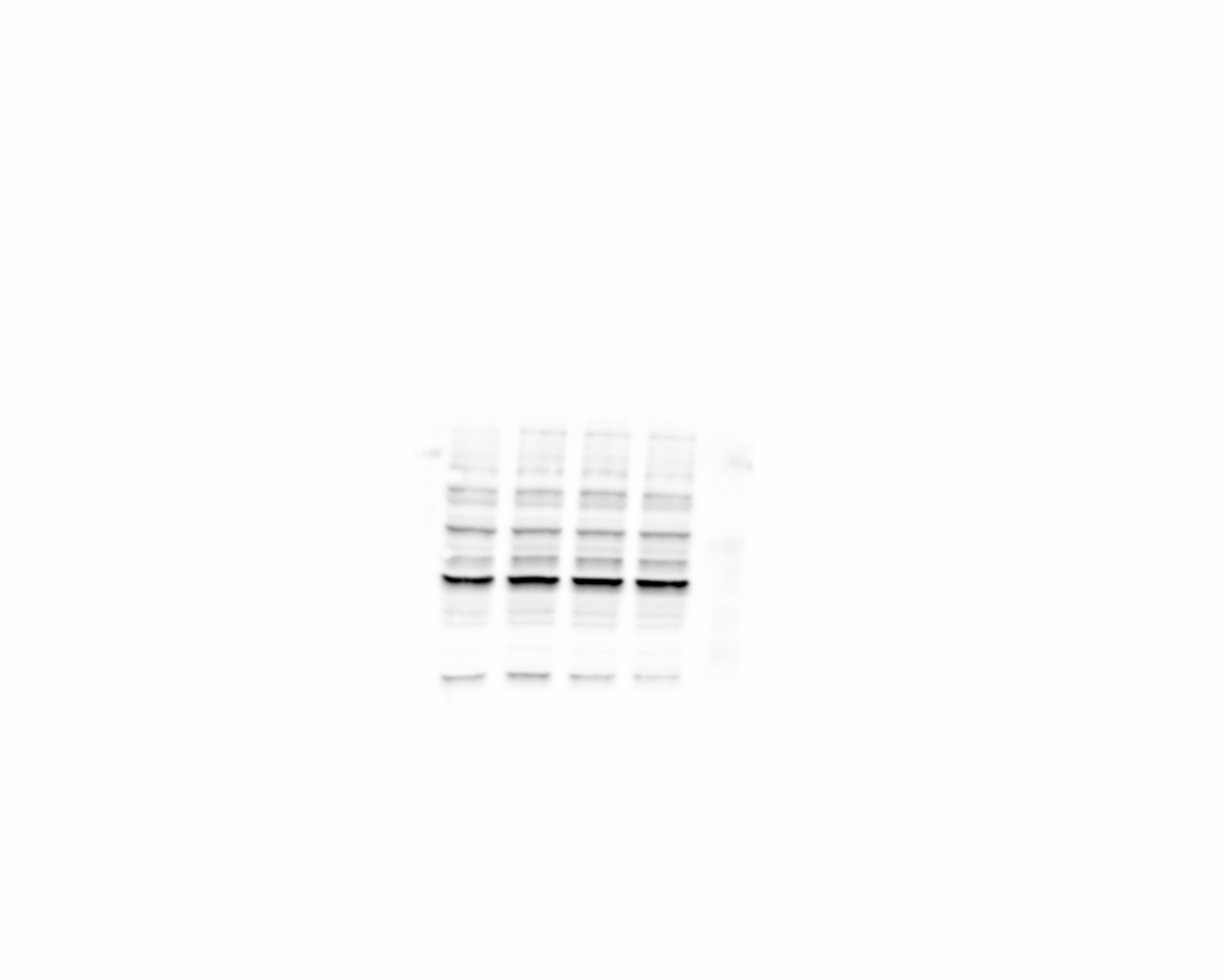

Supplement: Supplementary file 10 — Additional file 10. All Original and uncropped blots images used in manuscript. [file 12915_2022_1437_MOESM10_ESM.zip › blots images/Fig 5/Fig 5 C/CEP63/Fig5C-CEP63.tif]

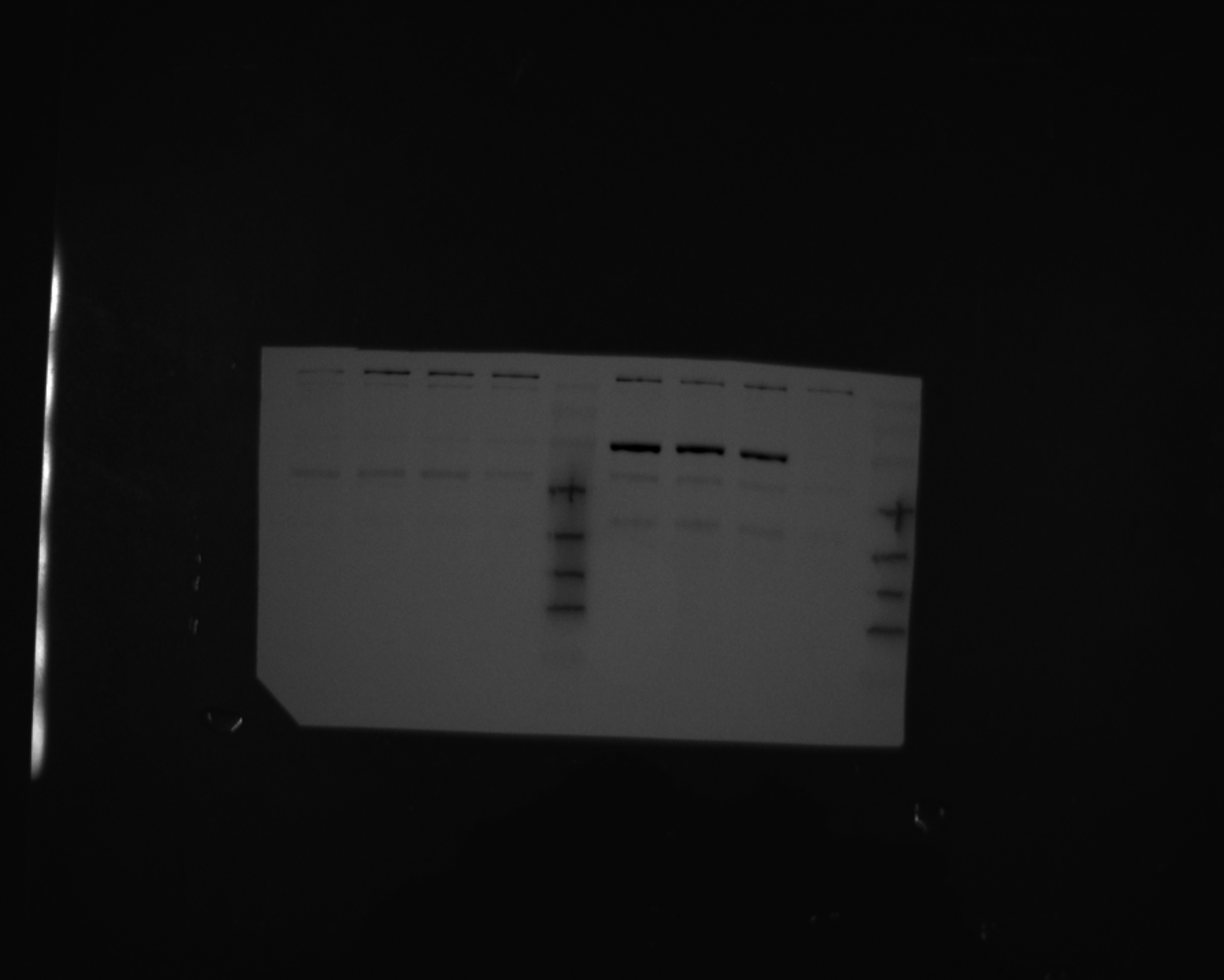

Supplement: Supplementary file 10 — Additional file 10. All Original and uncropped blots images used in manuscript. [file 12915_2022_1437_MOESM10_ESM.zip › blots images/Fig 5/Fig 5 C/CEP72/Fig5C-Right-4-lane-siRNA-anti-CEP72-marker.tif]

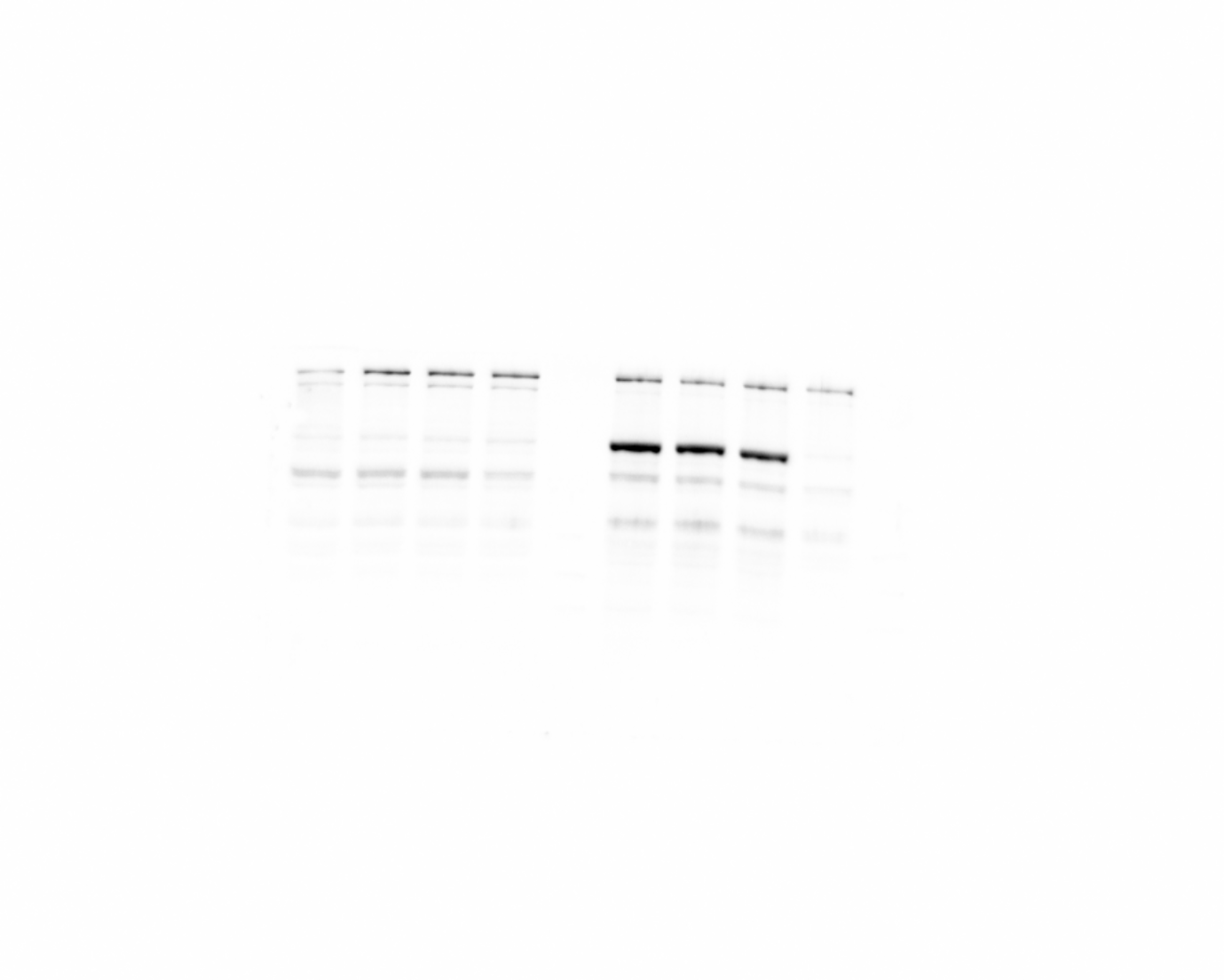

Supplement: Supplementary file 10 — Additional file 10. All Original and uncropped blots images used in manuscript. [file 12915_2022_1437_MOESM10_ESM.zip › blots images/Fig 5/Fig 5 C/CEP72/Fig5C-Right-4-lane-siRNA-anti-CEP72.tif]

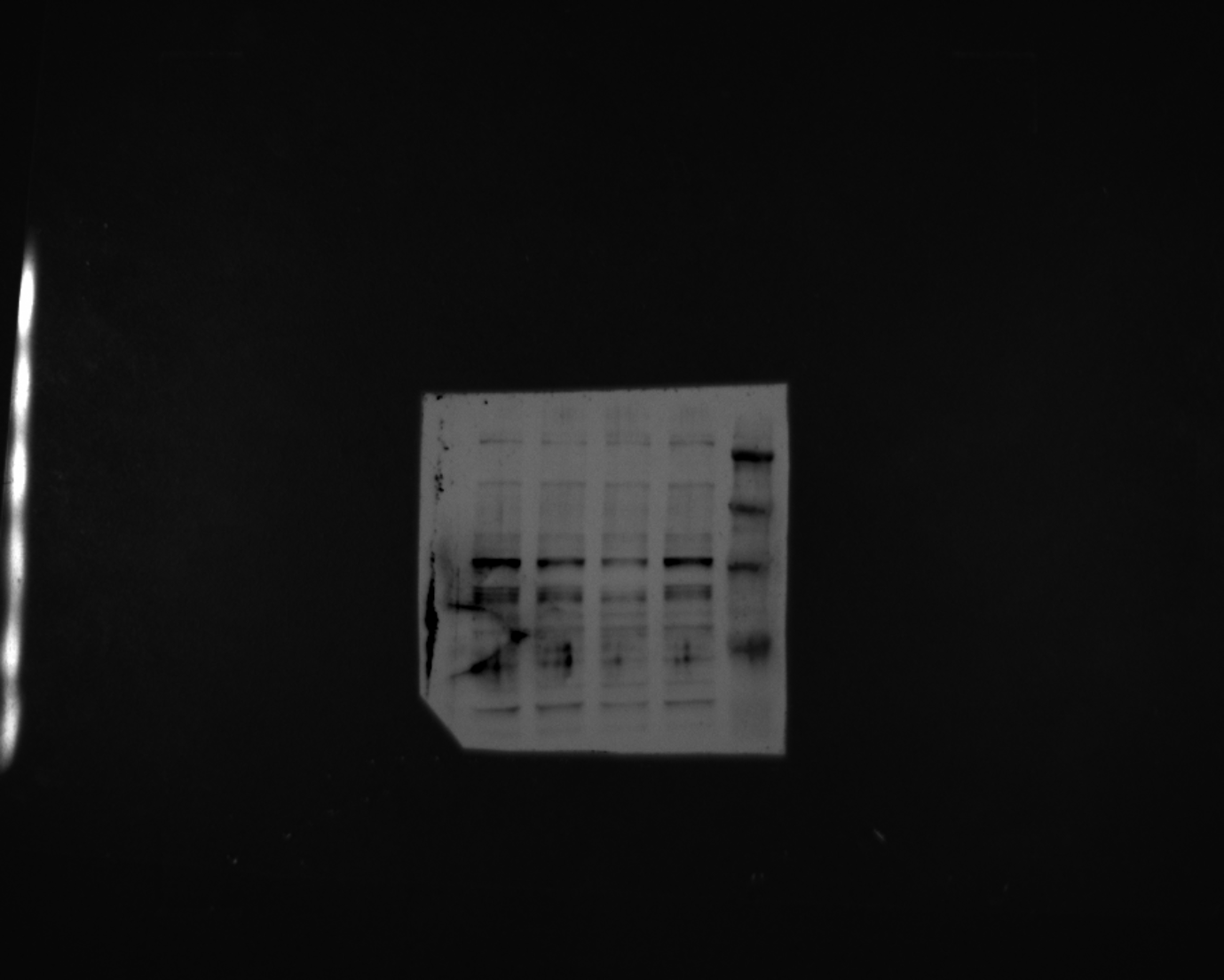

Supplement: Supplementary file 10 — Additional file 10. All Original and uncropped blots images used in manuscript. [file 12915_2022_1437_MOESM10_ESM.zip › blots images/Fig 5/Fig 5 C/HCR/Fig5C-HCR-marker.tif]

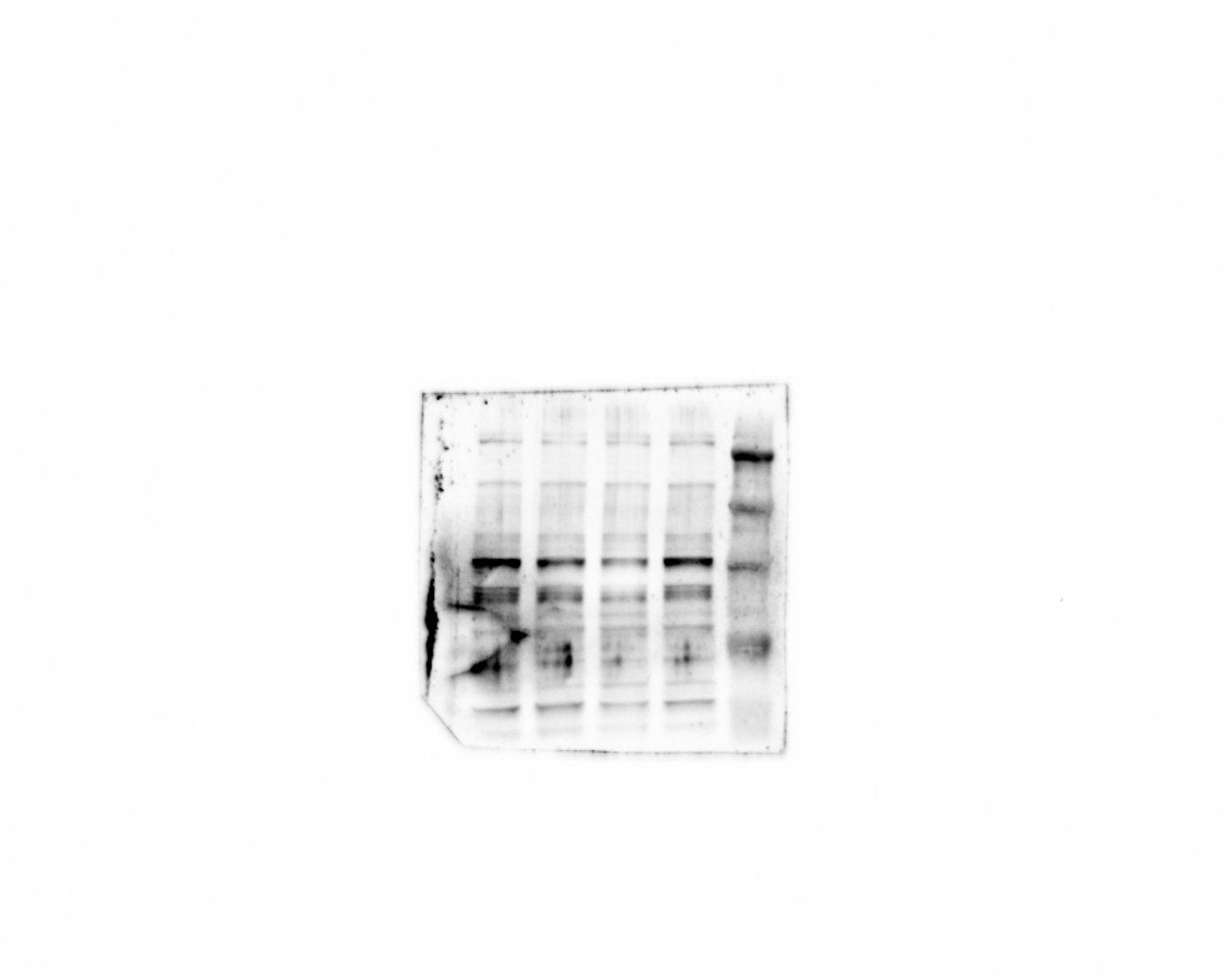

Supplement: Supplementary file 10 — Additional file 10. All Original and uncropped blots images used in manuscript. [file 12915_2022_1437_MOESM10_ESM.zip › blots images/Fig 5/Fig 5 C/HCR/FIg5C-HCR.tif]

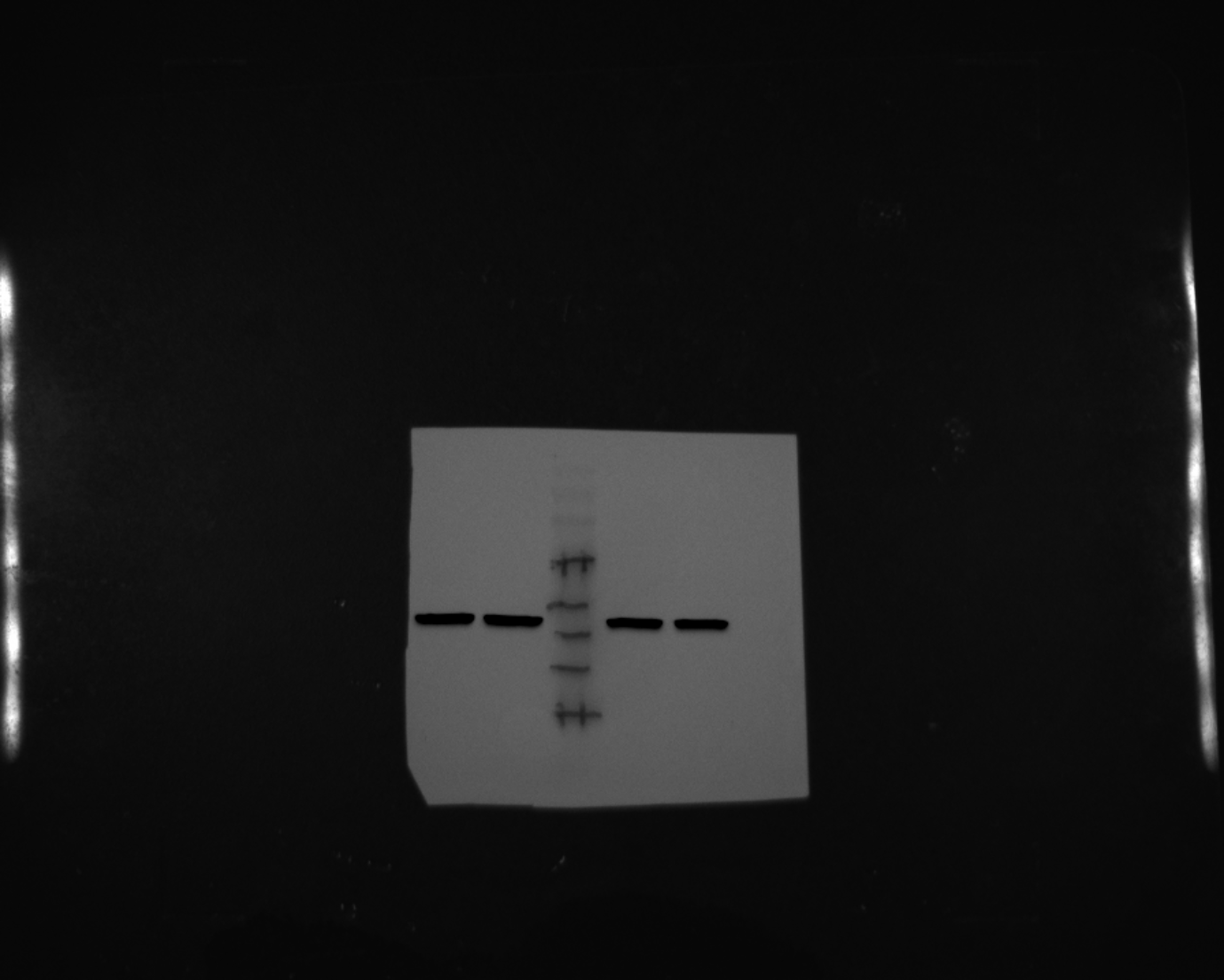

Supplement: Supplementary file 10 — Additional file 10. All Original and uncropped blots images used in manuscript. [file 12915_2022_1437_MOESM10_ESM.zip › blots images/Fig 5/Fig 5 E/left-siCEP152-right-siCEP63-beta-actin-marker.tif]

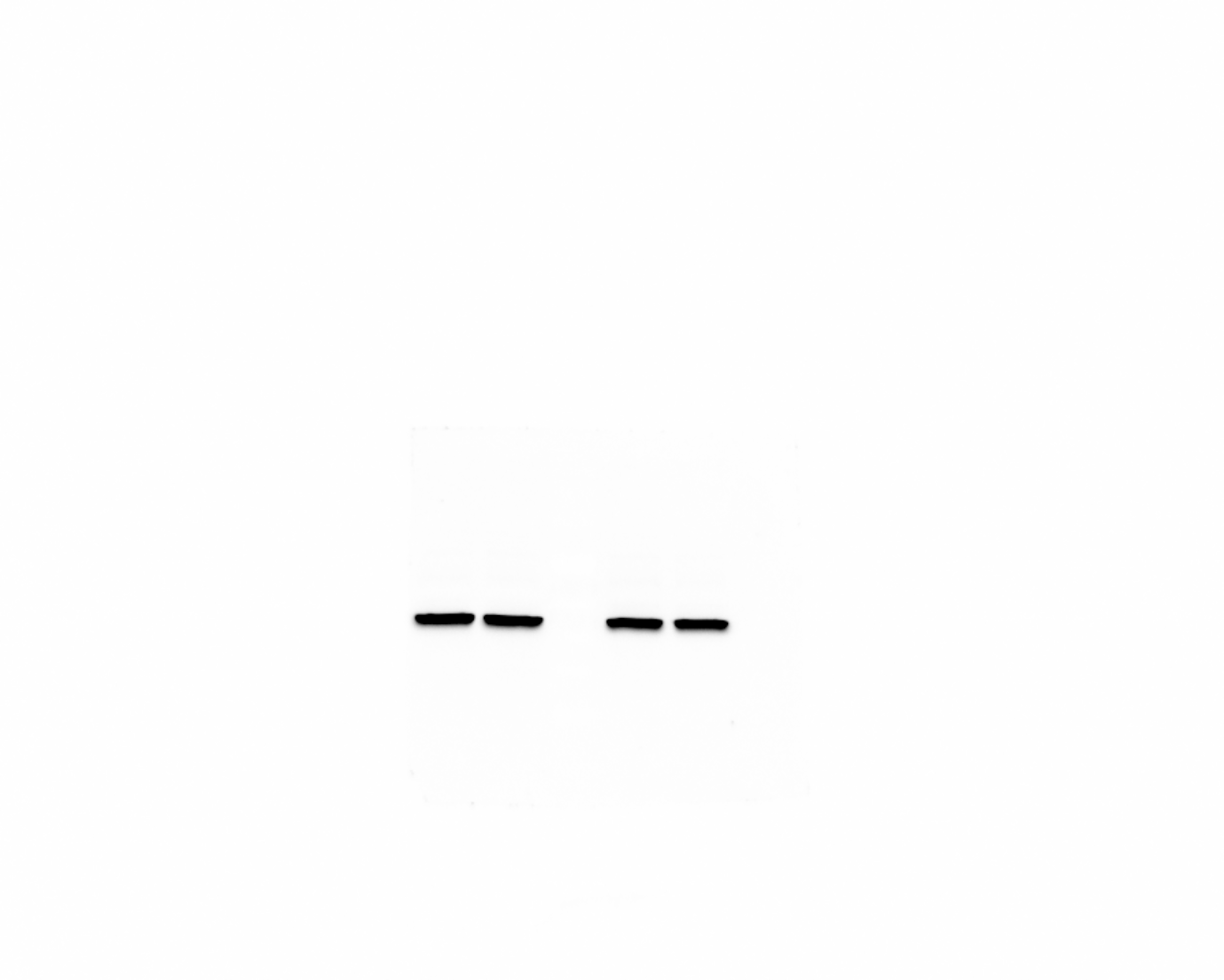

Supplement: Supplementary file 10 — Additional file 10. All Original and uncropped blots images used in manuscript. [file 12915_2022_1437_MOESM10_ESM.zip › blots images/Fig 5/Fig 5 E/left-siCEP152-right-siCEP63-beta-actin.tif]

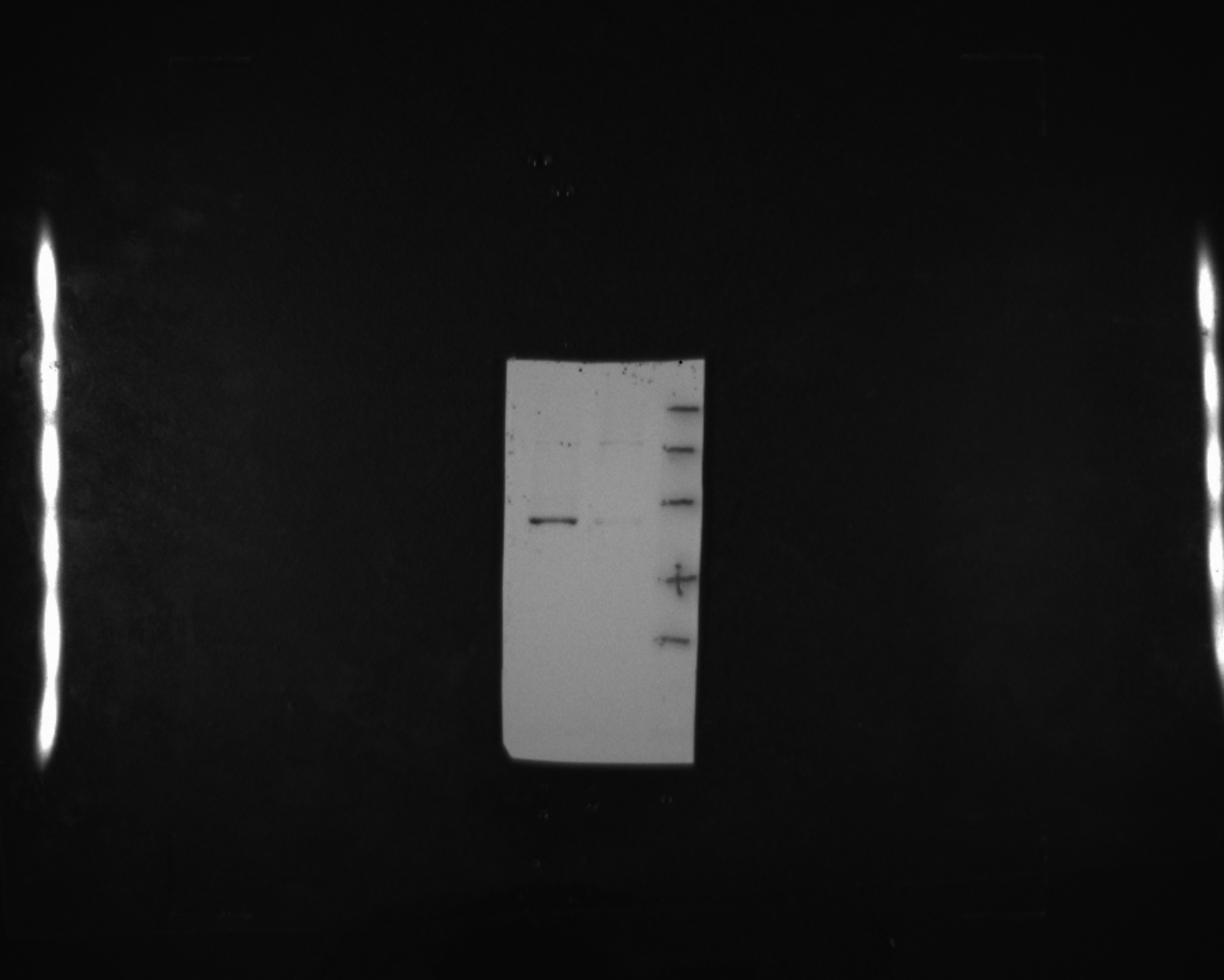

Supplement: Supplementary file 10 — Additional file 10. All Original and uncropped blots images used in manuscript. [file 12915_2022_1437_MOESM10_ESM.zip › blots images/Fig 5/Fig 5 E/siCEP152/Fig5E-siCEP152-CEP152-marker.tif]

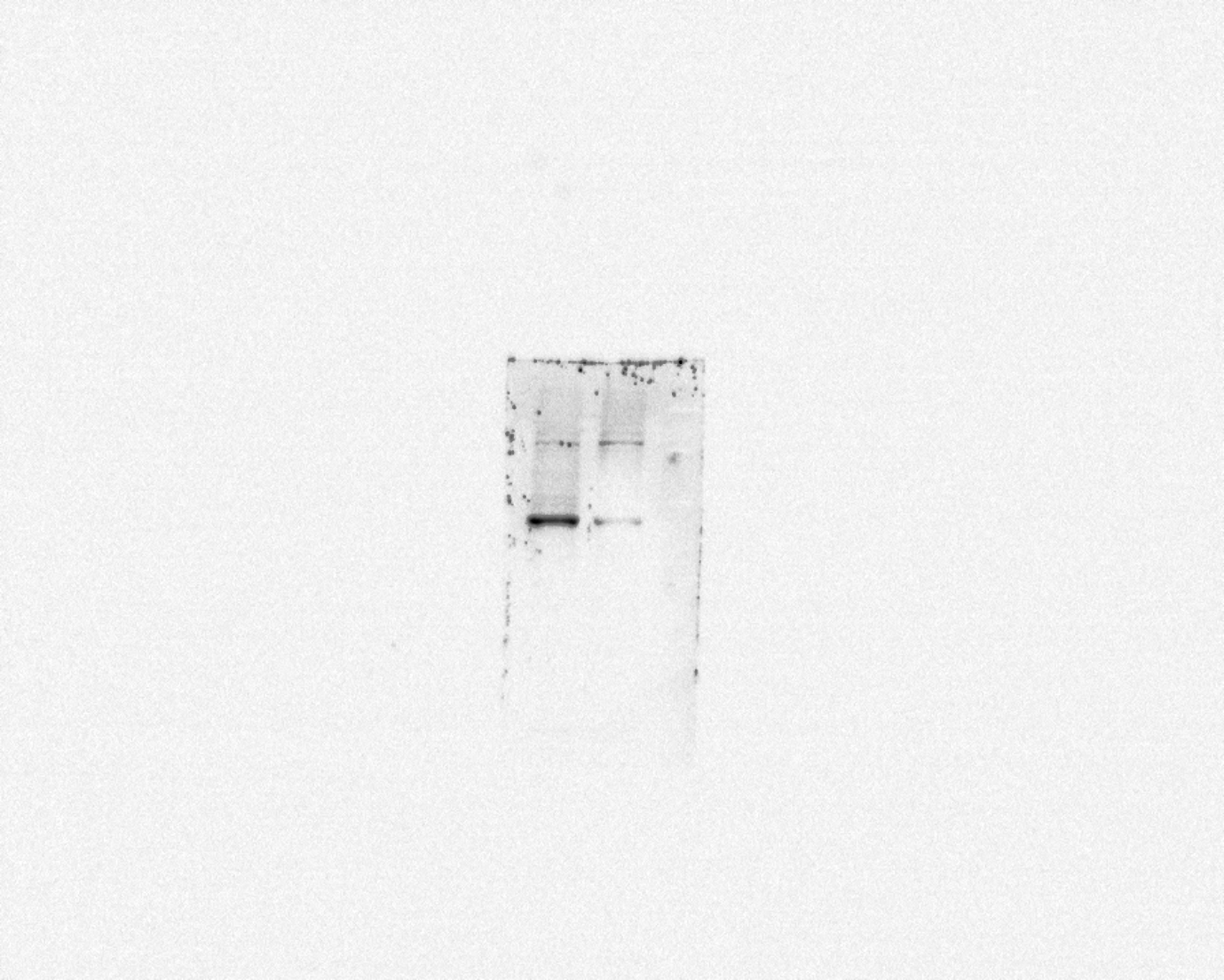

Supplement: Supplementary file 10 — Additional file 10. All Original and uncropped blots images used in manuscript. [file 12915_2022_1437_MOESM10_ESM.zip › blots images/Fig 5/Fig 5 E/siCEP152/Fig5E-siCEP152-CEP152.tif]

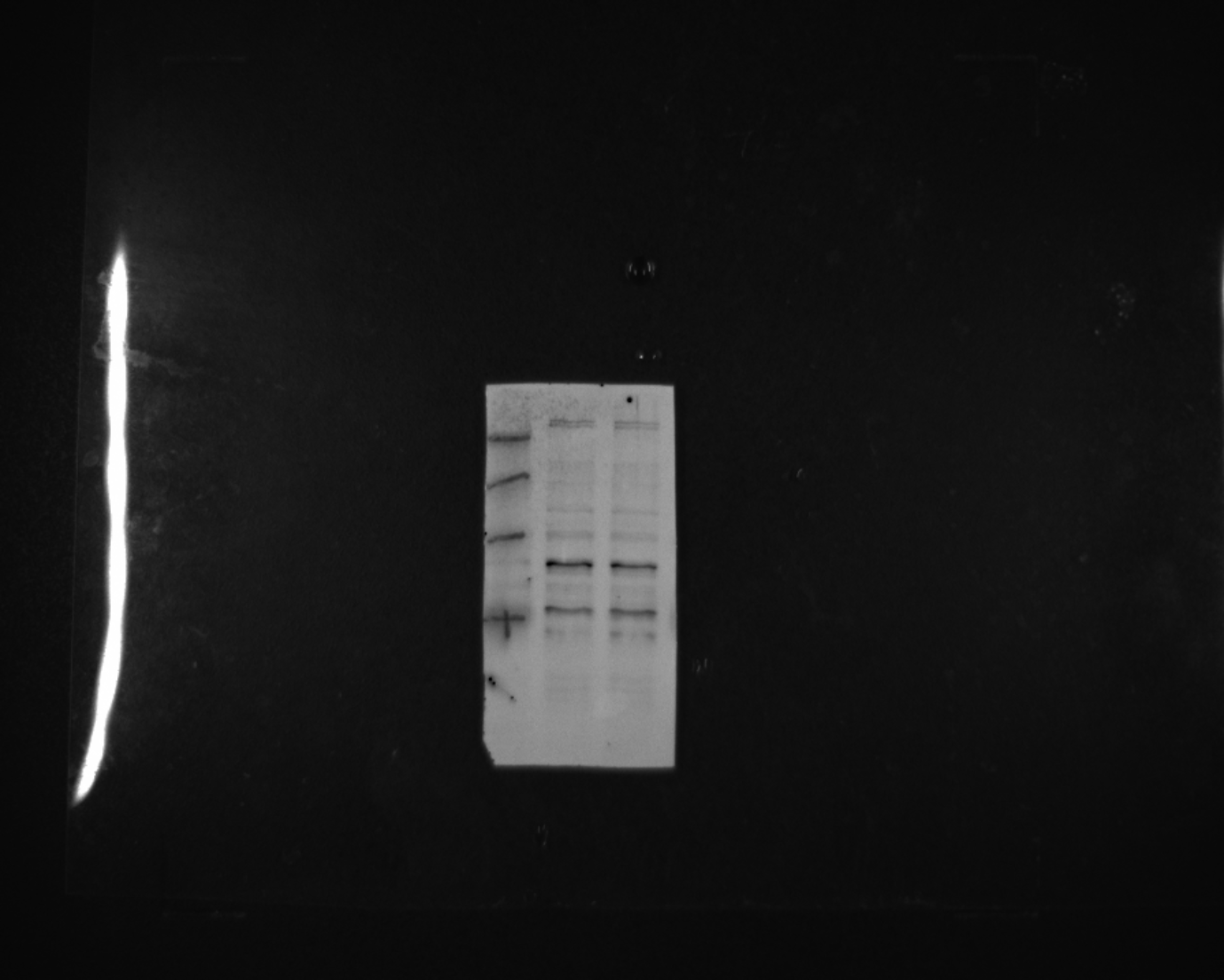

Supplement: Supplementary file 10 — Additional file 10. All Original and uncropped blots images used in manuscript. [file 12915_2022_1437_MOESM10_ESM.zip › blots images/Fig 5/Fig 5 E/siCEP152/Fig5E-siCEP152-HCR-marker.tif]

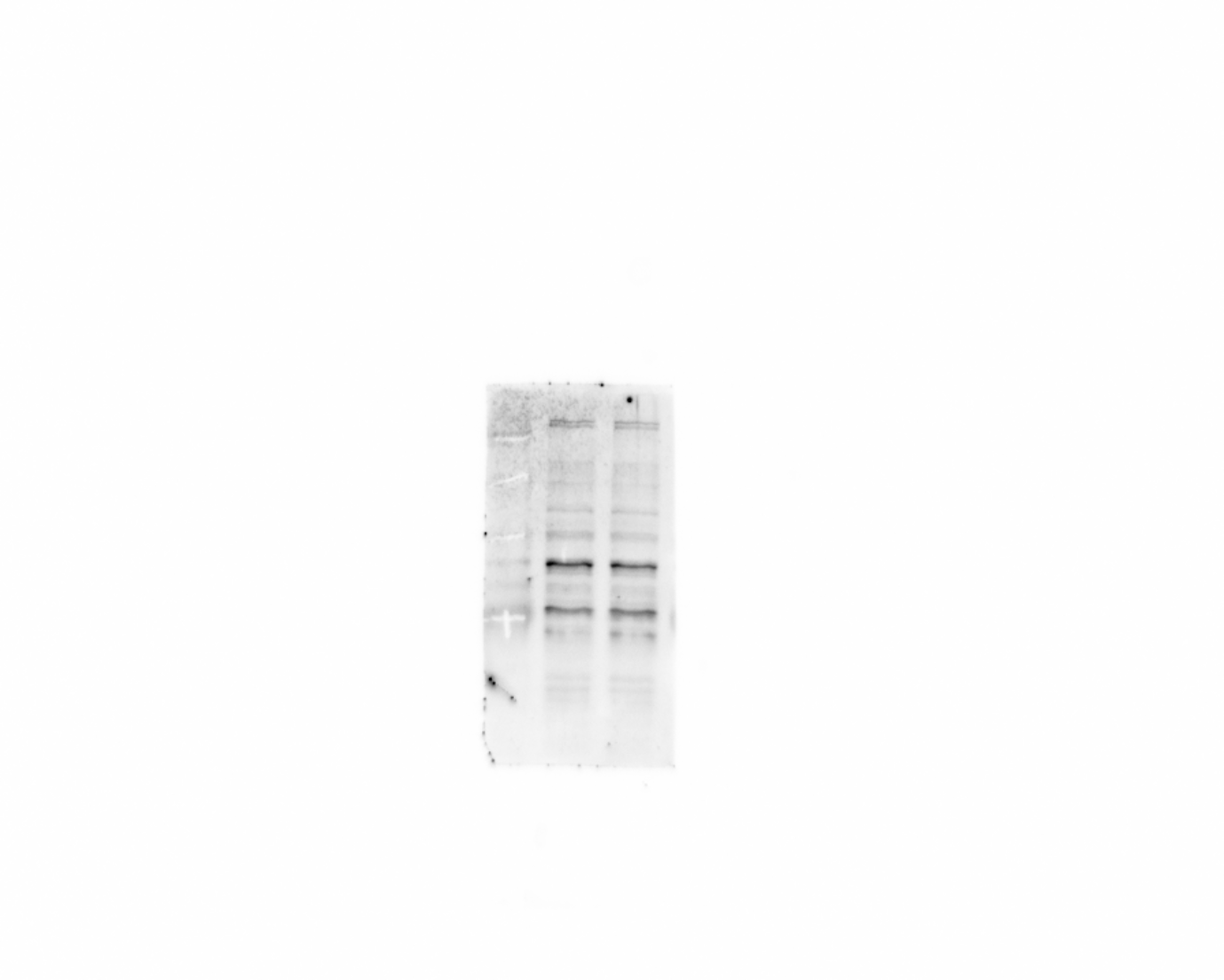

Supplement: Supplementary file 10 — Additional file 10. All Original and uncropped blots images used in manuscript. [file 12915_2022_1437_MOESM10_ESM.zip › blots images/Fig 5/Fig 5 E/siCEP152/Fig5E-siCEP152-HCR.tif]

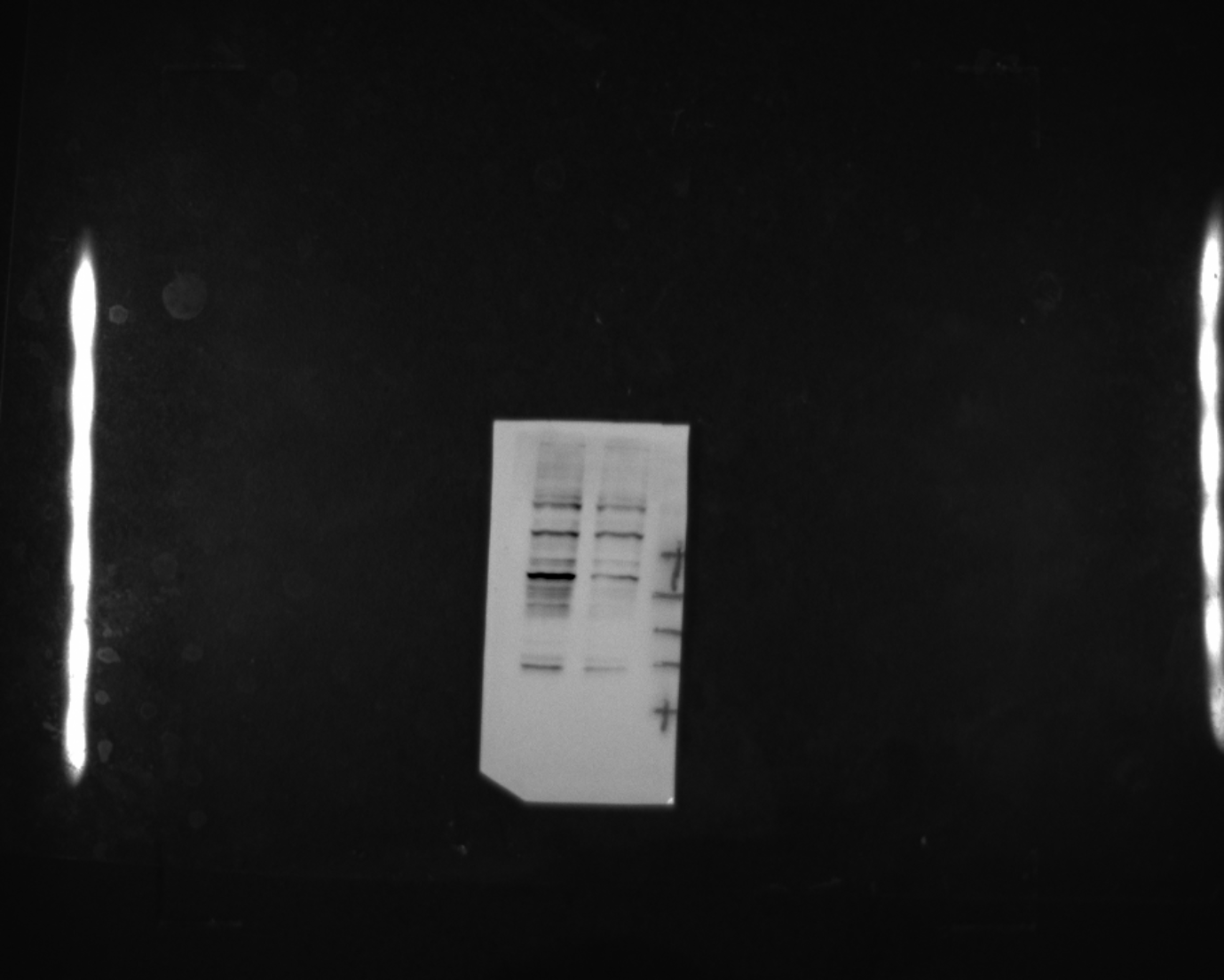

Supplement: Supplementary file 10 — Additional file 10. All Original and uncropped blots images used in manuscript. [file 12915_2022_1437_MOESM10_ESM.zip › blots images/Fig 5/Fig 5 E/siCEP63/Fig5E-siCEP63-CEP63-marker.tif]

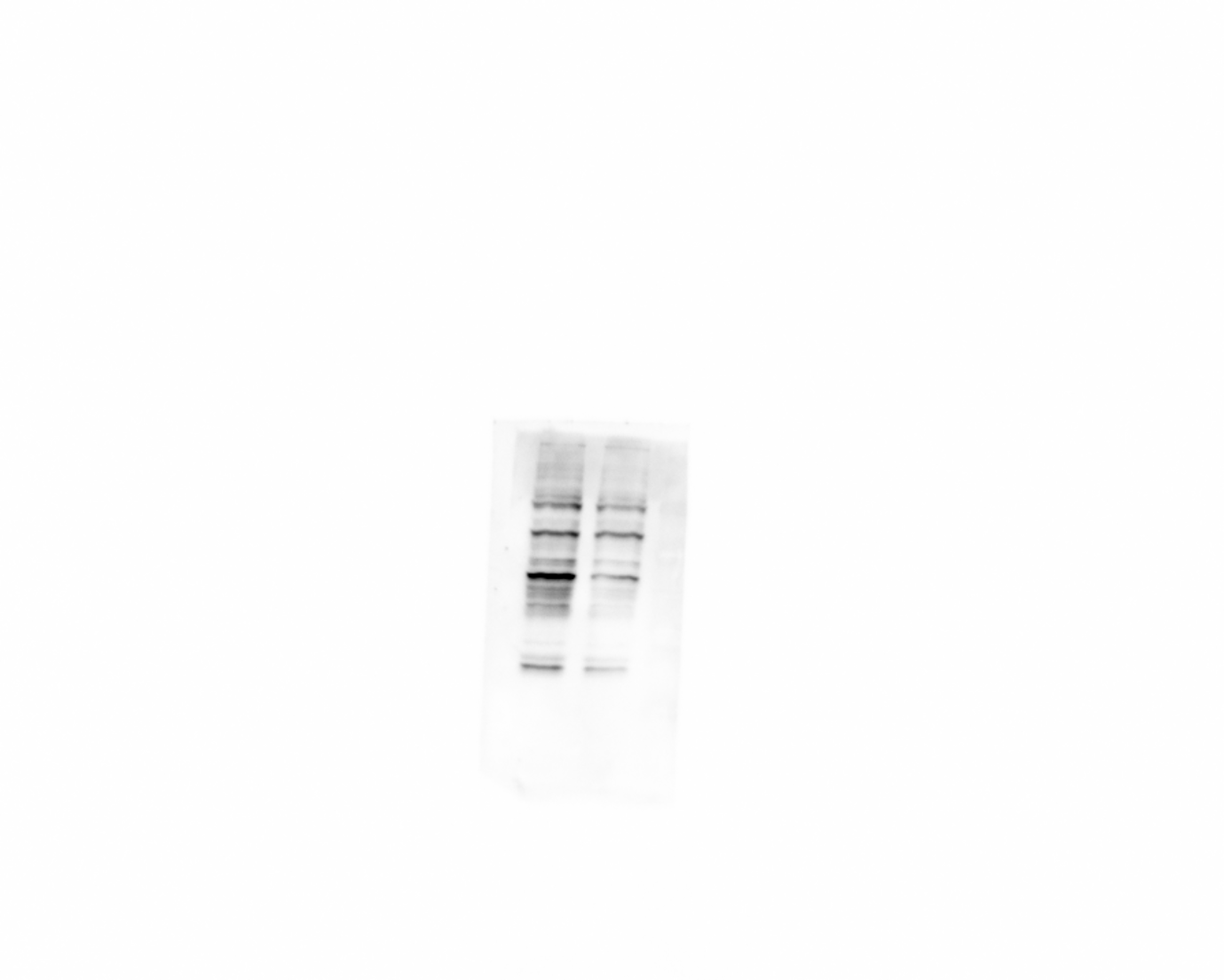

Supplement: Supplementary file 10 — Additional file 10. All Original and uncropped blots images used in manuscript. [file 12915_2022_1437_MOESM10_ESM.zip › blots images/Fig 5/Fig 5 E/siCEP63/Fig5E-siCEP63-CEP63.tif]

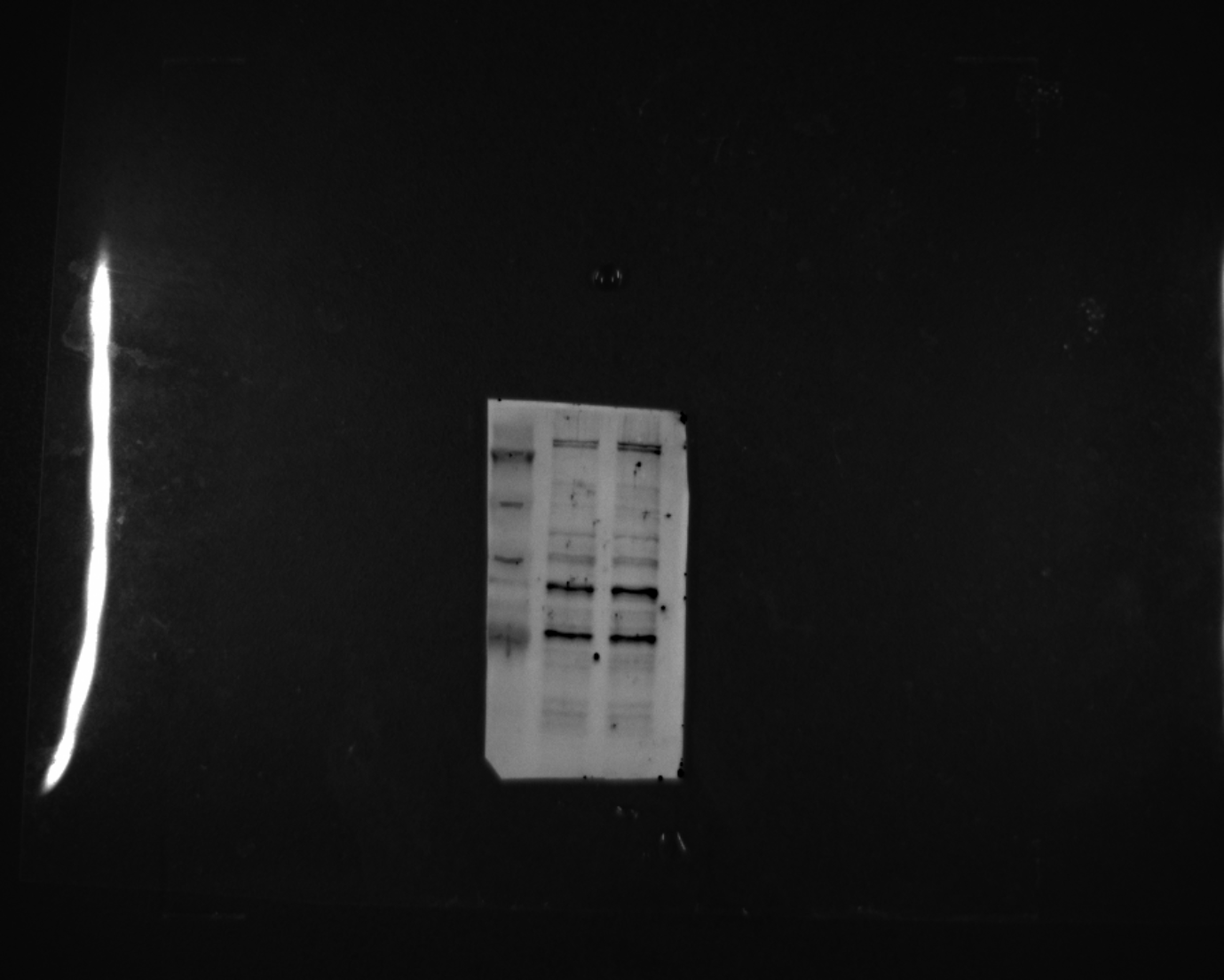

Supplement: Supplementary file 10 — Additional file 10. All Original and uncropped blots images used in manuscript. [file 12915_2022_1437_MOESM10_ESM.zip › blots images/Fig 5/Fig 5 E/siCEP63/Fig5E-siCEP63-HCR-marker.tif]

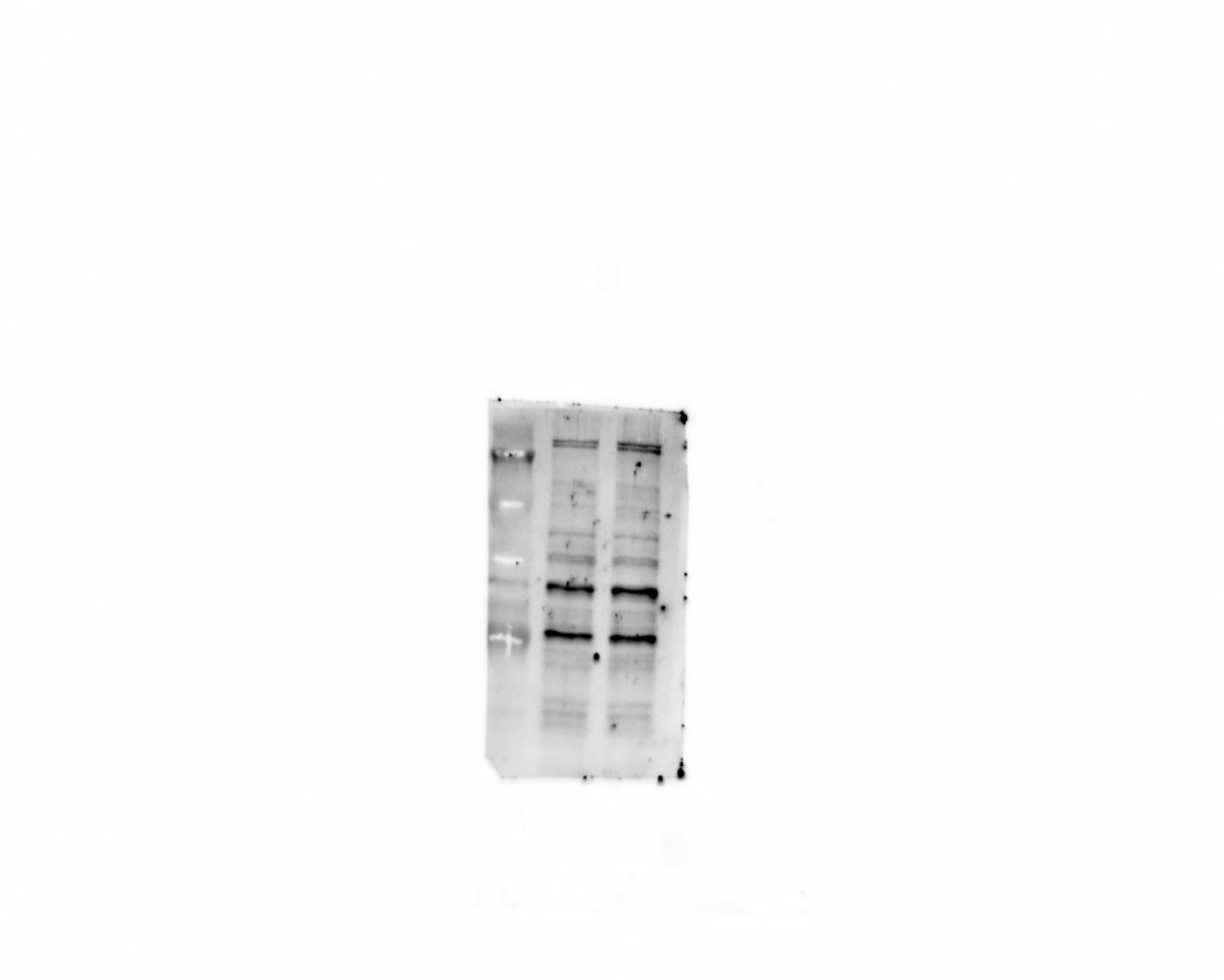

Supplement: Supplementary file 10 — Additional file 10. All Original and uncropped blots images used in manuscript. [file 12915_2022_1437_MOESM10_ESM.zip › blots images/Fig 5/Fig 5 E/siCEP63/Fig5E-siCEP63-HCR.tif]

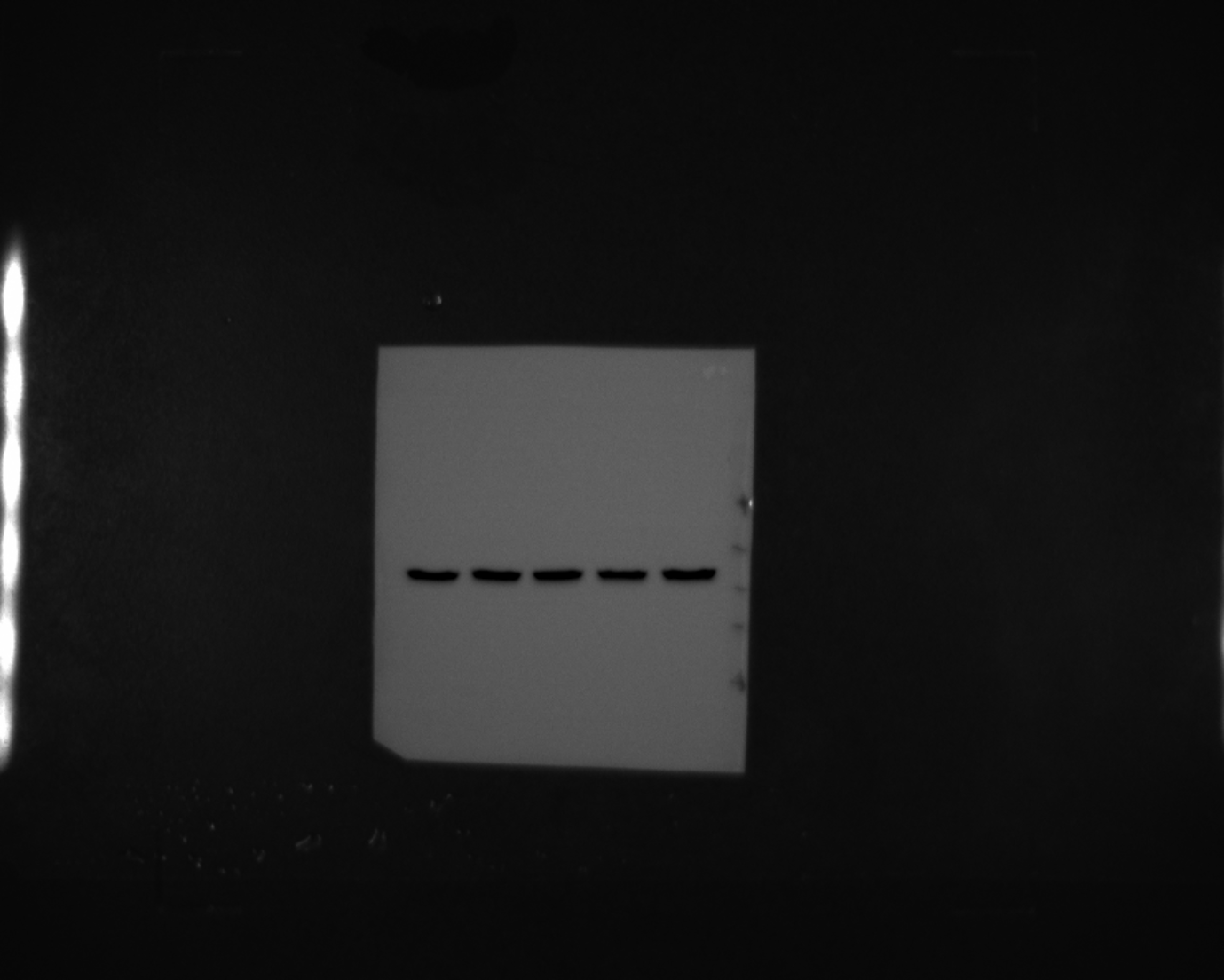

Supplement: Supplementary file 10 — Additional file 10. All Original and uncropped blots images used in manuscript. [file 12915_2022_1437_MOESM10_ESM.zip › blots images/Fig 7/Fig 7 C/beta-actin/Fig7C-beta-actin-marker.tif]

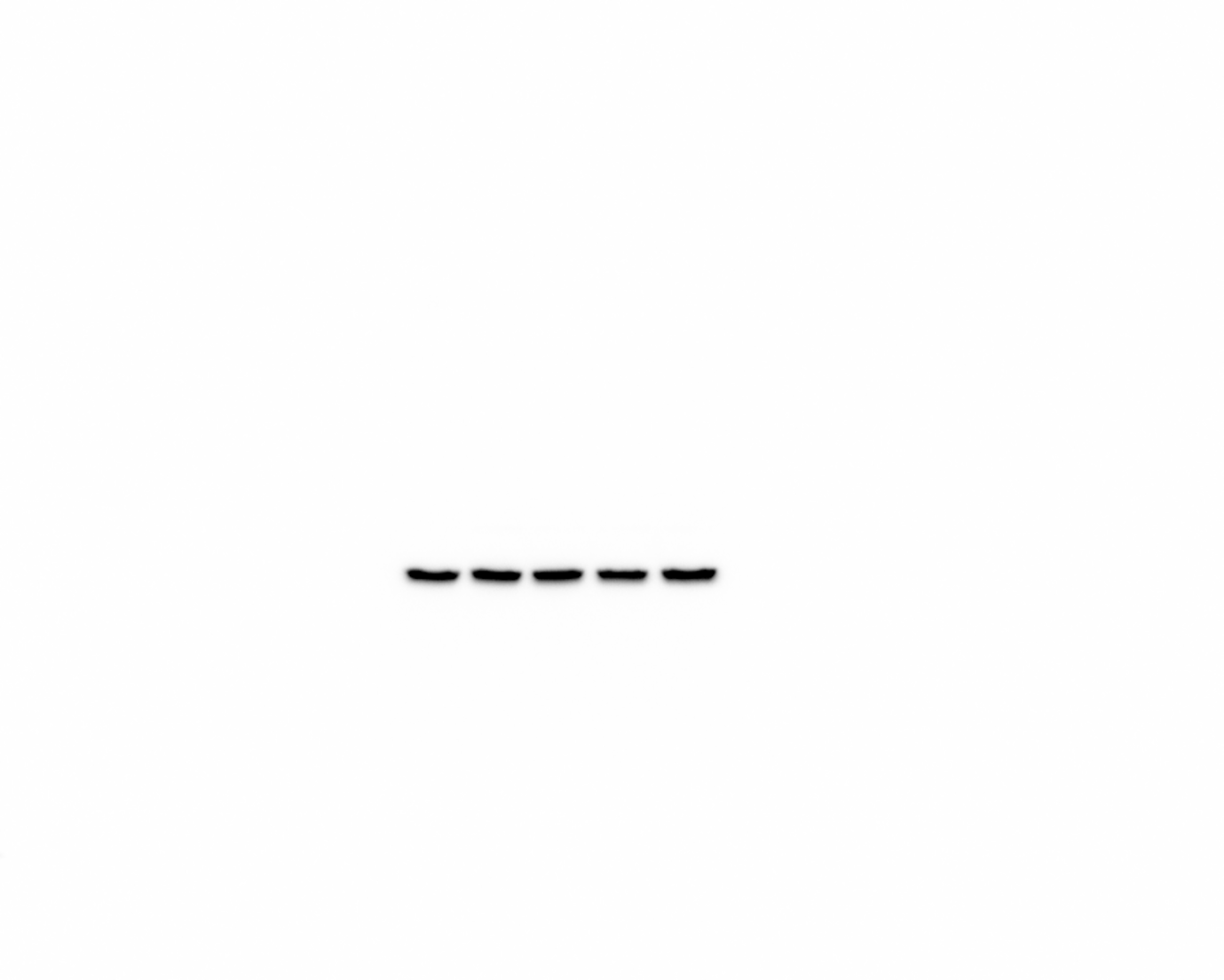

Supplement: Supplementary file 10 — Additional file 10. All Original and uncropped blots images used in manuscript. [file 12915_2022_1437_MOESM10_ESM.zip › blots images/Fig 7/Fig 7 C/beta-actin/Fig7C-beta-actin.tif]

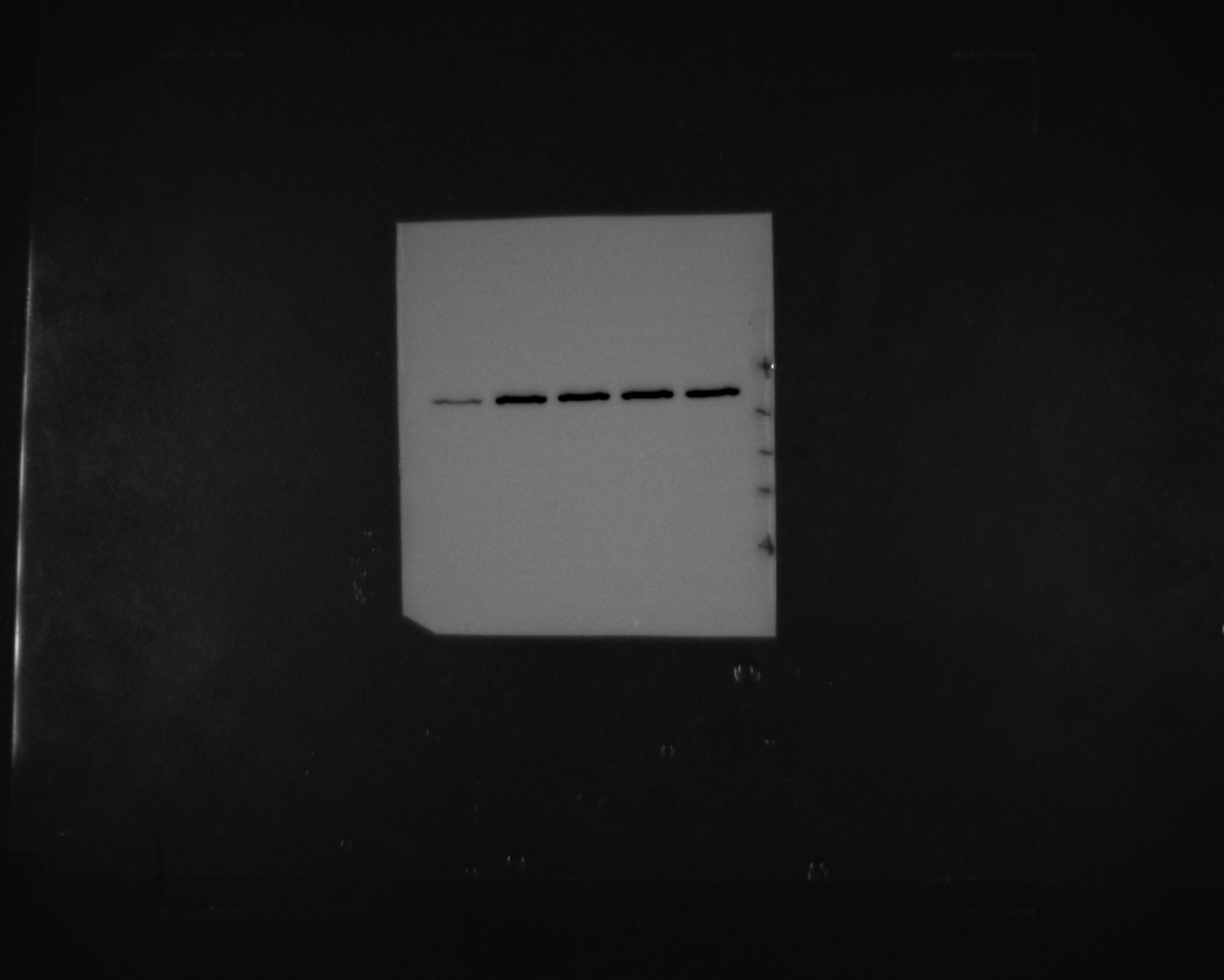

Supplement: Supplementary file 10 — Additional file 10. All Original and uncropped blots images used in manuscript. [file 12915_2022_1437_MOESM10_ESM.zip › blots images/Fig 7/Fig 7 C/cyclin B1/Fig7C-cyclinB1-marker.tif]

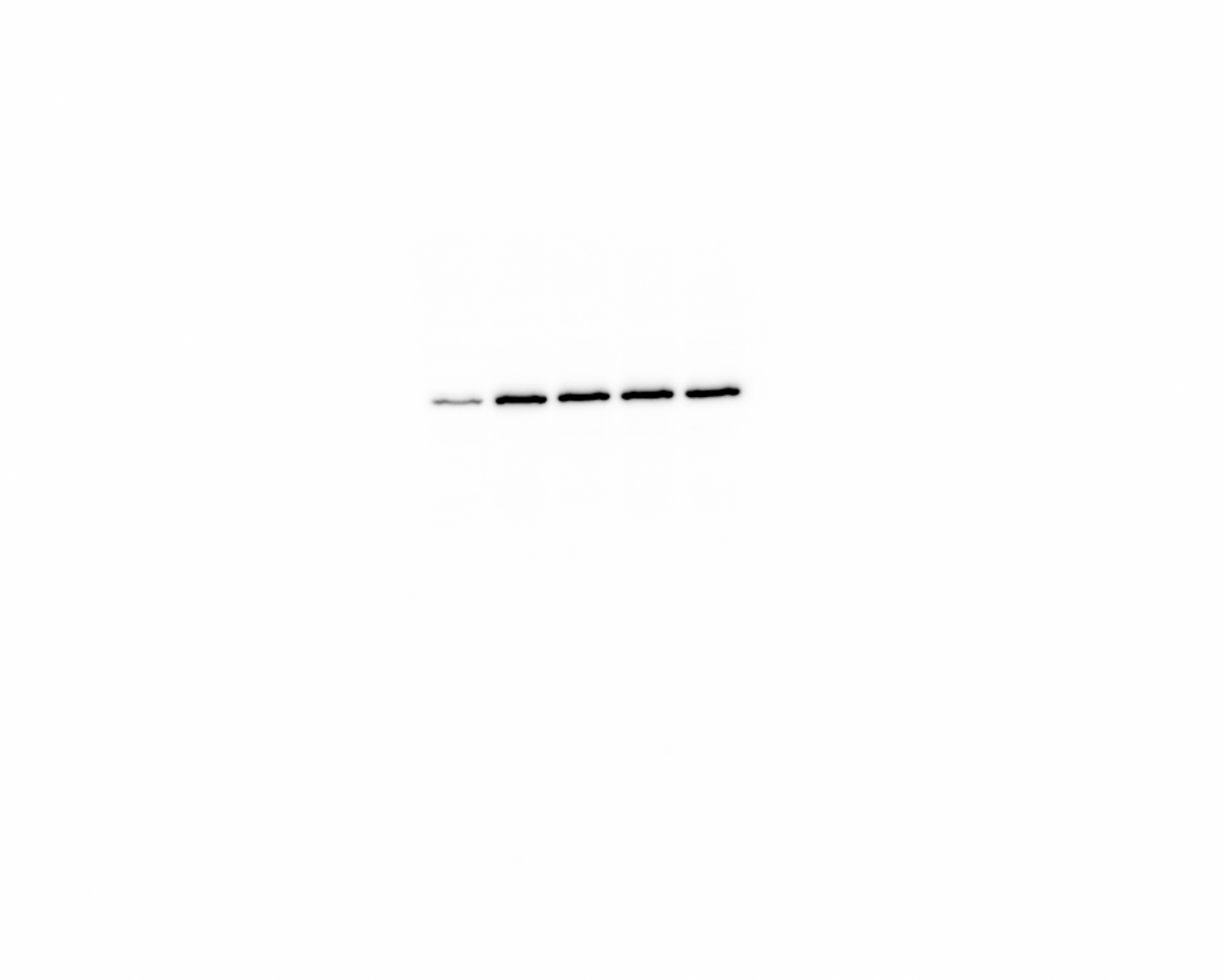

Supplement: Supplementary file 10 — Additional file 10. All Original and uncropped blots images used in manuscript. [file 12915_2022_1437_MOESM10_ESM.zip › blots images/Fig 7/Fig 7 C/cyclin B1/Fig7C-cyclinB1.tif]

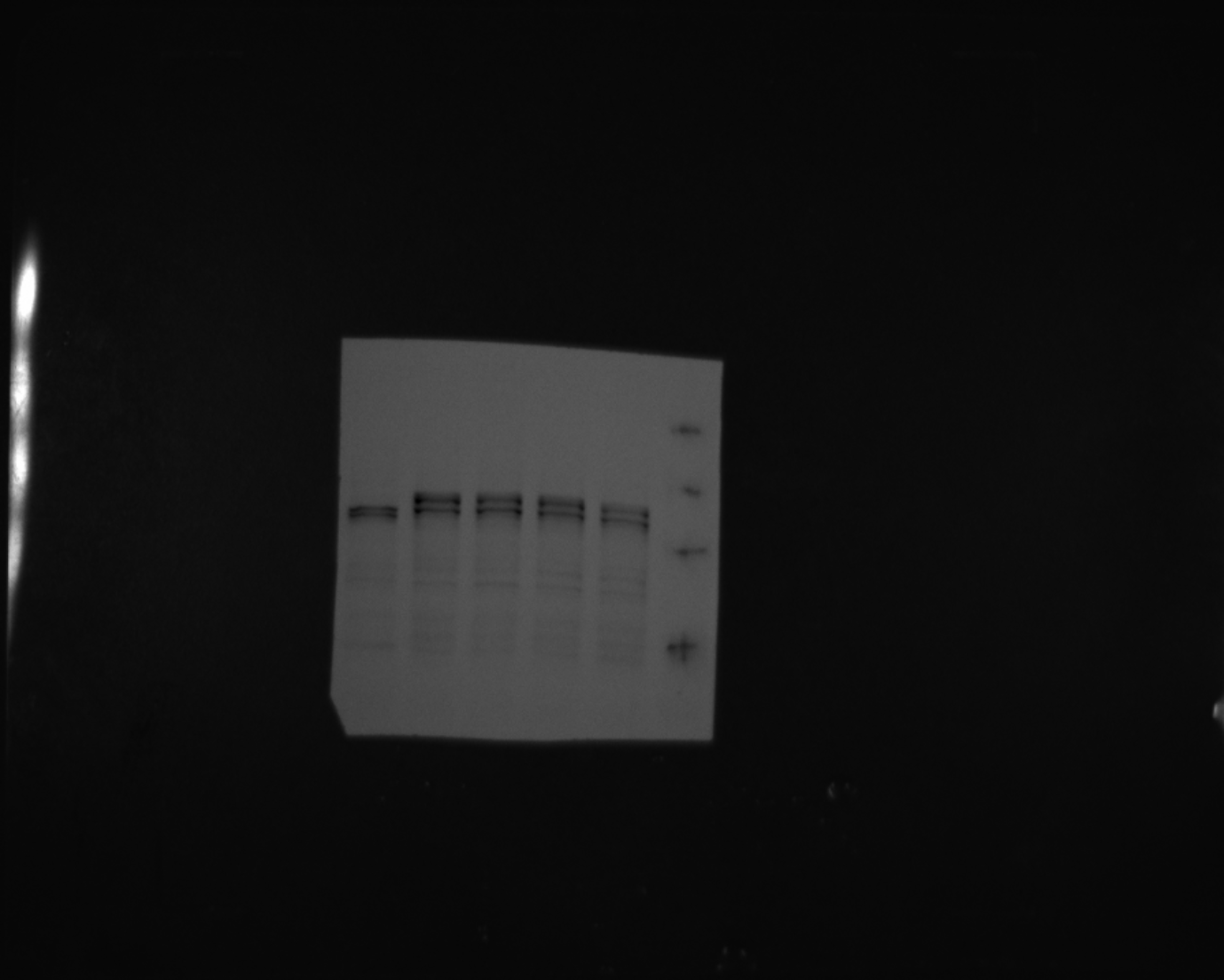

Supplement: Supplementary file 10 — Additional file 10. All Original and uncropped blots images used in manuscript. [file 12915_2022_1437_MOESM10_ESM.zip › blots images/Fig 7/Fig 7 C/HURP/Fig7C-HURP-marker.tif]

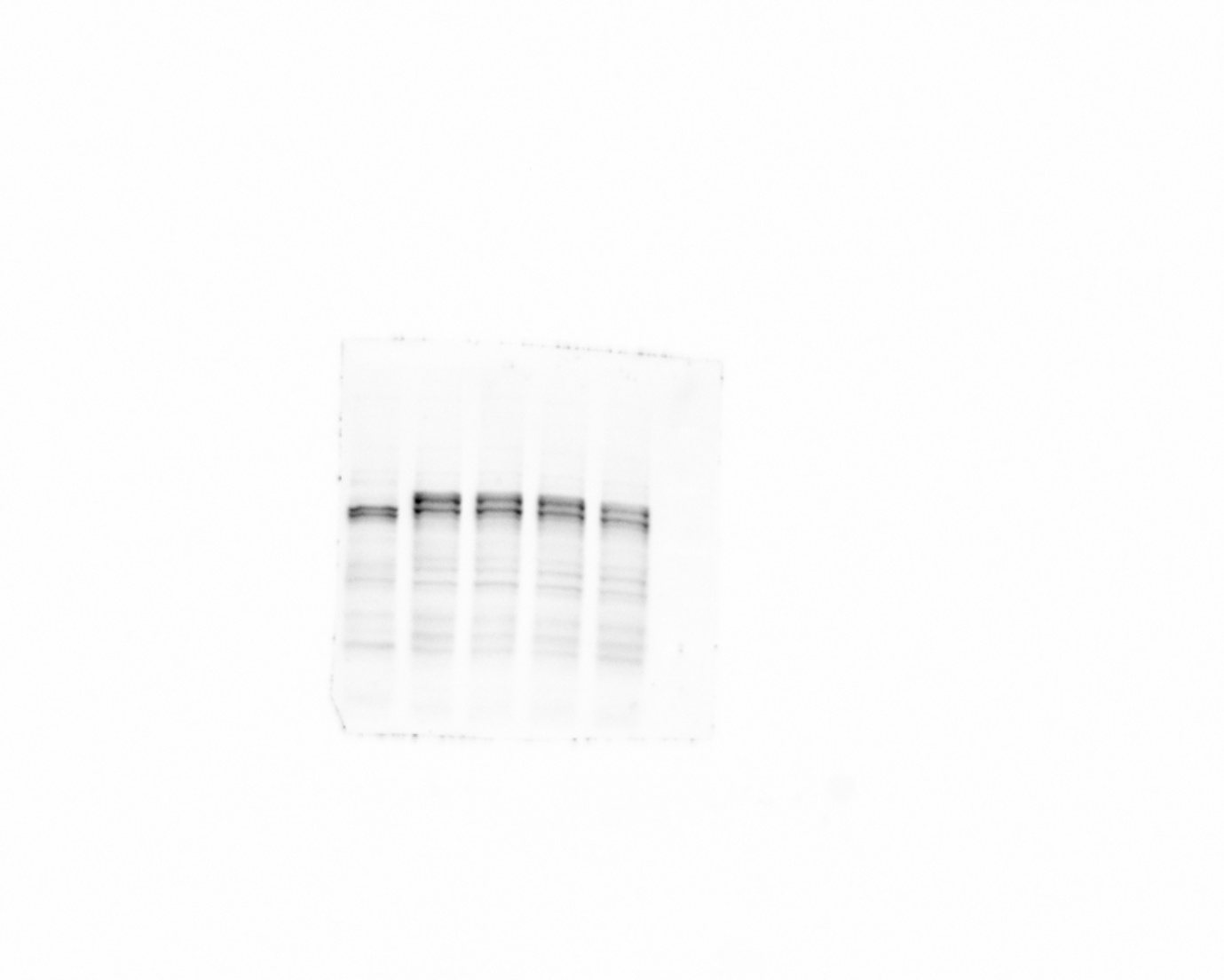

Supplement: Supplementary file 10 — Additional file 10. All Original and uncropped blots images used in manuscript. [file 12915_2022_1437_MOESM10_ESM.zip › blots images/Fig 7/Fig 7 C/HURP/Fig7C-HURP.tif]

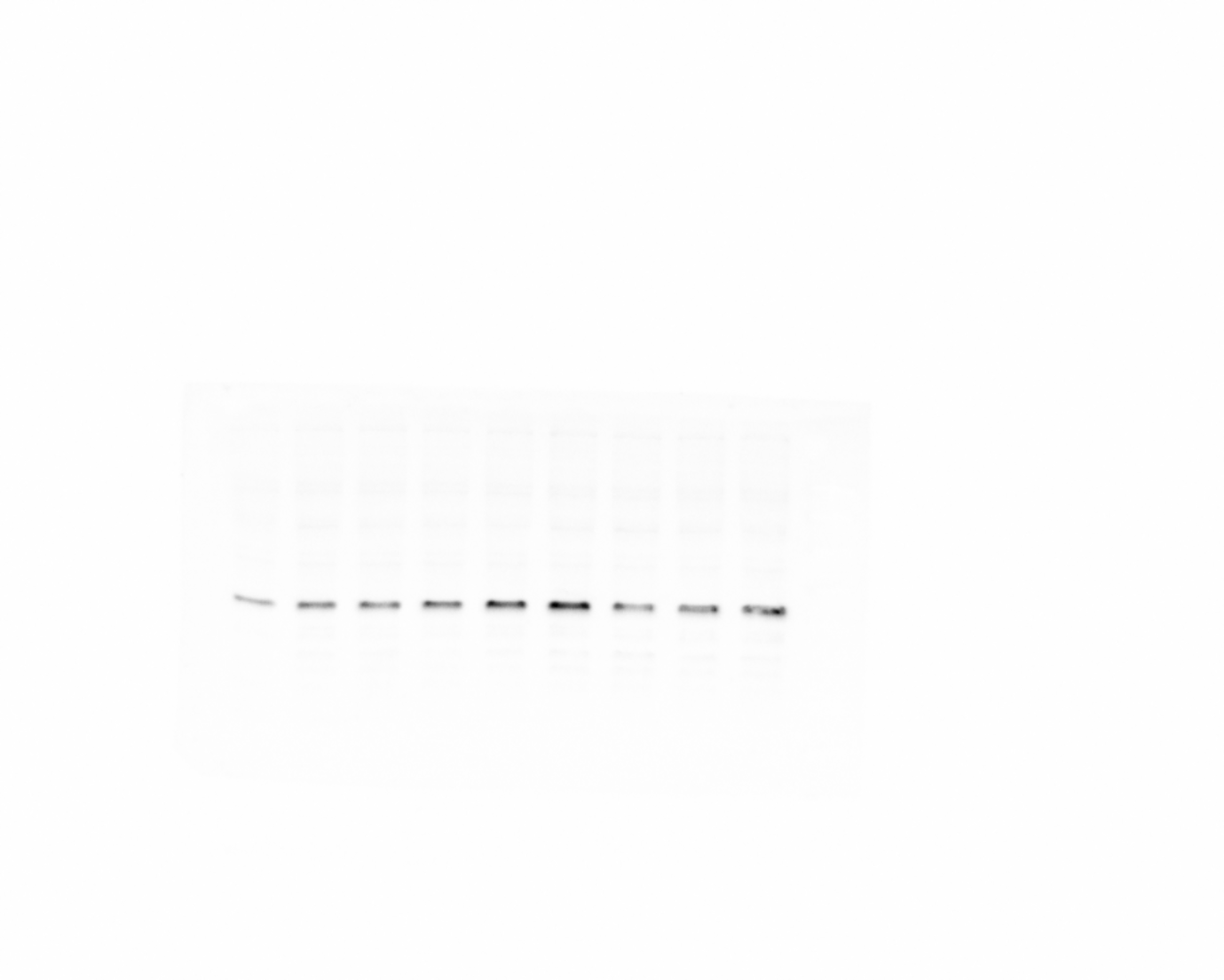

Supplement: Supplementary file 10 — Additional file 10. All Original and uncropped blots images used in manuscript. [file 12915_2022_1437_MOESM10_ESM.zip › blots images/Fig 7/Fig 7 C/Securin/FIg7C-lane5-9-siRNA-ecurin-used-in-paper.tif]

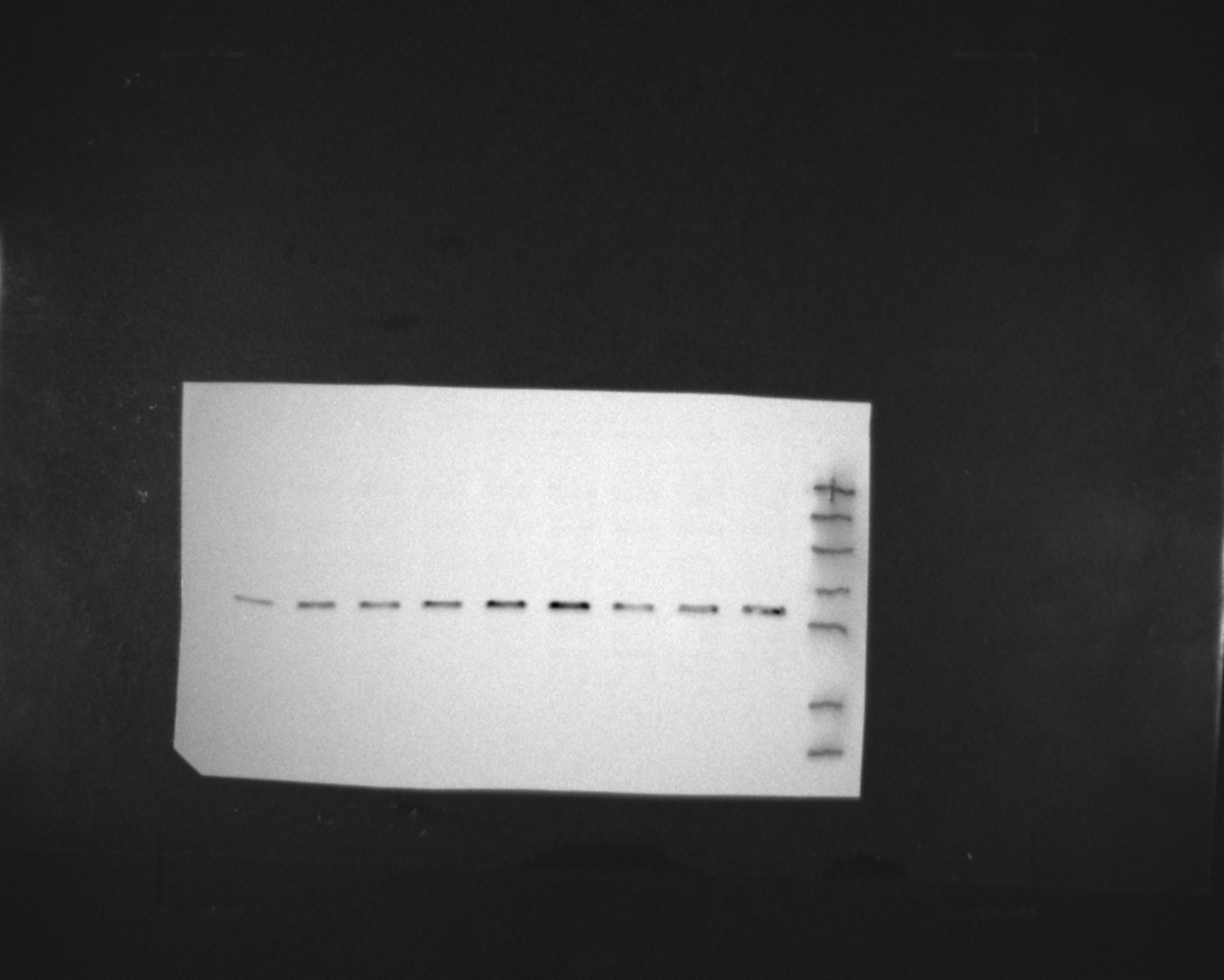

Supplement: Supplementary file 10 — Additional file 10. All Original and uncropped blots images used in manuscript. [file 12915_2022_1437_MOESM10_ESM.zip › blots images/Fig 7/Fig 7 C/Securin/Fig7C-lane5-9-siRNA-securin-marker-used-in-paper.tif]

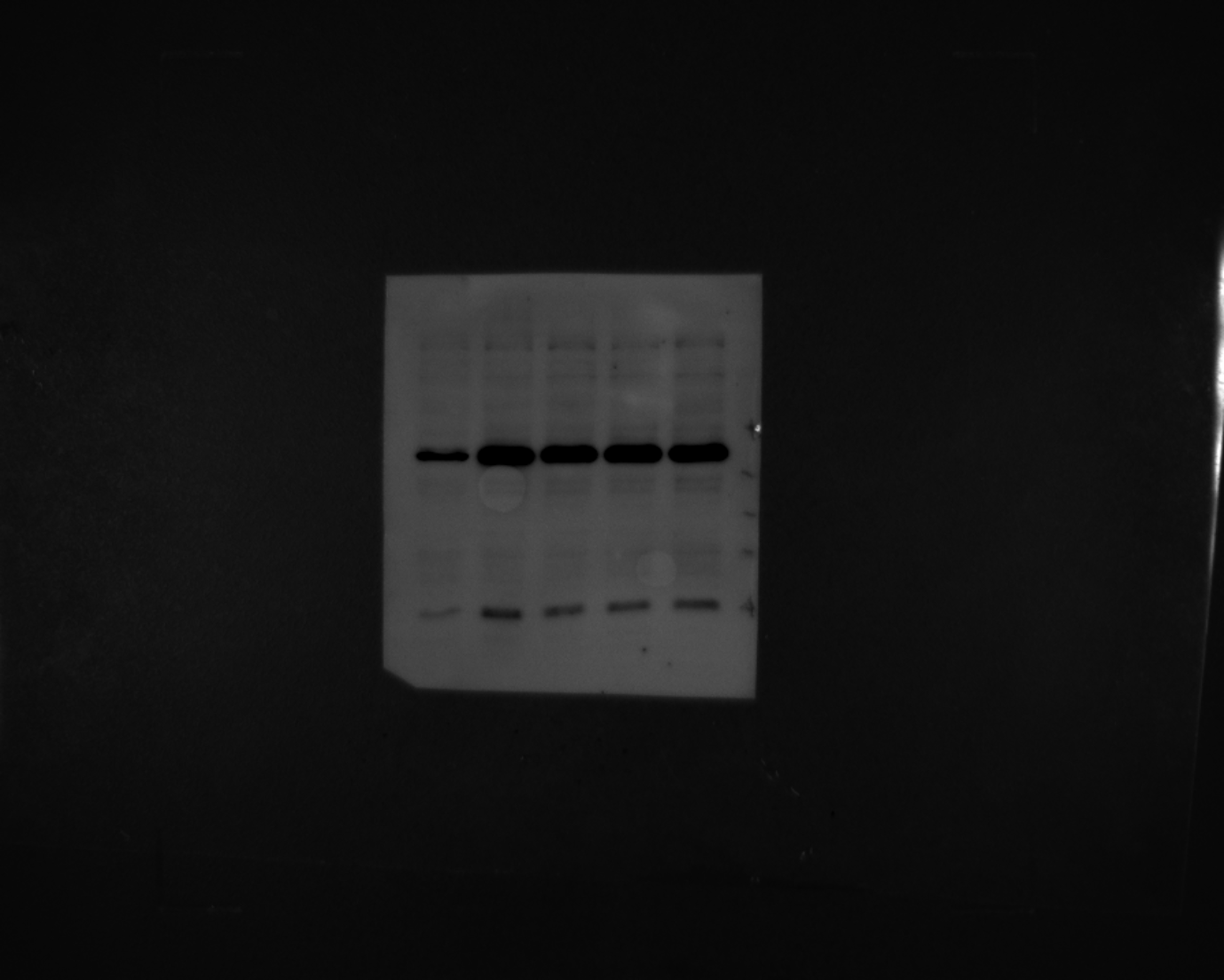

Supplement: Supplementary file 10 — Additional file 10. All Original and uncropped blots images used in manuscript. [file 12915_2022_1437_MOESM10_ESM.zip › blots images/Fig 7/Fig 7 C/Securin/siRNA-anti-securin-not-used-in-paper-redo-2-marker.tif]

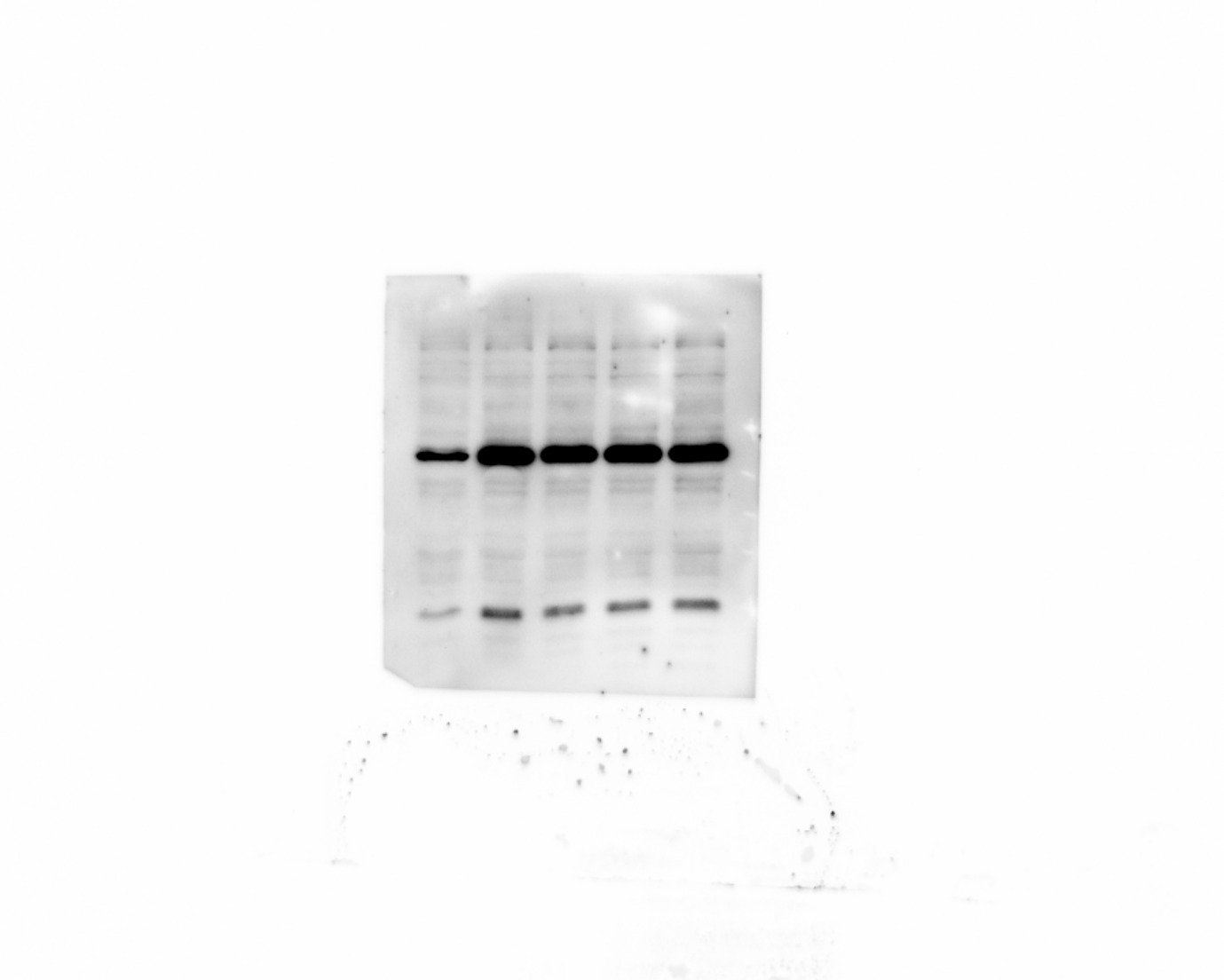

Supplement: Supplementary file 10 — Additional file 10. All Original and uncropped blots images used in manuscript. [file 12915_2022_1437_MOESM10_ESM.zip › blots images/Fig 7/Fig 7 C/Securin/siRNA-anti-securin-not-used-in-paper-redo-2.tif]

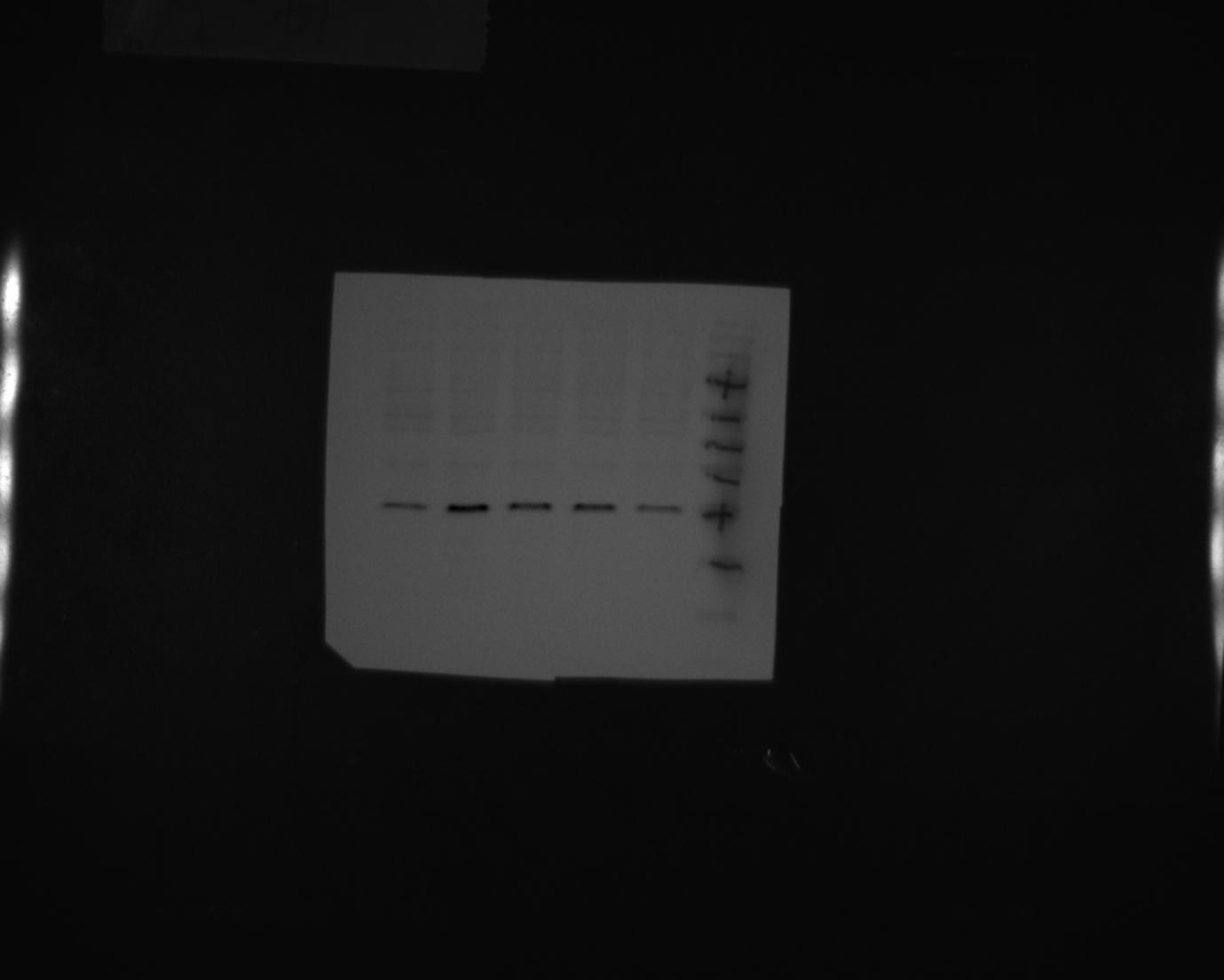

Supplement: Supplementary file 10 — Additional file 10. All Original and uncropped blots images used in manuscript. [file 12915_2022_1437_MOESM10_ESM.zip › blots images/Fig 7/Fig 7 C/Securin/siRNA-anti-securin-not-used-in-paper-redo-marker.tif]

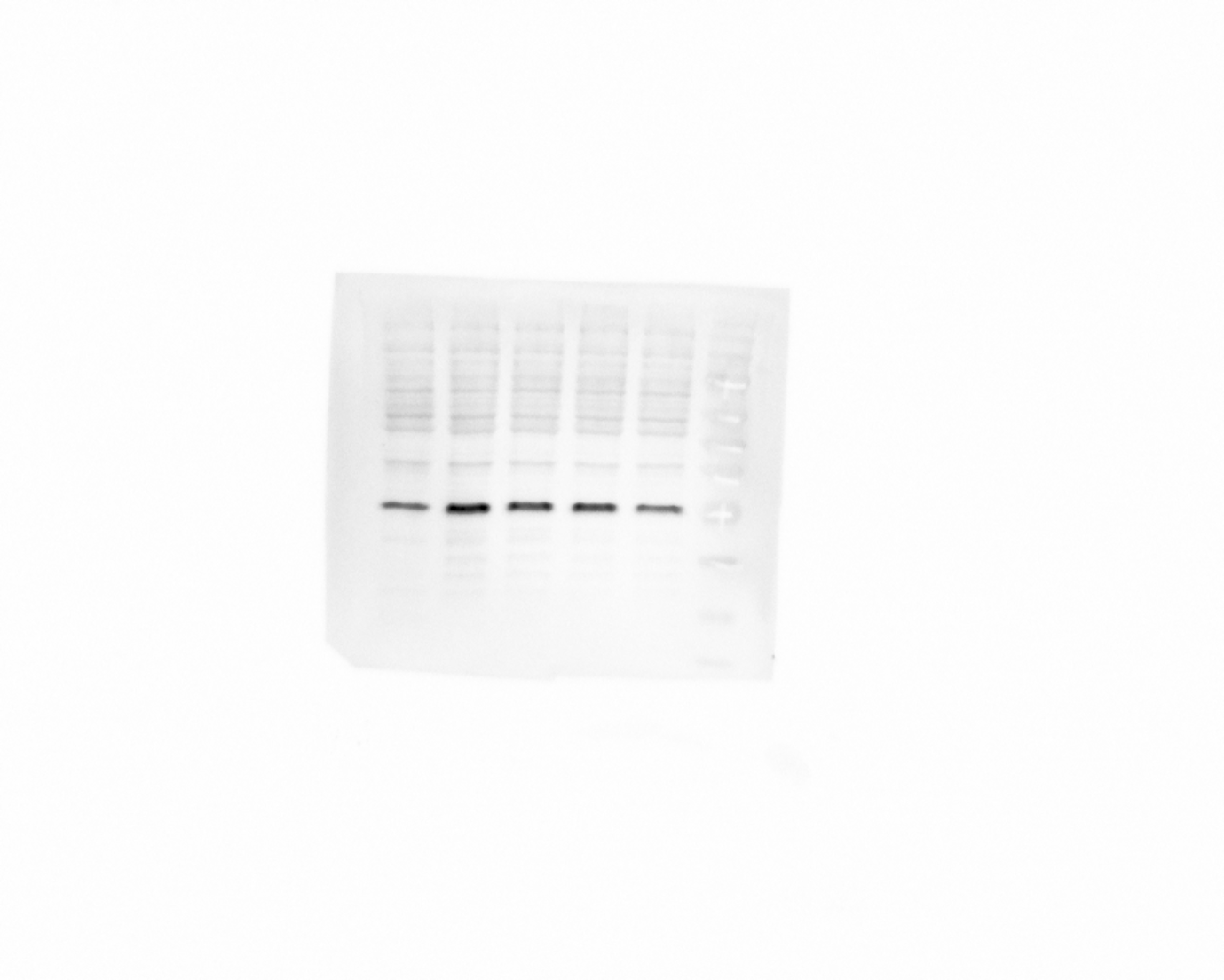

Supplement: Supplementary file 10 — Additional file 10. All Original and uncropped blots images used in manuscript. [file 12915_2022_1437_MOESM10_ESM.zip › blots images/Fig 7/Fig 7 C/Securin/siRNA-anti-securin-not-used-in-paper-redo.tif]

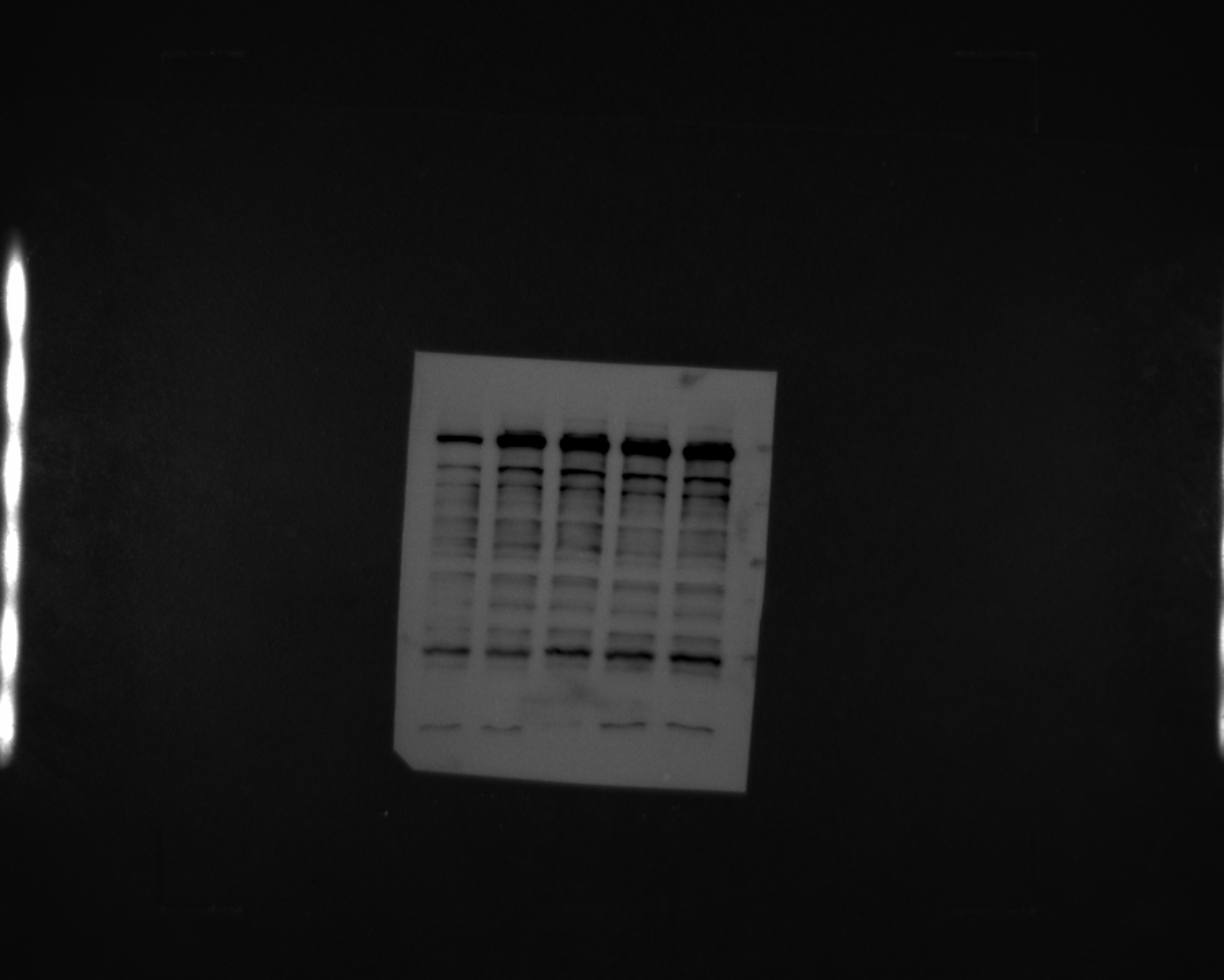

Supplement: Supplementary file 10 — Additional file 10. All Original and uncropped blots images used in manuscript. [file 12915_2022_1437_MOESM10_ESM.zip › blots images/Fig 7/Fig 7 C/Separase-and-Separase-cleaved/Fig7C-separase-marker.tif]

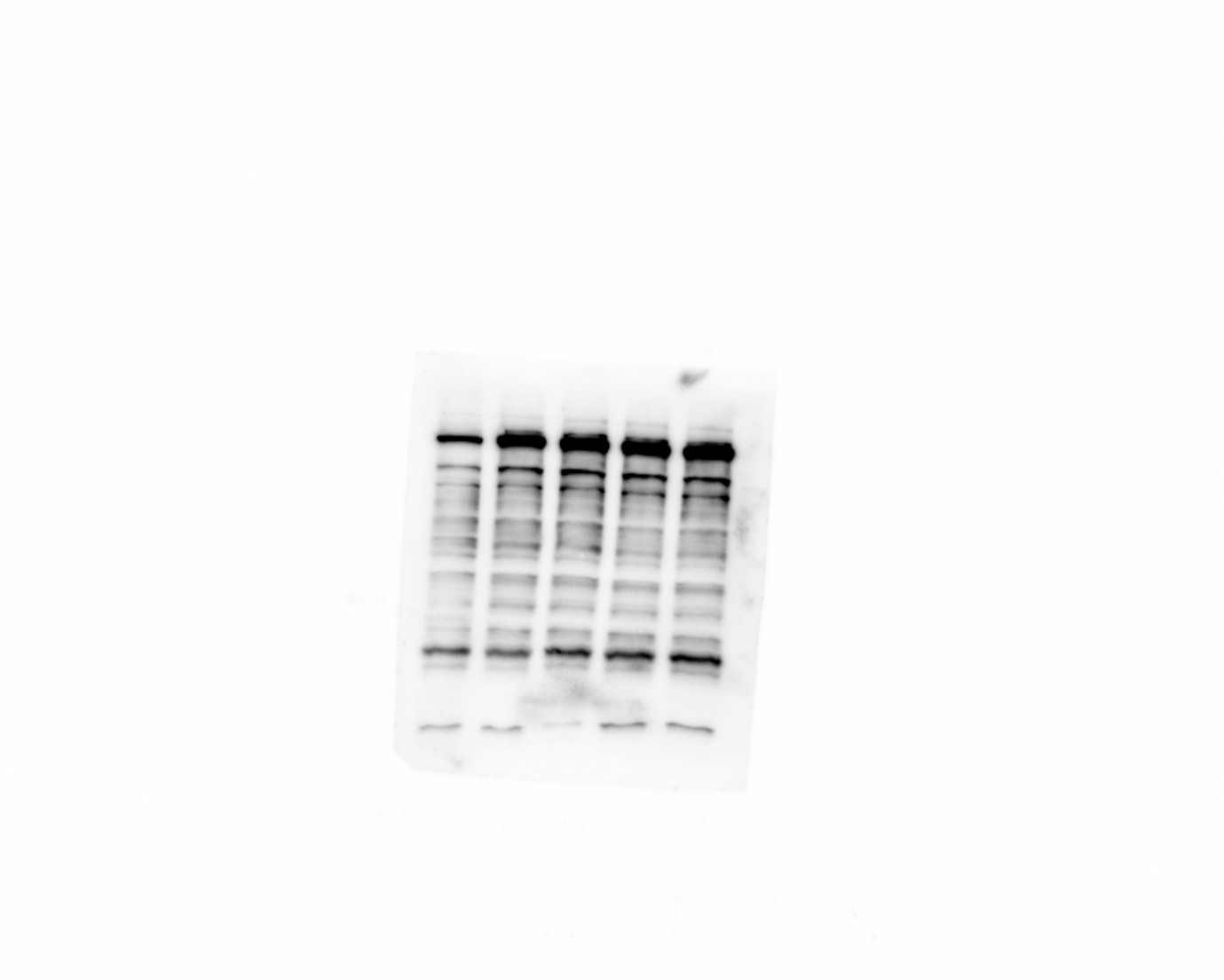

Supplement: Supplementary file 10 — Additional file 10. All Original and uncropped blots images used in manuscript. [file 12915_2022_1437_MOESM10_ESM.zip › blots images/Fig 7/Fig 7 C/Separase-and-Separase-cleaved/Fig7C-separase.tif]

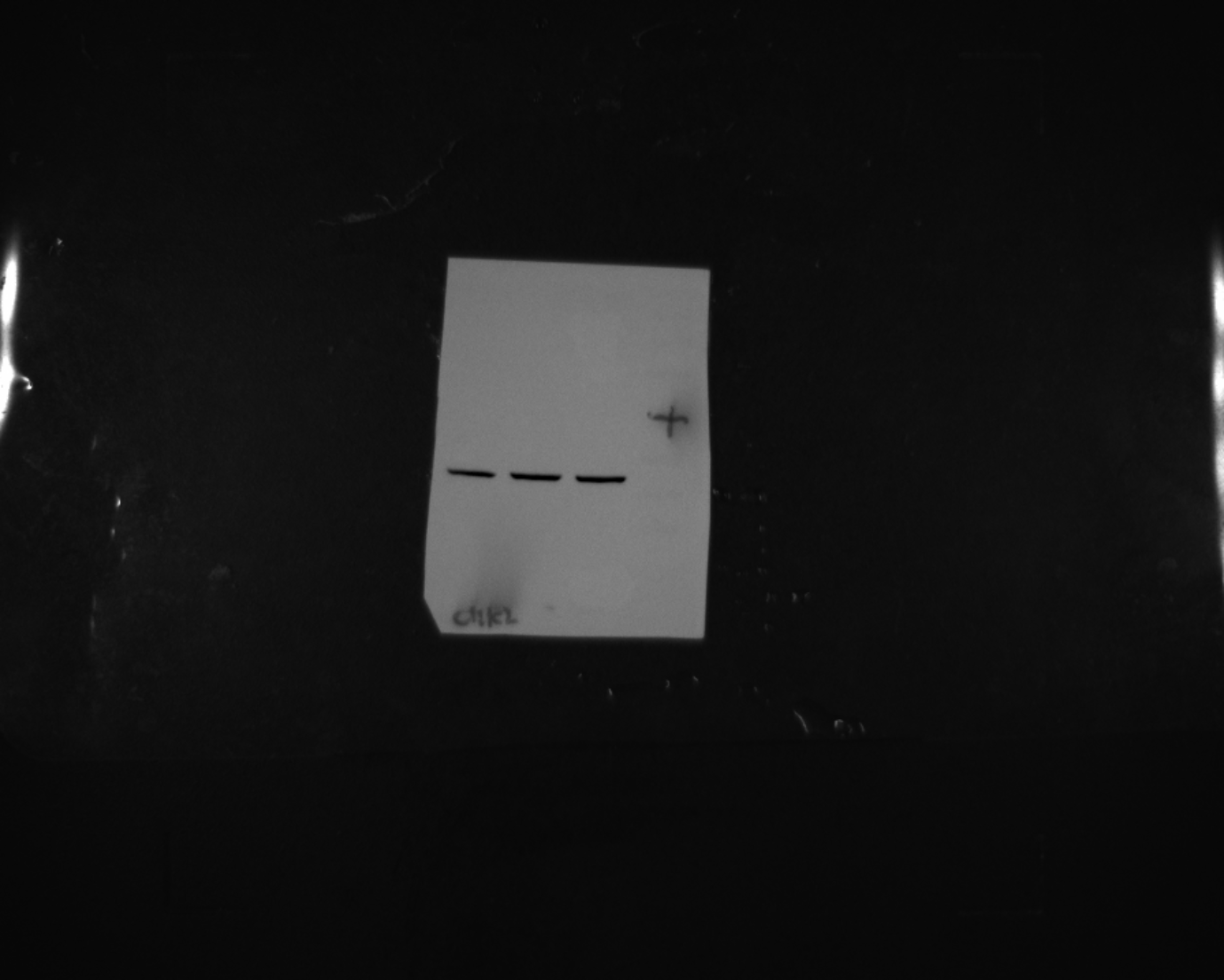

Supplement: Supplementary file 10 — Additional file 10. All Original and uncropped blots images used in manuscript. [file 12915_2022_1437_MOESM10_ESM.zip › blots images/Fig 7/Fig 7 G/beta-actin/Fig7G-beta-actin-marker.tif]

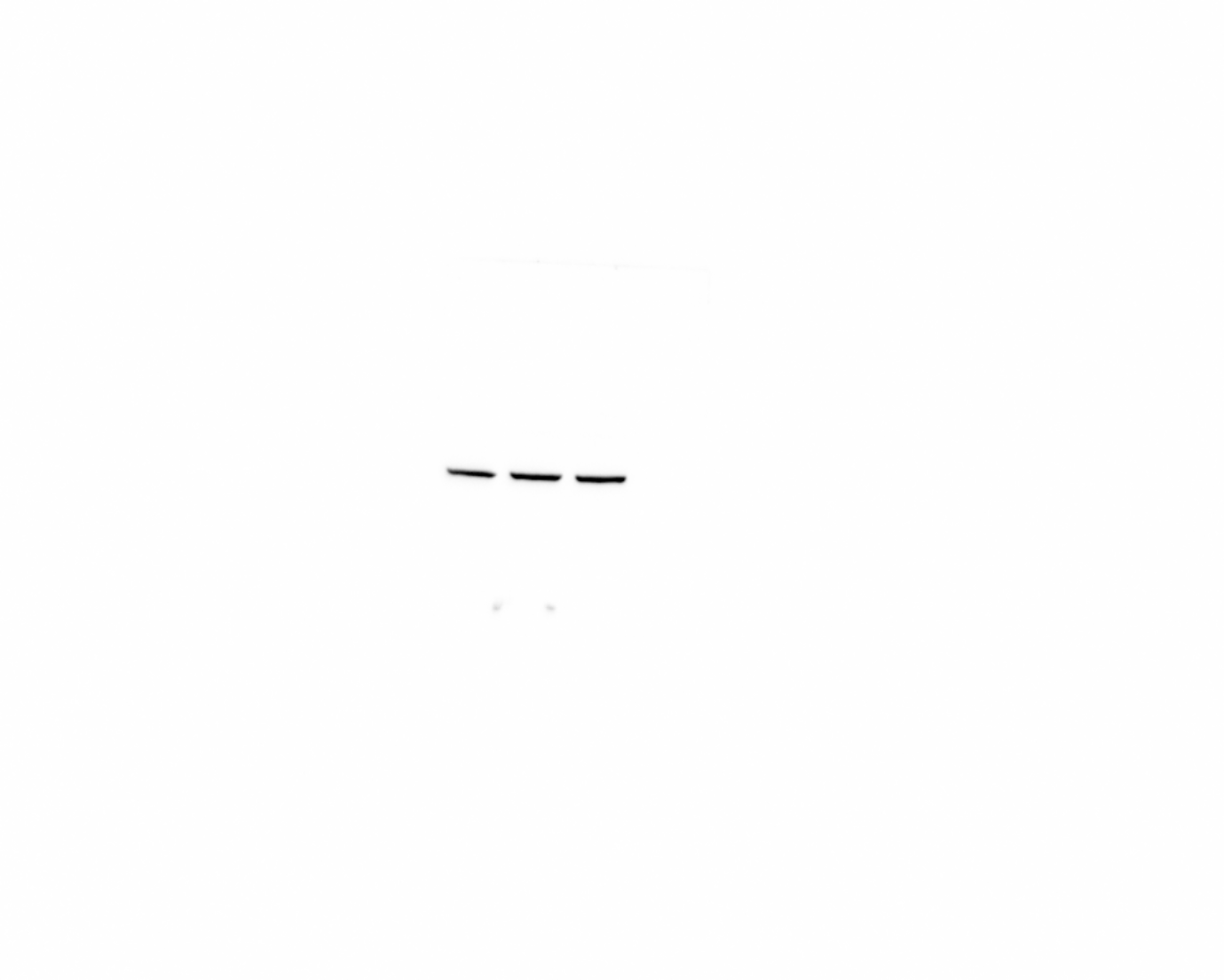

Supplement: Supplementary file 10 — Additional file 10. All Original and uncropped blots images used in manuscript. [file 12915_2022_1437_MOESM10_ESM.zip › blots images/Fig 7/Fig 7 G/beta-actin/Fig7G-beta-actin.tif]

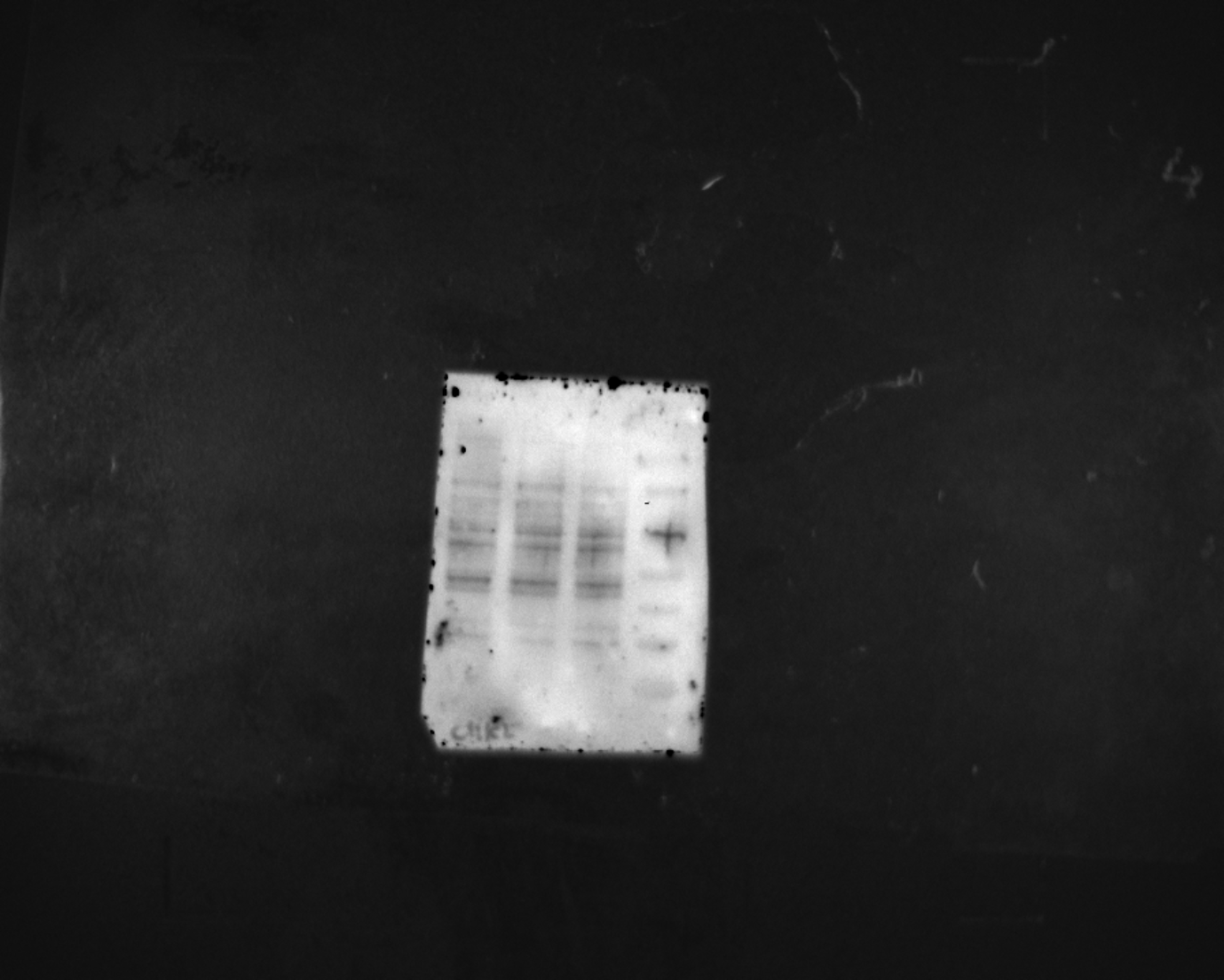

Supplement: Supplementary file 10 — Additional file 10. All Original and uncropped blots images used in manuscript. [file 12915_2022_1437_MOESM10_ESM.zip › blots images/Fig 7/Fig 7 G/chk2/Fig7G-CHK2-marker.tif]

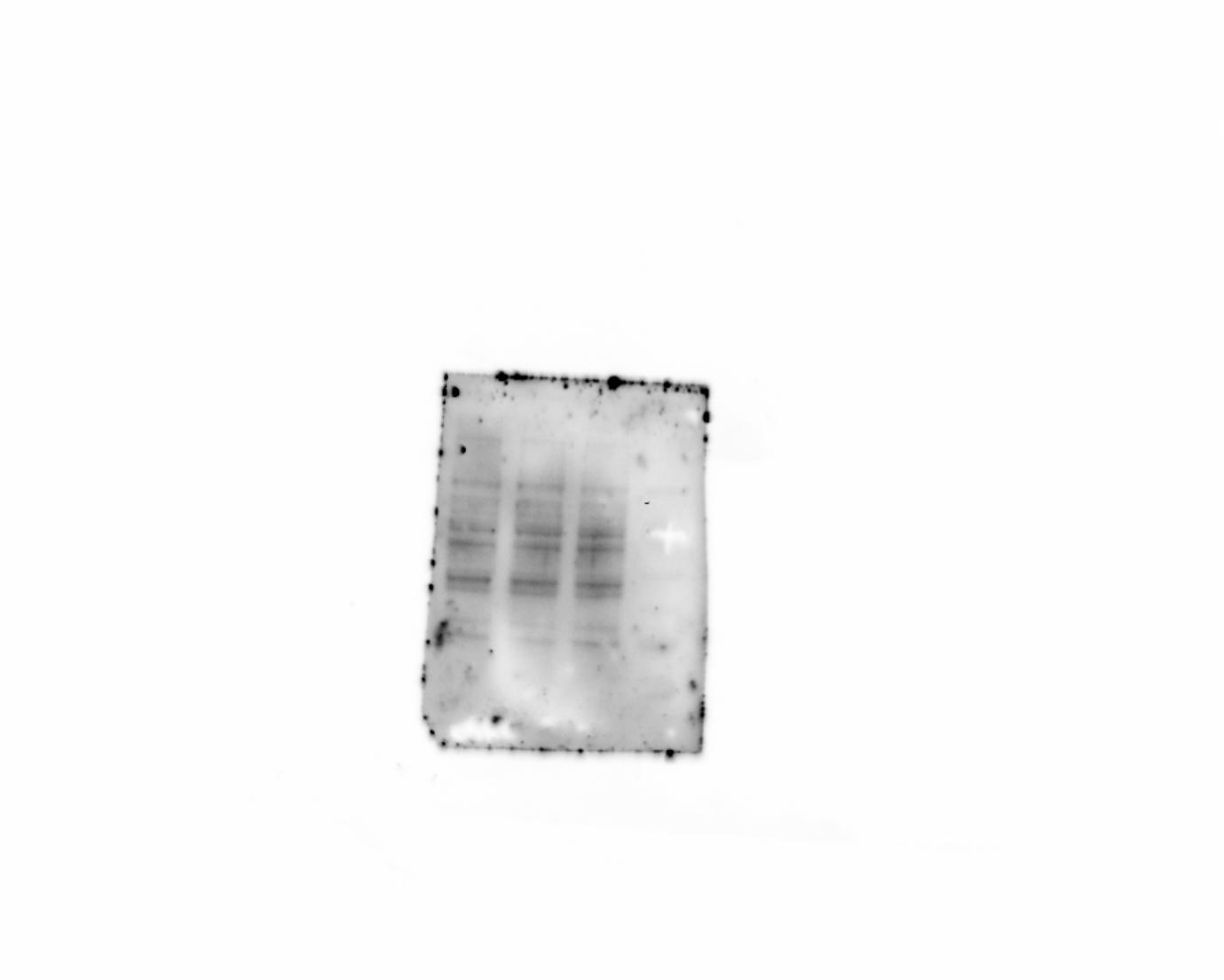

Supplement: Supplementary file 10 — Additional file 10. All Original and uncropped blots images used in manuscript. [file 12915_2022_1437_MOESM10_ESM.zip › blots images/Fig 7/Fig 7 G/chk2/Fig7G-CHK2.tif]

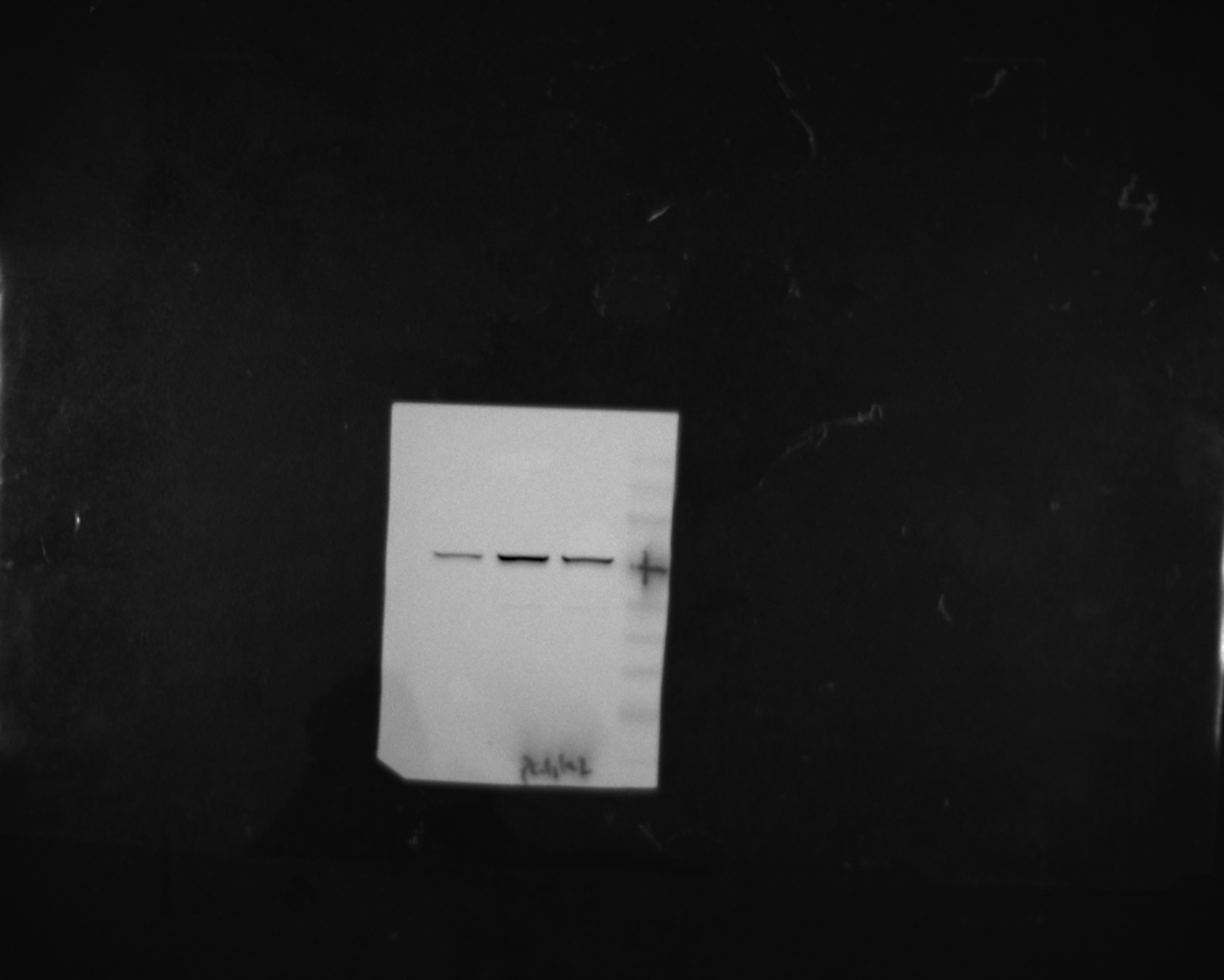

Supplement: Supplementary file 10 — Additional file 10. All Original and uncropped blots images used in manuscript. [file 12915_2022_1437_MOESM10_ESM.zip › blots images/Fig 7/Fig 7 G/pChk2/Fig7G-pCHK2-marker.tif]

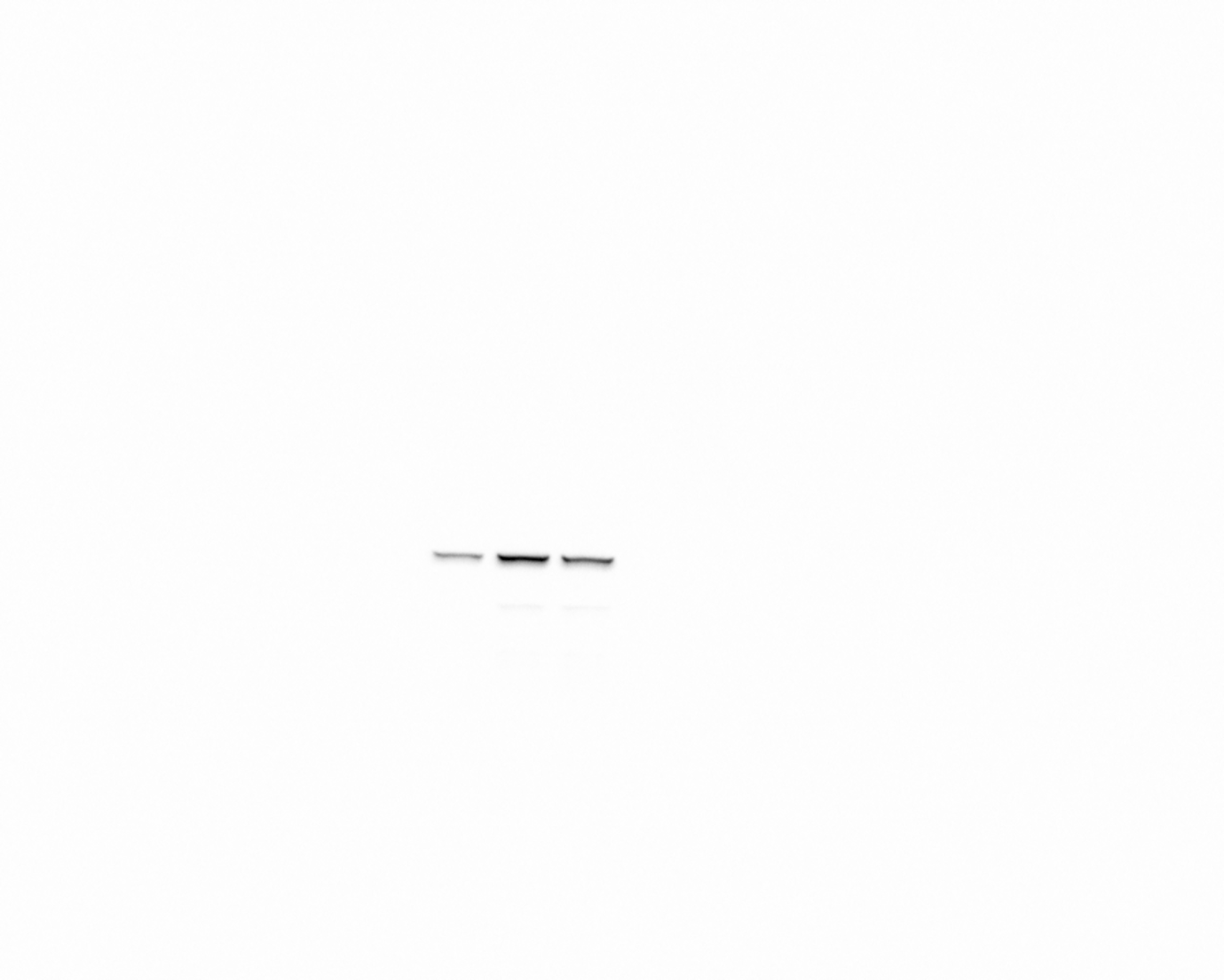

Supplement: Supplementary file 10 — Additional file 10. All Original and uncropped blots images used in manuscript. [file 12915_2022_1437_MOESM10_ESM.zip › blots images/Fig 7/Fig 7 G/pChk2/Fig7G-pCHK2.tif]
